# CD11b agonist GB1275 potentiates oncolytic bacterial therapy against pancreatic cancer in mouse tumor models

**DOI:** 10.1038/s41467-026-76823-7

**Published:** 2026-08-21

**Authors:** Bin Zhang, Meng Guan, Xiao-Ting Xie, Jia-Hua Zou, Kai Cheng, Yuan-Di Zhao, Bo Liu, Jin-Xuan Fan, Qiu-Ran Xu

**Affiliations:** 1 MOE Key Laboratory for Biomedical Photonics - Hubei Bioinformatics & Molecular Imaging Key Laboratory, Department of Biomedical Engineering, College of Life Science and Technology, Huazhong University of Science and Technology, Wuhan, Hubei PR China; 2 Department of Oncology, Huanggang Central Hospital of Yangtze University, Huanggang City, Hubei PR China; 3 Hubei Clinical Medical Research Center of Esophageal and Gastric Malignancy, Huanggang City, Hubei PR China; 4 Department of Chemistry, The Chinese University of Hong Kong, Hong Kong, Hong Kong SAR PR China; 5 Zhejiang Key Laboratory of Tumor Molecular Diagnosis and Individualized Medicine, Zhejiang Provincial People’s Hospital, Affiliated People’s Hospital, Hangzhou Medical College, Hangzhou, Zhejiang PR China

**Keywords:** Cancer immunotherapy, Bacterial techniques and applications, Tumour immunology, Pattern recognition receptors

## Abstract

Oncolytic bacteria are microorganisms with cytotoxic activity capable of lysing cancer cells while simultaneously activating both innate and adaptive immune responses to exert anti-tumor effects. However, the innate immune activation targeting oncolytic bacteria within the tumor microenvironment (TME) is a limiting factor. Here we find that the CD11b-targeting agonist GB1275 can modulate bacteria-immune interaction and inhibit the infiltration of CD11b⁺ innate immune cells such as neutrophils and macrophages, thereby suppressing the antimicrobial immune defenses in the TME in mice. This then avoids the clearance of oncolytic bacteria and increases the efficacy of bacteria-mediated tumor immunotherapy. Furthermore, the gemcitabine-loaded bacteria combined with GB1275 treatment activate innate immune responses in tumor, as mediated by Toll-like receptors (TLR), NOD-like receptors (NLR) and cGAS-STING pathways, while simultaneously enhancing dendritic cell (DC)-dependent antigen cross-presentation; this then upregulates the adaptive anti-tumor immune response and inhibits the growth of both subcutaneous and in situ pancreatic cancers in mouse tumor models. Our results therefore demonstrate that modulating innate immune responses while subsequently strengthening adaptive immune activation can significantly improve the efficacy of bacteria-mediated tumor immunotherapy.

## Abstract (web summary)

Cancer immune therapy by oncolytic bacteria triggers immune cell activation against the tumors, but the therapeutic effect may be compromised by ant-bacterial immune activity. Here the authors combine oncolytic bacteria with a CD11b agonist that alters the composition of tumor-infiltrating myeloid cells in mouse models to favor anti-tumor innate activation while preventing clearance of the bacteria.

## Introduction

Tumor immunotherapy can be traced back to the 19th century, when in the 1890s William Coley administered heat-inactivated *Streptococcus* and *Serratia marcescens* (Coley’s toxins) into malignant tumor tissues and observed tumor regression, thus pioneering the field of tumor immunotherapy^1,2^. Modern tumor immunotherapy aims to activate or reshape the body’s immune system to combat malignant tumors, and has demonstrated transformative potential in the clinic^3,4^. From immune checkpoint inhibitors to CAR-T cell therapy, these strategies have significantly improved the survival benefits of some patients by targeting immune regulatory nodes^5,6^. However, the complex immunosuppressive microenvironment of solid tumors, such as low antigen presentation efficiency, immune cell infiltration disorders and the continuous activation of inhibitory signaling pathways, remains challenges for current therapies^7–9^. In recent years, driven by the rapid development of immunology and synthetic biology, bacteria-mediated tumor immunotherapy has received widespread attention due to its unique tumor-targeting capability and immune-activating ability^10–13^. Studies have shown that certain facultative anaerobic bacteria possess the natural ability to target and colonize the hypoxic microenvironment of tumors, and their biological activity and dynamic interaction with the host immune system constitute a unique anti-tumor mechanism^14,15^. The oncolytic effect of bacteria relies not only on directly killing tumor cells, but is more attributed to rapidly triggered innate and adaptive immune responses. After colonizing the tumor microenvironment (TME), bacteria on the one hand directly activate the innate immune system and recruit innate immune cells, such as neutrophils, macrophages and natural killer (NK) cells, reprogramming the originally immunosuppressive “cold tumor” microenvironment into an immune-activated “hot tumor” state^16–18^; on the other hand, metabolites or toxins released by bacteria can directly kill tumor cells, while simultaneously inducing systemic adaptive immune responses^19–22^.

However, it is important to note that while activating the innate immune system, bacteria also face the risk of immune-mediated clearance. Overactive neutrophils massively infiltrate tumor tissues, clearing bacteria and establishing an innate immune barrier within the TME^23,24^. Simultaneously, macrophages play a critical role in antibacterial defense through direct phagocytosis of bacteria and functional synergy with neutrophils. This innate immunity-dominated antimicrobial response, while effectively controlling infection risks, paradoxically constitutes a “self-limiting bottleneck” in tumor therapy^25,26^. Therefore, how to coordinate the dynamic balance between the anti-tumor activity of bacteria and the host immune response is a key factor for enhancing the efficacy of bacterial immunotherapy. This involves improving the survival and proliferative capacity of bacteria within the TME to ensure their effective activation of the host immune system while avoiding rapid clearance due to excessive immune responses^27–29^. Recent studies have shown that genetically engineered *Salmonella* can stimulate tumor-associated macrophages (TAM) to produce interleukin-10, thereby exerting a “paralyzing” effect on their predator neutrophils. This hinders neutrophil motility and allows bacteria to transiently evade immune surveillance, achieving dual regulation of immune escape and anti-tumor immunity^30^. However, to fully unlock the therapeutic potential of bacteria, there is an urgent need to overcome the antimicrobial defense systems within the TME, particularly by intervening in the innate immune-mediated immune clearance mechanisms, in order to establish a more favorable immune microenvironment conducive to bacterial colonization and the exertion of their anti-tumor functions.

GB1275, a novel oral CD11b allosteric agonist, exerts its biological effects by stabilizing the active conformation of CD11b, with its activation independent of integrin saturation. GB1275 enhances CD11b⁺ cell adhesion both in vitro and in vivo, and effectively diminishes the migration of macrophages and neutrophils to lesion sites in multiple animal models of inflammation and tumors^31,32^. Numerous studies have further validated its anti-tumor potential. GB1275 not only remodels the TME and decreases myeloid cell infiltration into tumors, but also reshapes the phenotype of TAMs by activating interferon-related gene expression, thereby highlighting its promising prospects in combination therapeutic strategies^33,34^. At present, GB1275 has entered Phase I/II clinical trials (NCT04060342). Based on preclinical evidence demonstrating its ability to regulate the immunosuppressive TME, the trials are evaluating its monotherapy regimen as well as combination regimens with pembrolizumab and the chemotherapeutic regimen (nab-paclitaxel plus gemcitabine), in multiple malignancies including locally advanced or metastatic pancreatic cancer and colorectal cancer^35^. Preliminary data have shown that GB1275 exhibits low treatment-related toxicity and favorable safety characteristics. Given its excellent immunomodulatory activity, well-defined mechanism of action and promising clinical translation potential, we hypothesize that GB1275 can enhance bacteria-mediated anti-tumor immunotherapy by reducing the abnormal infiltration of CD11b⁺ cells, including neutrophils and macrophages, into tumor tissues.

In this study, we investigate the therapeutic combination of GB1275 and drug (gemcitabine)‑loaded bacteria for pancreatic cancer. GB1275 restricts intratumoral CD11b⁺ myeloid infiltration, leading to increased bacterial abundance, which in turn drives tumor necrosis and ultimately potentiates oncolysis. This combinatorial approach remodels the pancreatic tumor microenvironment (TME), converting immunologically “cold” tumors into “hot” ones. Mechanistically, it activates TLR, NLR, and cGAS‑STING innate immune signaling, thereby initiating adaptive immunity. Meanwhile, the combination induces tumor cell lysis, releasing tumor antigens that promote DC‑mediated tumor‑specific T cell responses. The regimen elicits potent anti-tumor activity in both subcutaneous and orthotopic pancreatic cancer models, covering different subtypes. Collectively, our work demonstrates that coordinated modulation of the bacteria–innate–adaptive immune axis optimizes bacterial‑based tumor immunotherapy.

## Results

### The intratumoral colonization of oncolytic bacteria recruits CD11b⁺ innate immune cells

**Fig. 1:**
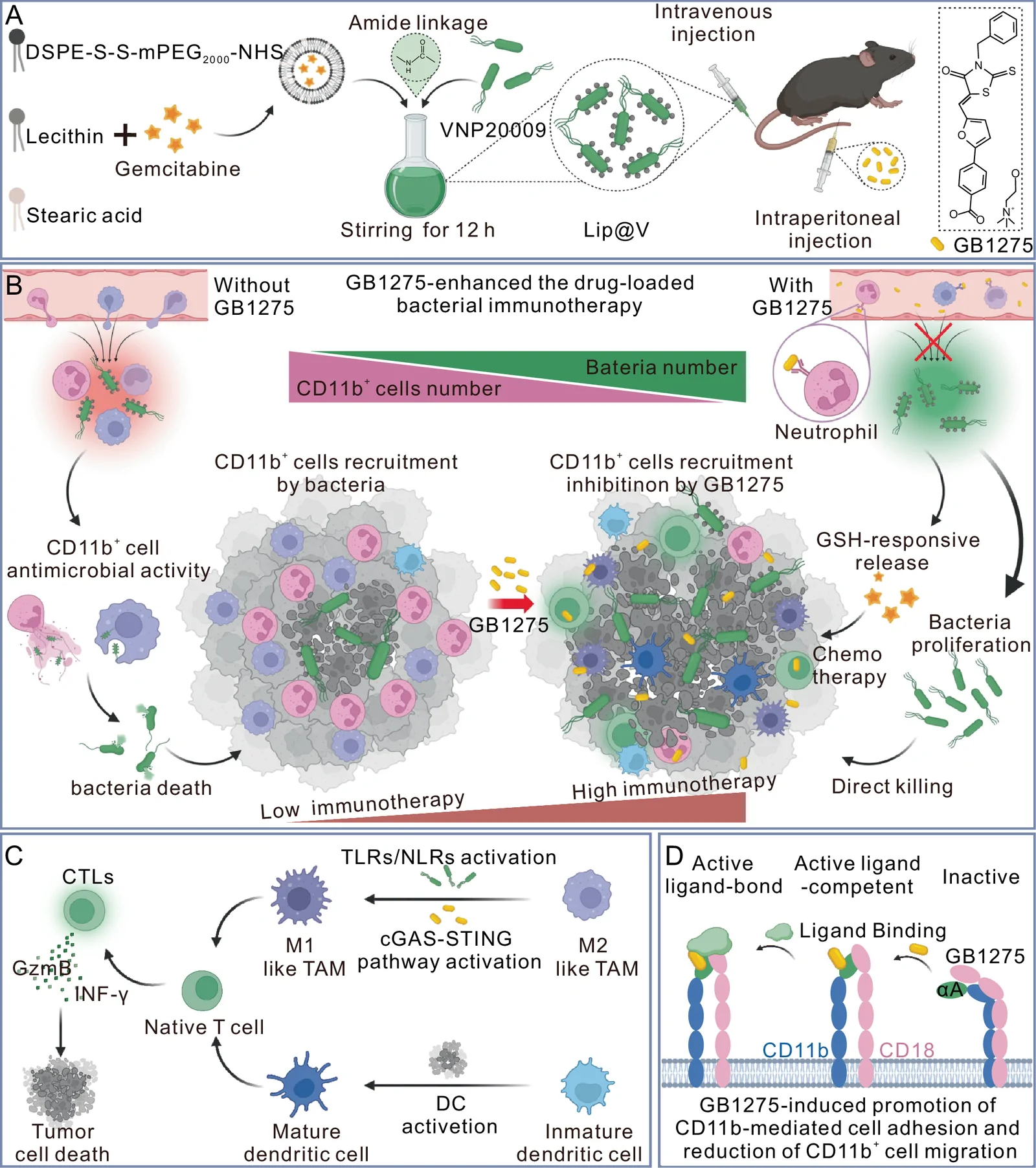
Schematic diagram of GB1275-enhanced gemcitabine-loaded bacteria anti-tumor effect. **A** Construction of gemcitabine-loaded oncolytic bacteria (Lip@V). **B** Principle of GB1275-enhanced Lip@V anti-tumor immunotherapy. **C** Activation of adaptive immune pathways by GB1275 in combination with Lip@V. **D** Promotion of CD11b-mediated cell adhesion and reduction of CD11b⁺ cell migration by GB1275. **A**–**D** These were created with BioRender.com. (Zhang, B. (2026) https://BioRender.com/kh7fesr).

**Fig. 2:**
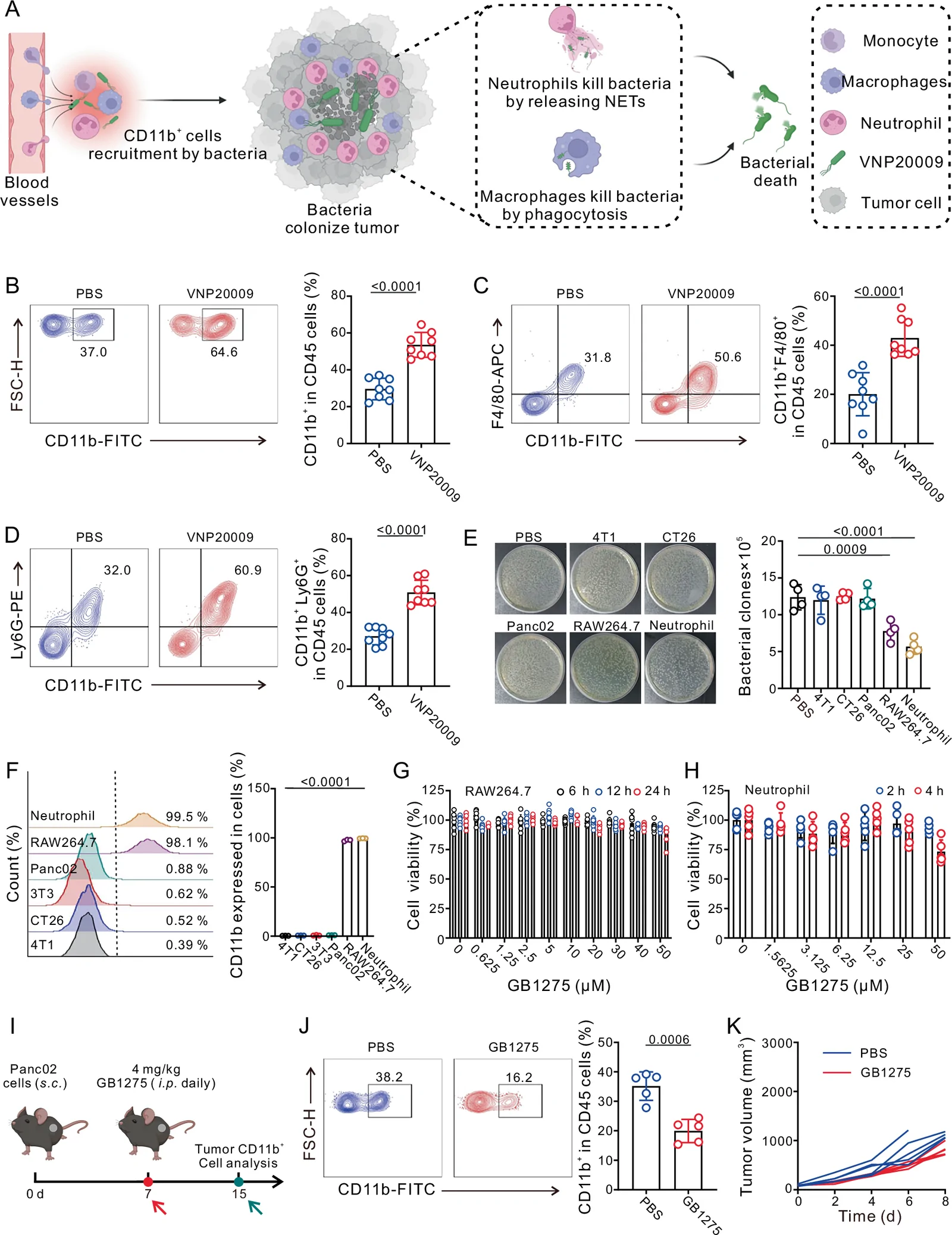
The intratumoral colonization by oncolytic bacteria recruits a large number of CD11b⁺ innate immune cells. **A** Schematic illustration of intratumoral bacterial colonization inducing CD11b⁺ innate immune cell excessive infiltration, impairing bacterial anti-tumor efficacy via immune clearance. CD11b is expressed on various innate immune cells, including monocytes, macrophages, and neutrophils, and plays a critical role in mediating cell adhesion, transendothelial migration, and other processes. NETs is the abbreviation for Neutrophil Extracellular Traps. **B**–**D** Flow cytometry analysis and corresponding statistics of the total CD11b⁺ cells, macrophages, and neutrophils in the tumor tissue after bacteria administration at 48 h. 5 × 10^6^ CFU of VNP20009 was administered intravenously via the tail vein, while the control group received PBS, *n* = 8 independent samples. **E** Representative photographs and corresponding quantification of bacteria incubated with different cells for 24 h, *n* = 4 independent samples. **F** Flow cytometry analysis and corresponding statistics of the expression levels of CD11b in different cells, *n* = 3 independent samples. **G**, **H** Cytotoxic effects of GB1275 on macrophages and neutrophils at indicated concentrations and time points, assessed by the CCK-8 assay. *n* = 8 independent samples in (**G**); *n* = 5 independent samples in (**H**). **I** Schematic illustration of the in vivo administration regimen of GB1275 in Panc02 subcutaneous tumor model; PBS administered in the same amount was taken as a control group. *s.c*., subcutaneous injection. *i.p*. daily, daily intraperitoneal injection. **J** Flow cytometry analysis and corresponding statistics of CD11b⁺ cells in tumor tissues on day 8 after the administration of GB1275, *n* = 5 independent samples. **K** Tumor growth curve during the administration of GB1275, *n* = 5 independent samples. The data are expressed as mean ± s.d. All replicates are biological, and the *P* values in (**B**–**D**, **J**) were determined using two-tailed Student’s *t* test. the *P* in (**E**, **F**) were determined using one-way ANOVA followed by Tukey’s multiple comparisons test. Source data are provided as a Source Data file. **A**, **I** These were created in BioRender. Zhang, B. (2026) https://BioRender.com/th7zath.

VNP20009, a genetically engineered *Salmonella typhimurium* strain designed for tumor-targeted therapy, was selected as the model strain in this study (Fig. 1). Although the Phase I clinical trials of VNP20009 failed to achieve the expected anti-tumor efficacy, crucially, this strain demonstrated favorable in vivo biosafety, indicating its potential as a relatively safe platform for further research and development^36^. The administration of bacterial agents triggers highly complex host immune responses, primarily involving the innate immune system’s rapid recognition and clearance of the bacteria. After successfully colonizing, the bacteria further induce the recruitment of innate immune cells into the tumor (Fig. 2A). Given that macrophages and neutrophils both express the CD11b surface molecule, we first analyzed the infiltration of CD11b⁺ cells, macrophages and neutrophils in tumor tissues on day 3 after bacterial administration in the Panc02 pancreatic cancer subcutaneous tumor mouse model via flow cytometry (Supplementary Fig. 1). Results demonstrated that bacterial treatment significantly increased the infiltration proportion of CD11b⁺ cells (Fig. 2B), macrophages (CD45⁺CD11b⁺F4/80⁺) (Fig. 2C), and neutrophils (CD45⁺CD11b⁺Ly6G⁺) (Fig. 2D) in tumor tissues. Consistent observations were obtained in 4T1 breast cancer and CT26 colon cancer subcutaneous tumor mouse models (Supplementary Fig. 2). This indicates that intratumoral bacterial colonization can significantly activate the innate immune response within the TME, inducing marked infiltration of innate immune cells including macrophages and neutrophils. Furthermore, this immune recruitment effect exhibits universal applicability across multiple tumor models, including pancreatic, breast, and colorectal cancers. Notably, macrophages and neutrophils, as pioneer responders of the innate immune system, eliminate pathogenic microorganisms through pattern recognition receptor (PRR)-mediated phagocytosis. Although previous studies have emphasized that activation of the innate immune system plays a crucial role in bacterial tumor therapy, it should be noted that these immune cells (e.g. macrophages and neutrophils) may also directly clear bacteria through phagocytosis, thereby weakening the direct anti-tumor effects of the bacteria^37,38^. To investigate the potential inhibitory effects of neutrophils and macrophages on bacterial activity, we evaluated the bactericidal capacity of murine peripheral blood-derived neutrophils and RAW264.7 macrophages in vitro, using other tumor cell lines as control groups. Results showed that following 24 h of co-culture with VNP20009, neutrophils and RAW264.7 macrophages achieved phagocytic clearance rates of 54.14% and 37.17% respectively (Fig. 2E), whereas other cells did not affect bacterial viability. The above experimental results revealed the key role of CD11b⁺ innate immune cell subset (including macrophages and neutrophils) in antimicrobial defense, indicating that this cell subset may constitute the core obstacle limiting the efficacy of bacterial tumor therapy. We hypothesize that simultaneously targeting and regulating macrophages and neutrophils could be a promising strategy to enhance bacterial anti-tumor therapy. Based on this, this study employed the CD11b-targeting agonist GB1275 to regulate the functional state of CD11b⁺ cells for optimizing bacterial therapy. Reports indicate that this compound can enhance the binding capacity of CD11b integrin to the extracellular matrix, effectively inhibiting the migration of CD11b⁺ cells to the lesion site in inflammatory animal models and tumor models^31–34^. First, flow-cytometry analysis revealed that CD11b is specifically highly expressed on the surface of macrophages and neutrophils, with barely detectable expression in tumor cells and normal tissue cells (Fig. 2F), thereby avoiding potential off-target effects of GB1275 on non-target cells. In vitro toxicity evaluation demonstrated that GB1275 exerts remarkably low toxicity against macrophages, neutrophils, various tumor cell lines (4T1, CT26, and Panc02 cells), and non-tumor cells (3T3 fibroblasts) even at high concentrations (Fig. 2G, H and Supplementary Fig. 3). These findings provide strong support for the in vivo safety profile of GB1275.

To investigate whether GB1275 can reduce CD11b⁺ cell infiltration into tumors in vivo, GB1275 (4 mg/kg) was administered daily by intraperitoneal injection in the Panc02 subcutaneous tumor model (Fig. 2I). Quantitative analysis by flow cytometry showed that the proportion of CD11b⁺ cells in the tumor tissue decreased from 35.16% in the control group to 19.92% in the GB1275-treated group, a 43.34% reduction (Fig. 2J), indicating that GB1275 effectively reduces CD11b⁺ cell infiltration into tumors. Although tumor weight exhibited a downward trend (Supplementary Fig. 4A), dynamic monitoring of tumor volume demonstrated no statistically significant anti-tumor efficacy with GB1275 monotherapy (Fig. 2K). Moreover, during long-term administration, mice in the experimental group exhibited no significant body weight fluctuations (Supplementary Fig. 4B) and displayed no observable toxic side effects such as diarrhea or decreased activity, confirming the in vivo safety of the GB1275 dosing regimen. Notably, GB1275 demonstrated consistent inhibitory effects on CD11b⁺ cell infiltration across multiple subcutaneous tumor models. In the 4T1 breast cancer model, CD11b⁺ cell infiltration rates decreased from 34.9% in controls to 21.24% after GB1275 treatment (Supplementary Fig. 5A). Similarly, in the CT26 colon cancer model, infiltration rate was reduced from 23.42% in control tumors to 14.38% in those treated with GB1275 (Supplementary Fig. 5B). Throughout the entire treatment period, mice in the experimental groups showed no significant body weight fluctuations, further confirming the in vivo safety of GB1275. However, in three tumor models (4T1 breast cancer, CT26 colon cancer, and Panc02 pancreatic cancer), although GB1275, by targeting and regulating the CD11b integrin-mediated cell migration pathway, can broadly inhibit the recruitment of CD11b⁺ cells in multiple solid tumors, its monotherapy may be insufficient to directly inhibit tumor growth. Based on these experimental results, we speculate that GB1275 may serve as a promising candidate for combination therapy by specifically inhibiting the abnormal infiltration of bacterial colonization-induced CD11b⁺ innate immune cells into the tumor site, thereby alleviating the local antimicrobial immune barrier and indirectly enhancing the anti-tumor efficacy of bacterial therapy.

### GB1275 effectively inhibits the abnormal infiltration of CD11b⁺ innate immune cells induced by bacterial intratumoral colonization

The activity of bacteria is the foundation for their anti-tumor effects. We first evaluated the toxic effects of GB1275 on bacteria through the CCK-8 assay, plate counting method, and growth curve monitoring. Results from the CCK-8 assay showed that after 24 h of co-incubation with GB1275 at 50 μM, the bacterial survival rate still remained at 90.8 ± 12.86% (Supplementary Fig. 6A). Even at a high concentration of 50 μM, bacterial growth on LB agar plates was nearly unaffected (Supplementary Fig. 6B). Additionally, bacterial growth curves exhibited no significant differences when incubated with varying concentrations of GB1275 **(**Supplementary Fig. 6C). These results indicate that GB1275 did not affect bacterial activity, thereby ensuring the effectiveness and stability of the bacteria in subsequent experiments.

**Fig. 3:**
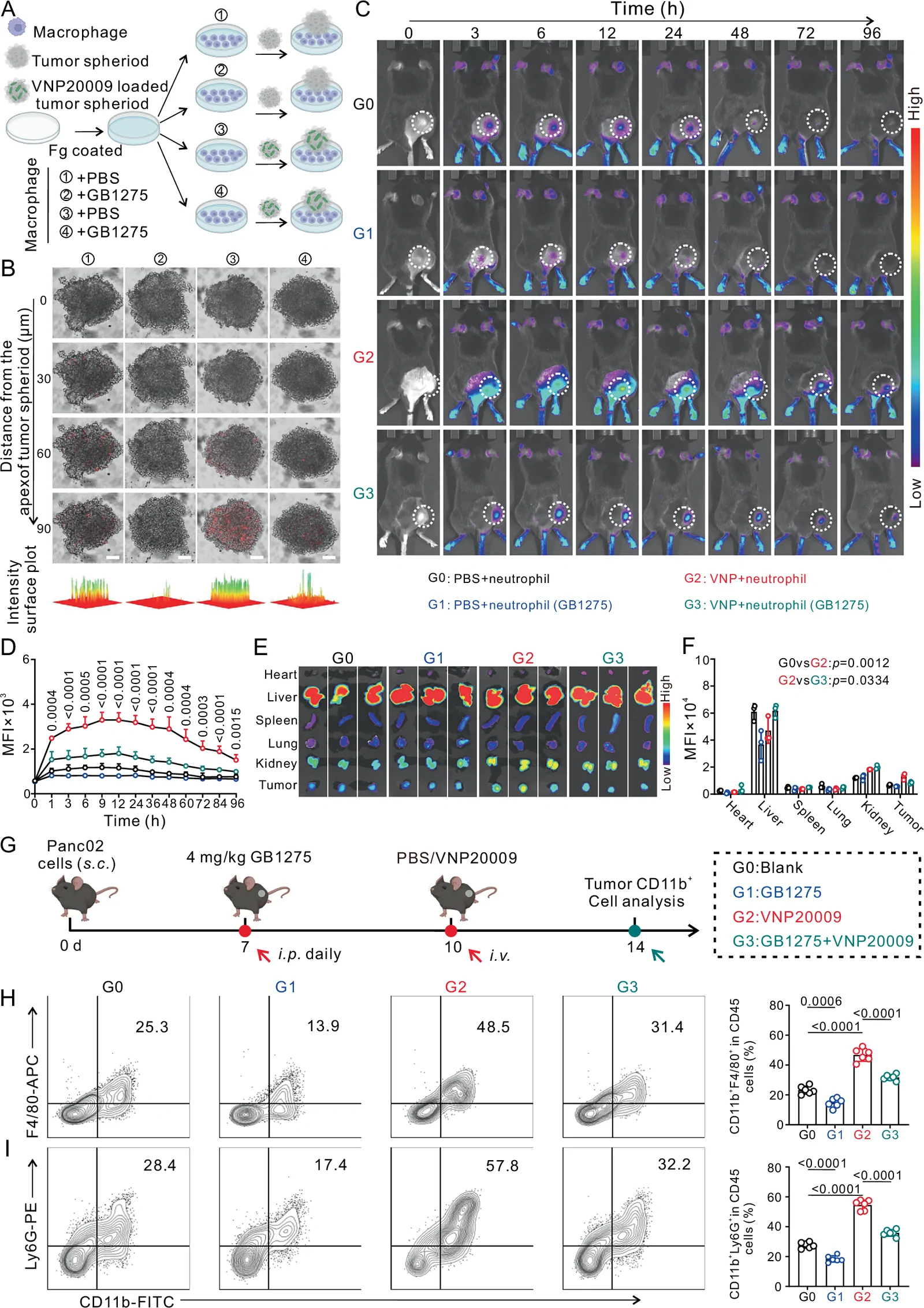
GB1275 effectively inhibits the abnormal infiltration of CD11b⁺ innate immune cells induced by bacterial intratumoral colonization. **A** Schematic illustration of co-culture model establishing macrophage-tumor spheroids/bacterial-infiltrated tumor spheroids. Fg (fibrinogen) was used to enhance macrophage adhesion. **B** Representative confocal fluorescence imaging of tumor spheroids/bacterial-infiltrated tumor spheroids infiltration by neutrophils after different treatments. The red fluorescence represents Cy5.5-labeled neutrophils. The bottom panel is a 3D surface plot of Cy5.5-labeled neutrophils. Scale bar: 20 μm. **C** Representative whole-body fluorescence imaging dynamically monitored the tumor infiltration of neutrophils after different experimental treatments. The dotted circle represents the tumor sites. **D** Fluorescence intensity over time curves at tumor sites after different experimental treatments, and the *P* values obtained in the chart represent the fluorescence statistical analysis of the tumor between G2 and G3, *n* = 5 independent samples. **E**, **F** Fluorescence imaging of major organs and corresponding quantitative fluorescence statistics were performed at 48 h after different experimental treatments. vs is the abbreviation for versus, used for comparison between two groups. The *P* values obtained in the chart represent the fluorescence statistical analysis of the tumor, *n* = 3 independent samples. **G** Schematic illustration of the experimental protocols for GB1275 to inhibit the abnormal infiltration of autologous CD11b⁺ innate immune cells induced by bacterial intratumoral colonization. *s.c*., subcutaneous injection. *i.p*. daily, daily intraperitoneal injection. *i.v*., intravenous injection. **H, I** Flow cytometry and corresponding statistics of macrophages and neutrophils in the tumor tissue after different treatments, *n* = 6 independent samples. The data are expressed as mean ± s.d. All replicates are biological, and the *P* values in (**D**) were determined using two-tailed Student’s *t* test. the *P* values in (**F**, **H**, **I**) were determined using one-way ANOVA followed by Tukey’s multiple comparisons tests. Source data are provided as a Source Data file. **A**, **G** These were created in BioRender. Zhang, B. (2026) https://BioRender.com/th7zath.

Based on the aforementioned research, we systematically evaluated the regulatory effects of GB1275 on bacteria-induced abnormal infiltration of CD11b⁺ innate immune cells (including macrophages and neutrophils) in tumor tissues. By constructing tumor spheroid models, we validated in vitro the capacity of GB1275 to inhibit bacterial intratumoral colonization-induced abnormal infiltration of macrophages and neutrophils. To this end, we established an in vitro bacteria-infiltrated tumor spheroid model to simulate bacterial intratumoral colonization, and evaluated its penetration ability by using Cy5.5-labeled VNP20009^39^. Confocal microscopy images revealed that after 3 h of co-incubation with tumor spheroids, bacteria had deeply infiltrated into the core region of the tumor spheroids (Supplementary Fig. 7), confirming the successful establishment of the bacterial-infiltrated tumor spheroid model. To simulate the in vivo microenvironment, we coated the bottom of cell culture dishes with fibrinogen to enhance macrophage adhesion capacity. A macrophages-tumor spheroid/bacterial-infiltrated tumor spheroid co-culture model was established using Cy5.5-labeled macrophages to evaluate the effects of different treatment conditions on macrophage infiltration into tumor spheroids (Fig. 3A). Fluorescence intensity analysis was performed through 3D reconstruction of the confocal fluorescence images, and subsequent spatial visualization was employed to quantify the depth of macrophage penetration into the tumor spheroids. Results showed that GB1275 reduced macrophage infiltration into tumor spheroids, whereas bacterial colonization in tumor spheroids significantly stimulated macrophage infiltration toward the tumor spheroids. Notably, after treatment with GB1275, a marked reduction in macrophage infiltration into bacterial-infiltrated tumor spheroids was observed (Supplementary Fig. 8), demonstrating that GB1275 effectively suppresses the abnormal macrophage infiltration induced by bacterial colonization in tumor spheroids. Subsequently, we replaced macrophages with neutrophils and employed the same experimental method to assess the effects of different treatment conditions on neutrophil infiltration into tumor spheroids. The results showed that GB1275 equally effectively suppressed the abnormal infiltration of neutrophils induced by bacterial colonization in tumor spheroids (Fig. 3B).

To further validate the effect of GB1275, an adoptive transfer strategy was employed: Cy5.5-labeled macrophages and neutrophils that had undergone different experimental treatments were administered via tail vein injection. We then dynamically monitored the tumor infiltration of these cells via whole-body fluorescence imaging in both Panc02 subcutaneous tumor models and their bacterial-colonized counterparts-the latter of which was established by tail vein injection of VNP20009 into tumor-bearing mice 12 h beforehand. We first performed systematic quantitative analysis of fluorescence intensity at the tumor sites of mice in each group. Based on this, we further conducted statistical analysis of significant differences in tumor fluorescence intensity data from Groups 2 and 3 at different time points, with the corresponding statistical results clearly labeled on the experimental plots. Results demonstrate that GB1275 effectively reduced the infiltration of macrophages and neutrophils into tumors (Supplementary Figs. 9–11 and Fig. 3C, D). In contrast, the bacterial colonization model group exhibited significantly higher fluorescence signal intensity in tumor tissues at all time points compared to other groups, indicating that intratumoral bacterial colonization can induce extensive infiltration of macrophages and neutrophils. Notably, statistical analysis clearly demonstrated that GB1275 pretreatment significantly reduced the fluorescence signal intensity in the tumor in the bacterial colonization model group, which indicated that GB1275 could effectively inhibit the abnormal infiltration of macrophages and neutrophils induced by intratumoral bacterial colonization. Additionally, 48 h after different experimental treatments, mice were euthanized, and tumor tissues and major organs were harvested for ex vivo fluorescence imaging (Supplementary Figs. 12 and 3E, F). The results showed that macrophages and neutrophils predominantly accumulated in the liver. Meanwhile, the fluorescence intensity distribution in tumor tissues was highly consistent with the whole-body fluorescence imaging data, further confirming that GB1275 suppresses the abnormal infiltration of macrophages and neutrophils induced by intratumoral bacterial colonization. Similar to the “seed-soil” hypothesis, circulating tumor cells tend to preferentially colonize the pre-established solid TME^40^. We further verified the targeting specificity of GB1275 by repeating the same experimental protocol using CD11b-negative Panc02 tumor cells. The results showed no significant differences in tumor infiltration of CD11b-negative cells among the groups (Supplementary Fig. 13 and Supplementary Fig. 14), clearly indicating that the effect of GB1275 is CD11b-dependent, with its inhibitory effects primarily targeting CD11b-positive immune cell populations. Subsequently, to further validate the in vivo regulatory effect of GB1275, we employed flow cytometry to quantitatively analyze the infiltration levels of endogenous CD11b⁺ cells, macrophages and neutrophils in tumor tissues of the Panc02 subcutaneous tumor model across different treatment groups (Fig. 3G). The results indicated that intratumoral bacterial colonization markedly increased the proportions of autologous CD11b⁺ cells (Supplementary Fig. 15), macrophages (Fig. 3H), and neutrophils (Fig. 3I) in tumor tissues, rising from 35.47%, 23.26%, and 27.61% to 62.88%, 46.75%, and 54.5%, respectively. However, after administration of GB1275, these proportions decreased to 44.55%, 31.76%, and 35.8%. These results confirm that GB1275 effectively suppressed the abnormal infiltration of autologous CD11b⁺ innate immune cells induced by such bacterial colonization. In vitro and in vivo experimental results show that GB1275 can significantly inhibit the abnormal infiltration of CD11b⁺ innate immune cells induced by bacterial intratumoral colonization. This highlights that the compound holds promise for optimizing the bacterial-mediated anti-tumor immunotherapy strategy by specifically reducing the tumor infiltration of CD11b⁺ innate immune cells, thereby enhancing the therapeutic effect.

### GB1275 enhances the anti-tumor immune effect of bacteria by reducing the intratumoral infiltration of CD11b⁺ innate immune cells

**Fig. 4:**
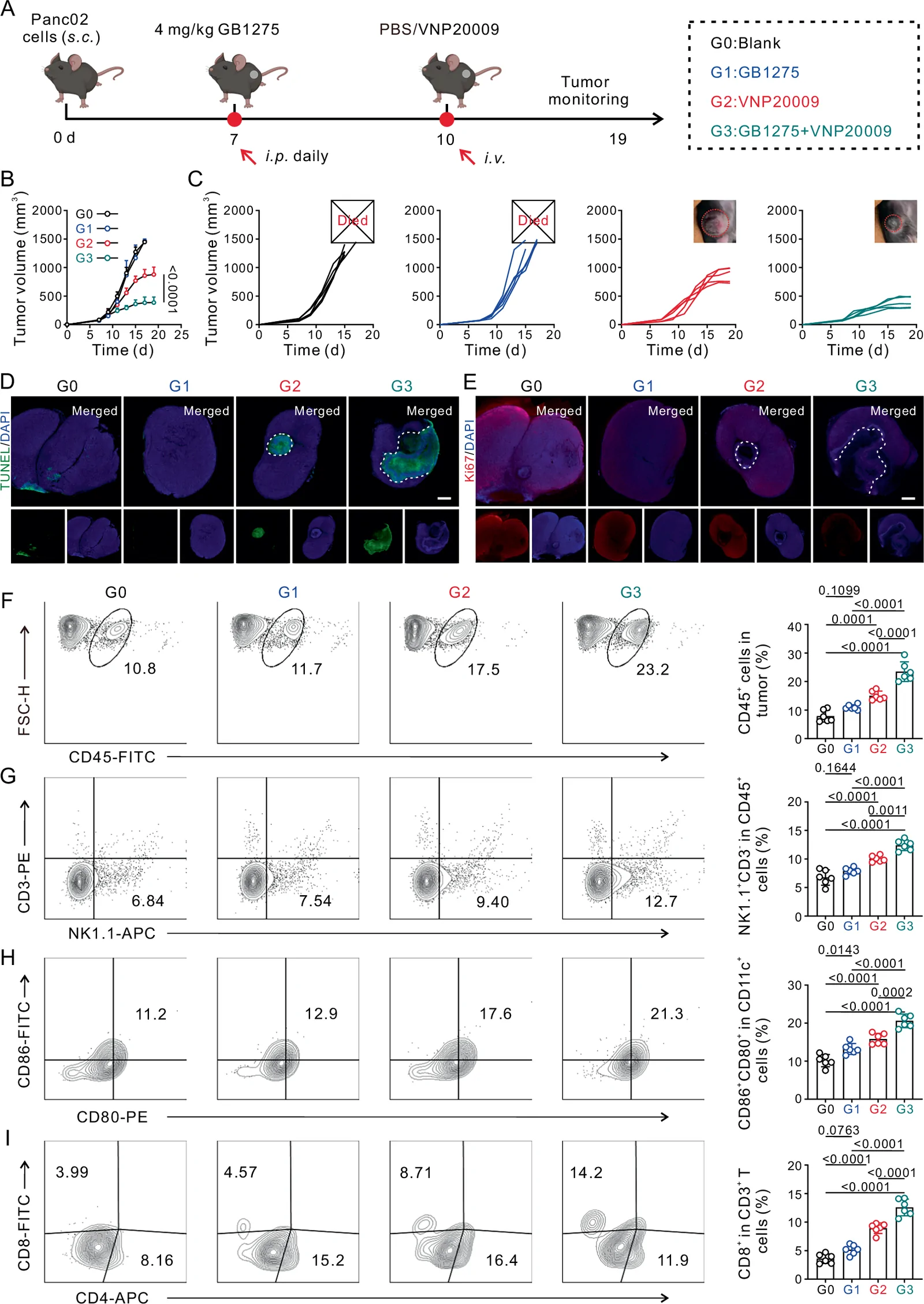
GB1275 enhances the anti-tumor immune effect of bacteria by reducing the intratumoral infiltration of CD11b⁺ innate immune cells. **A** Schematic illustration of the experimental protocols for GB1275-enhanced bacterial anti-tumor therapy. *s.c*., subcutaneous injection. *i.p*. daily, daily intraperitoneal injection. *i.v*., intravenous injection. **B**, **C** Tumor growth curves and individual tumor growth curves after different treatments. In Fig. C, the tumor images in the bar chart are representative images of each group of mice after the end of treatment. The term “died” indicates that mice were humanely euthanized and recorded as deceased when tumor volume exceeded 1500 mm^3^, *n* = 5 independent samples. **D**, **E** Representative images of TUNEL and Ki67 immunofluorescence staining of the largest cross section of Panc02 tumor tissue obtained at day 14 after different treatments. Scale bar: 1 mm. **F**–**I** Flow cytometry analysis and the corresponding statistics of CD45⁺ cells, NK cells, DC cells, and T cells infiltration in Panc02 tumor tissues after different treatments, *n* = 6 independent samples. The data are expressed as mean ± s.d. All replicates are biological, and the *P* values in B were determined using two-way ANOVA and the *P* values in (**F**–**H**) were determined using one-way ANOVA followed by Tukey’s multiple comparisons tests. Source data are provided as a Source Data file. **A** It was created in BioRender. Zhang, B. (2026) https://BioRender.com/th7zath.

We next systematically evaluated the anti-tumor efficacy of GB1275 in combination with bacterial therapy in the Panc02 subcutaneous tumor model (Fig. 4A). Results showed that the body weight of mice in all experimental groups remained stable throughout the treatment period, demonstrating the favorable biosafety profile of the combination therapy (Supplementary Fig. 16). Neither the control group nor the GB1275 monotherapy group exhibited significant tumor inhibition, while the bacteria monotherapy group exerted modest tumor growth inhibition. Notably, the combination treatment group exerted a marked anti-tumor effect, characterized by significantly slower tumor growth (Fig. 4B, C) and obvious tumor volume reduction (Supplementary Fig. 17). Moreover, after treatment completion, the major organs of mice from each group were collected for H&E staining, and all tissues showed no significant histological damage, further indicating the excellent biosafety of this therapeutic strategy (Supplementary Fig. 18). To further elucidate the mechanism underlying GB1275’s enhancement of bacterial therapy efficacy, we dynamically monitored changes in intratumoral bacterial counts using the plate counting method (Supplementary Fig. 19A). Results showed that intratumoral bacterial counts of the combination treatment group were significantly higher than those in the single-bacteria treatment group (Supplementary Fig. 19B, C). We further assessed the effect of GB1275 on the survival rate of intratumoral bacteria. Results demonstrated that GB1275 could significantly enhance the survival rate of bacteria within tumors (Supplementary Fig. 19D). Furthermore, we used mCherry-expressing VNP20009 instead of wild-type VNP20009 in the assay. Coupled with immunofluorescence imaging of tumor tissue sections, we further confirmed directly and intuitively that GB1275 treatment significantly increased bacterial colonization abundance in tumor tissues and extended their intratumoral survival time compared with the control group (Supplementary Fig. 20). This finding validates our initial hypothesis that GB1275 enhances the anti-tumor efficacy of bacterial therapy by reducing intratumoral infiltration of neutrophils and macrophages, thereby disrupting the intratumoral antibacterial immune barrier and indirectly increasing local bacterial counts within tumors. Chemokine receptor 2 (CCR2) inhibitors can inhibit myeloid cell migration via distinct mechanisms. To this end, we introduced the CCR2 inhibitor RS 504393, while additionally setting clodronate liposomes (macrophage depletor) and anti-Ly6G antibody (neutrophil depletor) as controls. By blocking myeloid cell infiltration into TME through different approaches, this study aimed to comparatively explore the regulatory effects of various reagents on the abundance and activity of intratumoral oncolytic bacteria (Supplementary Fig. 21A). We employed the LB plate counting method to quantitatively analyze intratumoral bacterial counts of each group, and calculated the relative survival rate of bacteria in each group with the PBS-treated group as the reference (Supplementary Fig. 21B–F). Results showed that RS 504393 did not increase VNP20009 counts, whereas clodronate liposomes and anti-Ly6G antibody both increased them to varying degrees. This indicates that intratumoral colonization of VNP20009 does not induce CCR2-dependent myeloid cell infiltration into tumors. Furthermore, we employed mCherry-expressing VNP20009 for fluorescence imaging of tumor tissue sections, thereby further verifying directly and intuitively the enhancing effect of clodronate liposomes and the anti-Ly6G antibody on intratumoral bacterial abundance and activity (Supplementary Fig. 22). These results confirm that clodronate liposomes and anti-Ly6G antibody can modulate abnormal myeloid cell infiltration and function via distinct mechanisms, and all exert potential tumor growth inhibition when combined with VNP20009. Notably, it is critical to clarify that GB1275 acts as a myeloid cell migration inhibitor, whereas clodronate liposomes and the anti-Ly6G antibody are specific myeloid cell depletors, with fundamentally distinct modes of action. On this basis, we further investigated the effects of combining GB1275, clodronate liposomes or anti-Ly6G antibody with VNP20009 on the survival time of tumor-bearing mice (Supplementary Fig. 23). The results showed that although the combination regimen based on clodronate liposomes and the anti-Ly6G antibody effectively prolonged the survival of mice, their systemic and sustained depletion of neutrophils and macrophages during the treatment and observation periods led to the death of some mice when the tumor volume was still below 1500 mm³. Based on the experimental context, these deaths were most likely attributable to bacterial infection. In contrast, the GB1275 combination regimen exhibited superior safety and a longer survival period. This difference in efficacy may be attributed to the low toxicity of GB1275 and its broad-spectrum targeted inhibition of aberrant myeloid cell migration, thereby achieving a more comprehensive regulatory range. Additionally, GB1275 itself can exert anti-tumor effects by polarizing TAMs toward a pro-inflammatory phenotype, thereby exerting a more thorough effect in protecting intratumoral bacteria and ameliorating the tumor immune microenvironment. To further elucidate the mechanisms underlying GB1275’s marked therapeutic efficacy, we performed Hematoxylin and Eosin (H&E) staining, Ki67 proliferation marker staining, and Terminal deoxynucleotidyl transferase dUTP Nick-End Labeling (TUNEL) assay on the maximal cross-sections of tumor tissues for histological and immunofluorescence analysis. H&E staining results showed that tumor tissues in the control group and GB1275 group remained nearly intact. Focal tumor necrosis was observed only in the VNP20009 monotherapy group, whereas the GB1275 + VNP20009 combination treatment group exhibited extensive tumor necrosis (Supplementary Fig. 24). Furthermore, TUNEL immunofluorescence staining (Fig. 4D) and analyses of Ki67 immunofluorescence (Fig. 4E) revealed opposing expression patterns of these markers. Compared with the control group and GB1275 monotherapy group, both the bacteria monotherapy group and combination treatment group showed decreased Ki67 expression (indicating reduced proliferation) and increased TUNEL-positive cells (indicating enhanced apoptosis), which were consistent with the findings from H&E staining. Notably, compared with the bacteria monotherapy group, the combination treatment group exhibited more pronounced and extensive apoptosis, with significantly weaker and more restricted proliferative activity (as indicated by Ki67 expression). These findings confirm that the bacterial monotherapy can induce focal tumor necrosis. However, its anti-tumor efficacy remains constrained by antimicrobial defense mechanisms mediated through innate immune responses within the TME. In comparison, GB1275 effectively reduces infiltration of CD11b⁺ innate immune cells into tumor tissues, suppresses antimicrobial defenses, and significantly elevates bacterial load in tumor tissue, thereby expanding necrotic regions and improving therapeutic outcomes. These findings highlight the critical role of GB1275 in enhancing the direct anti-tumor activity of oncolytic bacteria.

The anti-tumor effect of oncolytic bacteria stems not only from their ability to directly induce tumor cell apoptosis, but is also closely linked to the subsequently activated immune response. Via pathogen-associated molecular patterns (PAMPs, such as flagellin and lipopolysaccharide), bacteria can activate the TLRs signaling pathway, reshape the tumor immunosuppressive microenvironment, induce the infiltration of various anti-tumor immune cells, and thereby elicit robust adaptive immune responses^16–18^. Building on this foundation, we further explored the impact of GB1275 on bacteria-mediated immune responses (Supplementary Fig. 25A). Flow cytometric analysis revealed that bacterial monotherapy significantly increased the proportions of tumor-infiltrating CD45⁺ immune cells (Fig. 4F), NK cells (CD45⁺CD3⁻NK1.1⁺) (Fig. 4G), DCs (CD45⁺CD11c⁺CD86⁺CD80⁺) (Fig. 4H), CD8⁺ T cells (CD45⁺CD3⁺CD8⁺) (Fig. 4I), whereas CD4⁺ T cells (CD45⁺CD3⁺CD4⁺) exhibited no significant changes (Fig. 4I and Supplementary Fig. 25B). Notably, the combination of GB1275 with bacterial therapy further enhanced the infiltration levels of these anti-tumor immune cell populations. These findings confirm that GB1275 does not exert a negative impact on the infiltration of the aforementioned anti-tumor immune cells, which is attributed to the general CD11b-negative or low-expression profiles in these immune cell populations. The phenotypic state of macrophages plays a critical regulatory role in the TME. Although GB1275 effectively suppresses the abnormal infiltration of macrophages induced by intratumoral bacterial colonization, experimental data reveal that a considerable number of macrophages remain within tumor tissues. Based on this, we further analyzed the phenotypic characteristics of macrophages in the TME. Results showed that in the combination treatment group, the proportion of M2 macrophages (CD45⁺CD11b⁺F4/80⁺CD206⁺) converting to the anti-tumor M1 phenotype (CD45⁺CD11b⁺F4/80⁺CD86⁺) was significantly higher than that in the bacteria monotherapy groups (Supplementary Fig. 25C–G). This indicates that the increased intratumoral bacterial load not only helps directly kill tumor cells but also helps reverse the immunosuppressive TME by promoting M1 macrophage polarization. Additionally, we compared the macrophage polarization potential of GB1275, VNP20009, and their different combinations in vitro, with LPS, a classical inducer of macrophage polarization, as a positive control. The results showed that LPS and VNP20009 exhibited significantly stronger macrophage polarization potential than that of GB1275 (Supplementary Fig. 26). Although LPS and VNP20009 showed no significant difference in their ability to induce M1 macrophage polarization, this observation confirms that LPS and bacterial PAMPs exert comparable effects on M1 macrophage polarization. Taken together, these findings further validate the remarkable synergistic efficacy of the GB1275 + VNP20009 combination in reversing the immunosuppressive TME by modulating macrophage polarization and enhancing anti-tumor immune responses. In conclusion, the mechanism by which GB1275 enhances the anti-tumor effects of oncolytic bacteria relies on two pathways: First, by inhibiting the infiltration of CD11b⁺ innate immune cells into tumor tissues, it disrupts the intratumoral antimicrobial defense mechanisms in the TME, significantly elevates the number of intratumoral bacteria, and promotes bacterial proliferation and diffusion in the microenvironment, thereby expanding the scope of direct tumoricidal activity; Second, the elevated intratumoral bacterial load further strengthens the infiltration of anti-tumor immune cells and significantly enhances the intensity of immune responses. Thus, the observed tumor growth inhibition is attributed to the synergistic interplay between the enhanced direct tumoricidal activity of oncolytic bacteria and the robust activation of anti-tumor immune responses. This confirms that modulating the bacteria-immune interactions by specifically targeting CD11b⁺ innate immune cells represents a novel and effective strategy to boost oncolytic bacterial immunotherapy for pancreatic cancer.

### The combination of GB1275 with Lip@V demonstrates enhanced tumor suppression efficacy

**Fig. 5:**
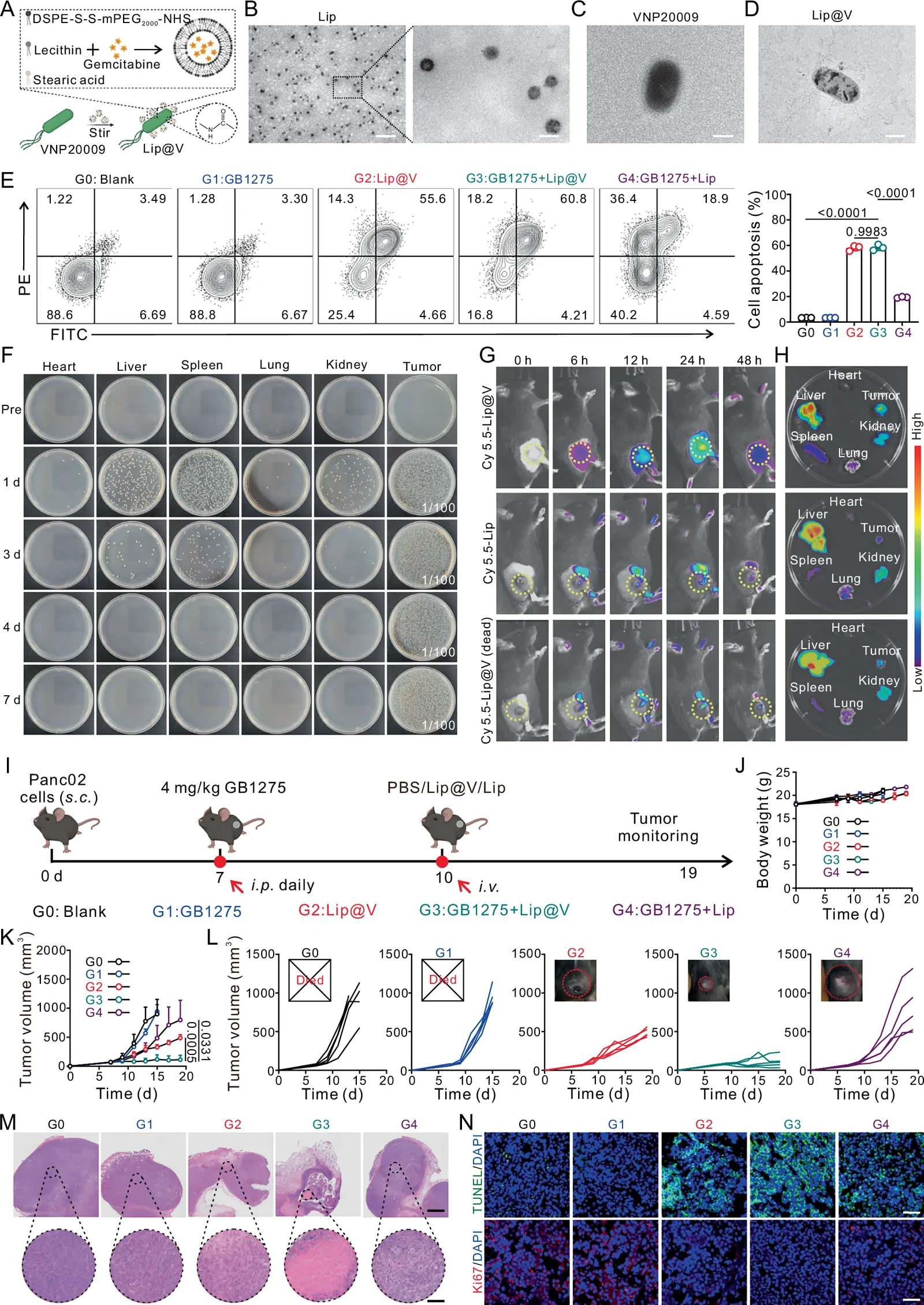
The combination of GB1275 with Lip@V demonstrates enhanced tumor suppression efficacy. **A** Schematic illustration of the experimental protocols for the construction of Lip@V. **B**–**D** TEM images of Lip, VNP20009, and Lip@V. Scale bar, 500 nm and 200 nm (enlarged image). **E** Flow cytometry analysis and the corresponding apoptosis rate of Panc02 cells after different treatments, *n* = 3 independent samples. **F** Representative photographs of bacterial colonization in major organs and tumors obtained from Panc02 tumor-bearing mice at different time points after intravenous administration of Lip@V. *n* = 3 independent samples. **G** Representative whole-body fluorescence imaging at various time points after different treatments. Cy5.5-Lip@V (dead) denotes the Lip-modified dead VNP20009. **H** Representative fluorescence imaging of major organs and tumors at 24 h after different treatments, *n* = 3 independent samples. **I** Schematic illustration of the experimental protocols for the combination of GB1275 with Lip@V for anti-tumor therapy. *s.c*., subcutaneous injection. *i.p*. daily, daily intraperitoneal injection. *i.v*., intravenous injection. **J** Changes in mouse body weights after different treatments, *n* = 5 independent samples. **K**, **L** Tumor growth curves and individual tumor growth curves after different treatments. In Fig. L, the tumor images are representative images of each group of mice after the end of treatment. The term “Died” indicates that mice were humanely euthanized and recorded as deceased when tumor volume exceeded 1500 mm^3^, *n* = 5 independent samples. **M**, **N** Representative images of H&E staining, TUNEL, and Ki67 immunofluorescence staining of tumor tissues after different treatments on day 14. Scale bar: 1 mm and 125 μm (enlarged image) in M. Scale bar: 200 μm in N. The data are expressed as mean ± s.d. All replicates are biological, and the *P* values in (**E**) were determined using one-way ANOVA and the *P* values in K were determined using two-way ANOVA followed by Tukey’s multiple comparisons test. Source data are provided as a Source Data file. **A**, **I** These were created in BioRender. Zhang, B. (2026) https://BioRender.com/th7zath.

Facultative anaerobic bacteria can target and colonize the hypoxic microenvironment of solid tumors; their autonomous chemotactic motility enables penetration through dense stromal barriers, overcoming the bottleneck of low delivery efficiency of conventional drugs in pancreatic cancer treatment^14^. To further boost the anti-tumor efficacy, we encapsulated gemcitabine, a clinical first-line chemotherapeutic drug for pancreatic cancer, within GSH-responsive nanoliposomes, and conjugated these nanoliposomes to the surfaces of oncolytic bacteria via amide linkages, thus constructing drug-loaded bacterial constructs (Lip@V) (Fig. 5A). First, gemcitabine-loaded nanoliposomes were fabricated via the thin-film hydration method. Transmission electron microscopy (TEM) characterization revealed that the liposomes exhibited a uniform spherical morphology with an average particle size of approximately 200 nm (Fig. 5B and Supplementary Fig. 27A). To ensure the viability of oncolytic bacteria, we systematically evaluated the effects of different liposome concentrations on bacterial activity prior to constructing Lip@V. As validated by CCK-8 assay, bacterial growth curve analysis, and plate counting method, nanoliposomes at concentrations ≤ 3 mg/mL exhibited no significant inhibition on bacterial activity (Supplementary Fig. 27B–D). Based on these results, 3 mg/mL nanoliposomes were incubated with oncolytic bacteria overnight to achieve stable immobilization of nanoliposomes on the bacterial surface via amide bond-mediated covalent conjugation. Further TEM characterization revealed that, in contrast to the smooth surface of unmodified oncolytic bacteria, densely anchored nanoliposome particles were observable on the surface of Lip@V, confirming the successful conjugation of nanoliposomes to the bacterial surface (Fig. 5C, D). To visually verify the conjugation between liposomes and bacteria, we prepared FITC-labeled Lip@V (FITC-Lip@V) by incorporating DSPE-mPEG2000-FITC into the liposome. Confocal laser scanning microscopy (CLSM) imaging showed that FITC-Lip@V exhibited significant green fluorescence signals compared to Lip@V, further confirming the efficient conjugation of liposomes to bacterial surfaces (Supplementary Fig. 27E). Attenuated *Salmonella typhimurium* VNP20009 possesses the capability to actively invade cells. To assess the invasive properties of Lip@V toward tumor cells, FITC-Lip@V was co-cultured with Panc02 tumor cells in vitro, and the invasion efficiency was analyzed via flow cytometry. Results demonstrated a time-dependent enhancement in the tumor cell invasion efficiency of FITC-Lip@V (Supplementary Fig. 27F). Upon invading Panc02 tumor cells, Lip@V releases gemcitabine in response to high intracellular GSH levels, thereby inducing cytotoxicity. To quantify this cytotoxic effect, CCK-8 assays were performed to quantify the cytotoxic effects of Lip@V and VNP20009 monotherapy on tumor cells at varying concentrations. Experimental results demonstrated that compared with VNP20009 monotherapy, Lip@V exhibited significantly enhanced tumor cell-killing capacity, providing a theoretical foundation for subsequent in vivo anti-tumor experiments (Supplementary Fig. 27G).

Prior to proceeding with the in vivo anti-tumor evaluation of Lip@V, we first assessed the ability of different combinations to induce tumor cell apoptosis in vitro using Annexin V/PI double staining assay. Flow cytometry results showed that both the Lip@V monotherapy group and the GB1275 + Lip@V combination therapy group exhibited significantly enhanced tumor-killing capacity compared with other control groups, and this enhanced apoptotic effect was primarily attributed to Lip@V (Fig. 5E). Subsequently, we evaluated the in vivo biological safety of Lip@V by dynamically monitoring blood biochemical indicators and blood cell parameters in mice on days 1 and 7 post-administration. Results showed that although some liver function indicators (ALT/AST) and blood cell parameters (PLT/Lymph) exhibited transient fluctuations during the initial administration period, all abnormal indicators returned to physiological levels on day 7. Meanwhile, other hematological parameters remained stable throughout the observation period, confirming the favorable in vivo biological safety of Lip@V (Supplementary Fig. 28). VNP20009 demonstrates active targeting and selective colonization in solid tumor tissues following intravenous injection. Unlike the rapid clearance of these attenuated bacteria in normal tissues, the compromised immune surveillance function within the hypoxic-necrotic area enables their transient proliferation in this region. To quantitatively evaluate the tissue distribution dynamics of Lip@V, tumor-bearing mice were sacrificed at various time points after tail vein administration of Lip@V. Major organs (liver, spleen, kidney, and lung) and tumor tissues were harvested from the sacrificed mice, homogenized under sterile conditions, and the bacterial burden of Lip@V in these tissues was quantified via the plate counting method. Results indicated that while residual Lip@V colonies were detected in normal organs at early time points post-administration, nearly complete clearance of Lip@V from normal organs was achieved by day 3 post-administration (Fig. 5F and Supplementary Fig. 29). This demonstrates the host immune system’s efficient clearance capacity in normal tissues, while an increased bacterial burden was detected in tumor tissues. Subsequently, the tumor-targeting capacity of Lip@V was evaluated using in vivo whole-body fluorescence imaging. Cy5.5-labeled Lip@V (Cy5.5-Lip@V) were administered via the tail vein to Panc02 subcutaneous tumor-bearing mice. Heat-inactivated Lip@V (Cy5.5-Lip@V (dead), serving as a non-targeting control due to loss of bacterial activity and free Cy5.5-labeled nanoliposomes (Cy5.5-Lip) were set as control groups. Fluorescence signals at the tumor sites were dynamically monitored using a whole-body fluorescence imaging system at predetermined time points. Results demonstrated that the Cy5.5-Lip@V group showed time-dependent increase in tumor fluorescence intensity, which peaked at 24 h post-administration and remained significantly higher than that of the Cy5.5-Lip@V (dead) and Cy5.5-Lip groups throughout the observation period (Fig. 5G). This indicates that living bacteria can achieve active tumor targeting through their chemotactic motility combined with the hypoxic selectivity of the TME. Additionally, mice were sacrificed 24 h post-treatment, and their major organs and tumor tissues were harvested for ex vivo organ fluorescence imaging. Results showed that the fluorescence signal distribution at the tumor site exhibited a high degree of consistency with the in vivo whole-body imaging, and the tumor fluorescence intensity in the Cy5.5-Lip@V group was significantly higher than that of the Cy5.5-Lip@V (dead) and Cy5.5-Lip groups (Fig. 5H and Supplementary Fig. 30). This further confirms the excellent tumor-targeting capability of Lip@V, providing critical support for precise drug delivery and localized tumor therapy. Although liposomal modification had no adverse effect on the viability or tumor-targeting capacity of VNP20009, the anti-tumor efficacy of such bacteria is highly dependent on their ability to activate the anti-tumor immune system. Thus, compared with unmodified parental VNP20009, liposome-bacterium conjugates are likely to elicit distinct anti-tumor immune responses. In this study, using PBS as a negative control, we focused on the innate immune system, which is characterized by rapid and robust responses, to investigate whether modification with empty liposomes would affect the intratumoral immune activation ability of bacteria (eLip@V). Experimental results showed that liposome modification exerted no significant effect on the intratumoral innate immune responses induced by bacteria (Supplementary Fig. 31). It is possible that the GSH-responsive liposomes used in our study can undergo cleavage to release gemcitabine in response to high levels of GSH overexpressed in the TME. These liposomes undergo site-specific lysis at the tumor site and therefore do not affect bacterial activity or interfere with the physiological functions and immune effects mediated by bacteria. These results lay a solid experimental foundation for verifying the feasibility of in vivo anti-tumor immunotherapy based on the combination of GB1275 + Lip@V.

Subsequently, synergistic anti-tumor experiments between GB1275 and Lip@V were designed and performed in vivo (Fig. 5I). Results showed that body weights of mice in all treatment groups remained stable without significant fluctuations throughout the therapeutic cycle, further confirming the favorable in vivo safety profile of the proposed regimen (Fig. 5J). Compared with control groups, GB1275 monotherapy exerted negligible tumor growth inhibition. In contrast, both the Lip@V group and the GB1275 + Lip demonstrated moderate anti-tumor efficacy. Notably, the GB1275 + Lip@V combination treatment group exhibited the strongest anti-tumor effect, significantly suppressing tumor growth compared with all other groups (Fig. 5K, L and Supplementary Fig. 32). This potent efficacy is attributed to GB1275, which significantly enhances the intratumoral drug delivery efficiency of Lip@V and reinforces its bacterial colonization capacity in the TME by inhibiting the infiltration of CD11b⁺ innate immune cells. The active tumor-targeting capability and deep stromal penetration ability of Lip@V further enhance the localized enrichment of chemotherapeutic agents, thereby achieving tumor suppression effects significantly superior to those of all other control and monotherapy groups. To further validate this potent efficacy at the histological level, tumor tissues were subjected to H&E staining (Fig. 5M), TUNEL assay, and Ki67 immunofluorescence staining (Fig. 5N). Results demonstrated that the GB1275 + Lip@V combination therapy group exhibited the most extensive necrotic regions, the strongest TUNEL signals (indicating high apoptotic rate), and the weakest Ki67 fluorescence intensity (reflecting suppressed cell proliferation) in tumor sections, further confirming the enhanced tumor-destructive capacity of the combinatorial regimen. Additionally, histopathological examination of major organs (heart, liver, spleen, lung, and kidney) from all treatment groups revealed no obvious pathological damage or abnormal lesions (Supplementary Fig. 33). This finding, consistent with the stable body weight observations during the therapeutic cycle, reconfirms the excellent in vivo biosafety profile of the GB1275 + Lip@V combinatorial therapeutic strategy. Pancreatic cancer is clinically characterized by poor prognosis and short overall survival, and novel immunotherapeutic strategies have emerged as a promising direction to improve the survival outcomes of patients. To further validate the therapeutic value of our proposed regimen, we evaluated the effects of different treatment combinations on the survival of Panc02 tumor-bearing mice. Experimental results demonstrated that the GB1275 + Lip@V combination therapy group significantly prolonged the median survival time (MST) of mice compared with the PBS control group, GB1275 monotherapy group, Lip@V monotherapy group, and GB1275 + Lip group (Supplementary Fig. 34). However, in clinical settings, pancreatic ductal adenocarcinoma is often diagnosed and treated only when the disease progresses to the advanced stage. Due to significant differences in TME and stromal components between early and advanced tumors, the therapeutic effects may exhibit substantial heterogeneity. Given the high malignancy and rapid progression of Panc02 tumor, this study adopted a tumor volume of approximately 1500 mm^3^ as the definition criterion for the advanced model, and initiated the predetermined treatment regimen at this time point. Specifically, the predetermined therapeutic regimen was initiated when the tumors reached this volume (Supplementary Fig. 35). Notably, experimental results demonstrated that, compared with other control and monotherapy groups (PBS, GB1275 monotherapy, Lip@V monotherapy, and GB1275 + Lip group), the GB1275 + Lip@V combination therapy exhibited significantly superior tumor growth inhibitory efficacy against advanced Panc02 pancreatic cancer. This finding suggests that the combination therapy regimen also possesses promising application potential and clinical translational value in the treatment of advanced pancreatic cancer.

### The combination of GB1275 with Lip@V profoundly reshapes the pancreatic cancer immunosuppressive microenvironment and activates adaptive immunity

**Fig. 6:**
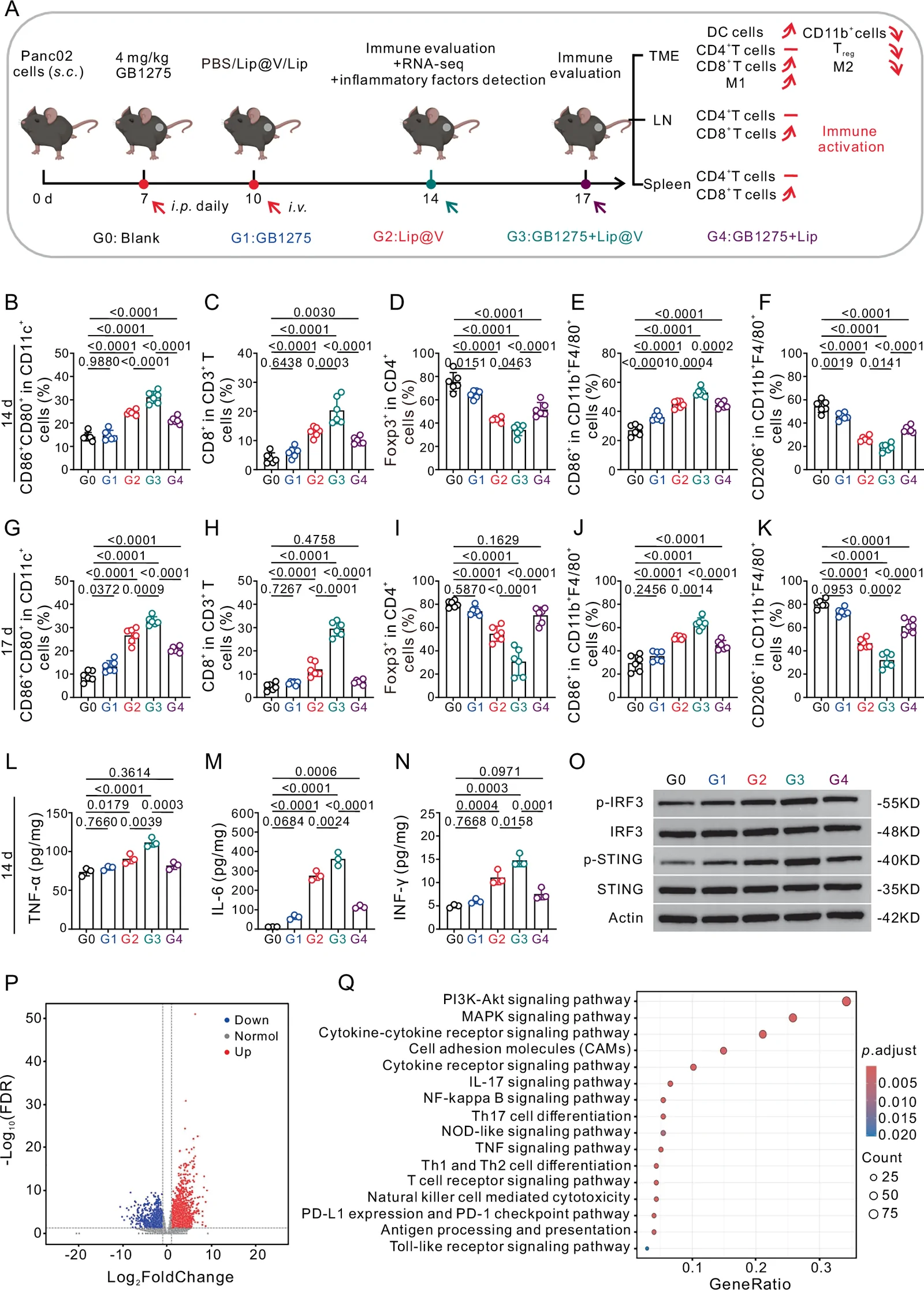
The combination of GB1275 with Lip@V profoundly reshapes the pancreatic cancer immunosuppressive microenvironment and activates adaptive immunity. **A** Schematic illustration of the experimental protocols for in vivo immune responses triggered by GB1275 + Lip@V. *s.c*., subcutaneous injection. *i.p*. daily, daily intraperitoneal injection. *i.v*., intravenous injection. LN represents lymph nodes. Corresponding flow-cytometry analysis statistics of (**B**, **G**) DCs, (**C**, **H**) CD8⁺ T cells, and (**D**, **I**) Tregs infiltration in tumor tissues after different treatments on day 14 and 17, *n* = 6 independent samples. Corresponding flow analysis statistics of the proportion of (**E**, **J**) M1 and (**F**, **K**) M2 macrophages in tumor tissue after different treatments on day 14 and 17, *n* = 6 independent samples. **L**–**N** The levels of TNF-α, IL-6, and IFN-γ in tumor tissues after different treatments, *n* = 3 independent samples. The data are expressed as mean ± s.d. All replicates are biological, and the *P* values in B-N were determined using one-way ANOVA followed by Tukey’s multiple comparisons test. **O** Representative immunoblot of p-STING, STING, p-IRF3, IRF3, and actin (loading control) in tumor tissues after different treatments. On day 14 after treatment, untreated (blank) and GB1275 + Lip@V-treated tumors were taken for transcriptome sequencing, *n* = 5 independent samples. **P** Volcano plot for identifying differentially expressed genes before and after GB1275 + Lip@V treatment. The overall distribution of regulated genes is represented by a volcano plot, in which the abscissa and ordinate represent the fold change of gene expression in different samples and statistical significance (*P* value) of the difference in gene expression, respectively. **Q** The enrichment scatter plot to show KEGG enrichment analysis of a selection of immune-related KEGG terms. Gene ratio is the ratio of the number of differential genes to the total number of genes in the pathway. Source data are provided as a Source Data file. **A** It was created in BioRender. Zhang, B. (2026) https://BioRender.com/th7zath.

Pancreatic cancer is characterized by a highly immunosuppressive TME, whose immune evasion mechanisms significantly limit the efficacy of conventional therapies^41^. Therefore, this study focuses on the TME-reshaping effect of the GB1275 + Lip@V combination therapy in pancreatic cancer and aims to elucidate the key mechanisms by which this therapeutic regimen activates adaptive immunity (Fig. 6A). In the Panc02 subcutaneous tumor-bearing mouse model, tumor tissues, inguinal lymph nodes, and spleens were harvested on days 14 and 17 post-Lip@V administration to analyze the phenotypes of various immune cell subsets via flow cytometry. Results demonstrated that GB1275 significantly reduced the aberrant infiltration of CD11b⁺ innate immune cells and their subsets induced by Lip@V intratumoral colonization within the TME (Supplementary Fig. 36).

The activation of antigen-presenting cells (APCs) is an essential step in initiating adaptive immune responses. Experimental results demonstrated that the proportion of mature DCs in tumor tissues was significantly higher in the GB1275 + Lip@V combination treatment group compared with other control and monotherapy groups. This finding indicates that the combined regimen effectively activates DCs to initiate robust anti-tumor adaptive immune responses, laying a foundation for subsequent T cell-mediated immunity (Fig. 6B, G, and Supplementary Fig. 37). Specifically, Lip@V, with its excellent tumor-targeting capability and deep tissue penetration ability, can precisely deliver higher concentrations of encapsulated gemcitabine to the tumor site, and gemcitabine is released in response to the highly expressed GSH in the TME, enabling efficient tumor cell killing. Meanwhile, the addition of GB1275 enhances the tumor cell death effect induced by the bacteria. As a result of the synergistic cytotoxicity of gemcitabine and VNP20009, a large amount of TAAs was released from apoptotic/necrotic tumor cells. These TAAs are subsequently captured and processed by DCs, further promoting DC maturation and initiating downstream adaptive immune activation, consistent with the elevated mature DC proportion observed in the combination group.

T cell infiltration within tumor tissues is critical for initiating effective adaptive anti-tumor immune responses. In this experiment, the GB1275 + Lip@V combination therapy group maintained the highest infiltration levels of CD8⁺ T cells in tumor tissues from days 14 to 17 after different treatments, aligning with the activation cycle of adaptive immunity (Fig. 6C, H and Supplementary Fig. 38). In stark contrast, monotherapy groups induced limited T cell recruitment. Notably, intratumoral CD4⁺ T cell infiltration levels did not exhibit significant differences across all treatment groups and control groups, which may be attributed to the combinatorial regimen primarily targeting the activation and recruitment of CD8⁺ T lymphocytes, the core effector cells of anti-tumor adaptive immunity. Furthermore, immunofluorescence staining for CD8⁺ T cells in tumor tissues across treatment groups revealed the highest fluorescence intensity in the combination group, confirming augmented CD8⁺ T cell infiltration (Supplementary Fig. 39). Additionally, we observed that in mice treated with the GB1275 + Lip@V combination therapy, the proportion of CD8⁺ T cells in inguinal lymph nodes and spleens, key secondary lymphoid organs for T cell activation, proliferation, and homing, was significantly higher than that in all control and monotherapy groups (Supplementary Fig. 40). This finding further confirms that the combination regimen can induce a robust systemic adaptive immune response, as the elevated CD8⁺ T cell proportion in lymphoid organs reflects enhanced T cell priming and expansion. Collectively, these results underscore the great potential of the GB1275 + Lip@V combination in boosting anti-tumor adaptive immune responses, both systemically and within the TME.

Tregs (CD45⁺CD4⁺Foxp3⁺), TAM (particularly the M2 phenotype), and myeloid-derived suppressor cells collectively constitute critical components of the immunosuppressive TME in pancreatic cancer-all of which potently suppress the anti-tumor activity of effector CD8⁺ T lymphocytes and impede the initiation of effective adaptive immune responses. Flow cytometric analysis revealed that the combination therapy significantly reduced the proportion of Tregs compared with other groups (Fig. 6D, I and Supplementary Fig. 41). Although GB1275 effectively diminished the aberrant infiltration of macrophages triggered by Lip@V intratumoral colonization, a considerable fraction of macrophages still persisted within tumor tissues. Given the marked functional heterogeneity of TAMs, with M2-polarized TAMs being the dominant immunosuppressive subtype in the TME that impairs anti-tumor immunity, a targeted characterization of intratumoral macrophage subtypes and their functional states was therefore performed. The results demonstrated that the GB1275 + Lip@V combination therapy significantly increased the intratumoral proportion of M1 macrophages, proinflammatory and anti-tumorigenic macrophages that enhance effector immune cell function, compared with all control and monotherapy groups (Fig. 6E, J, and Supplementary Fig. 42). Concomitantly, the proportion of M2 macrophages-the dominant immunosuppressive subtype that impairs anti-tumor immunity-was significantly reduced in the combination group (Fig. 6F, K, and Supplementary Fig. 42). This M1/M2 phenotypic shift indicates that the combination regimen not only diminishes TAM infiltration but also reshapes the functional state of residual TAMs to create a pro-anti-tumor TME. Notably, the GB1275 + Lip@V combination regimen also exerted potent M1 polarization-inducing effects on RAW264.7 murine macrophage cells in vitro, substantially elevating the proportion of M1-polarized cells (Supplementary Fig. 43). Collectively, these in vivo and in vitro data demonstrate that the combination of GB1275 and Lip@V effectively reverses the immunosuppressive TME through reshaping macrophage phenotypic polarization (upregulating M1 anti-tumor subtype and downregulating M2 immunosuppressive subtype), reducing the infiltration of Tregs, and enhancing anti-tumor effector immune responses (e.g., DC maturation and CD8⁺ T cell infiltration).

To further elucidate the molecular mechanisms underlying TME remodeling and macrophage phenotypic polarization, we performed quantitative PCR (qPCR) to analyze the expression levels of representative M1/M2-associated genes in sorted intratumoral TAMs. The results demonstrated that compared with all control and monotherapy groups, the combination therapy group exhibited significantly upregulated expression of M1-associated markers including iNOS, TNF-α, and IL-6. Conversely, the expression of M2-associated immunosuppressive markers, such as SOCS2 and CD206, was markedly downregulated (Supplementary Fig. 44). These findings reveal the molecular basis for macrophage reprogramming from an M2 to an M1 phenotype. Furthermore, the levels of representative inflammatory cytokines in tumor tissues were quantified using ELISA kits. The data showed that the GB1275 + Lip@V combination therapy significantly elevated the concentrations of TNF-α, IL-6, and IFN-γ in the tumor tissue (Fig. 6L-N). Notably, the IFN-γ secretion level was substantially higher than that in control groups. IFN-γ plays a critical role in the activation, proliferation, and functional execution of CD8⁺ T cells. Western blot analysis further revealed that the increased IFN-γ levels were closely associated with the activation of the cGAS-STING signaling pathway (Fig. 6O). Specifically, both GB1275 and VNP20009 can activate the cGAS-STING pathway and promote type I interferon expression, but they act through distinct molecular mechanisms. GB1275, as a CD11b agonist, regulates macrophage phenotypic polarization (switching from M2 to M1) and modulates innate immune responses by engaging the endogenous immune receptor CD11b, thereby indirectly amplifying STING pathway activation. In contrast, VNP20009 directly triggers host cGAS-STING recognition via exogenous microbial nucleic acids, initiating a canonical cytosolic DNA-sensing innate immune response^42^. This dual-activation mechanism not only amplifies innate immune responses but also provides sustained stimulatory signals for T cell-mediated adaptive immunity.

To further investigate the specific mechanisms by which GB1275 + Lip@V activates immune responses, we performed transcriptome sequencing analysis (RNA-seq) on tumor tissues from eight mice (five in the blank group and five in the GB1275 + Lip@V treatment group) on day 4 post-treatment, and generated heat maps using the expression data of selected genes from each group. The results showed 2533 upregulated genes and 1711 downregulated genes (Fig. 6P and Supplementary Fig. 45A). Differentially expressed genes were analyzed using Kyoto Encyclopedia of Genes and Genomes (KEGG) pathway and Gene Ontology (GO) enrichment analyses. As shown in Fig. 6, we selected 35 inflammation/immune-related pathway genes displayed in the scatter plot (Fig. 6Q and Supplementary Fig. 45B), which were categorized into four major classes: immune signaling pathways, inflammatory response, innate immune response, and adaptive immune response.

Compared with the blank group, key immune pathways in the GB1275 + Lip@V group were upregulated, such as pattern recognition receptors including TLRs and NLRs pathways in innate immune responses (Fig. 6Q and Supplementary Fig. 46A), chemokines/cytokines for immune activation (Fig. 6Q and Supplementary Fig. 46B, C), NK cell-mediated cytotoxicity (Fig. 6Q), DC differentiation and antigen processing/presentation (Fig. 6Q, Supplementary Fig. 45B and 46D), and T cell functions for adaptive immunity (Fig. 6Q, Supplementary Figs. 45B and 46E). These messenger RNA (mRNA) data provide further evidence for the activation of innate and adaptive immune responses following combination therapy.

Flow cytometry analysis combined with transcriptome sequencing data has confirmed that GB1275 + Lip@V combination therapy can efficiently and durably activate anti-tumor adaptive immune responses through multiple targets, achieving deep remodeling of the pancreatic cancer TME. Specifically, the combined therapy of GB1275 and Lip@V activates the adaptive immune system mainly through the following two aspects: (1) The combined application of GB1275 and bacteria activates multiple innate immune pathways including TLRs, NLRs, and the cGAS-STING pathway, significantly enhancing the body’s innate immune response. The core mechanism of this strategy lies in maintaining the dominant proportion of M1 macrophages within the tumor, thereby effectively transforming the immunosuppressive TME into an environment conducive to anti-tumor immunity. This transformation promotes the sustained release of various immune-related cytokines, which is crucial for the recruitment, proliferation, and activation of T cells, as well as the enhancement of their cytotoxic activity, supporting the effective activation of adaptive immunity. (2) The gemcitabine-loaded bacteria effectively trigger anti-tumor immune responses through the dual synergistic effect between GSH-responsive drug release and bacteria-mediated oncolytic effects. The gemcitabine-mediated chemotherapy combined with the bacterial lysis mechanism not only enhances the direct killing effect on tumor cells but also induces immunogenic cell death (ICD), thereby promoting the release of TAAs. These antigens are subsequently captured and presented by DCs, activating the clonal expansion of specific tumor-targeted T lymphocytes. This process activates and reinforces the adaptive immune response, promoting the body’s effective recognition and elimination of tumor cells.

The above results confirmed that the GB1275 + Lip@V combination therapy regimen exerts remarkable anti-tumor immunotherapeutic efficacy in the Panc02 pancreatic cancer model. However, it should be noted that different pancreatic ductal adenocarcinomas exhibit inherent heterogeneity, a characteristic that may compromise the universality of the therapeutic regimen. Pancreatic ductal adenocarcinoma is frequently accompanied by epithelial-mesenchymal transition (EMT). Tumors of distinct EMT subtypes possess markedly divergent invasive and metastatic potential, which may greatly compromise the broad-spectrum anti-tumor efficacy of therapeutic regimens. To investigate the impact of tumor heterogeneity on the therapeutic efficacy of the GB1275 + Lip@V combination therapy, we further introduced the KPC-derived mouse PDAC model for parallel validation. We first performed WB analysis on KPC and Panc02 cells as well as their derived subcutaneous tumor tissues to detect the expression of epithelial marker E-cadherin and mesenchymal markers N-cadherin and Vimentin. Cellular WB results demonstrated that KPC cells barely expressed E-cadherin, while N-cadherin and Vimentin were weakly but significantly expressed relative to E-cadherin, indicating an inherent mesenchymal-biased phenotype of KPC cells in vitro (Supplementary Fig. 47A). Panc02 cells exhibited high levels of N-cadherin and Vimentin, which were also markedly higher than E-cadherin, revealing that Panc02 cells likewise presented a mesenchymal tendency under in vitro culture. Since epithelial-mesenchymal transition (EMT) is a dynamic event during pancreatic cancer progression in vivo, we further examined these EMT markers in corresponding tumor tissues. E-cadherin was significantly upregulated in KPC tumors and its abundance exceeded that of N-cadherin, demonstrating prominent mesenchymal-epithelial transition (MET) occurring during in vivo tumor formation of KPC cells (Supplementary Fig. 47B). No obvious difference in total Vimentin expression was observed between the two tumor groups, which could be explained by widespread Vimentin production by fibroblasts within the tumor microenvironment, masking intrinsic differences derived from tumor cells. In Panc02 tumors, the expression gap between E-cadherin and N-cadherin was negligible. Compared with cultured Panc02 cells, E-cadherin expression remained unaltered in tumor tissues, whereas N-cadherin was sharply downregulated, suggesting gradual loss of mesenchymal traits and an intermediate epithelial-mesenchymal hybrid status for Panc02 tumors. We further validated these expression patterns via immunohistochemical staining, which was consistent with WB results: KPC tumors displayed markedly stronger E-cadherin staining than Panc02 tumors, while Vimentin immunoreactivity showed no apparent distinction between groups (Supplementary Fig. 47C). Taken together, results from cellular and tissue WB and immunohistochemistry indicated that both cell lines exhibited mesenchymal phenotypes in vitro, yet underwent divergent phenotypic reprogramming after in vivo implantation. KPC-derived tumors underwent robust MET and converted to an epithelial subtype, whereas Panc02-derived tumors resided in an epithelial-mesenchymal hybrid state. These two models with distinct EMT phenotypes enable subsequent evaluation of the broad-spectrum anti-tumor efficacy of our combination regimen against pancreatic cancers with disparate EMT status.

We systematically evaluated the tumor-inhibitory effects of different treatment combinations on KPC tumors and conducted long-term dynamic follow-up (Supplementary Fig. 48A)^43^. During the treatment period, no significant fluctuations were detected in the body weight of the mice, indicating that this therapeutic regimen has favorable safety in the KPC tumor model (Supplementary Fig. 48B). The results demonstrated that the GB1275 + Lip@V combination therapy still exerted robust tumor-inhibitory activity in this model, with complete tumor regression observed in one tumor-bearing mouse (Supplementary Fig. 48C, D, Supplementary Fig. 49 and Supplementary Fig. 50). Furthermore, analyses of intratumoral immune responses showed that the intratumoral infiltration patterns of CD11b⁺ cells in the KPC model were consistent with the results in the Panc02 model (Supplementary Fig. 51A). Additionally, the GB1275 + Lip@V group significantly increased the proportions of mature DCs (Supplementary Fig. 51B), CD8⁺ T cells (Supplementary Fig. 51C), and M1 macrophages (Supplementary Fig. 51E, G) in KPC tumor tissues, while markedly reducing the proportions of Tregs (Supplementary Fig. 51D) and M2 macrophages (Supplementary Fig. 51F, G). This suggests that the combination therapy can effectively reverse the immunosuppressive TME of KPC tumors and exert potent anti-tumor immune activation activity. Collectively, these results confirm that the GB1275 + Lip@V combination therapy regimen holds great potential for application in a variety of pancreatic cancer models, and its therapeutic efficacy is not easily interfered with by the inherent heterogeneity of pancreatic cancer itself.

### The combination of GB1275 with Lip@V significantly inhibits the progression of orthotopic pancreatic cancer

**Fig. 7:**
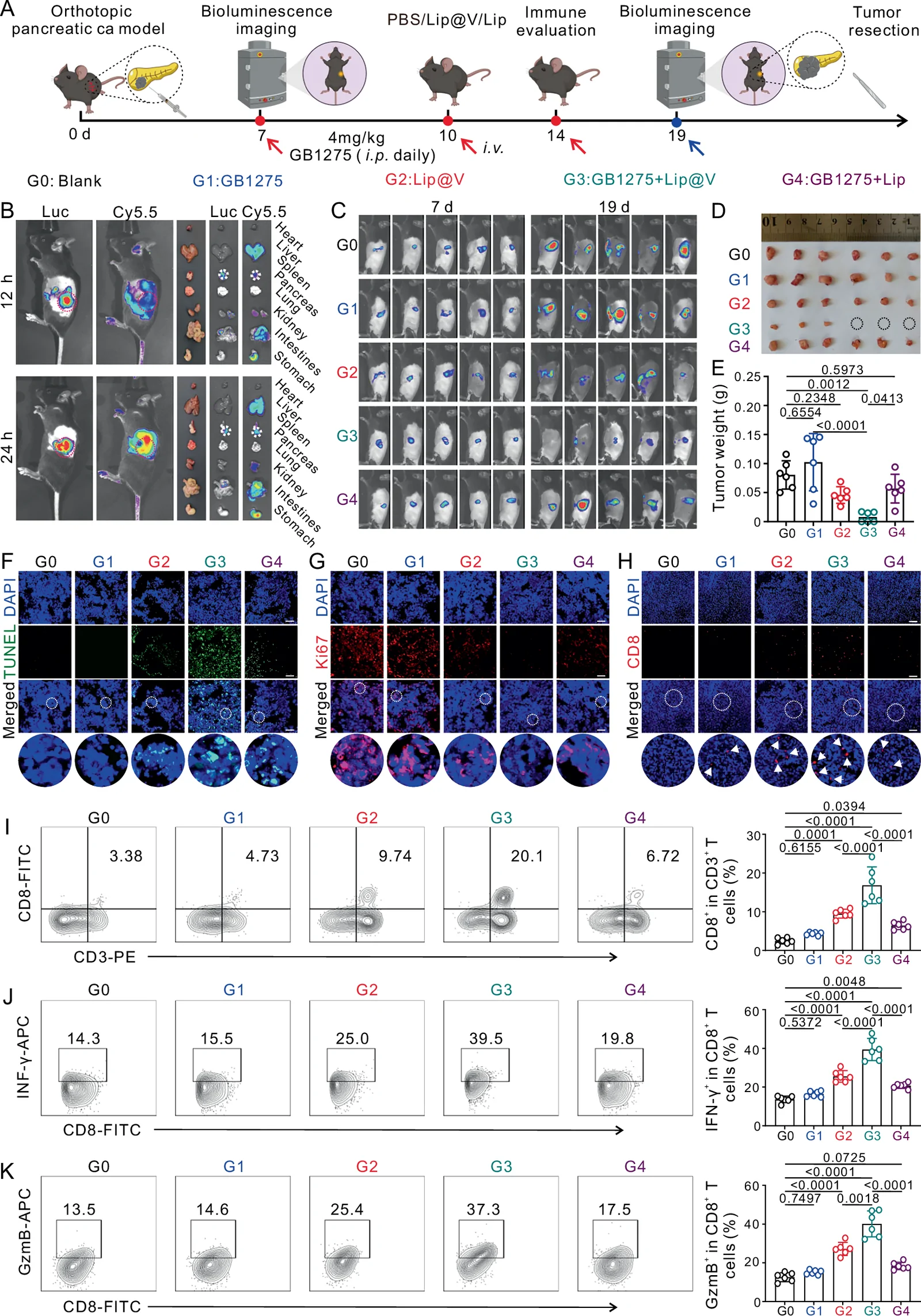
The combination of GB1275 with Lip@V significantly inhibits the progression of orthotopic pancreatic cancer. **A** Schematic illustration of the experimental protocols for the combination therapy of GB1275 and Lip@V in the treatment of in situ pancreatic cancer. *s.c*., subcutaneous injection. *i.p*. daily, daily intraperitoneal injection. *i.v*., intravenous injection. **B** Representative fluorescence imaging combined with bioluminescence imaging co-localizes the enrichment of Lip@V in orthotopic pancreatic tumor at different time points post-administration. The figure includes whole-body fluorescence imaging and whole-body bioluminescence imaging of the in situ pancreatic cancer model, white light images of major organs and tissues, as well as fluorescence imaging and bioluminescence imaging. The fluorescent signals originate from Cy5.5-labeled Lip@V, while the bioluminescence imaging results from light emission produced by Panc02-luc cells catalyzing the substrate. **C** In vivo bioluminescence imaging of in situ pancreatic cancer after different treatments, *n* = 5 independent samples. **D**, **E** Photographs and tumor weight analysis of the harvested tumors after different treatment groups, *n* = 6 independent samples. **F**, **G** Representative images of TUNEL and Ki67 immunofluorescence staining of tumor tissues after different treatments. Scale bar: 200 μm. **H** Representative images of CD8⁺ T cells immunofluorescence staining of tumor tissue after different treatments. To clearly localize the target cells, white triangular arrows were used in the figure to more explicitly highlight the T cells. Scale bar: 200 μm. **I**–**K** Flow cytometry analysis and corresponding statistics of CD8⁺ T cells, IFN-γ-secreting CD8⁺ T cells, and GzmB-secreting CD8⁺ T cells infiltration in tumor tissues after different treatments on day 14, *n* = 6 independent samples. The data are expressed as mean ± s.d. All replicates are biological, and the *P* values in (**E**, **I**–**K**) were determined using one-way ANOVA followed by Tukey’s multiple comparisons test. Source data are provided as a Source Data file. **A** It was created in BioRender. Zhang, B. (2026) https://BioRender.com/th7zath.

To precisely mimic the clinicopathological characteristics of pancreatic cancer, we established an orthotopic pancreatic cancer model based on female C57BL/6 mice (Fig. 7A). Panc02 cells stably expressing luciferase (Luc) were injected into the subcapsular region of the pancreas via surgical operation, and dynamic monitoring by an in vivo imaging system (IVIS) showed that tumor cells successfully formed orthotopic lesions (Supplementary Fig. 52). A dual-modality imaging technique (Cy5.5 fluorescence imaging combined with Luc bioluminescence detection) was used to validate the orthotopic tumor targeting of Lip@V: at 12 h and 24 h after injection, Cy5.5 signals showed high spatial co-localization with the tumor bioluminescent regions, confirming that Lip@V can actively target and colonize orthotopic pancreatic cancer lesions, providing a precise delivery basis for subsequent therapy (Fig. 7B).

In vivo imaging system monitoring of tumor progression demonstrated that the GB1275 + Lip@V combination treatment group exhibited significant therapeutic effects in the orthotopic model. The bioluminescence intensity on day 19 was markedly reduced compared with that in the control groups (Fig. 7C). Additionally, after mice were sacrificed, tumors were resected, photographed, and weighed at the end of treatment. The combination therapy group showed the strongest tumor suppression capacity, with three out of six mice achieving complete tumor regression (Fig. 7D, E and Supplementary Fig. 53), further confirming the robust efficacy of the combination therapy. To further evaluate the effects of combining GB1275 with Lip@V on tumor tissues, we conducted immunofluorescence staining analyses using TUNEL and Ki67 immunofluorescence staining assays. The results showed that the combination treatment group exhibited the highest TUNEL-positive signals (Fig. 7F) and lowest Ki67 expression levels (Fig. 7G), indicating that this therapeutic strategy exerts stronger destructive effects on orthotopic tumor tissues. Furthermore, flow cytometric analysis demonstrated that GB1275 retained the capacity to reduce bacteria-induced aberrant infiltration of CD11b⁺ cells in the orthotopic pancreatic cancer model, showing high consistency with previous experimental findings (Supplementary Fig. 54). Previous experiments demonstrated that the GB1275 + Lip@V combination therapy significantly activates adaptive immunity through dual mechanisms, manifesting as enhanced T cell recruitment, proliferation, activation, and cytotoxicity. Therefore, we specifically evaluated CD8⁺ T cell immune responses via flow cytometry in the Panc02 orthotopic pancreatic cancer model. Experimental results indicated that the GB1275 + Lip@V combination therapy group induced a significantly higher proportion of tumor-infiltrating CD8⁺ T cells compared with the other groups (Fig. 7I). Moreover, the proportions of effector phenotype markers granzyme B (CD45⁺CD3⁺CD8⁺GzmB⁺) (Fig. 7J) and IFN-γ (CD45⁺CD3⁺CD8⁺IFN-γ⁺) (Fig. 7K) were significantly upregulated, indicating a potent cytotoxic T lymphocyte (CTL) response. Additionally, immunofluorescence staining for CD8⁺ T cells in tumor tissues showed the strongest fluorescence signal in the combination therapy group, further confirming enhanced CD8⁺ T cell infiltration (Fig. 7H). Collectively, GB1275 combined with Lip@V therapy not only more effectively inhibited the growth of orthotopic pancreatic cancer but also significantly activated the adaptive immune response, highlighting its great potential as a promising therapeutic strategy for pancreatic cancer.

## Discussion

We have confirmed the critical role of innate immune-mediated antibacterial defense mechanisms in limiting bacterial anti-tumor efficacy, and further uncovered the “double-edged sword” role of CD11b⁺ innate immune cells (macrophages and neutrophils) in bacteria-mediated tumor therapy**—**they act as key hubs for initiating anti-tumor immune cascades, yet their overactivation forms an intrinsic barrier by clearing intratumoral bacteria and blunting therapeutic efficacy. Based on this, we propose a strategy to target innate immune responses via the CD11b-targeted agonist GB1275, which simultaneously mitigates excessive innate immune barriers while enhancing adaptive immune activation. This approach aims to achieve synergistic enhancement of “bacteria-immune interactions,” offering a novel avenue for pancreatic cancer immunotherapy.

Although the immunosuppressive microenvironment formed by local hypoxic necrosis in tumors provides conditions for the colonization and proliferation of a small number of bacteria, the anti-tumor efficacy of bacteria is still significantly limited. This study confirmed that intratumoral bacterial colonization induces aberrant infiltration of macrophages and neutrophils, and the inhibitory effect of these cells on bacterial activity was verified through in vitro experiments. From a theoretical perspective, synchronous targeted regulation of the antibacterial defense mechanisms mediated by neutrophils and macrophages may represent an ideal strategy to enhance the efficacy of bacteria-mediated anti-tumor immunotherapy. In view of the fact that neutrophils and macrophages co-express CD11b, this study selected GB1275, a non-toxic small-molecule agonist that can specifically target and inhibit CD11b⁺ cells, to enhance the anti-tumor immune efficacy of VNP20009. Through systematic verification by in vitro and in vivo experiments, GB1275 was shown to effectively inhibit the aberrant infiltration of CD11b⁺ immune cells and their subsets (neutrophils and macrophages) induced by intratumoral bacterial colonization, and we further elucidated the core mechanism underlying its enhanced anti-tumor effect: increased intratumoral bacterial load in tumor tissues not only exerts a direct oncolytic effect, but also achieves a synergistic anti-tumor effect by activating the host immune system. Based on this, we subsequently constructed the gemcitabine-loaded oncolytic bacterium Lip@V. The combined therapy of GB1275 + Lip@V can effectively inhibit the growth of subcutaneous and orthotopic Panc02 pancreatic cancer, reverse the immunosuppressive microenvironment of pancreatic cancer, and exert a significant inhibitory effect on advanced tumors while greatly prolonging the survival time of tumor-bearing mice. More importantly, this combined therapy can be extended to the treatment of KPC pancreatic cancer, and its therapeutic efficacy is not affected by the inherent heterogeneity of pancreatic cancer, reflecting favorable broad therapeutic applicability. This also implies that the combined therapy is promising for direct application in the treatment of other tumor models.

William Coley pioneered anti-tumor immunotherapy using Coley’s toxin prepared from heat-inactivated bacteria, which was later replaced by radiotherapy and chemotherapy due to efficacy and safety concerns. In recent years, advances in synthetic biology and immunology have facilitated the initiation of clinical trials for live bacteria-based anti-tumor therapy, yet most have yielded suboptimal outcomes. To date, only BCG has been successfully applied in the clinical treatment of bladder cancer. Although the phase I clinical trial of VNP20009 did not achieve the expected anti-tumor efficacy, this strain exhibited favorable in vivo safety, indicating its potential as a relatively safe agent for subsequent research. GB1275 is a novel oral allosteric agonist of CD11b with a unique mechanism of action that circumvents the dose limitations of early CD11b-targeting strategies. Numerous studies have confirmed that it can regulate the immune microenvironment, inhibit the infiltration of myeloid cells into tumors, and exert anti-tumor effects. In addition, results from the phase I/II clinical trials of GB1275 have demonstrated its excellent biological safety; it has been combined with pembrolizumab, nab-paclitaxel and gemcitabine, respectively, for the treatment of pancreatic cancer, colorectal cancer and gastric cancer^35^. Although we have only verified the tumor-inhibitory effect of the combination therapy of GB1275 and gemcitabine-loaded VNP20009 in animal models to date, this study provides a highly promising combination therapy regimen for the clinical management of pancreatic cancer. Nevertheless, bacteria-mediated anti-tumor immunotherapy necessitates a delicate balance between efficacy and risks. As live biotherapeutics, bacteria carry the risks of systemic disseminated infection, inflammatory imbalance, virulence reversion mutation of attenuated strains, and infection in immunocompromised patients. Subsequent research should focus on screening low-virulence and genetically stable bacterial strains, optimizing dosing regimens, formulating personalized treatment plans and implementing real-time toxicity monitoring throughout the treatment process, so as to maximize the anti-tumor effects of bacteria while safeguarding patient safety to the greatest extent.

## Methods

### Ethical statement

All animal experiments were approved by the Animal Experimentation Ethics Committee of Huazhong University of Science and Technology (IACUC Number: S904), and all procedures complied with the approved tumor size limits.

### Cell lines and animals

NIH 3T3 cells (Cat# CRL-1658) were provided by Wuhan Service Biotechnology Co., Ltd., while murine colon cancer cells (CT26, Cat# CRL-2638), pancreatic cancer cells (Panc02, Cat# CRL-2553), luciferase-labeled mouse pancreatic cancer cells (Panc02-LUC, derived from Pan02, Cat# CRL-2553), and RAW 264.7 macrophages (ATCC, Cat# TIB-71) were obtained from the American Type Culture Collection (ATCC). Special thanks are due to Professor Gan Lu from the College of Life Sciences and Technology, Huazhong University of Science and Technology (HUST) for the kind donation of the KPC cell line for this study. Attenuated *Salmonella* VNP20009 (Cat# 202165) was purchased from Biosafer Plasmid & Strain Company and cultured in Luria-Bertani broth at 37 °C. All cell lines were maintained in Dulbecco’s Modified Eagle Medium (DMEM) supplemented with 10% (vol/vol) fetal bovine serum, 100 U/mL penicillin, and 0.1 mg/mL streptomycin, under humidified conditions at 37 °C with 5% CO_2_ and 90% humidity, and the medium was refreshed every two days. All cell lines used in this study were confirmed to be mycoplasma-free.

Female BALB/c mice (BALB/cAnNCrl, 8-week-old weighing 18–20 g) and C57BL/6 mice (C57BL/6NCrl, 8-week-old weighing 18–20 g) were acquired from Charles River Laboratories Animal Technology Co., Ltd., Beijing. Only female mice were used in this study, as this is the standard and widely used sex in established pancreatic cancer models to ensure tumor engraftment consistency and minimize inter-animal variability. The animals were housed under controlled environmental conditions (temperature: 20–22 °C, humidity: 30–70%, 12 h light / 12 h dark cycle) with free access to food and water. Starting from the day of grouping and treatment, tumor length (*L*) and width (*W*) were measured with a vernier caliper every two days, and tumor volume was calculated as *V* = *L* × *W*^2^/2. To minimize animal discomfort, tumor burden should not exceed 20% of the animal’s normal body weight during routine experiments according to the “Guidelines for Humane Endpoints in Animal Experiments” (CNCA-RB/T 173-2018). In this experiment, tumor burden was required not to exceed 10% of each animal’s body weight, and the maximum allowable volume for subcutaneous dorsal tumors was 1500 mm^3^. At the end of the mouse experiments, euthanasia was performed in accordance with animal welfare standards via isoflurane anesthesia.

### Materials and reagents

Phosphate-buffered saline (PBS, Cat# 10010023), Luria-Bertani (LB, Cat# 10855001), and Dulbecco’s Modified Eagle Medium (DMEM, Cat# 11965092) were purchased from Thermo Fisher Scientific (Shanghai, China). The RNA isolation kit (Cat# RC101), HiScript III All-in-one RT SuperMix Perfect for qPCR kit (Cat# R333), and Taq Pro Universal SYBR qPCR Master Mix (Cat# Q712) were obtained from Vazyme Biotech Co., Ltd. (Nanjing, China). The Omni-Easy™ One-Step PAGE Gel Rapid Preparation Kit (Cat# PG212) was acquired from Shanghai Epizyme Biomedical Technology Co., Ltd. (Shanghai, China). Fetal bovine serum (FBS, Cat# 11011-8611) was purchased from Zhejiang Tianhang Biotechnology Co., Ltd. (Hangzhou, China). Fibrinogen (Cat# F108831), lecithin (Cat# L108738), stearic acid (Cat# S108292), peptone (Cat# P108238), and yeast extract (Cat# Y108231) were purchased from Shanghai Aladdin Biochemical Technology Co., Ltd. (Shanghai, China). Brefeldin A (Cat# S1536), Phorbol 12-myristate 13-acetate (PMA, Cat# S1819), ionomycin (Cat# S1672), the BCA Protein Assay Kit (Cat# P0012), CCK-8 assay kit (Cat# C0037), SDS-PAGE sample loading buffer (5×, Cat# P0015L), penicillin-streptomycin (Cat# C0222), RNase-free ddH_2_O (Cat# R0021), 4% paraformaldehyde fixative solution (Cat# P0099), and Tween-20 (Cat# ST825) were supplied by Shanghai Beyotime Biotechnology Co., Ltd. (Shanghai, China). DSPE-S-S-mPEG2000-NHS was obtained from Xi’an Ruixi Biotechnology Co., Ltd. Gemcitabine (Cat# HY-17026) was obtained from MedChEmexpress (New Jersey, USA). GB1275/ADH-503 (Cat# S82477) were purchased from Shanghai Yuanye Biotechnology Co., Ltd. (Shanghai, China) and MedChemExpress LLC (New Jersey, USA). Anti-Ly-6G was purchased from Merck Millipore (Darmstadt, Germany). Clodronate liposomes were purchased from Solarbio (Beijing, China). RS 504393 was purchased from Shanghai Yuanye Biotechnology Co., Ltd. (Shanghai, China). Fluorophore-labeled antibodies including FITC anti-mouse CD45 (Clone S18009F, Cat# 103106, 1: 200), FITC anti-mouse CD11b (Clone M1/70, Cat# 1012347, 1: 200), PerCP anti-mouse CD11b (Clone M1/70, Cat# 10124315, 1: 200), APC anti-mouse F4/80 (Clone BM8, Cat# 12314071, 1: 200), PE anti-mouse Ly-6G (Clone 1A8, Cat# 12764777, 1: 200), FITC anti-mouse CD86 (Clone GL-1, Cat# 1050254, 1: 200), PE anti-mouse CD206 (Clone C068C2, Cat# 14177424, 1: 200), PE anti-mouse CD3 (Clone 17A2, Cat# 100247, 1: 200), APC anti-mouse CD4 (Clone RM4-5, Cat# 10059224, 1: 200), PE anti-mouse FOXP3 (Clone MF-14, Cat# 12644660, 1: 100), FITC anti-mouse CD8a (Clone 53-6.7, Cat# 10070153, 1: 200), APC anti-mouse CD11c (Clone N418, Cat# 11731813, 1: 200), PE anti-mouse CD80 (Clone 16-10A1, Cat# 10470043, 1: 200), APC anti-mouse IFN-γ (Clone XMG1.2, Cat# 50580993, 1: 100) and APC anti-mouse Granzyme B (Clone QA16A02, Cat# 372214429, 1: 100) were purchased from BioLegend (California, USA). APC-Cy7 anti-human CD45 antibody (Clone 2D1, Cat# 557833, 1: 200) was obtained from BD Biosciences (California, USA). Cy5 goat anti-rabbit IgG (Polyclonal, Cat# GB27303, 1: 500) and FITC goat anti-rabbit IgG (Polyclonal, Cat# GB22303, 1: 200) were obtained from Servicebio (Wuhan, China).

### Tumor model construction

To establish the murine 4T1 subcutaneous breast cancer model, approximately 1 × 10^6^ 4T1 cells were injected into the lower right dorsal region of female BALB/c mice (8-week-old mice weighing 18–20 g). For the murine CT26 subcutaneous colon cancer model, the same number of CT26 cells was similarly inoculated into the lower right dorsal area of female Balb/c mice (8-week-old mice weighing 18–20 g) using the same method. The murine Panc02 subcutaneous pancreatic cancer model was constructed by injecting approximately 1 × 10^6^ Panc02 cells into the lower right dorsal flank of female C57BL/6 mice (8-week-old mice weighing 18–20 g). When the tumor volume reached approximately 70 mm^3^, subsequent relevant experiments were initiated; for the advanced tumor model, experiments were initiated when the tumor volume reached approximately 150 mm^3^. The KPC tumor model was established by inoculating approximately 1 × 10^6^ KPC cells into the subcutaneous raised area of the subcutaneous site of the right lower back of female C57BL/6 mice.

For the orthotopic pancreatic cancer model, C57BL/6 mice were anesthetized using a mixture of 1–2% isoflurane in oxygen via a nose cone. The fur at the surgical site was clipped, and the mice were positioned laterally on a clean bench under sterile conditions. After locating the pancreas beneath the rib cage by palpation, a small surgical incision was made to carefully exteriorize the pancreas. Approximately 25 μL of Panc02-LUC tumor cells (1 × 10^7^ cells/mL) were injected into the pancreas. The incision was closed with absorbable sutures, and the surgical site was disinfected to ensure asepsis throughout the procedure.

### In vivo tissue flow-cytometry analysis

For the in vivo tissue cell flow cytometric analysis, all procedures involving mouse tissues were performed as follows. Mice were euthanized according to humane endpoints guidelines, and tumors, spleens, or lymph nodes were carefully excised. These tissues were processed into single-cell suspensions by gently grinding through a 70 μm cell strainer. After centrifugation, red blood cells (RBCs) were lysed using RBC lysis buffer, followed by another centrifugation step and resuspension of the cells in pre-chilled PBS. Except for the analysis of IFN-γ⁺CD8⁺ T and GzmB⁺CD8⁺ T cells, the cell number was counted using a hemocytometer, and the cell concentration was adjusted to the required level before staining with the appropriate antibodies according to the manufacturer’s instructions. Post-incubation, cells were washed and resuspended in 200 μL of cold PBS for subsequent flow cytometric analysis.

For the analysis of IFN-γ⁺CD8⁺ T and GzmB⁺CD8⁺ T cells, after RBC lysis, the single-cell suspensions were resuspended in 10% DMEM medium containing ionomycin (1 μg/mL) and phorbol 12-myristate 13-acetate (PMA; 50 ng/mL) for stimulation over a period of 2 h. Following stimulation, brefeldin A (10 μg/mL) was added, and the samples were sealed for further incubation for an additional 4 h. After incubation, cells were centrifuged, resuspended in PBS, and stained with surface marker antibodies for 40 min. Post-staining, cells were fixed with 200–500 μL of 4% paraformaldehyde fixative solution for 15 min, followed by two washes with permeabilization buffer and resuspension in PBS. Intracellular proteins were stained at room temperature for 40 min. Finally, cells were washed, resuspended in 200 μL of PBS, and prepared for flow cytometric analysis. Flow-cytometry data were collected with CytExpert (ver. 2.3.0.84).

### In vitro 3D tumor spheroid assay

Three-dimensional Panc02 tumor spheroids were established as follows. Harvested Panc02 cells were resuspended in DMEM supplemented with 10% FBS at a density of 10^4^ cells/mL. Aliquots (200 μL) were dispensed into U-bottom 96-well plates (MS-9096UZ) and centrifuged at 2000 × *g* for 15 min. Spheroid formation was monitored during 48 h of culture. For establishing the bacterial infiltration model, 10^5^ CFU of VNP20009 was added to the spheroids and co-cultured for 3 h. To assess macrophage infiltration into tumor spheroids, harvested macrophages were labeled with Cy5.5 and seeded (10^6^ cells/well) into 6-well plates pre-coated with fibrinogen (Fg). Four experimental groups were designed: Groups 1 and 3: Untreated macrophages; Groups 2 and 4: Macrophages pretreated with 1 mM GB1275 for 1 h. Following pretreatment, tumor spheroids were added to Groups 1 and 2, while bacteria-infiltrated spheroids were introduced to Groups 3 and 4. All groups were incubated under standard conditions (37 °C, 5% CO_2_, 90% humidity) for 3 h, and macrophage infiltration was visualized via confocal microscopy. Images were collected with FluoView31S (ver.2.3.1.163). Neutrophil infiltration assays were performed identically.

### Preparation of Lip@V

In brief, DSPE-S-S-mPEG2000-NHS (3 mg), lecithin (2 mg), and stearic acid (1 mg) were dissolved in 1 mL of chloroform and added to a 10 mL round-bottom flask. The mixture was gently stirred for 10 min, followed by vacuum drying to remove chloroform, leaving a thin film on the flask wall. Subsequently, 200 μL of phosphate-buffered saline (PBS) containing gemcitabine (4 mg) was added to the flask under light protection, and the mixture was hydrated to form a clear solution. Following hydration, 100 μL of the aforementioned lipid solution was mixed with 1 mL of VNP20009 suspension (2 × 10^9^ CFU/mL) and incubated at 4 °C overnight with continuous stirring. The resulting Lip@V complexes were harvested by centrifugation, washed three times with PBS to remove any unincorporated components. Finally, the pellet was resuspended in 1 mL of PBS for further use.

### Whole-body fluorescence imaging

As for CD11b⁺ cell recruitment in Panc02 subcutaneous tumors, macrophages were labeled with Cy5.5, washed, and pretreated with 1 mM GB1275 for 1 h. Panc02 tumor-bearing mice were randomized into four groups: Groups 1 and 2: No VNP20009 pretreatment; Groups 3 and 4: Intravenous (*i.v*.) injection of 5 × 10^6^ CFU VNP20009. At 12 h post-injection, Groups 1 and 3 received *i.v*. administration of 5 × 10^6^ Cy5.5-labeled untreated macrophages, while Groups 2 and 4 received GB1275-pretreated macrophages. Whole-body fluorescence imaging was performed at predetermined time points. Neutrophil and Panc02 tumor recruitment assays were conducted using the identical protocol. As for Lip@V tumor targeting, Cy5.5-labeled Lip@V (1 × 10^7^ CFU) was administered intravenously, and whole-body fluorescence imaging was performed at sequential time points. As for Orthotopic Pancreatic tumor dual-mode imaging, following Cy5.5-Lip@V injection, mice were intraperitoneally administered 100 μL D-luciferin (300 μg/mL) at various points in time. Fluorescence and bioluminescence signals were acquired within 5–10 min using a multimodal imaging system.

### Viability assay of VNP20009

As for cytotoxicity of CD11b⁺ cells on VNP20009, macrophages and neutrophils were harvested and co-incubated with VNP20009 for 24 h. Following incubation, bacterial viability was assessed using the LB agar plate counting method. Panc02, CT26,  and 4T1 cells served as control groups to ensure that the assay conditions did not affect bacterial growth.

As for viability of GB1275 on VNP20009, different concentrations of GB1275 were incubated with VNP20009 (1 × 10^7^ CFU) for 24 h. Bacterial viability was determined using the CCK-8 assay. Specifically, VNP20009 was added to each well of a 96-well cell culture plate at a concentration of 1 × 10^7^ CFU per well, followed by the addition of different concentrations of GB1275. The cultures were incubated at 37 °C with shaking. At different time points, bacterial growth was monitored by measuring the absorbance at 600 nm (OD_600_) using a microplate reader. Additionally, after incubation for 24 h, bacterial survival was quantified using the LB agar plating method.

### In vitro cell viability assay

The cytotoxicity of GB1275 against cells was evaluated using the CCK-8 assay. 4T1 cells were harvested and seeded at a density of 1 × 10^4^ cells per well in a 96-well plate. After 24 h of incubation to allow cell attachment, the culture medium was replaced with serum-free medium containing various concentrations of GB1275. The cells were incubated for 6, 12, or 24 h, and cell viability was assessed using the CCK-8 assay. The same protocol was used to evaluate the cytotoxic effects of GB1275 on CT26 cells, Panc02 cells, 3T3 cells, and macrophages. Neutrophils were treated with GB1275 for 2 or 4 h under identical conditions, with the only difference being the incubation time.

The cytotoxicity of Lip@V against cells was also evaluated using the CCK-8 assay. Panc02 cells were harvested and seeded at a density of 1 × 10^4^ cells per well in a 96-well plate. Following 24 h of incubation to ensure cell adherence, the medium was replaced with serum-free medium containing different concentrations of VNP20009 or Lip@V. The cells were incubated for an additional 24 h, and cell viability was measured by the CCK-8 assay.

### Distribution of bacteria in vivo

For the biodistribution study of Lip@V, Panc02 pancreatic cancer-bearing mice were injected intravenously via the tail vein with 5 × 10^6^ CFU Lip@V. At predetermined time points, the mice were euthanized and major organs along with tumors were harvested under sterile conditions. The collected tissues were homogenized using a tissue dissociator. The resulting homogenates were serially diluted in PBS and plated onto LB agar plates. After incubation at 37 °C for 24 h, bacterial colonies were counted, and the bacterial concentration was calculated according to colony counts, corresponding dilution factors, and tissue weights.

In a separate experiment to evaluate the effect of GB1275 on bacterial distribution, Panc02 tumor-bearing mice were randomly divided into two groups. One group received intraperitoneal injections of GB1275 at a concentration of 4 mg/mL continuously, while the other group was administered PBS as a control. On the third day, both groups were injected intravenously via the tail vein with 5 × 10^6^ CFU of VNP20009. At designated time points, the mice were euthanized, and tumors were harvested under sterile conditions. The tumors were homogenized using a tissue dissociator, and the homogenates were plated onto LB agar plates. Following a 24 h incubation period at 37 °C, bacterial colonies were enumerated. The bacterial concentration was determined according to colony counts, the appropriate dilution factors, and the weight of the tumor tissues.

### In vivo anti-tumor experiments

To investigate the enhancement of VNP20009 anti-tumor efficacy by GB1275, Panc02 pancreatic cancer-bearing mice were randomly divided into four groups and treated as follows: On day 7, groups 2 and 4 were given continuous intraperitoneal injection of GB1275 (4 mg/mL) until the end of the treatment. On day 10, groups 3 and 4 were injected intravenously via the tail vein with 5 × 10^6^ CFU of VNP20009, while groups 1 and 2 were administered 100 μL of PBS as controls. Tumor volumes and mouse weights were measured every two days, and photographs of the mice were taken every four days.

To assess the promotion of intratumoral VNP20009 survival by GB1275, Panc02 tumor-bearing mice were administered with PBS, GB1275, 3-Methyladenine, RS 504393, clodronate liposomes and anti-Ly6G antibody according to the standard experimental protocol. After 72 h post-administration, 5 × 10^6^ CFU VNP20009 was injected via the tail vein. Tumor tissues were harvested at different time points, homogenized, and subjected to colony counting using the LB agar plate counting method. In a parallel assay, VNP20009 was replaced with mCherry-expressing VNP20009, the above procedures were repeated, and the tumor tissues were collected for fluorescent staining analysis.

#### Enhancement of Lip@V anti-tumor efficacy by GB1275

To explore the enhancement of Lip@V anti-tumor efficacy by GB1275, Panc02 tumor-bearing mice were randomized into five groups and treated as follows: On day 7, groups 2, 4 and 5 were given continuous intraperitoneal injection of GB1275 (4 mg/mL) to the treatment endpoint. On day 10, groups 3 and 4 were injected intravenously with 5 × 10^6^ CFU of Lip@V, group 5 was injected with an equivalent dose of Lip alone, and groups 1 and 2 were given 100 μL of PBS as controls. As before, tumor volumes and mouse weights were monitored every two days, and photographic documentation was performed every four days. For the treatment of advanced tumors, the advanced tumor therapy experiment was initiated on day 10 after tumor inoculation, with all other treatments consistent with the above protocols. For the mouse survival experiment, a tumor volume exceeding 1500 mm^3^ was defined as the endpoint criterion, and the survival status of mice treated with different treatment combinations was continuously monitored for a 30-day experimental observation period.

### Enhancement of Lip@V anti-tumor efficacy against in situ pancreatic cancer by GB1275

For orthotopic Panc02 pancreatic cancer models, mice were randomly assigned to five groups and treated following the same protocol as the Lip@V anti-tumor efficacy enhancement assay described previously. Additionally, on day 7 and 19, each mouse was intraperitoneally injected with 100 μL of D-luciferin at a concentration of 3 mg/mL. Bioluminescence imaging was conducted within 5–10 min after intraperitoneal injection using an in vivo imaging system to quantify tumor burden.

### In vivo safety assessment

To evaluate the biosafety of Lip@V, female C57BL/6 J healthy mice were randomly divided into three groups. Two experimental groups received intravenous injections of 5 × 10^6^ CFU of Lip@V via the tail vein on day 0 and 6, while the control group received no treatment. On day 7, blood samples (400 µL per mouse) were collected from the hearts of the mice. Of these samples, 300 µL were placed into tubes containing anticoagulant and subjected to centrifugation for 10 min. The resulting supernatant was used for liver and kidney function analysis. Additionally, 100 µL of whole blood from each mouse was utilized for routine hematological testing. Upon completion of the GB1275-enhanced Lip@V anti-tumor experiment, the mice were euthanized, and their heart, liver, spleen, lung, and kidney tissues were harvested. These organs were fixed in 4% paraformaldehyde, dehydrated, embedded, sectioned, and subsequently stained with hematoxylin and eosin (H&E). Histopathological examinations were performed using an optical microscope to assess any potential tissue damage or abnormalities.

### Tumor tissue RNA extraction

Upon completion of the GB1275-enhanced Lip@V anti-tumor experiment, tumor tissues were collected, immediately snap-frozen in liquid nitrogen, and ground into a fine powder. RNA isolation reagent (1 mL) was added to the powdered tissue, which was incubated on a shaker for 2 h to facilitate complete lysis. After the incubation period, the samples were centrifuged to collect the tissue lysate, and 200 μL of chloroform was added to the solution. The mixture was vigorously vortexed for 15 seconds to form an emulsion and subsequently placed on ice for 5 min. The samples were centrifuged at 4 °C at 12,000 × g for 15 min. The upper aqueous phase was carefully transferred to a new centrifuge tube, and an equal volume of pre-chilled isopropanol was added. The mixture was gently inverted several times to mix and left on ice for 10 min. Following this, the samples underwent another centrifugation at 4 °C at 12,000 × *g* for 10 min. After discarding the supernatant, the RNA pellet was washed with 1 mL of 75% ethanol (prepared with RNase-free ddH_2_O). The tubes were inverted several times and allowed to stand at room temperature for 3–5 min before being centrifuged again at 4 °C at 1,200 g for 10 min. After removing the supernatant, the RNA pellet was dried for 2–5 min and dissolved in an appropriate volume of RNase-free ddH_2_O. Finally, RNA concentration was quantified using a microspectrophotometer.

### Statistics and reproducibility

Experiments in this manuscript were performed with at least three replicates in Figs. 3B, C, 4F–H, 5B–D, 5O, P and 7F–H. Data are expressed as mean ± S.D. Significance between two groups was assessed by unpaired two-tailed Student’s *t* test. For analyses involving multiple groups, one-way or two-way ANOVA with Tukey’s multiple comparisons test was performed. Values with *P* < 0.05 were considered significant. Exact *P* values were provided accordingly in the figures. All the statistical analyses were performed using GraphPad Prism (8.0). In this study, the sample size was determined based on prior experimental experience and standard practices. We did not use statistical methods to predetermine the sample size. During data analysis, no data were excluded. We have provided a detailed description of the experimental methods to ensure that other researchers can replicate the experiment and verify the reproducibility of the results. Flow-cytometry data were analyzed with FlowJo (ver. 10.8.1). ImageJ (ver. 1.4.3.67) was used to analyze fluorescent grayscale. Confocal images were analyzed with FluoView31S (ver.2.3.1.163). The plots were assembled with Coreldraw x8 (ver. 18.0.0.450).

### Reporting summary

Further information on research design is available in the Nature Portfolio Reporting Summary linked to this article.

## Supplementary information

Supplementary Information

Reporting Summary

Transparent Peer Review file

## Source data

Source data

## Data Availability

All data generated or analyzed during this study are included in this published article and its Supplementary Information file. The raw RNA-seq sequencing data generated in this study have been deposited in the NCBI Sequence Read Archive (SRA) under BioProject accession PRJNA1501942. Processed gene expression matrices are available on Figshare (DOI: 10.6084/m9.figshare.32943350). Source data are provided with this paper.
