# Supplementary material for: CD11b agonist GB1275 potentiates oncolytic bacterial therapy against pancreatic cancer in mouse tumor models
Source: Nat Commun. 2026 Aug 21;17:10739. doi: 10.1038/s41467-026-76823-7 (PMC13661619; doi:10.1038/s41467-026-76823-7)
Supplement: Supplementary file 1 [file 41467_2026_76823_MOESM1_ESM.pdf]

Supplementary Information

CD11b agonist GB1275 potentiates oncolytic bacterial therapy against pancreatic cancer in mouse tumor models

Bin Zhang[1,#], Meng Guan[1,#], Xiao-Ting Xie[1,#], Jia-Hua Zou[1,2,3], Kai Cheng[4,*], Yuan-Di Zhao[1,*], Bo Liu[1,*], Jin-Xuan Fan[1,*], Qiu-Ran Xu[5,*]

[1] MOE Key Laboratory for Biomedical Photonics - Hubei Bioinformatics & Molecular Imaging Key Laboratory, Department of Biomedical Engineering, College of Life Science and Technology, Huazhong University of Science and Technology, Wuhan 430074, Hubei, P.R. China

[2] Department of Oncology, Huanggang Central Hospital of Yangtze University, No.126 Qi'an Road, HuangZhou District, Huanggang City 438000, Hubei, P.R. China

[3] Hubei Clinical Medical Research Center of Esophageal and Gastric Malignancy, Huanggang City, Hubei, 438021, P.R. China

[4] Department of Chemistry, The Chinese University of Hong Kong, Hong Kong SAR 99999, P.R.China

[5] Zhejiang Key Laboratory of Tumor Molecular Diagnosis and Individualized Medicine, Zhejiang Provincial People's Hospital, Affiliated People's Hospital, Hangzhou Medical College, Hangzhou 310014, Zhejiang, P.R. China

[#] These authors contributed equally: Bin Zhang, Meng Guan, Xiao-Ting Xie.

*Correspondence: kaicheng@cuhk.edu.hk (K. Cheng); zydi@mail.hust.edu.cn (Y.D. Zhao); lbyang@mail.hust.edu.cn (B. Liu); jxfan@hust.edu.cn (J.X. Fan); xuqiuran@hmc.edu.cn (Q.R. Xu)

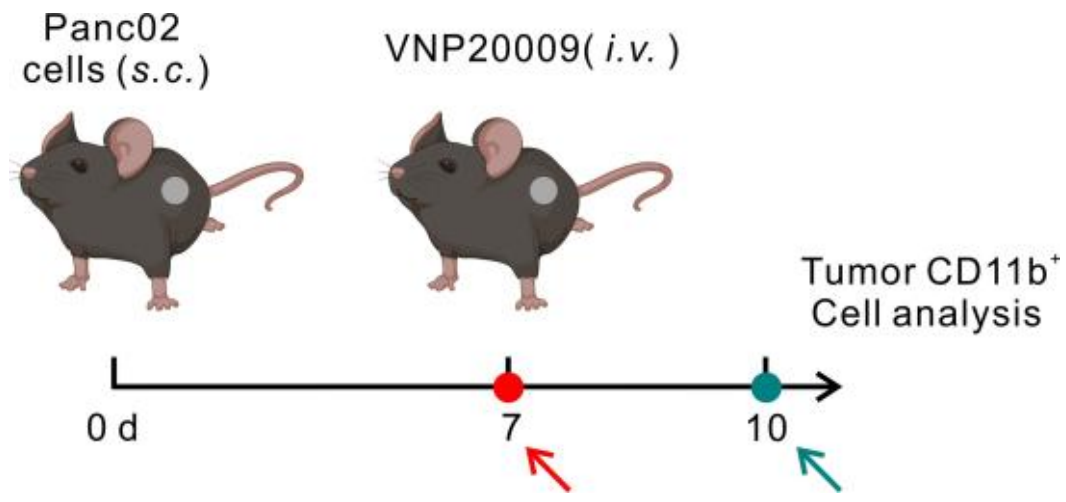

Supplementary Fig. 1 Schematic illustration of the in vivo administration regimen of Panc02 tumor model. *s.c.*, subcutaneous injection, *i.v.*, intravenous injection. This figure was Created in BioRender. Zhang, B. (2026) https://BioRender.com/79b3hb8.

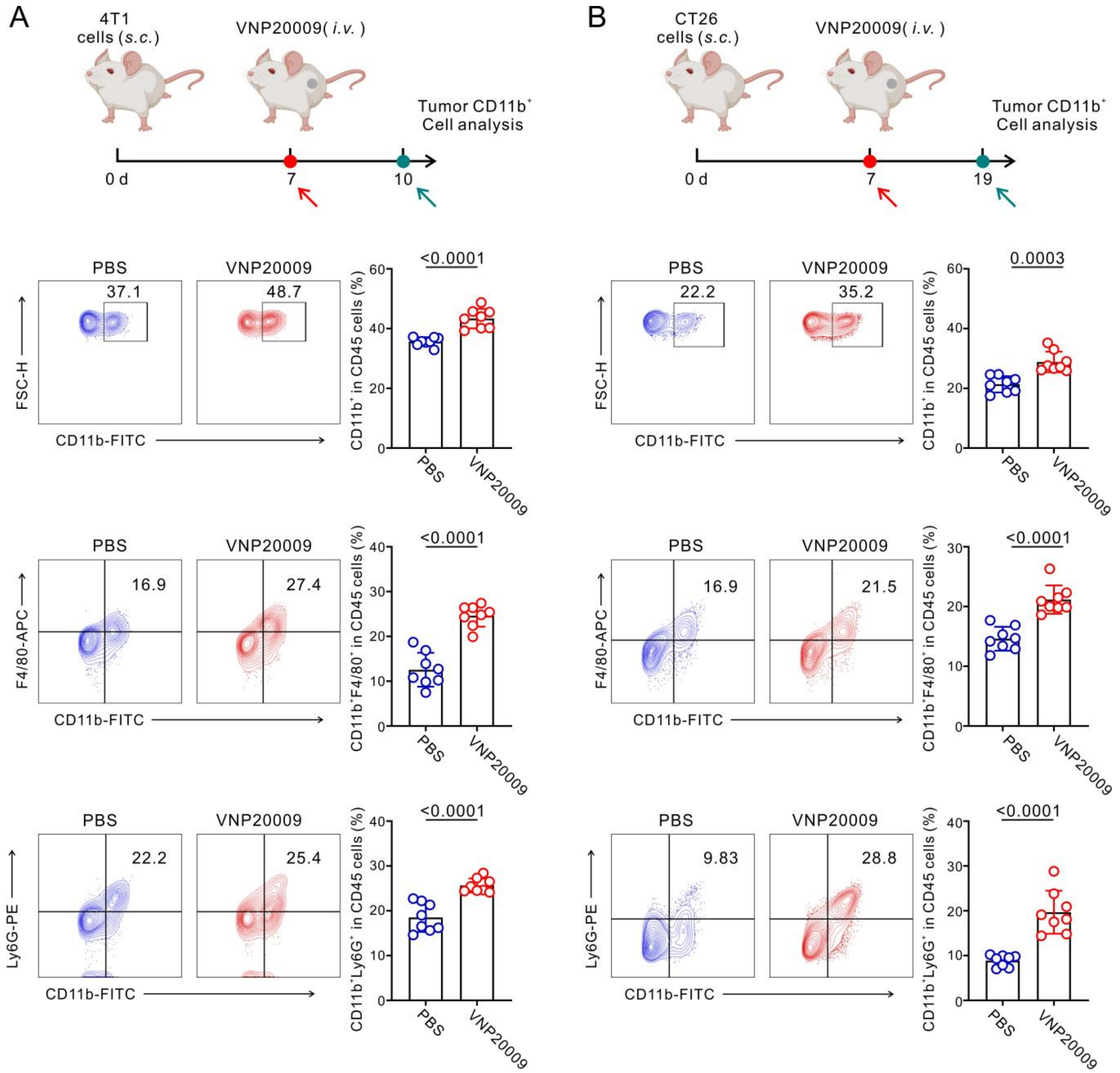

Supplementary Fig. 2 Schematic illustration of the in vivo administration regimen of VNP20009, Flow cytometry analysis and corresponding statistics of the total CD11b$^+$ cells, macrophages, and neutrophils within (**A**) 4T1 tumor tissue and (**B**) CT26 tumor tissue after bacteria administration at 48 h. 5×10^6 CFU of VNP20009 was administered intravenously via the tail vein, while the control group received PBS. *s.c.*, subcutaneous injection. *i.v.*, intravenous injection, *n* = 8 independent samples. The data are expressed as mean ± s.d. All replicates are biological, and the *P* values were determined using

two-tailed Student's t test. Source data are provided as a Source Data file. Figure A, B was created in BioRender. Zhang, B. (2026) https://BioRender.com/79b3hb8.

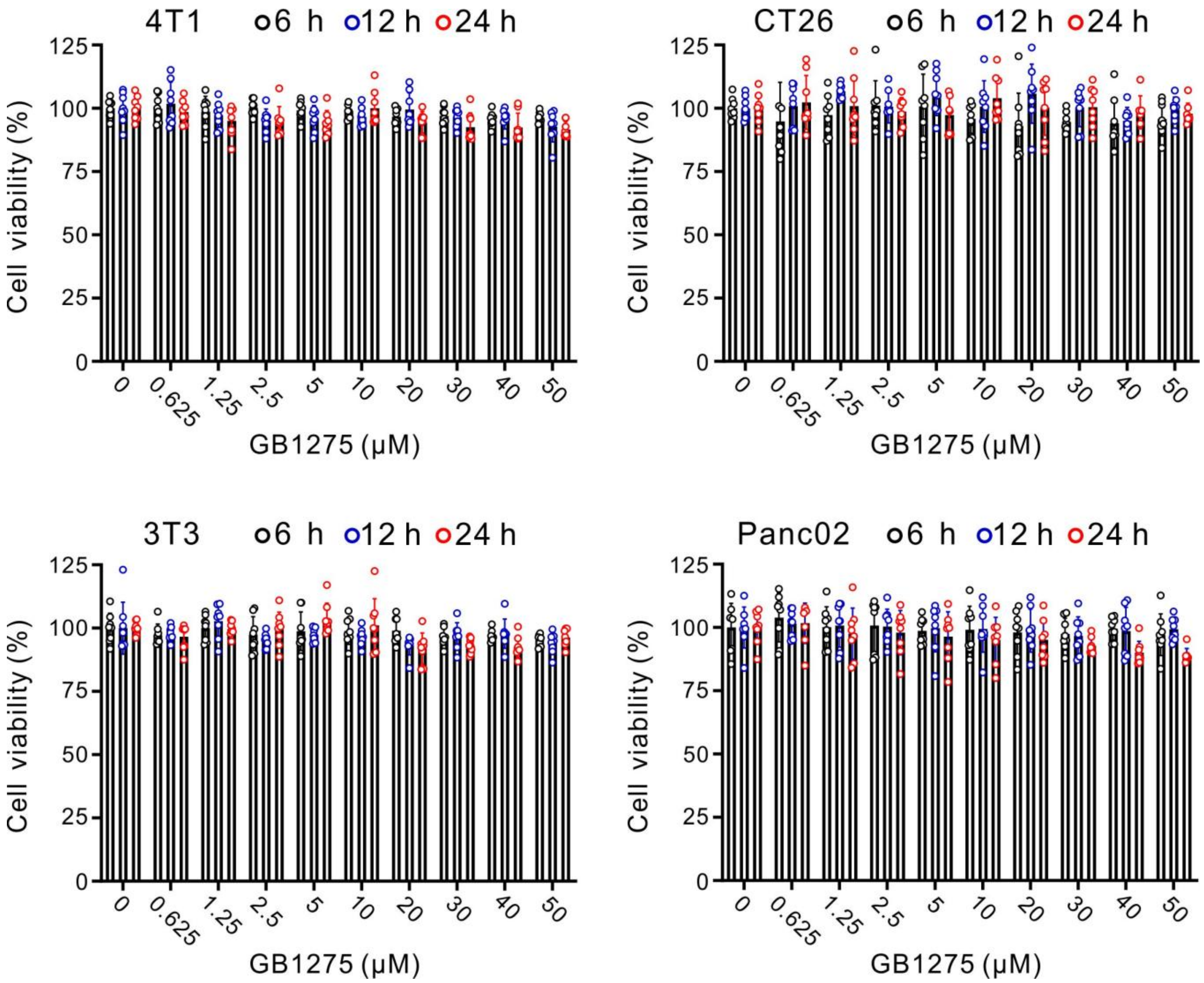

Supplementary Fig. 3 Cytotoxic effects of GB1275 on different cells at indicated concentrations and time points, assessed by the CCK-8 assay, n = 8 independent samples. Source data are provided as a Source Data file.

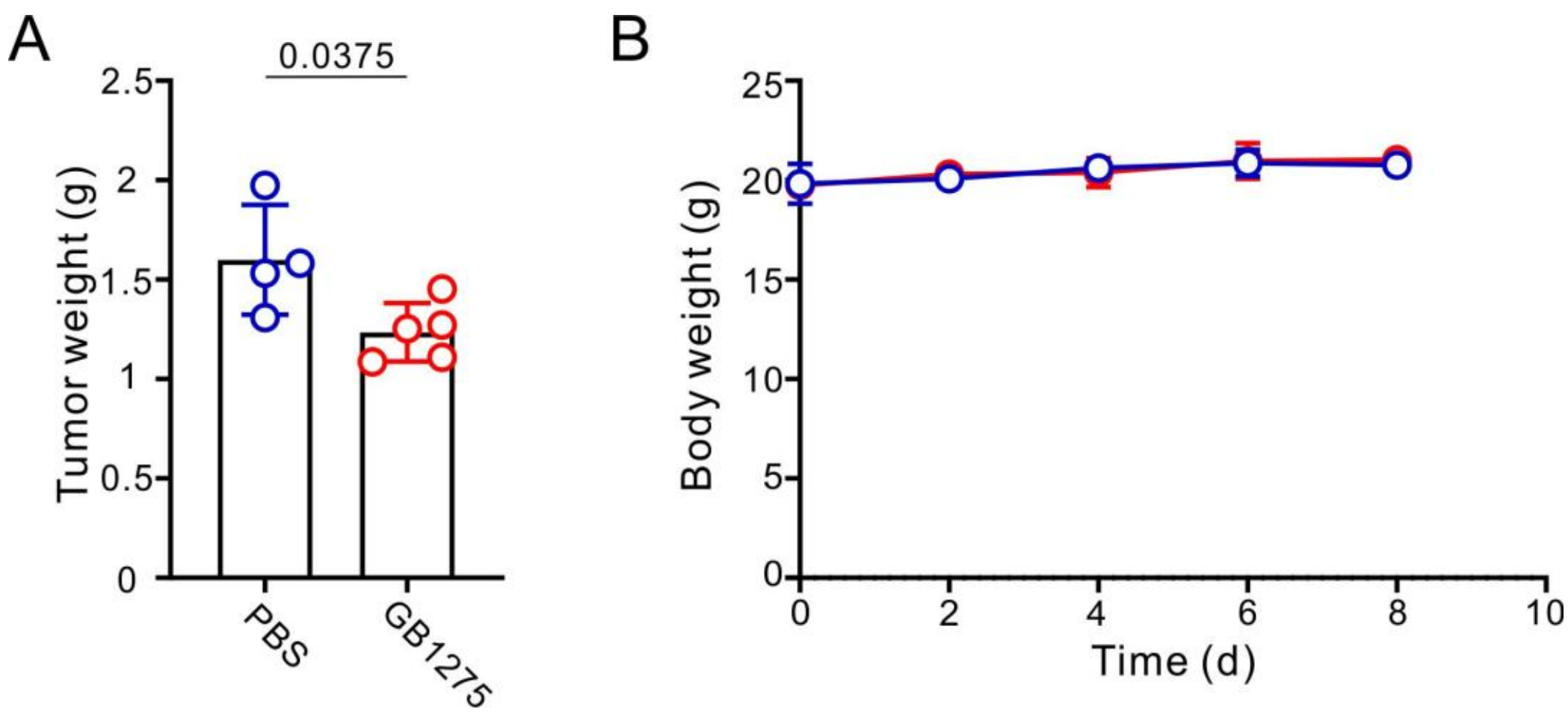

Supplementary Fig. 4 **A** Statistics of isolated tumor weights after the end of treatment, *n* = 4 independent samples in PBS group; *n* = 5 independent samples in GB1275 group. **B** Changes in mouse body weights during the administration of GB1275 in Panc02 subcutaneous tumor mouse model, *n* = 5 independent samples. The data are expressed as mean ± s.d. All replicates are biological, and the *P* value in A was determined using two-tailed Student's t test. Source data are provided as a Source Data file.

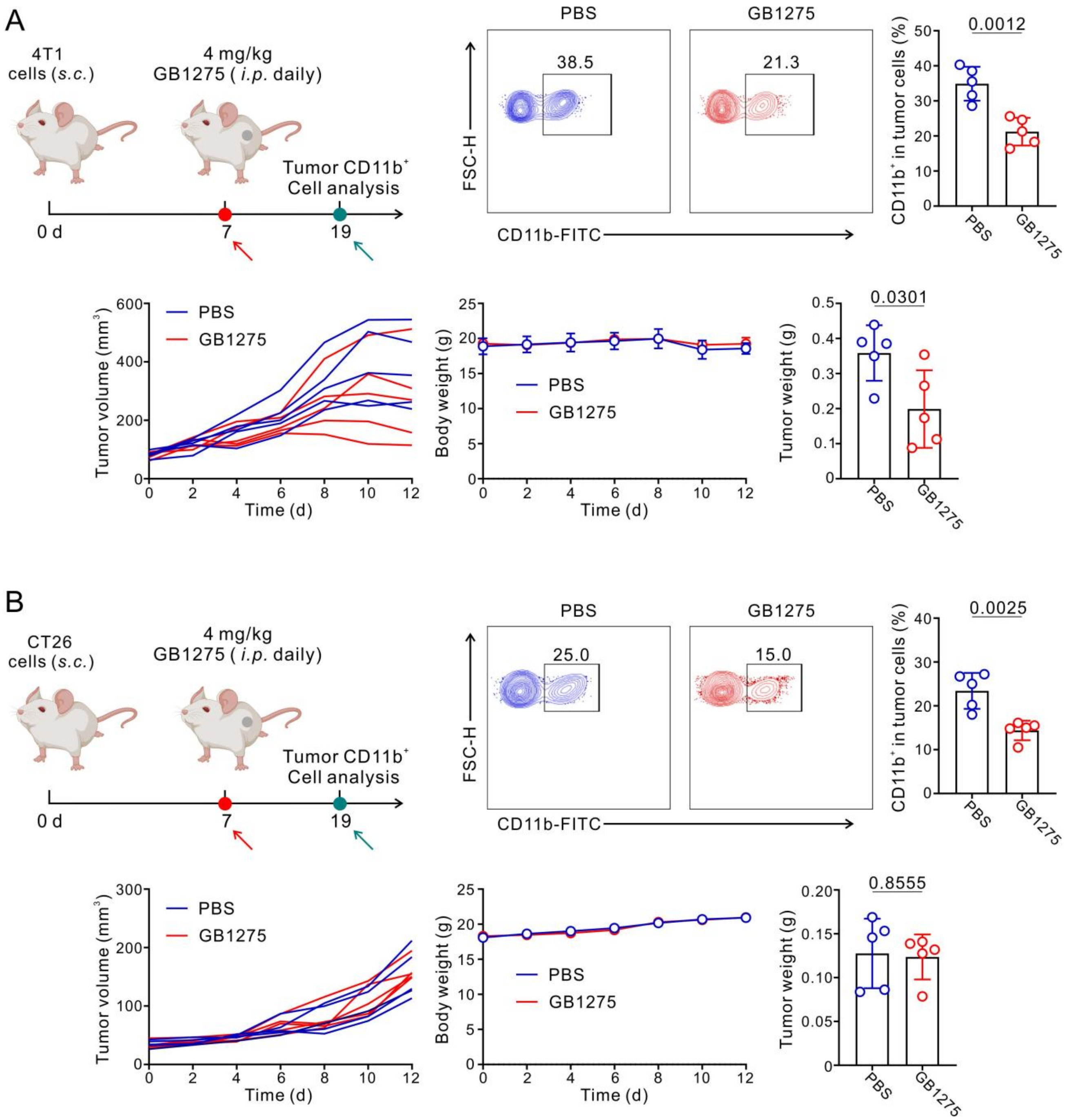

Supplementary Fig. 5 The information in the figure including: Schematic illustration of the in vivo administration regimen of GB1275 in (**A**) 4T1 subcutaneous tumor model and (**B**) CT26 subcutaneous tumor model, with an equal volume of PBS as a control. *s.c.*, subcutaneous injection. *i.p.* daily, daily intraperitoneal injection. Flow cytometry analysis and corresponding statistics of CD11b$^+$ cells in tumor tissues after the administration of GB1275 for 8 days. Tumor growth curve and changes in

mouse body weights during the administration of GB1275. Statistics of isolated tumor weights after the end of treatment, $n = 5$ independent samples. The data are expressed as mean ± s.d. All replicates are biological, and the P values were determined using two-tailed Student's t test. Source data are provided as a Source Data file. Figure A, B was was created in BioRender. Zhang, B. (2026) https://BioRender.com/79b3hb8.

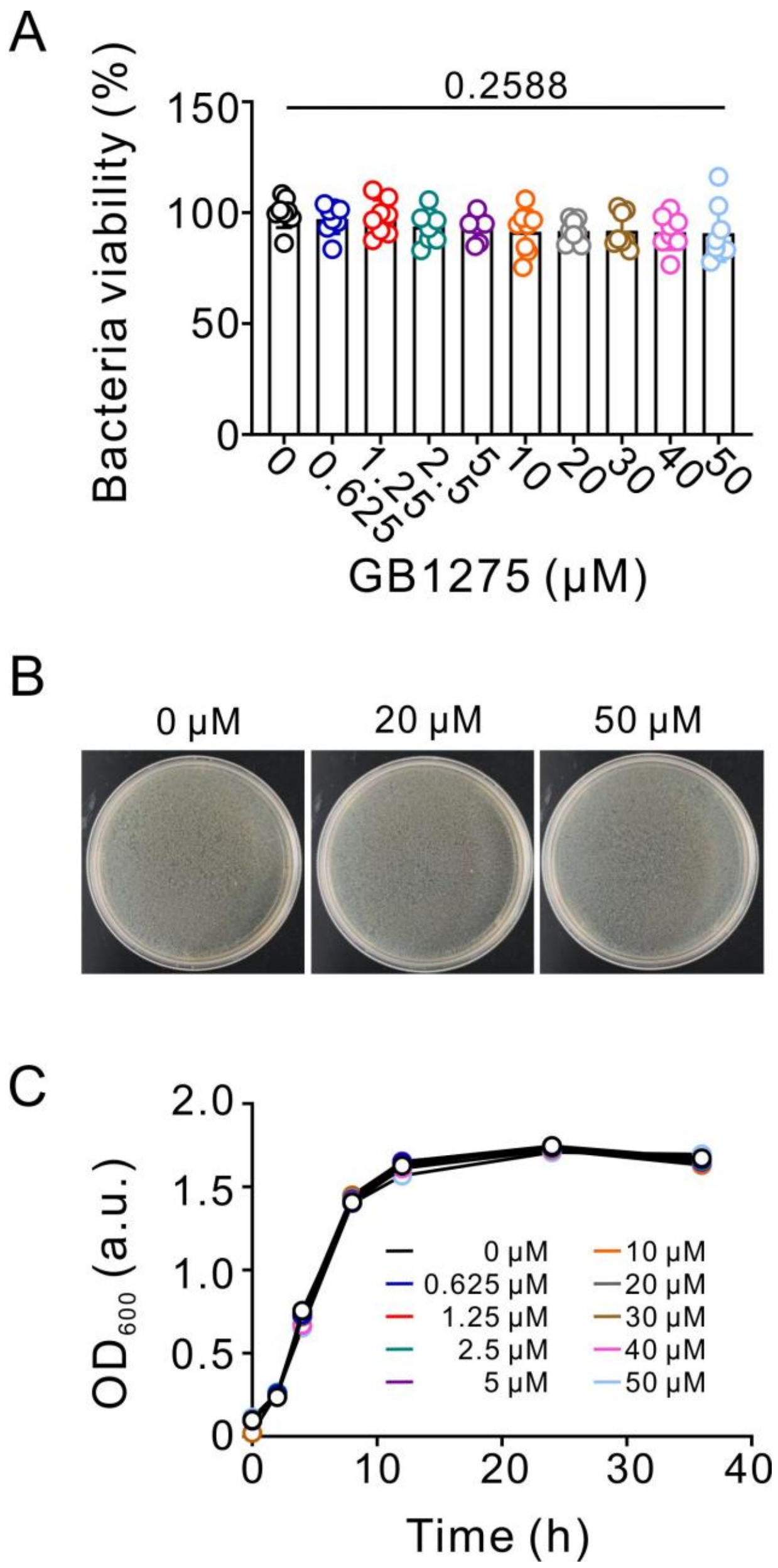

Supplementary Fig. 6 **A** Cytotoxic effects of GB1275 on VNP20009 at indicated concentrations and time points, assessed by the CCK-8 assay, n = 8 independent samples. **B** Representative photographs of bacterial cultures after incubation of bacteria with different concentrations of GB1275 for 24 h. **C** The growth curves of VNP20009 after incubation with different concentrations of GB1275, n = 8 independent samples. The data are expressed as mean ± s.d. All replicates are biological, and the P value in A was determined using one-way ANOVA followed by Tukey's multiple comparisons tests. Source data are provided as a Source Data file.

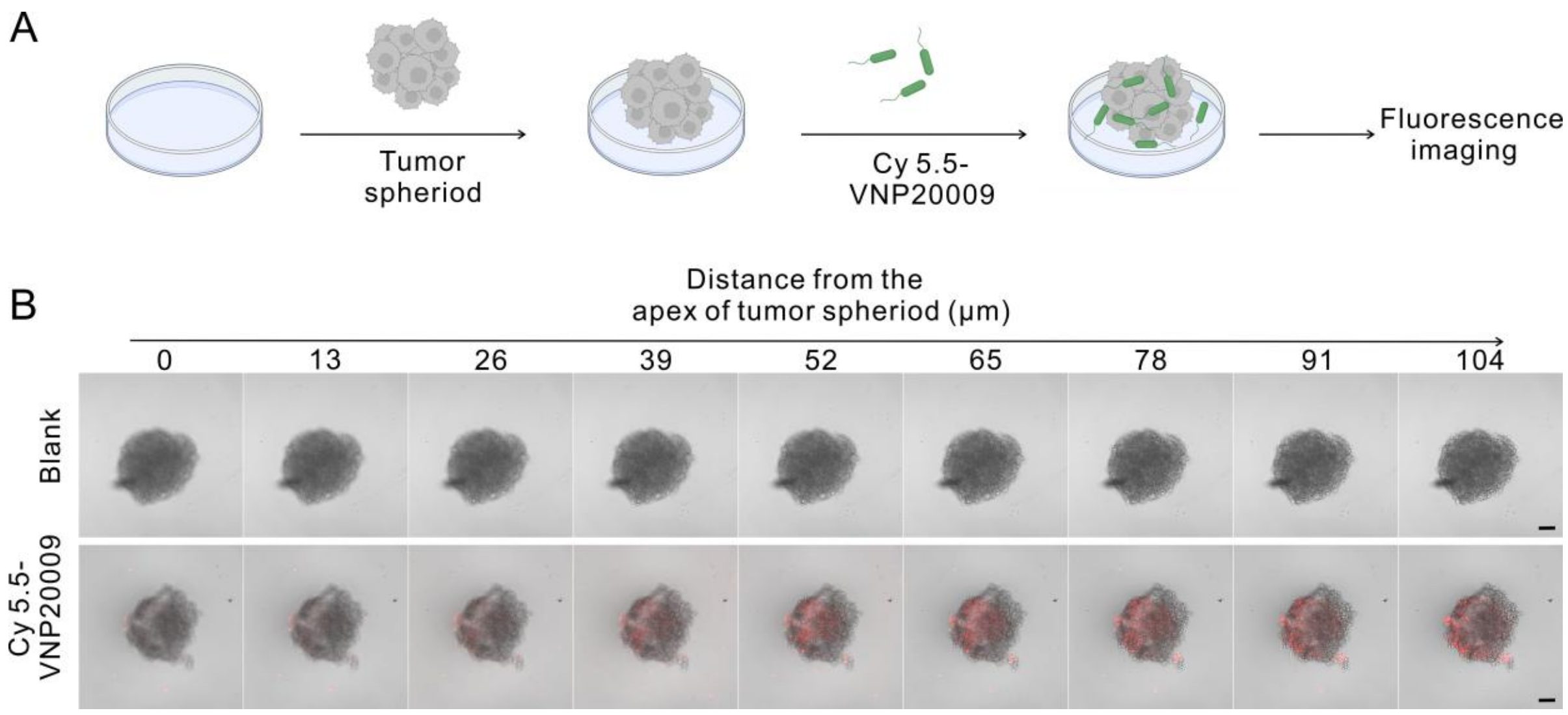

Supplementary Fig. 7 **A** Schematic illustration of the construction of bacteria-infiltrated tumor spheroid. **B** Confocal fluorescence imaging of Cy5.5-labeled VNP20009 infiltration into tumor spheroids. Scale bar: 20 μm. Source data are provided as a Source Data file. Figure A was created in BioRender. Zhang, B. (2026) https://BioRender.com/79b3hb8.

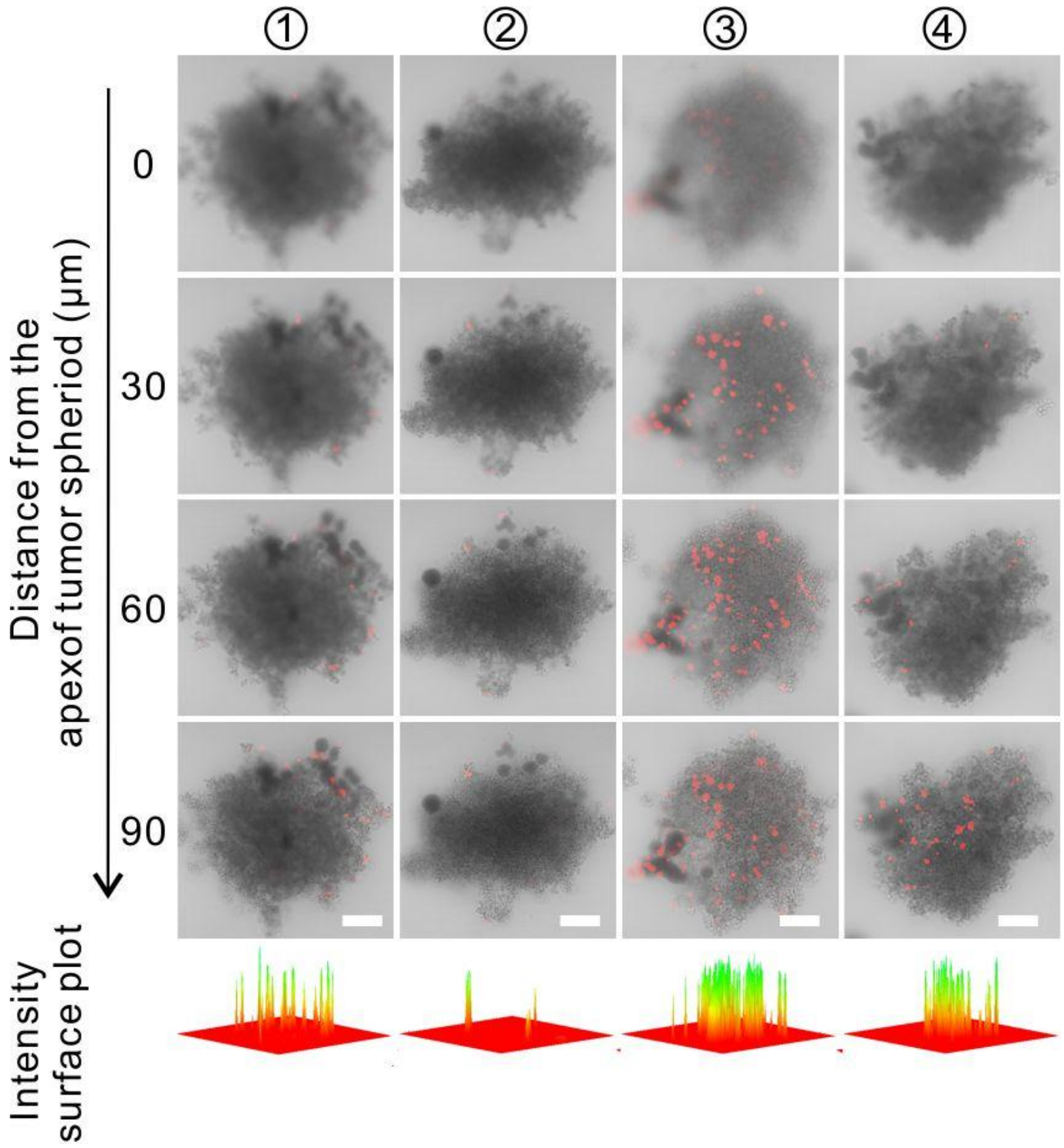

Supplementary Fig. 8 Representative confocal fluorescence imaging of tumor spheroids/bacterial-infiltrated tumor spheroids infiltration by macrophages after different treatments. The red fluorescence represents Cy5.5-labeled macrophages. The bottom panel is a 3D surface plot of Cy5.5-labeled macrophages. Scale bar: 20 μm.

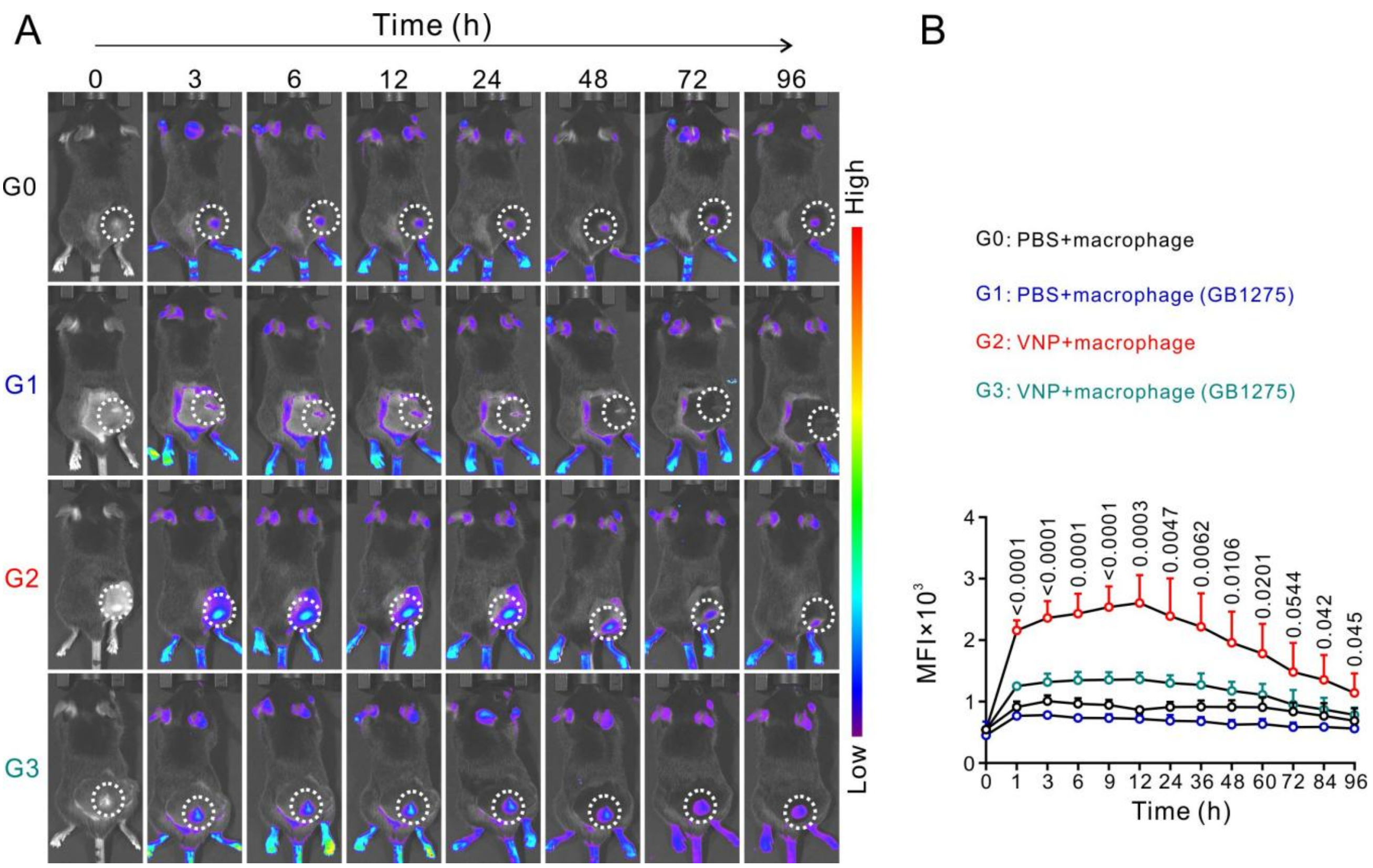

Supplementary Fig. 9 **A** Representative whole-body fluorescence imaging dynamically monitored the tumor infiltration of macrophages after different experimental treatments. The dotted circle represents the tumor sites. **B** Fluorescence intensity over time curves at tumor sites after different experimental treatments, and the *P* values obtained in the chart represent the fluorescence statistical analysis of the tumor between G2 and G3, *n* = 5 independent samples. The data are expressed as mean ± s.d. All replicates are biological, and the *P* values in B were determined using two-tailed Student's t test. Source data are provided as a Source Data file.

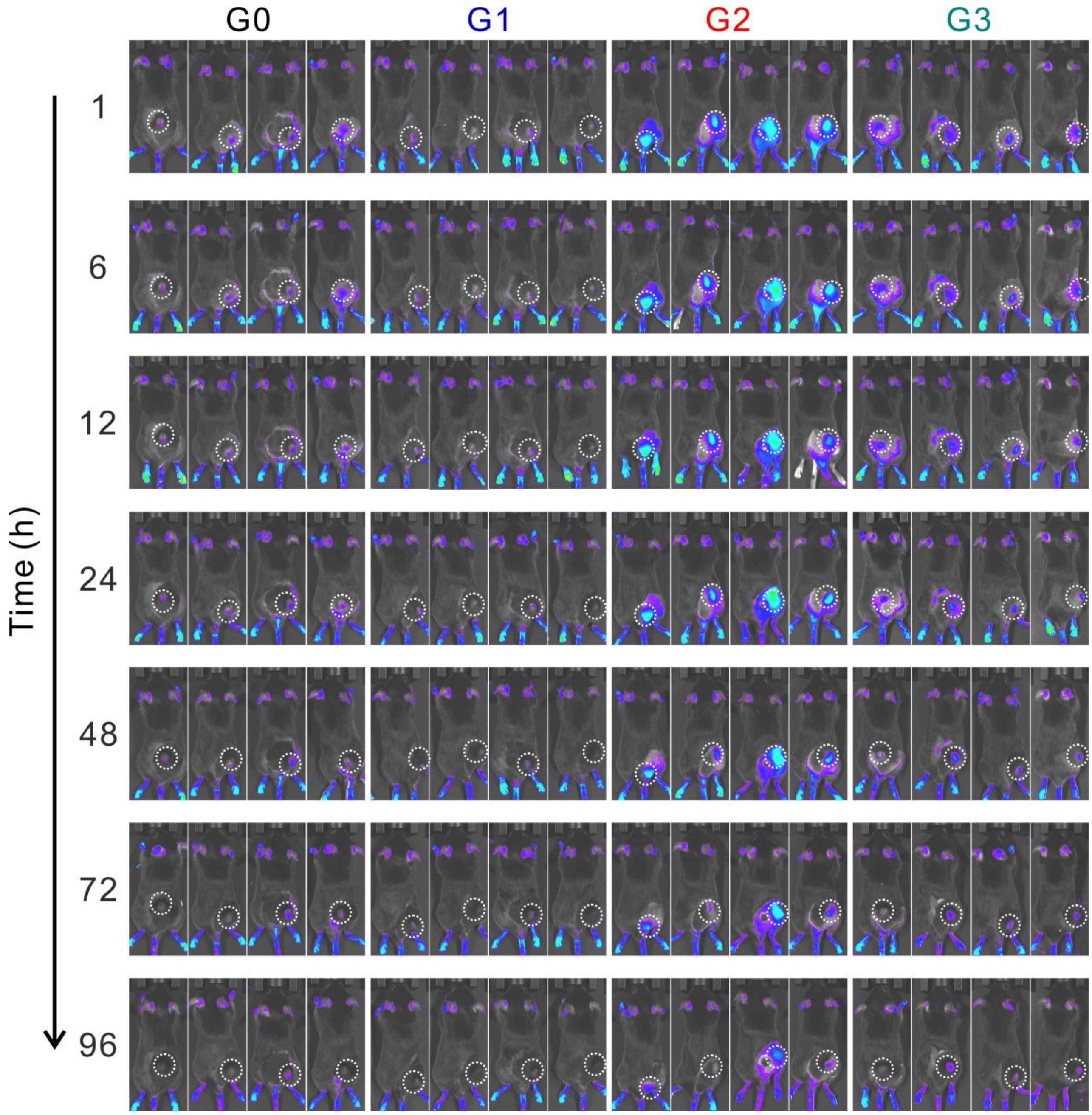

Supplementary Fig. 10 Whole-body fluorescence imaging dynamically monitored the tumor infiltration of macrophages after different experimental treatments. This is another four images corresponds to Supplementary Fig. 9. The dotted circle represents the tumor site.

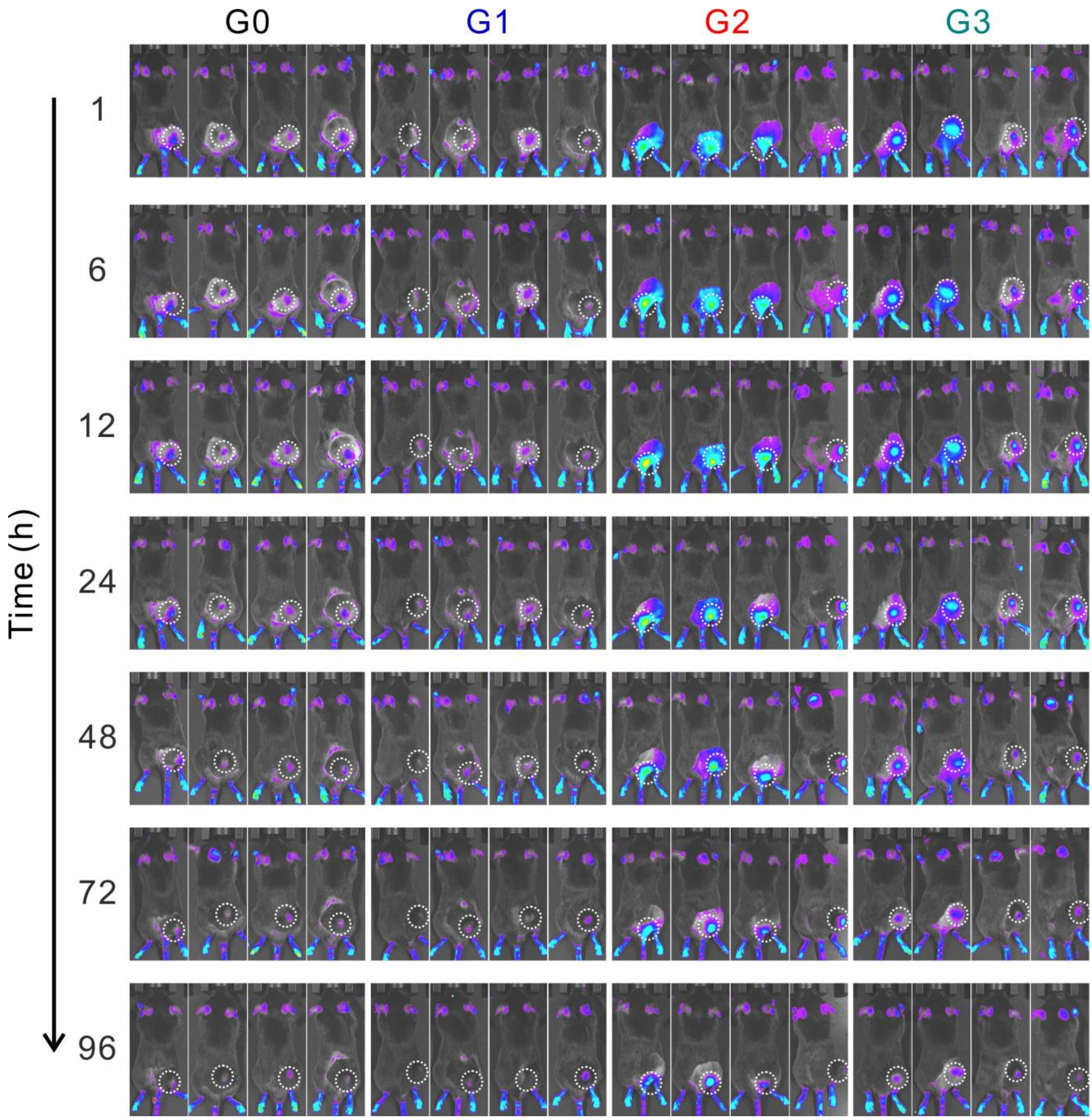

Supplementary Fig. 11 Whole-body fluorescence imaging dynamically monitored the tumor infiltration of neutrophils after different experimental treatments. This is another four images corresponds to Fig. 3C. The dotted circle represents the tumor site.

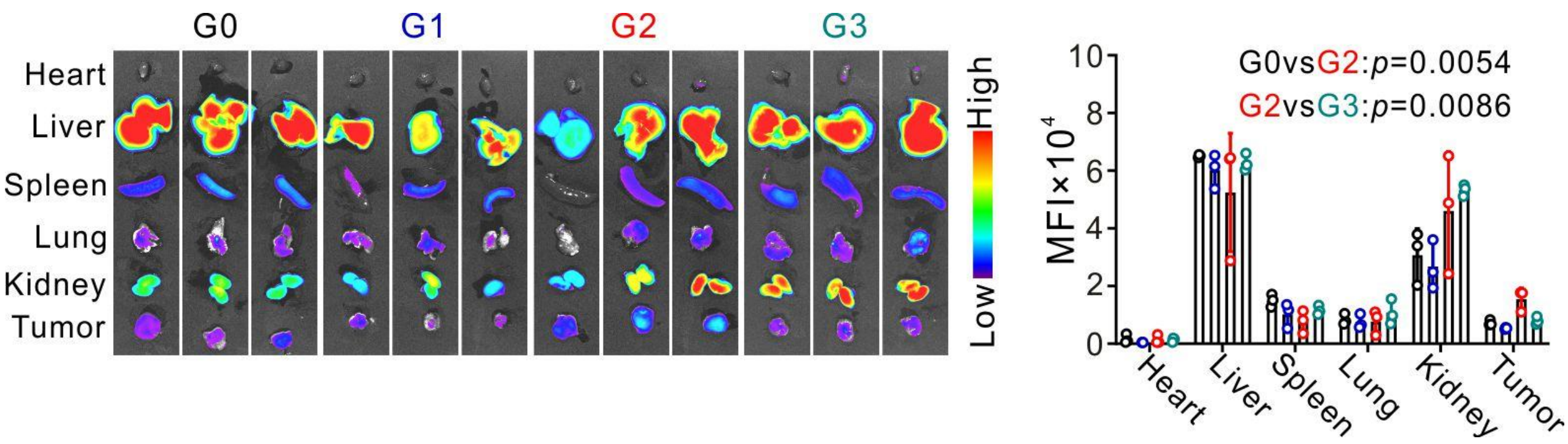

Supplementary Fig. 12 Fluorescence imaging of major organs and corresponding quantitative fluorescence statistics were performed at 48 h after injection of Cy5.5-macrophages. vs is the abbreviation for versus, used for comparison between two groups. The *P* values obtained in the chart represent the fluorescence statistical analysis of the tumor, *n* = 3 independent samples. The data are expressed as mean ± s.d. All replicates are biological, and the *P* values were determined using one-way ANOVA followed by Tukey's multiple comparisons tests. Source data are provided as a Source Data file.

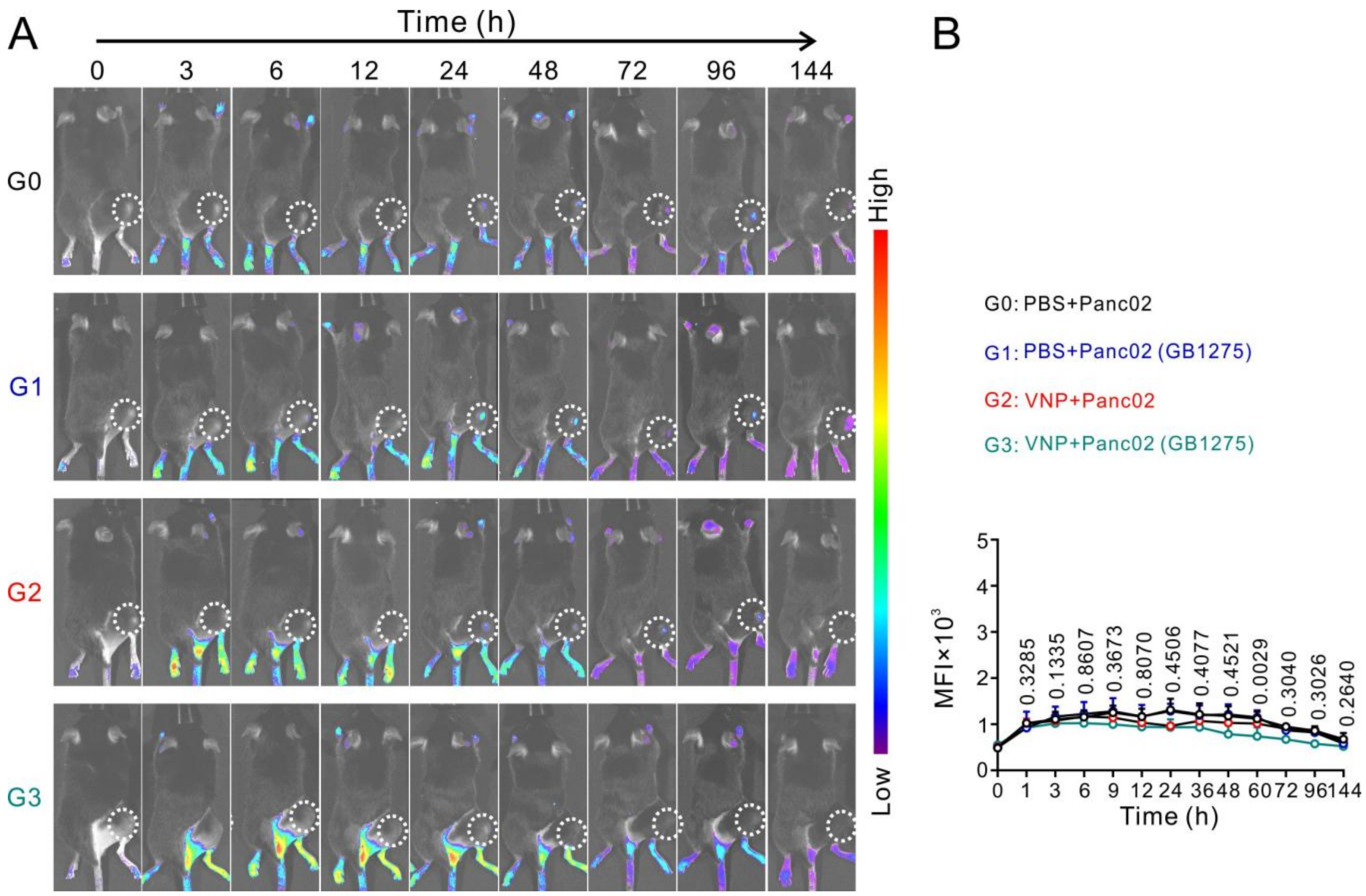

Supplementary Fig. 13 **A** Representative whole-body fluorescence imaging dynamically monitored the tumor infiltration of Panc02 cells after different experimental treatments. The dotted circle represents the tumor site. **B** Fluorescence intensity over time curves at tumor sites after different experimental treatments, and the *P* values obtained in the chart represent the fluorescence statistical analysis of the tumor between G2 and G3, $n = 3$ independent samples. The data are expressed as mean ± s.d. All replicates are biological, and the *P* values in B were determined using two-tailed Student's t test. Source data are provided as a Source Data file.

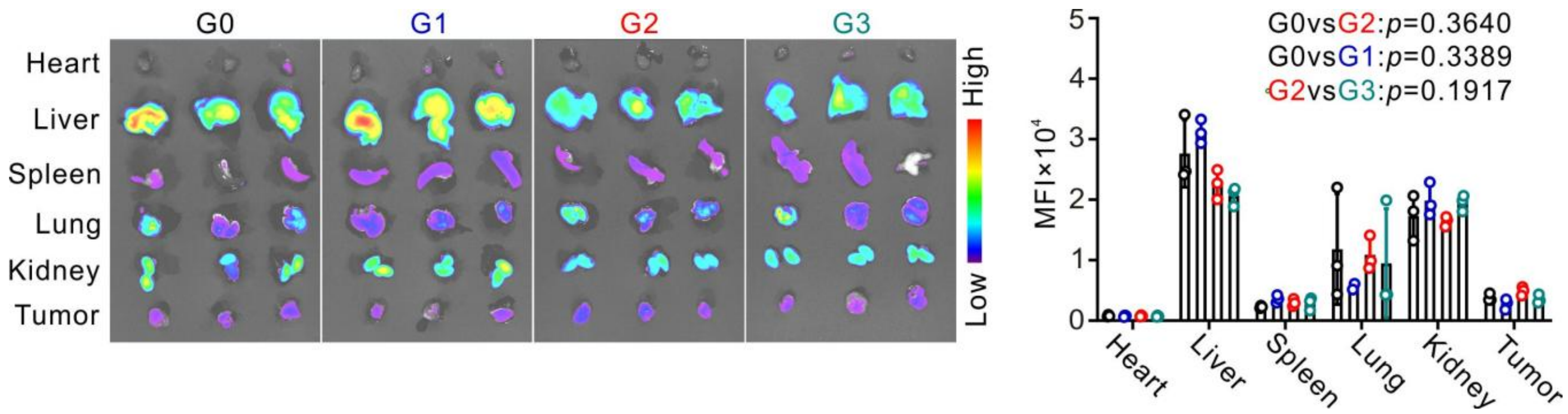

Supplementary Fig. 14 Fluorescence imaging of major organs and corresponding quantitative fluorescence statistics were performed at 48 h after injection of Cy5.5-Panc02. vs is the abbreviation for versus, used for comparison between two groups. The bar chart shows the fluorescence statistics of the corresponding organs, and the *p* values obtained in the chart represent the fluorescence statistical analysis of the tumor, $n = 3$ independent samples. The data are expressed as mean ± s.d. All replicates are biological, and the *P* values were determined using one-way ANOVA followed by Tukey's multiple comparisons tests. Source data are provided as a Source Data file.

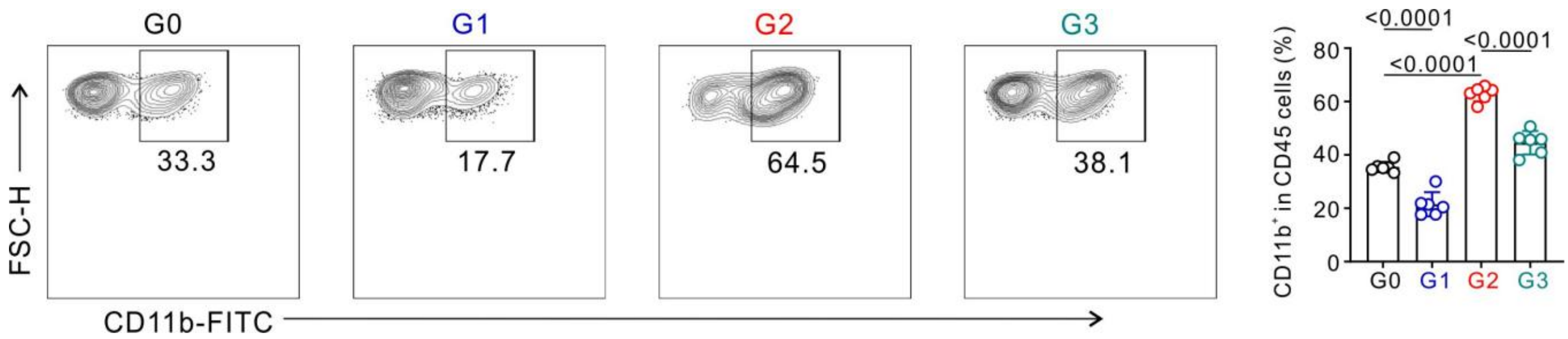

Supplementary Fig. 15 Flow cytometry and corresponding statistics of the total CD11b^{+} cells in the tumor tissue after different treatments, *n* = 6 independent samples. G0: Blank; G1: GB1275; G2: VNP20009; G3: GB1275 + VNP20009. The data are expressed as mean ± s.d. All replicates are biological, and the *P* values was determined using one-way ANOVA followed by Tukey's multiple comparisons tests. Source data are provided as a Source Data file.

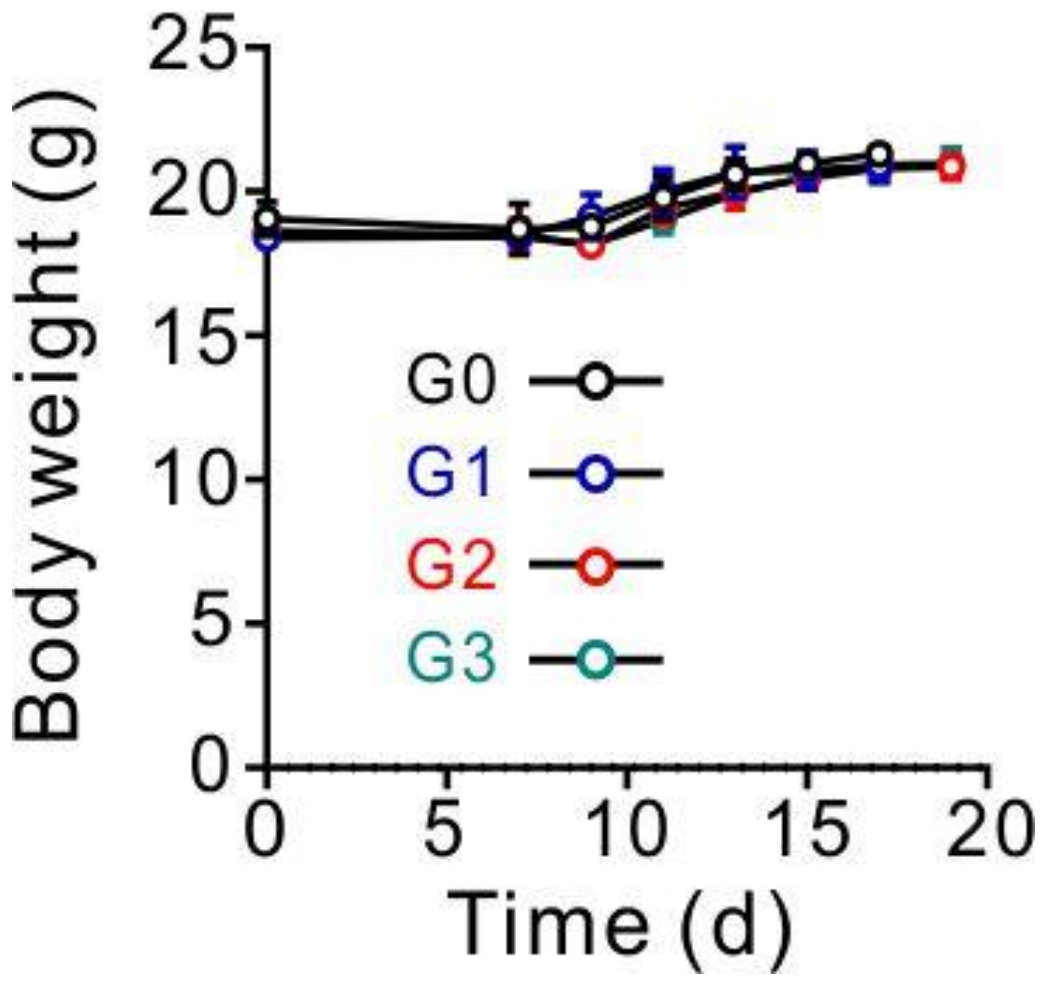

Supplementary Fig. 16 Changes in mouse body weights after different treatments. *n* = 5 independent samples. G0: Blank; G1: GB1275; G2: VNP20009; G3: GB1275 + VNP20009. Source data are provided as a Source Data file.

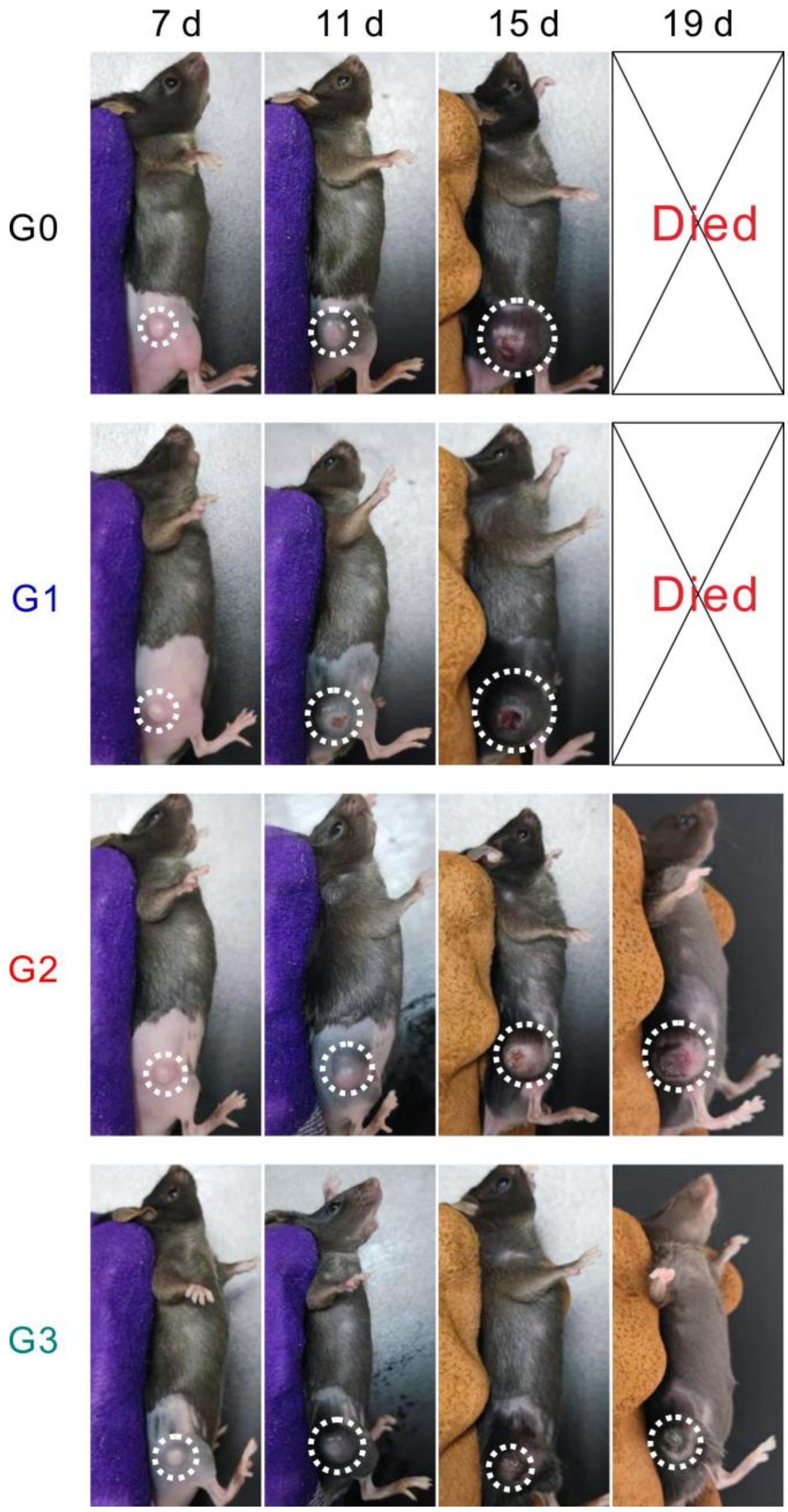

Supplementary Fig. 17 Representative photographs of mice at different time points after different treatments. The dotted circle represents the tumor site. The term "Died" indicates that mice were humanely euthanized and recorded as deceased when tumor volume exceeded 1500 mm^3, in accordance with animal research ethical guidelines requiring the establishment of humane endpoints. G0: Blank; G1: GB1275; G2: VNP20009; G3: GB1275 + VNP20009.

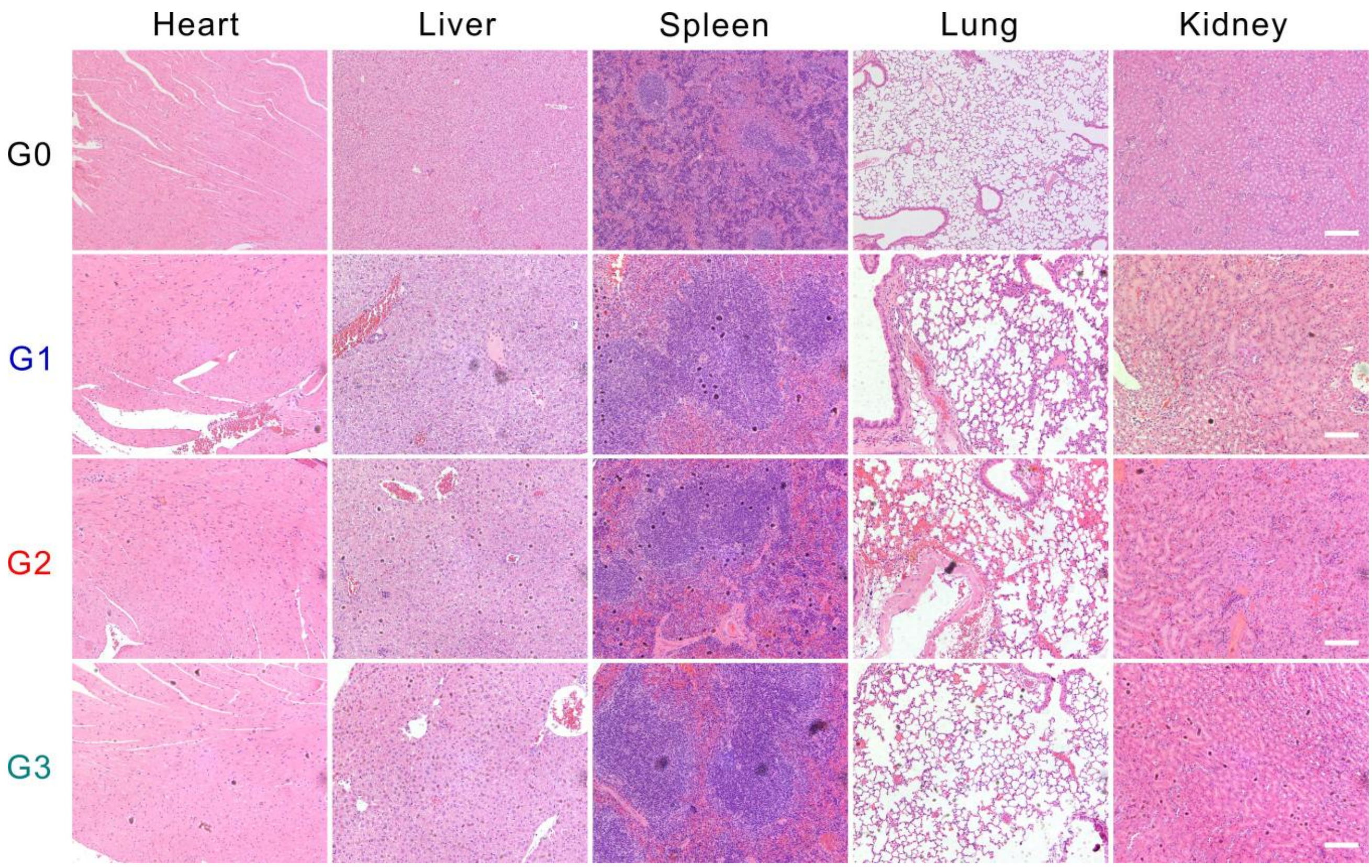

Supplementary Fig. 18 Representative photographs of H&E staining of major organs after different treatments. G0: Blank; G1: GB1275; G2: VNP20009; G3: GB1275 + VNP20009. Scale bar: 200 μm.

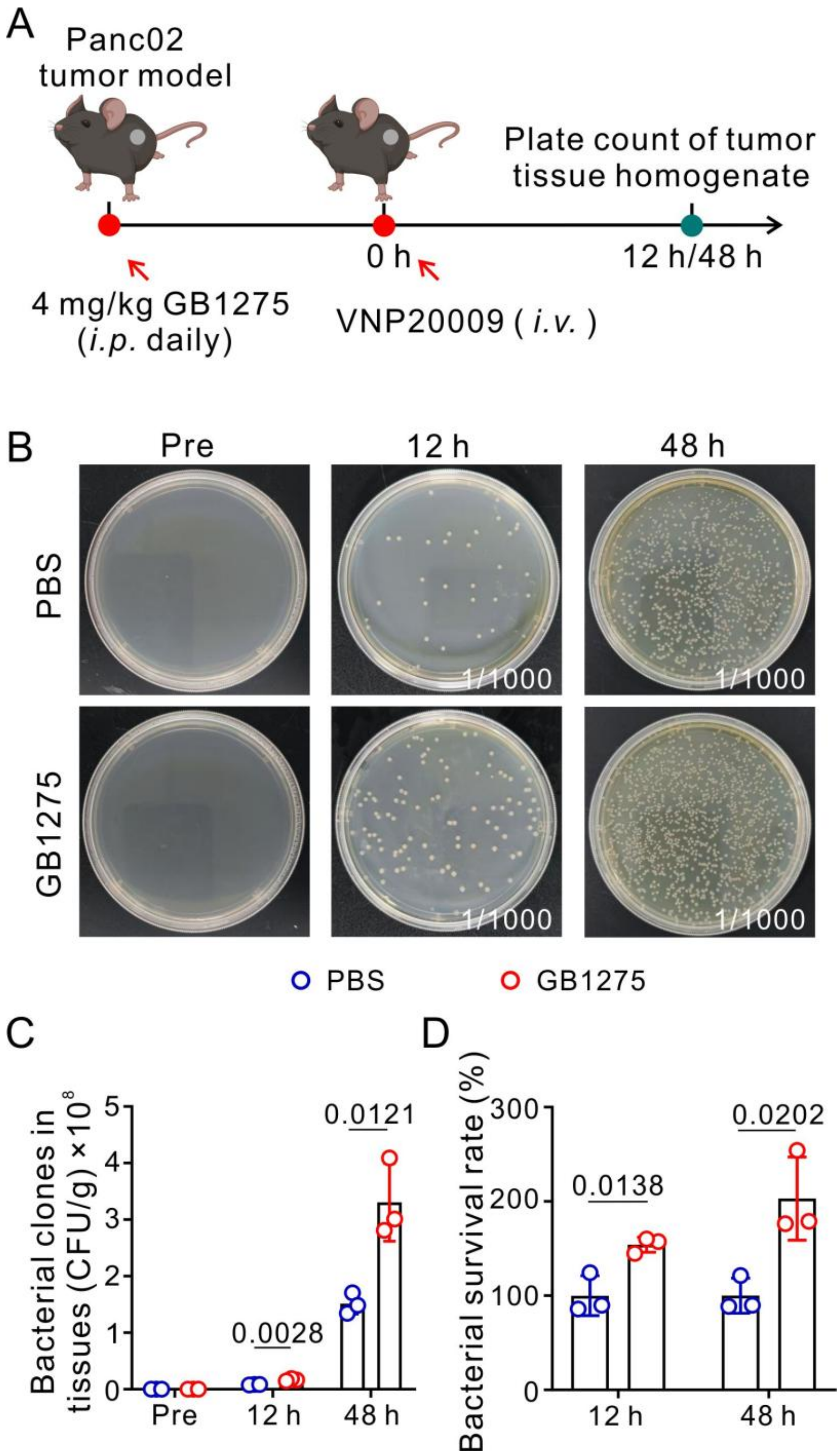

Supplementary Fig. 19 **A** Schematic illustration of the in vivo administration regimen of GB1275 and VNP20009 in Panc02 subcutaneous tumor model, with an equal volume of PBS as the control. *i.p.*, daily intraperitoneal injection. *i.v.*, intravenous injection. **B**, **C** Representative photographs and corresponding quantification of bacterial colonization in tumors obtained from Panc02 tumor bearing mice at different time points after different treatments, $n = 3$ independent samples. **D** Bacterial survival

rates in tumor tissues of different groups at different time points, $n = 3$ independent samples. The data are expressed as mean ± s.d. All replicates are biological, and the P values were determined using two-tailed Student's t test. Source data are provided as a Source Data file. Figure A was created in BioRender. Zhang, B. (2026) https://BioRender.com/79b3hb8.

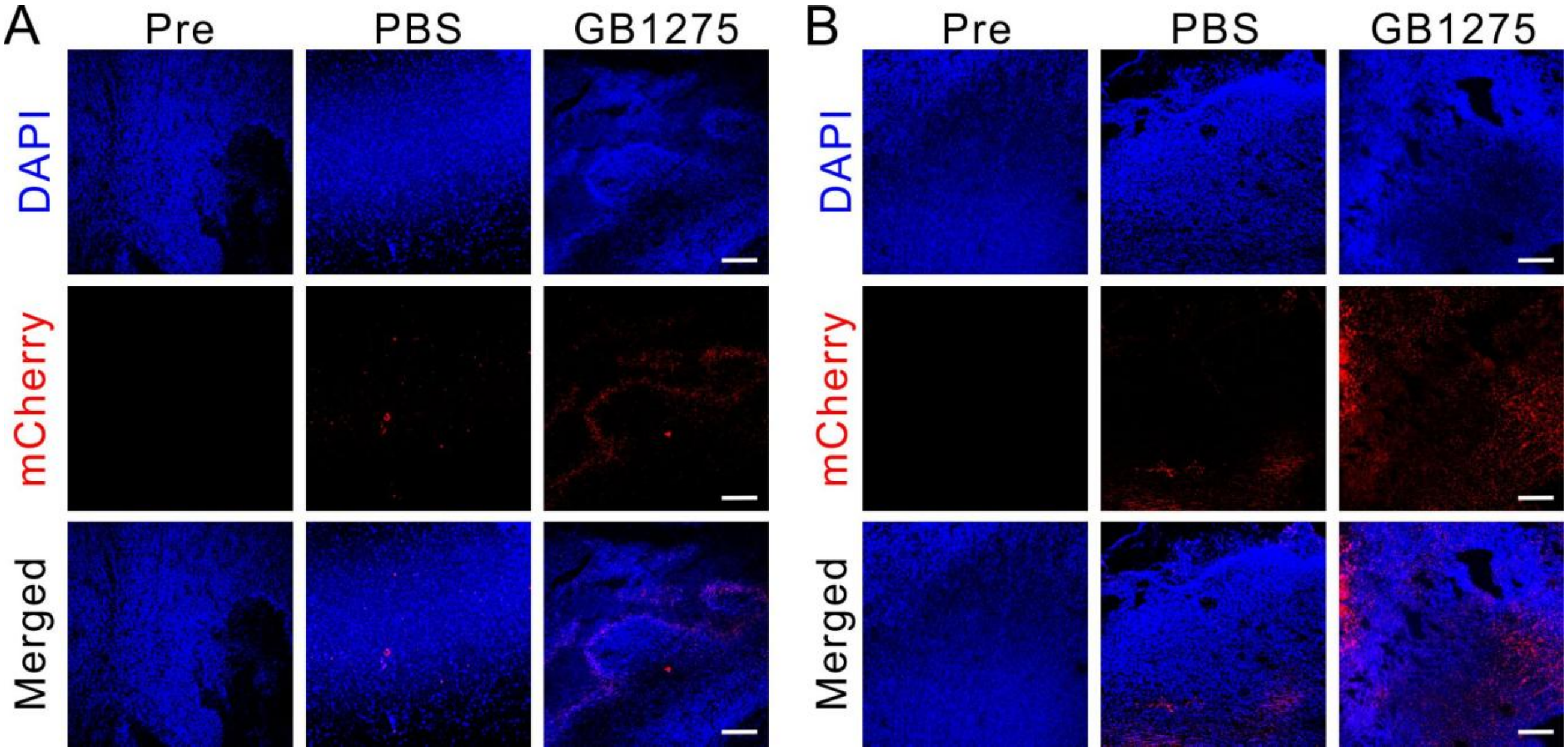

Supplementary Fig. 20 Representative mCherry fluorescence images of VNP20009 in tumor tissues after different treatments at (**A**) 12 h and (**B**) 48 h. Scale bar: 200 μm.

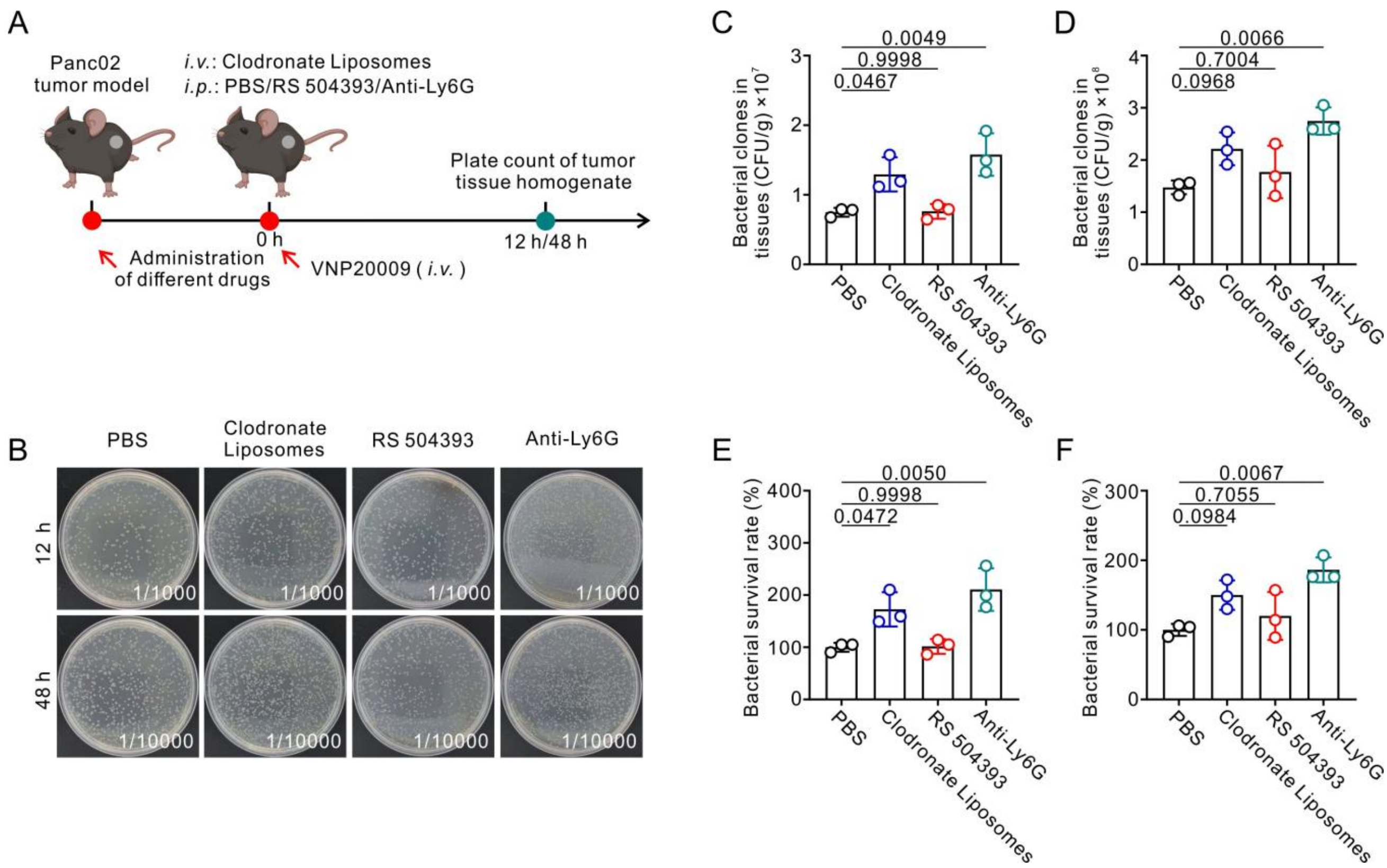

Supplementary Fig. 21 **A** Schematic illustration of the in vivo administration regimen of different drugs and VNP20009 in Panc02 subcutaneous tumor model. *i.p.*, intraperitoneal injection. *i.v.*, intravenous injection. **B** Representative photographs and corresponding quantification of bacterial colonization in tumors obtained from Panc02 tumor-bearing mice at different time points (**C**: 12 h and **D**: 48 h) after different treatments, $n = 3$ independent samples. Bacterial survival rates in tumor tissues of different groups at (**E**) 12 h and (**F**) 48 h, $n = 3$ independent samples. The data are expressed as mean ± s.d. All replicates are biological, and the P values were determined using one-way ANOVA followed by Tukey's multiple comparisons tests. Source data are provided as a Source Data file. Figure A was created in BioRender. Zhang, B. (2026) https://BioRender.com/79b3hb8.

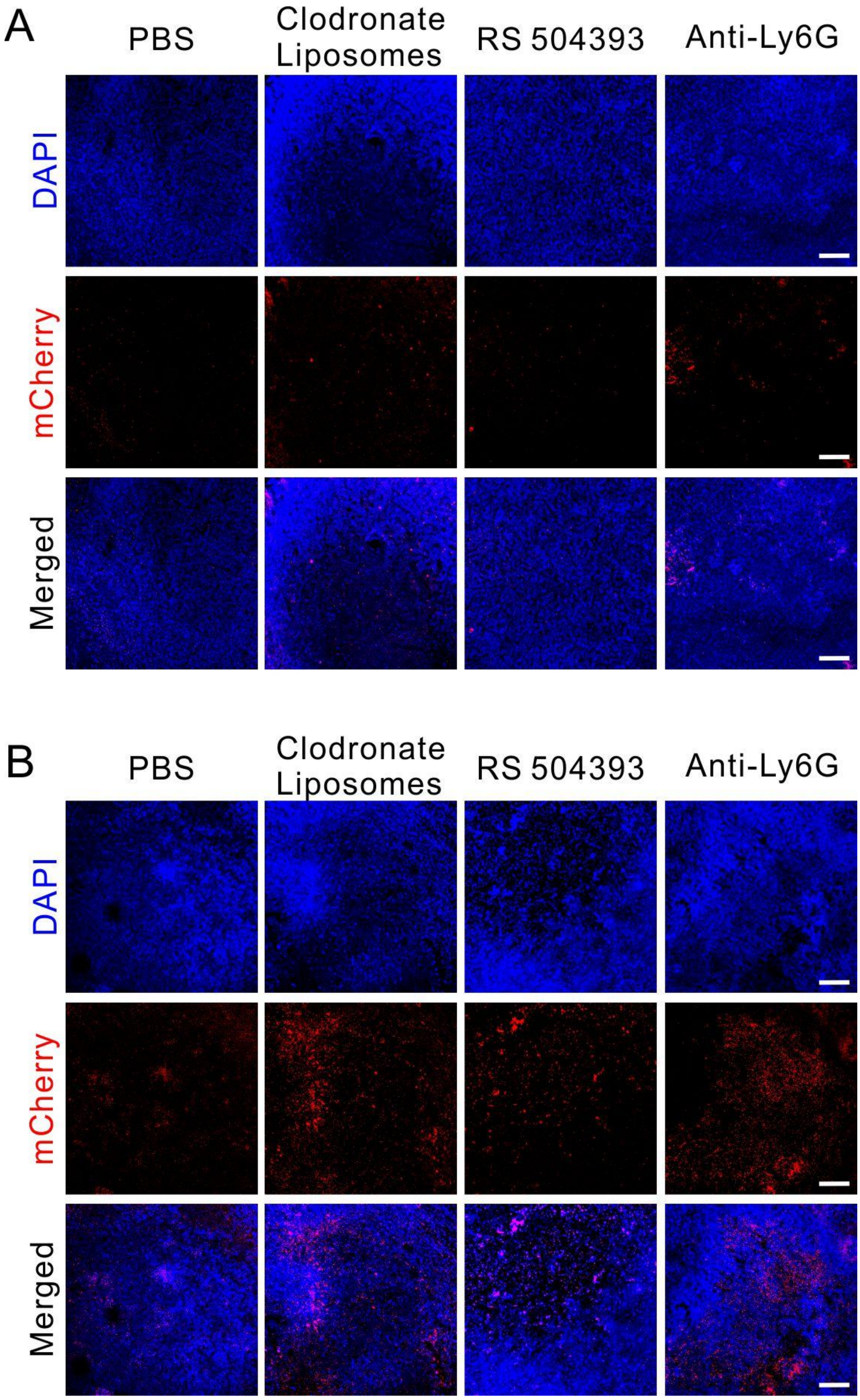

Supplementary Fig. 22 Representative mCherry fluorescence images of VNP20009 in tumor tissues after different treatments at (**G**) 12 h and (**H**) 48 h. Scale bar: 200 μm.

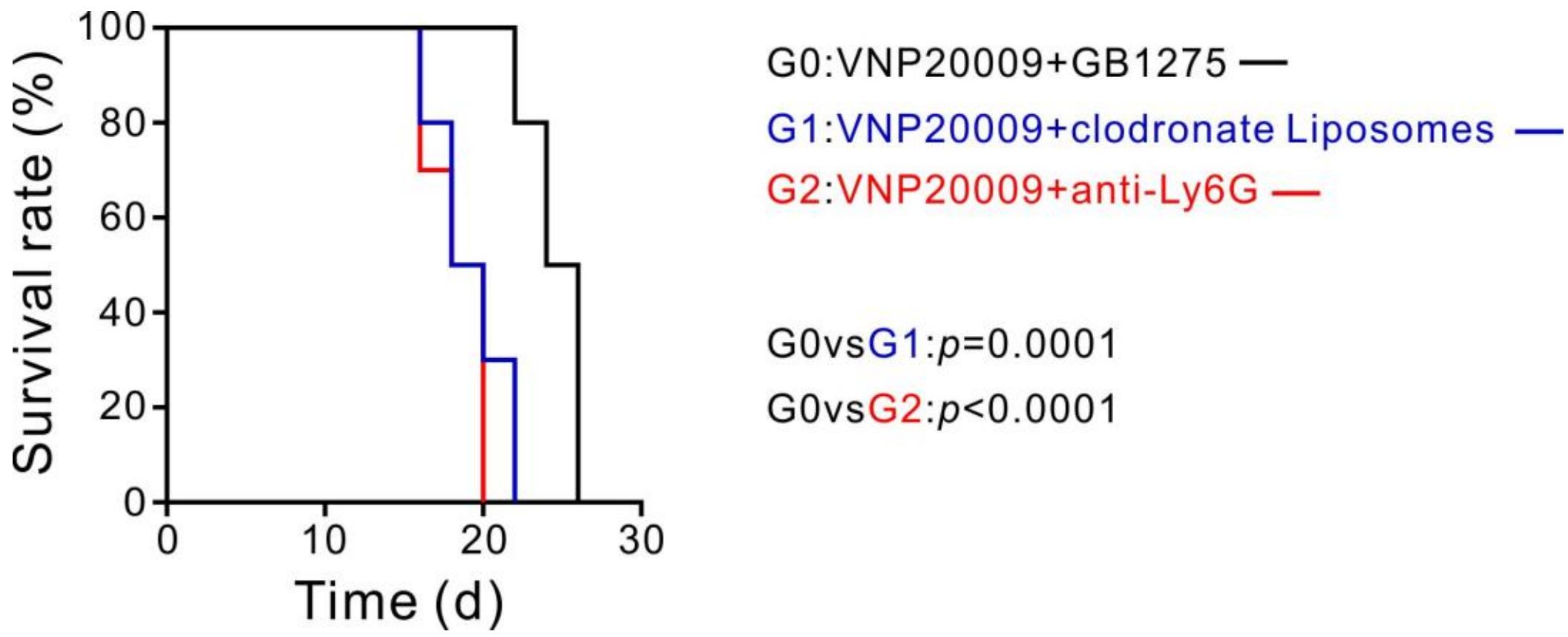

Supplementary Fig. 23 Monitoring of survival time of mice after different treatments. vs is the abbreviation for versus, used for comparison between two groups. n = 10 independent samples. Source data are provided as a Source Data file.

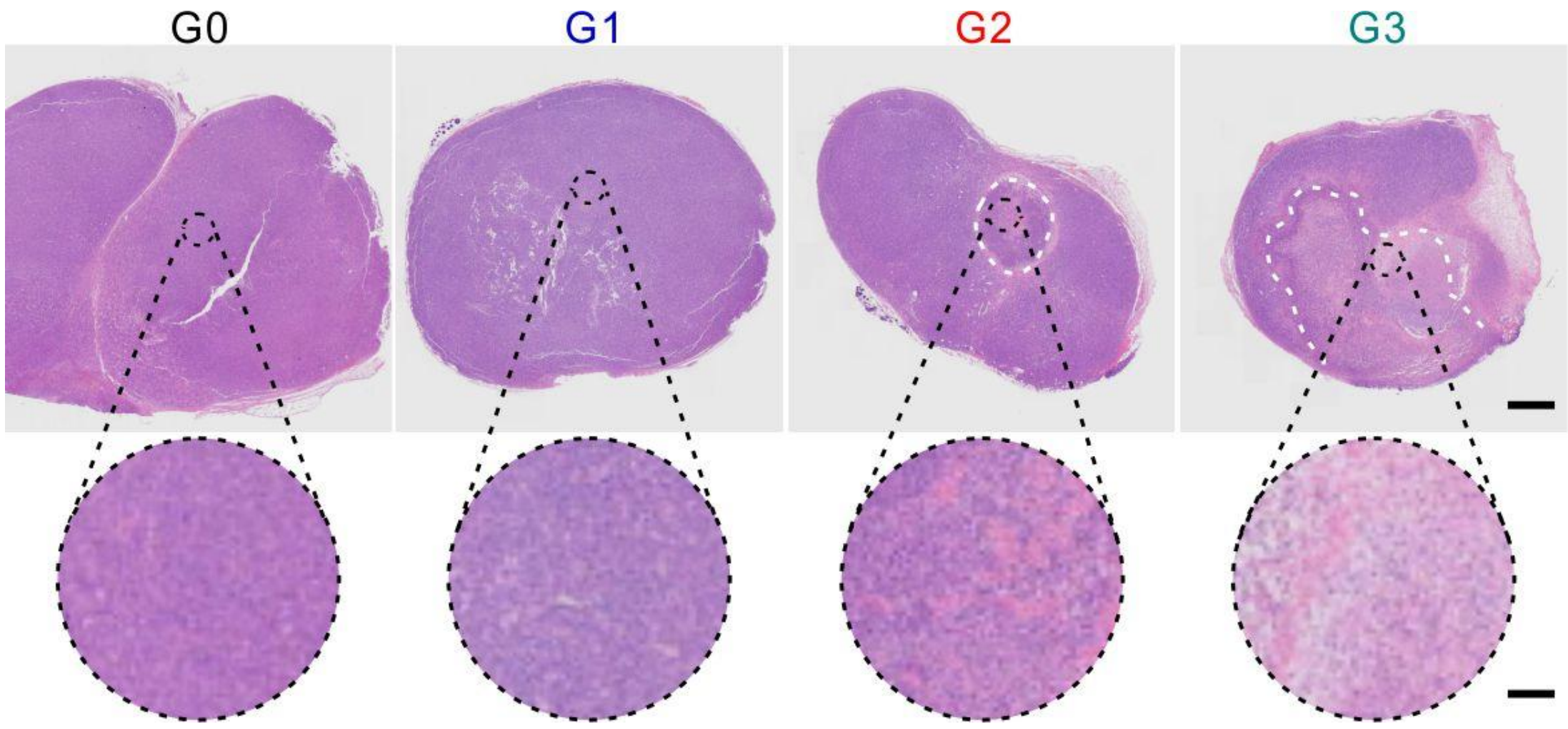

Supplementary Fig. 24 Representative images of H&E staining of the largest cross section of Panc02 tumor tissue obtained at day 14 after different treatments. G0: Blank; G1: GB1275; G2: VNP20009; G3: GB1275 + VNP20009. Scale bar: 1 mm and 100 µm (enlarged image).

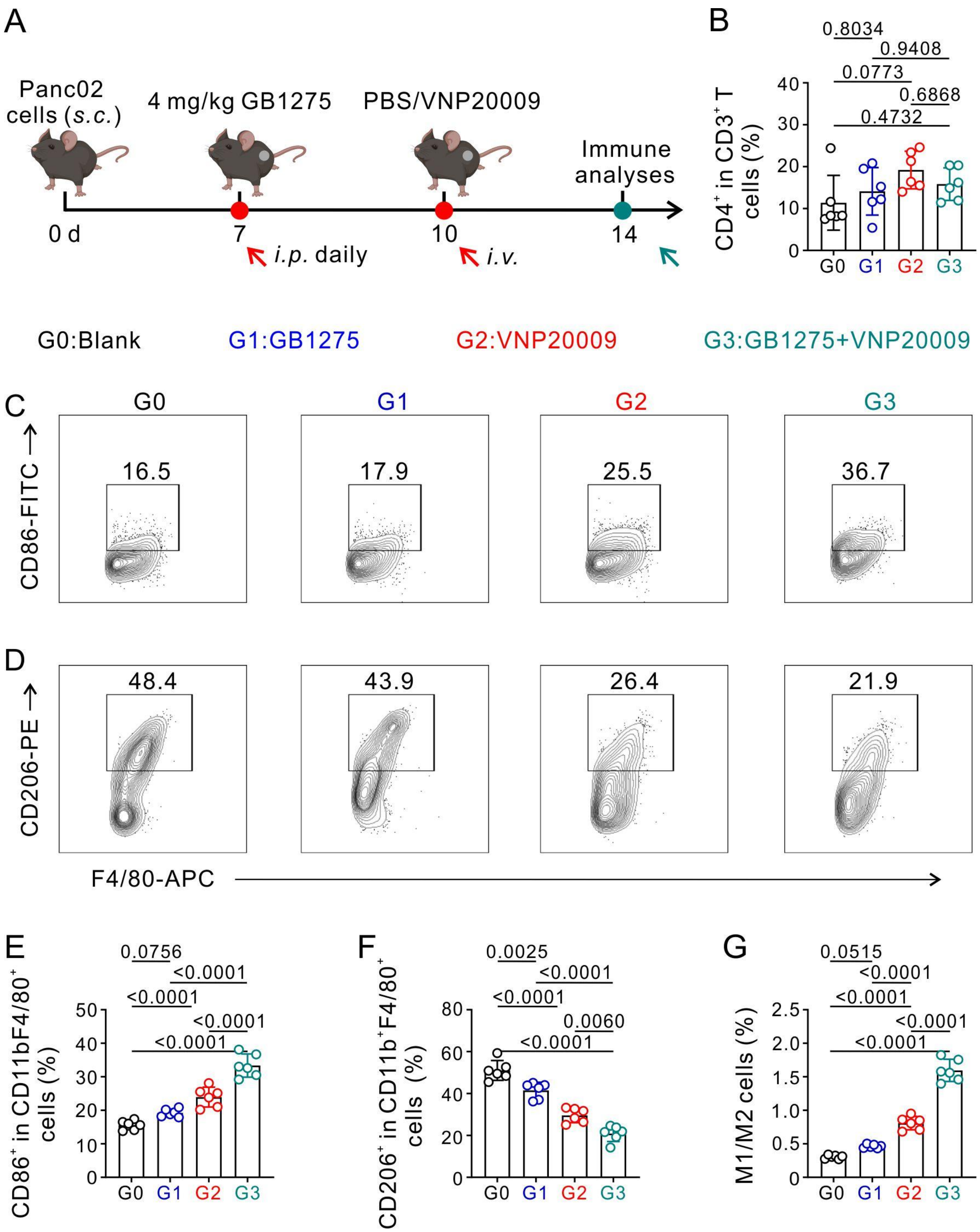

Supplementary Fig. 25 **A** Schematic illustration of the experimental protocols for immune cell infiltration in tumor tissues after different treatments. *s.c.*, subcutaneous injection. *i.p.* daily, daily intraperitoneal injection. *i.v.,* intravenous injection. **B** Corresponding statistic of $CD4^{+}$ T cells

infiltration in Panc02 tumor tissues after different treatments (corresponding to Fig. 4I), n = 6 independent samples. Flow cytometry analysis and corresponding statistics of the proportion of (**C, E**) M1 and (**D, F**) M2 macrophages in tumor tissue after different treatments, n = 6 independent samples. **G** The corresponding statistics of the M1/M2 cells ratio in Panc02 tumor tissues after different treatments n = 6 independent samples. The data are expressed as mean ± s.d. All replicates are biological, and the P values were determined using one-way ANOVA followed by Tukey's multiple comparisons tests. Source data are provided as a Source Data file. Figure A was was created in BioRender. Zhang, B. (2026) https://BioRender.com/79b3hb8.

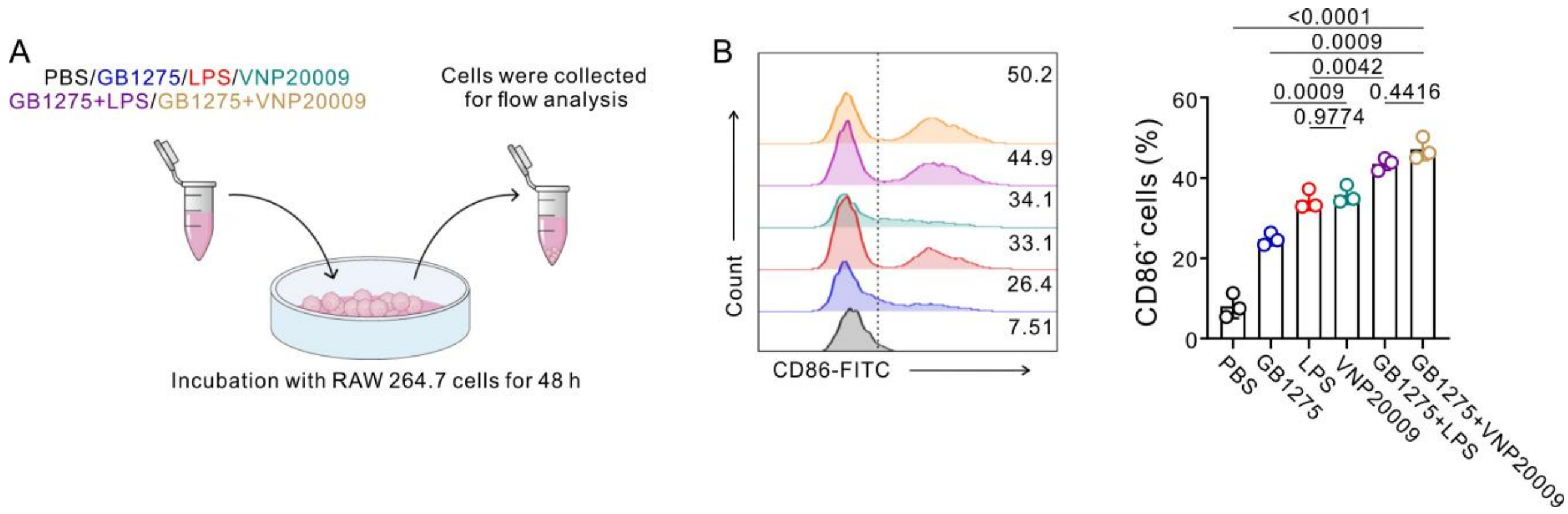

Supplementary Fig. 26 **A** Schematic illustration of the experimental protocols for macrophage polarization in vitro. **B** Flow cytometry analysis and corresponding statistics of $CD86^+$ expression levels after different treatments, *n* = 3 independent samples. The data are expressed as mean ± s.d. All replicates are biological, and the *P* values were determined using one-way ANOVA followed by Tukey's multiple comparisons. Source data are provided as a Source Data file.

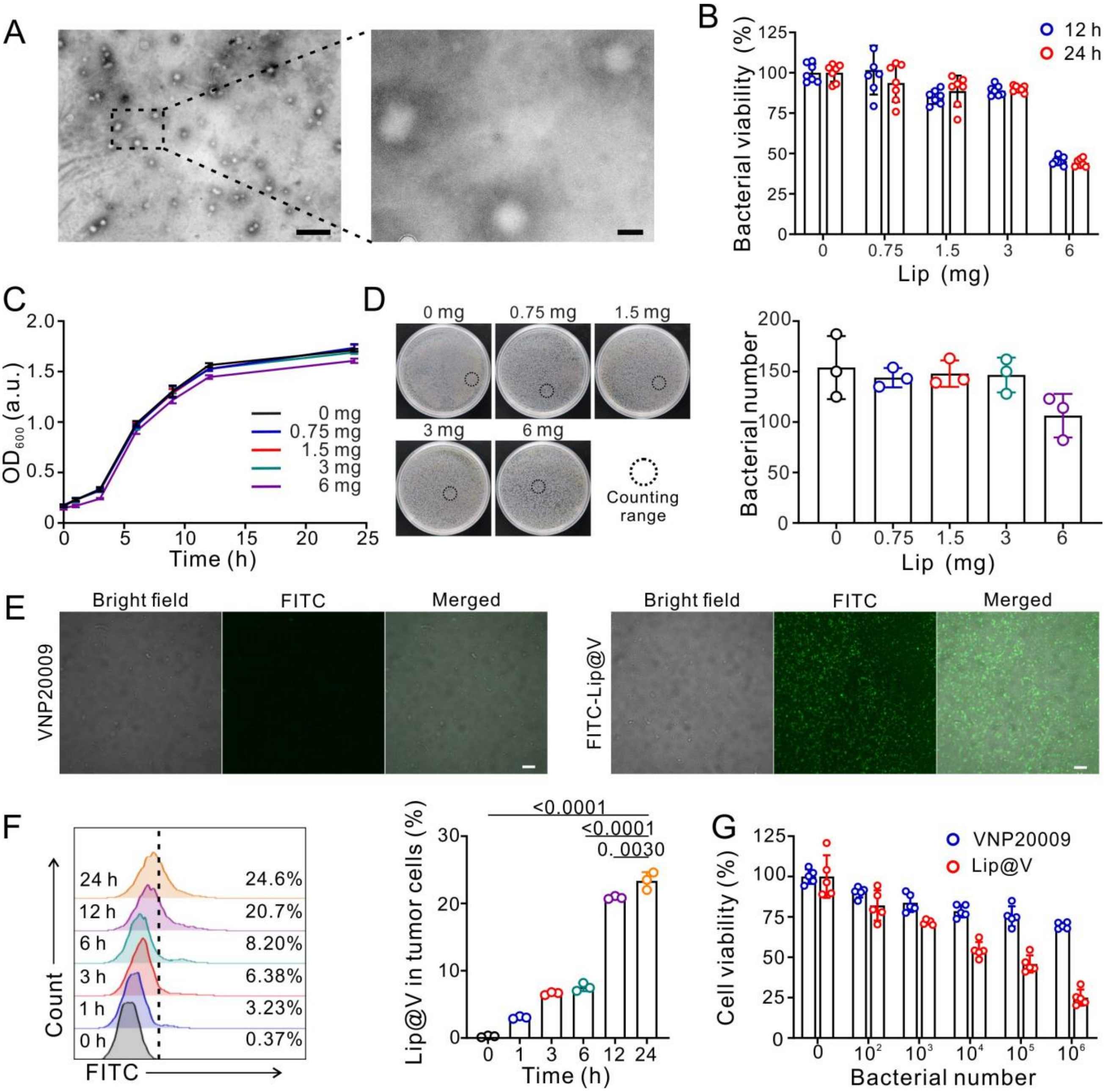

Supplementary Fig. 27 **A** TEM images of phosphotungstic acid-stained drug-loaded liposomes (Lip), Scale bars: 1 μm and 200 nm (enlarged image). **B** Cytotoxic effects of Lip on VNP20009 at indicated concentrations and time points, assessed by the CCK-8 assay, *n* = 7 independent samples. **C** The growth curves of VNP20009 after incubation with different concentrations of Lip, *n* = 10 independent samples. **D** Representative photographs of bacterial cultures and corresponding statistics after incubation of bacteria with different concentrations of Lip for 12 h, *n* = 3 independent samples. **E**

Representative confocal fluorescence imaging of VNP20009 and FITC-Lip@V. Green fluorescence signals originate from FITC. Scale bars: 10 μm. **F** Flow cytometry and corresponding statistical analysis of tumor cells infiltrated by FITC-Lip@V at various time points, *n* = 3 independent samples. **G** Cytotoxicity of different concentrations of VNP20009 and Lip@V on Panc02 cells after incubation, assessed by the CCK-8 assay, *n* = 5 independent samples. The data are expressed as mean ± s.d. All replicates are biological, and the *P* values in F were determined using one-way ANOVA followed by Tukey's multiple comparisons. Source data are provided as a Source Data file.

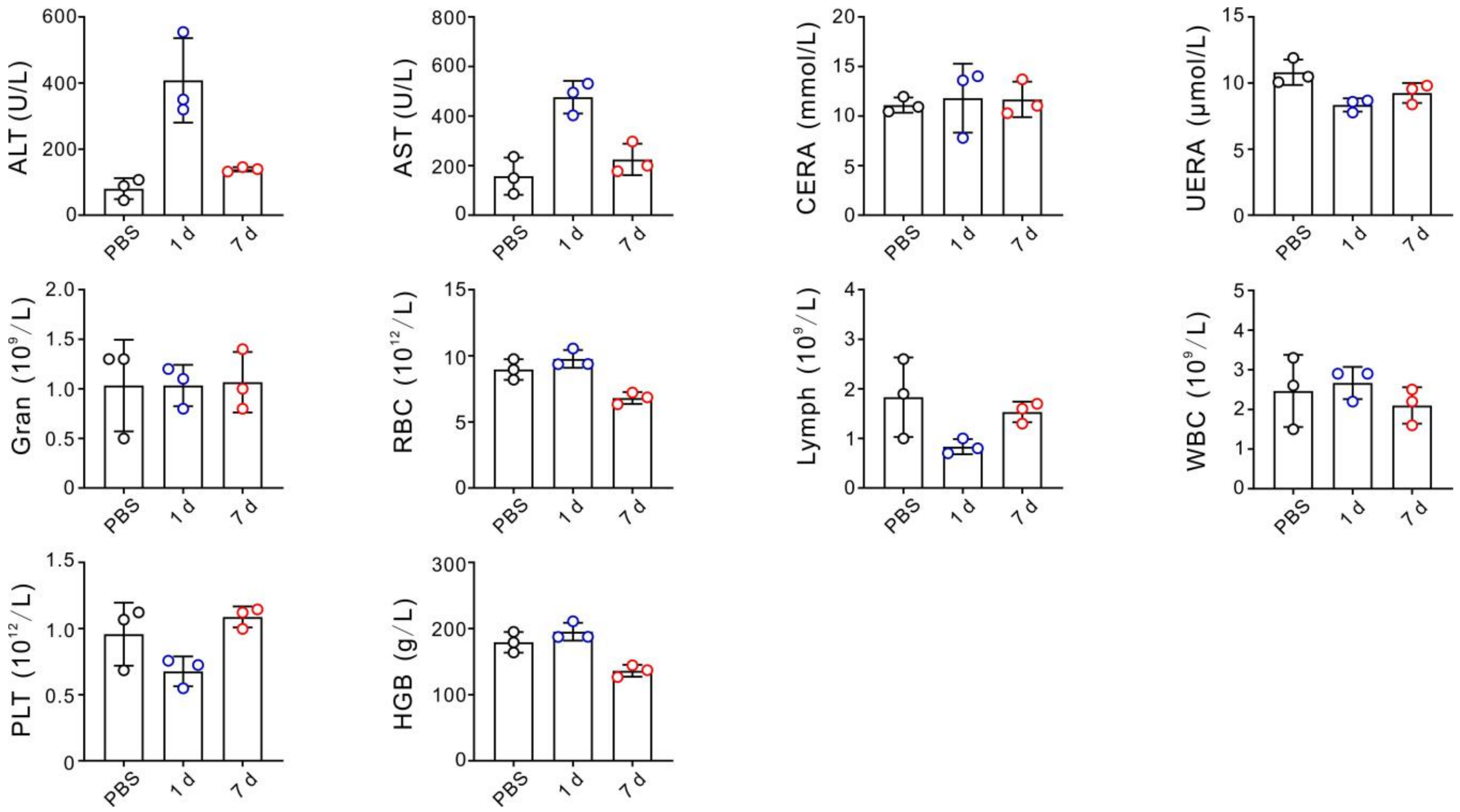

Supplementary Fig. 28 Blood routine analysis of mice at different time points after Lip@V administration. 5×10^6 CFU of Lip@V was administered intravenously via the tail vein, with an equal volume of PBS as the control, *n* = 3 independent samples. Source data are provided as a Source Data file.

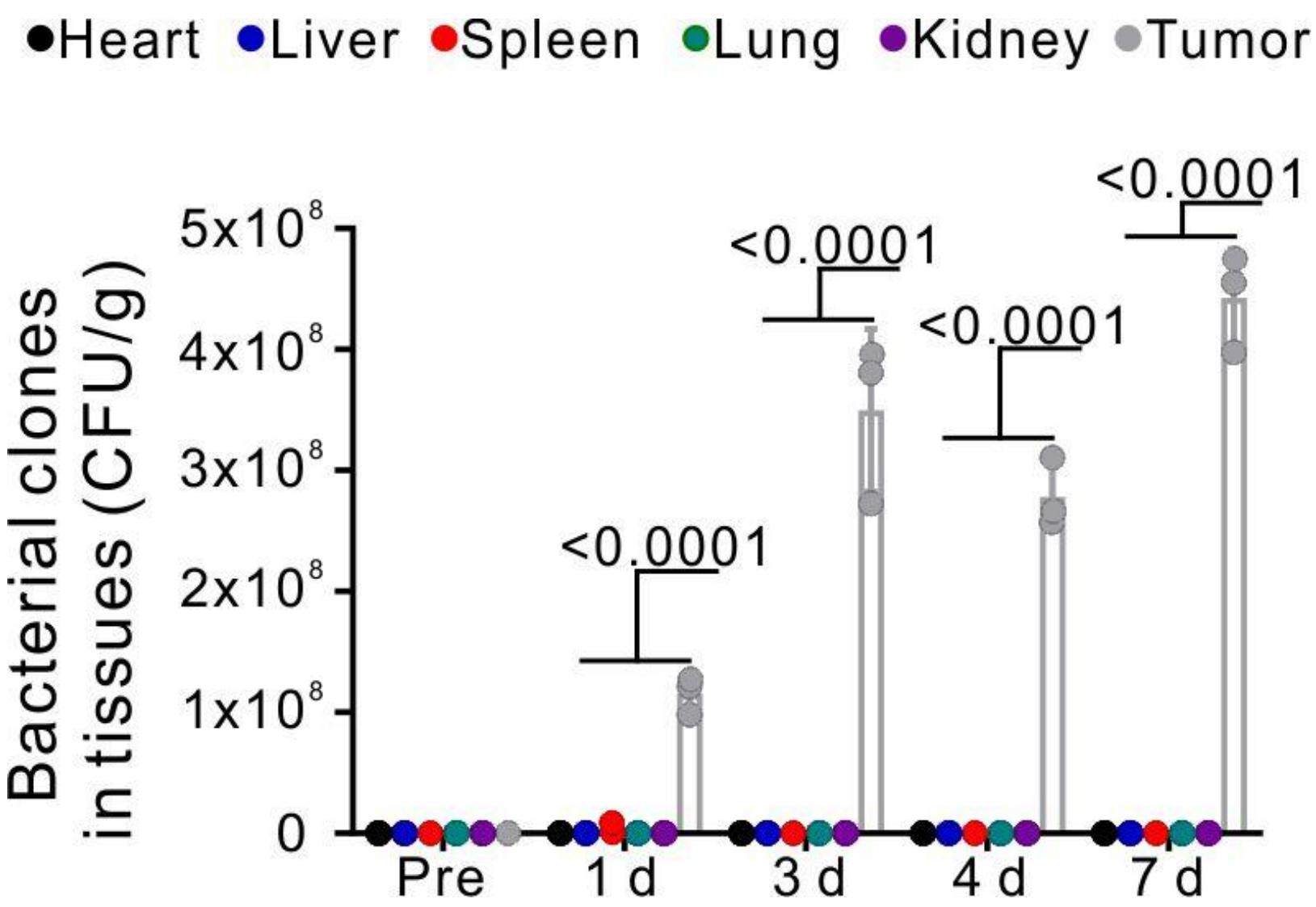

Supplementary Fig. 29 Quantification of bacterial colonization in major organs and tumors obtained from Panc02 tumor-bearing mice at different time points after intravenous administration of Lip@V, *n* = 3 independent samples. The data are expressed as mean ± s.d. All replicates are biological, and the *P* values were determined using one-way ANOVA followed by Tukey's multiple comparisons. Source data are provided as a Source Data file.

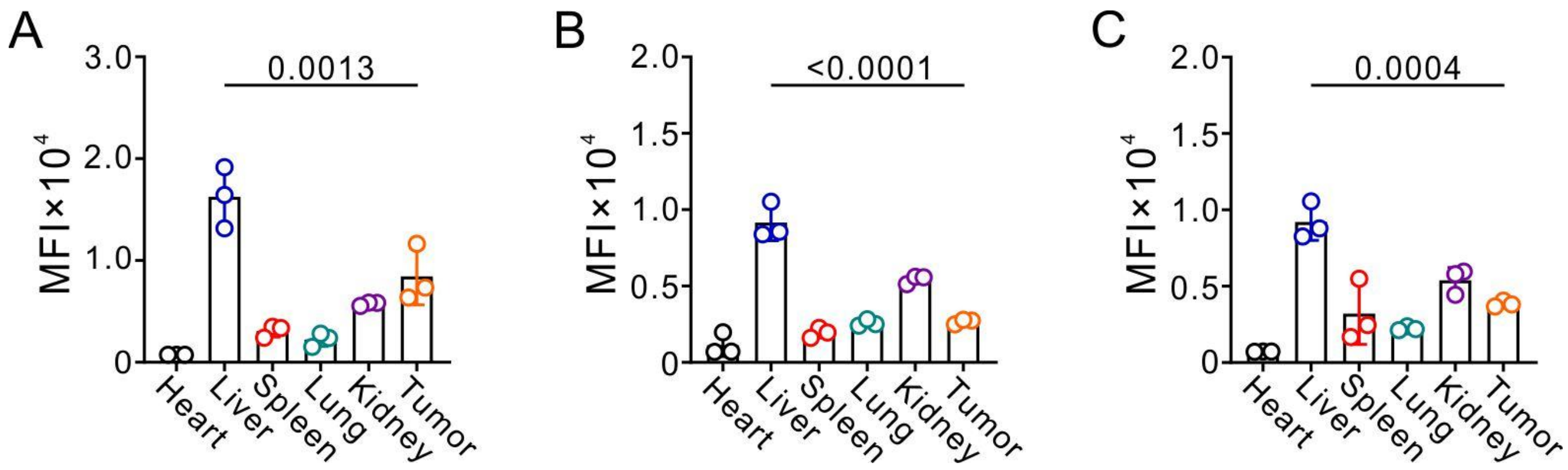

Supplementary Fig. 30 **A-C** Corresponding fluorescence statistics of major organs and tumors at 24 h after intravenous injection of Cy-5.5-Lip@V, Cy-5.5-Lip, and Cy-5.5-Lip@V (dead), n = 3 independent samples. The data are expressed as mean ± s.d. All replicates are biological, and the P values were determined using one-way ANOVA followed by Tukey's multiple comparisons test. Source data are provided as a Source Data file.

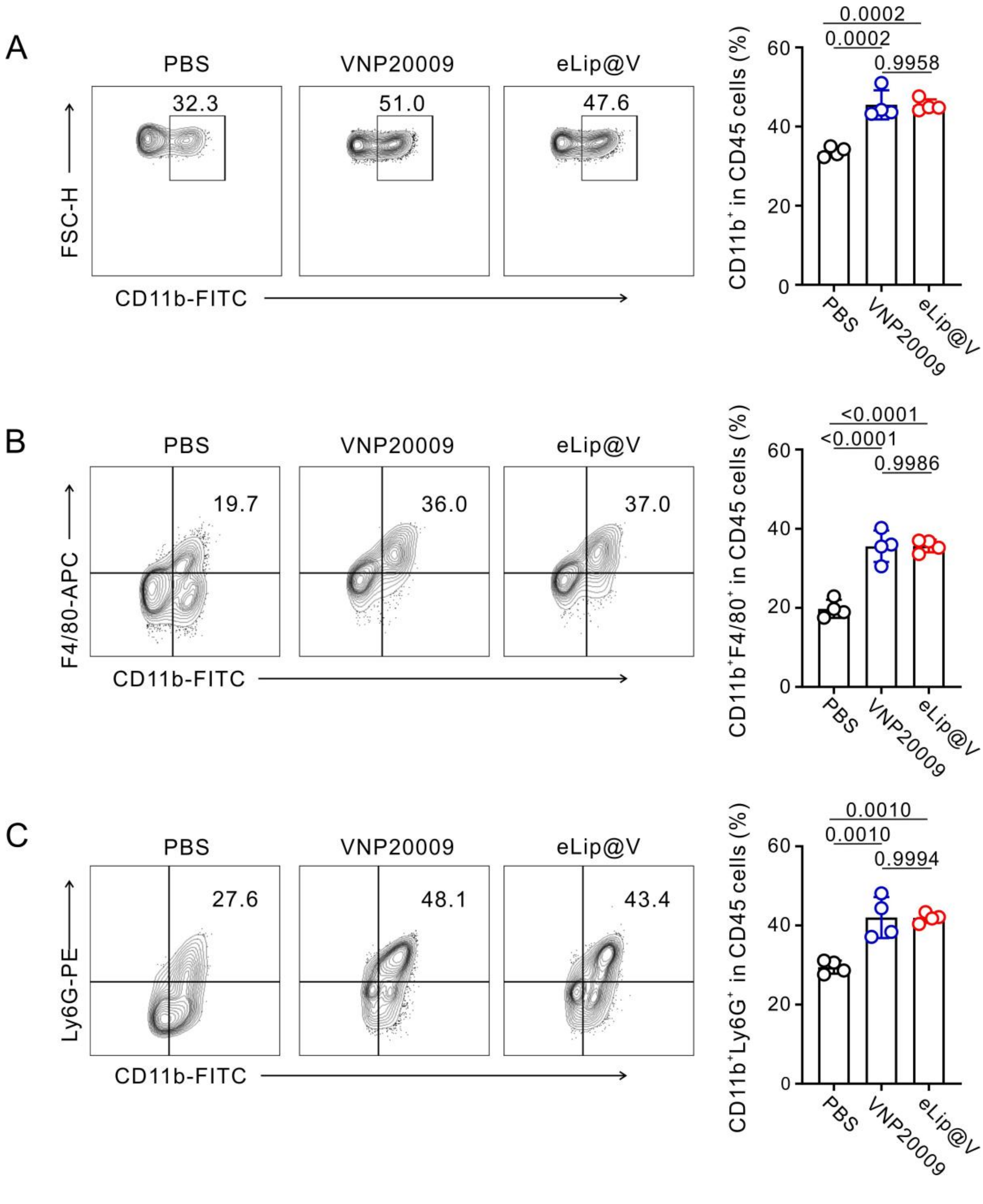

Supplementary Fig. 31 **A-C** Flow cytometry analysis and corresponding statistics of CD11b⁺ cells, macrophages, and neutrophils infiltration in tumor tissues after different treatments, $n = 4$ independent samples. eLip represents empty liposomes. The data are expressed as mean ± s.d. All replicates are biological, and the *P* values were determined using one-way ANOVA followed by Tukey's multiple comparisons. Source data are provided as a Source Data file.

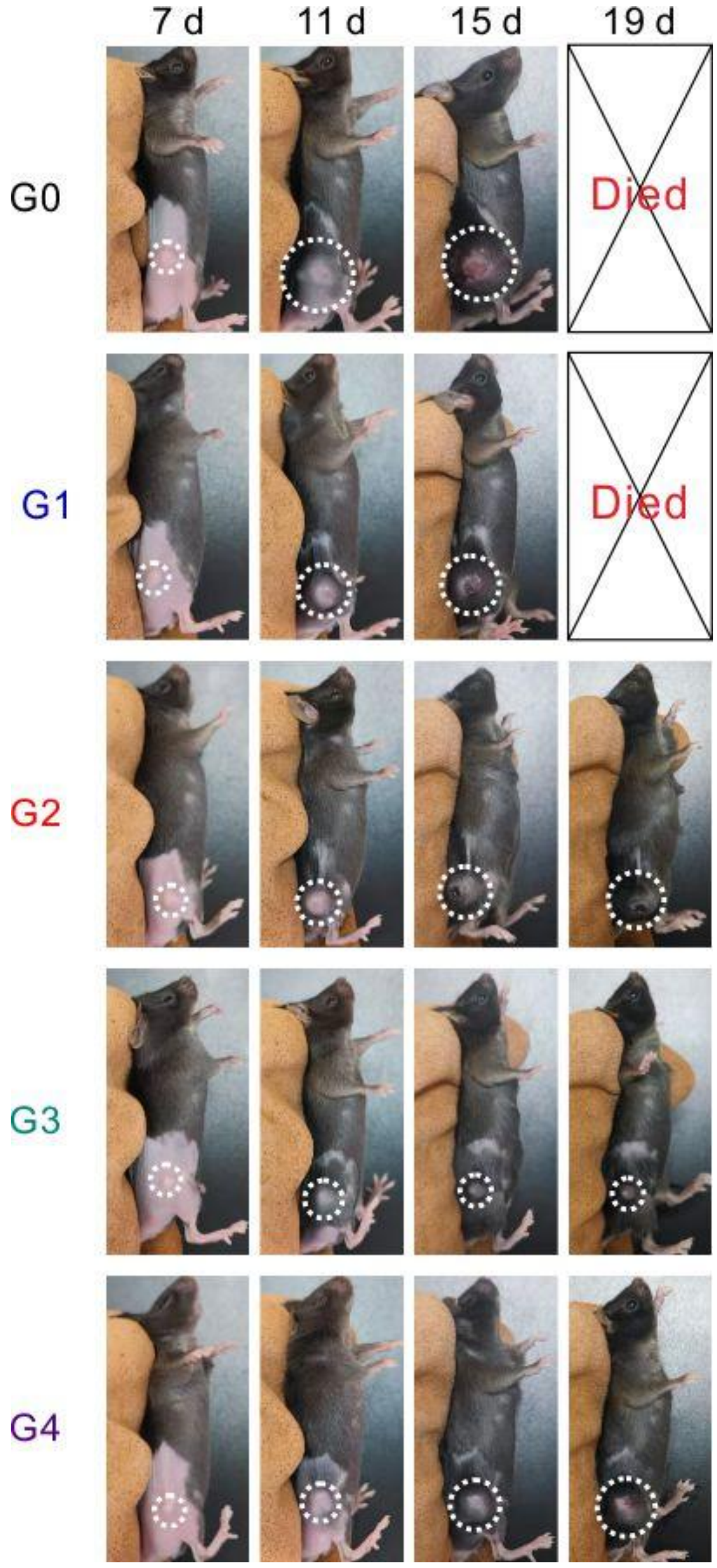

Supplementary Fig. 32 Representative photographs of mice at different time points after different treatments. The dotted circle represents the tumor site. The term "Died" indicates that mice were humanely euthanized and recorded as deceased when tumor volume exceeded 1500 mm^3, in accordance with animal research ethical guidelines requiring the establishment of humane endpoints. G0: Blank; G1: GB1275; G2: Lip@V; G3: GB1275 + Lip@V; G4: GB1275 + Lip.

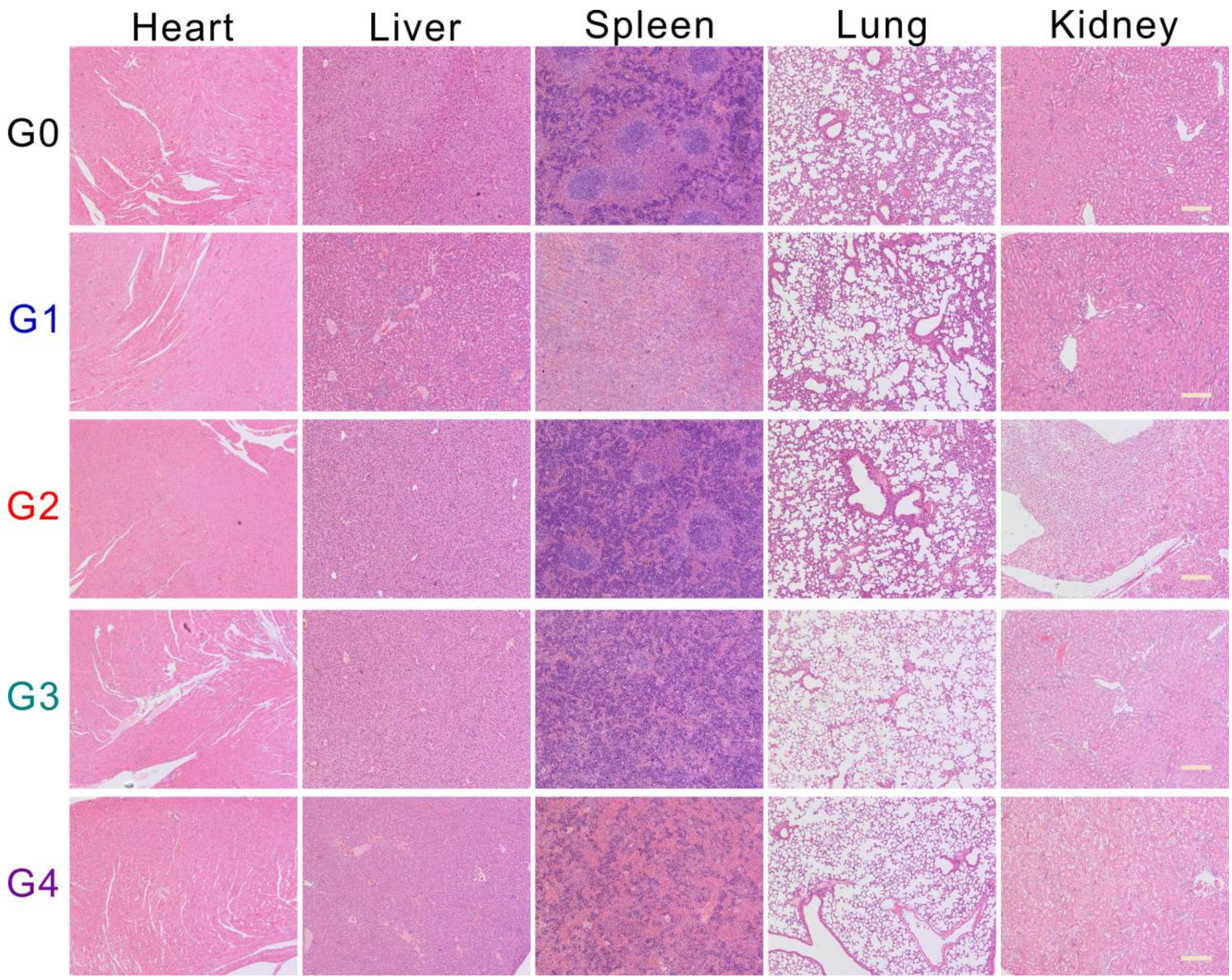

Supplementary Fig. 33 Representative photographs of H&E staining of major organs after different treatments. G0: Blank; G1: GB1275; G2: Lip@V; G3: GB1275 + Lip@V; G4: GB1275 + Lip. Scale bar: 200 μm.

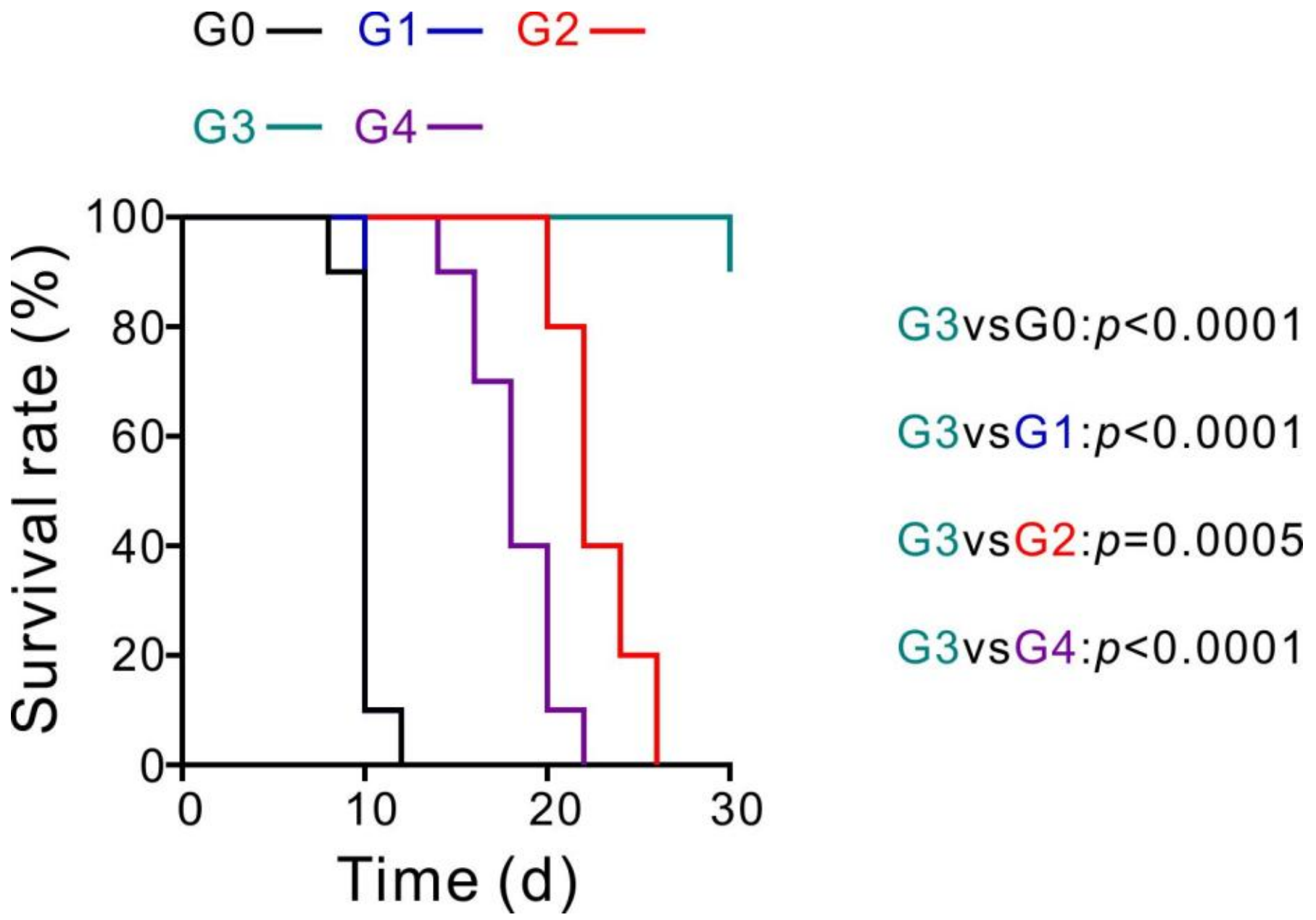

Supplementary Fig. 34 Survival curves of mice after different treatments, *n* = 10 independent samples. vs is the abbreviation for versus, used for comparison between two groups. G0: Blank; G1: GB1275; G2: Lip@V; G3: GB1275 + Lip@V; G4: GB1275 + Lip. Source data are provided as a Source Data file.

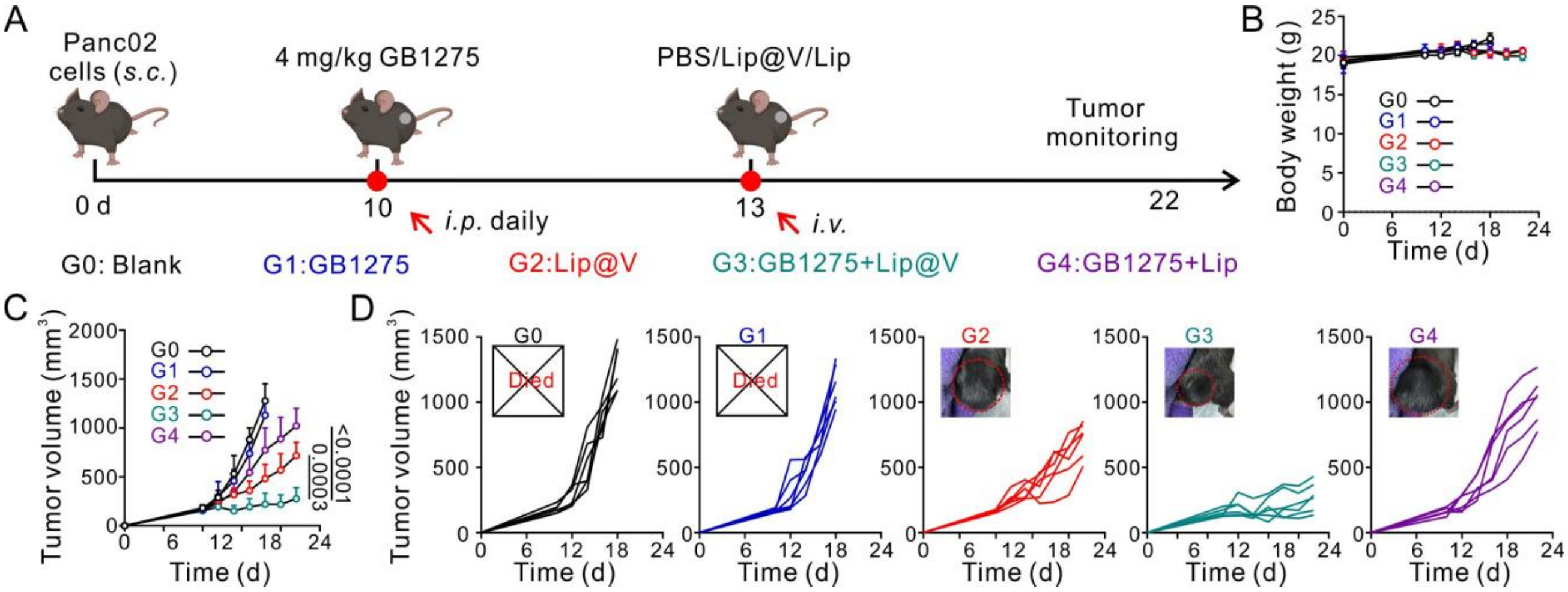

Supplementary Fig. 35 **A** Schematic illustration of the experimental protocols for GB1275-enhanced bacterial anti-tumor therapy for advanced tumors. *s.c.*, subcutaneous injection. *i.p.* daily, daily intraperitoneal injection. *i.v.,* intravenous injection. **B** Changes in mouse body weights of mice after different treatments. *n* = 6 independent samples. **C, D** Tumor growth curves and individual tumor growth curves after different treatments. In supplementary Fig. 36D, the tumor images in the bar chart are representative images of each group of mice after the end of treatment. The term "Died" indicates that mice were humanely euthanized and recorded as deceased when tumor volume exceeded 1500 mm^3, in accordance with animal research ethical guidelines requiring the establishment of humane endpoints, *n* = 6 independent samples. The data are expressed as mean ± s.d. All replicates are biological, and the *P* values in C were determined using two-way ANOVA followed by Tukey's multiple comparisons. Source data are provided as a Source Data file. Figure A was created in BioRender. Zhang, B. (2026) https://BioRender.com/79b3hb8.

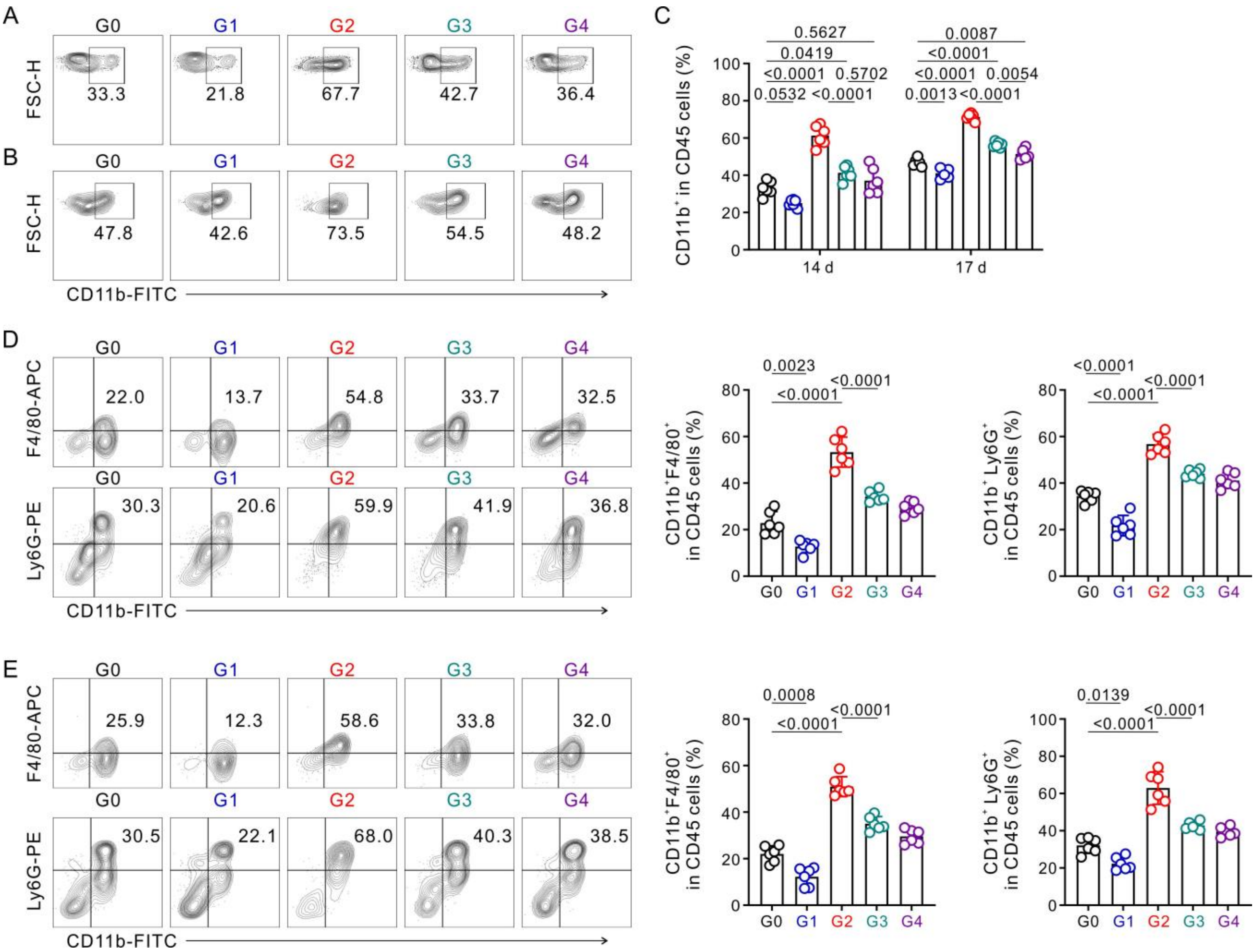

Supplementary Fig. 36 **A-C** Flow cytometry analysis and corresponding statistics of CD11b⁺ cells infiltration in tumor tissues after different treatments on day 14 and day 17. Flow cytometry analysis and corresponding statistics of macrophages and neutrophils cells infiltration in tumor tissues after different treatments on (**D**) day 14 and (**E**) day 17, *n* = 6 independent samples. G0: Blank; G1: GB1275; G2: Lip@V; G3: GB1275 + Lip@V; G4: GB1275 + Lip. The data are expressed as mean ± s.d. All replicates are biological, and the *P* values were determined using one-way ANOVA followed by Tukey's multiple comparisons. Source data are provided as a Source Data file.

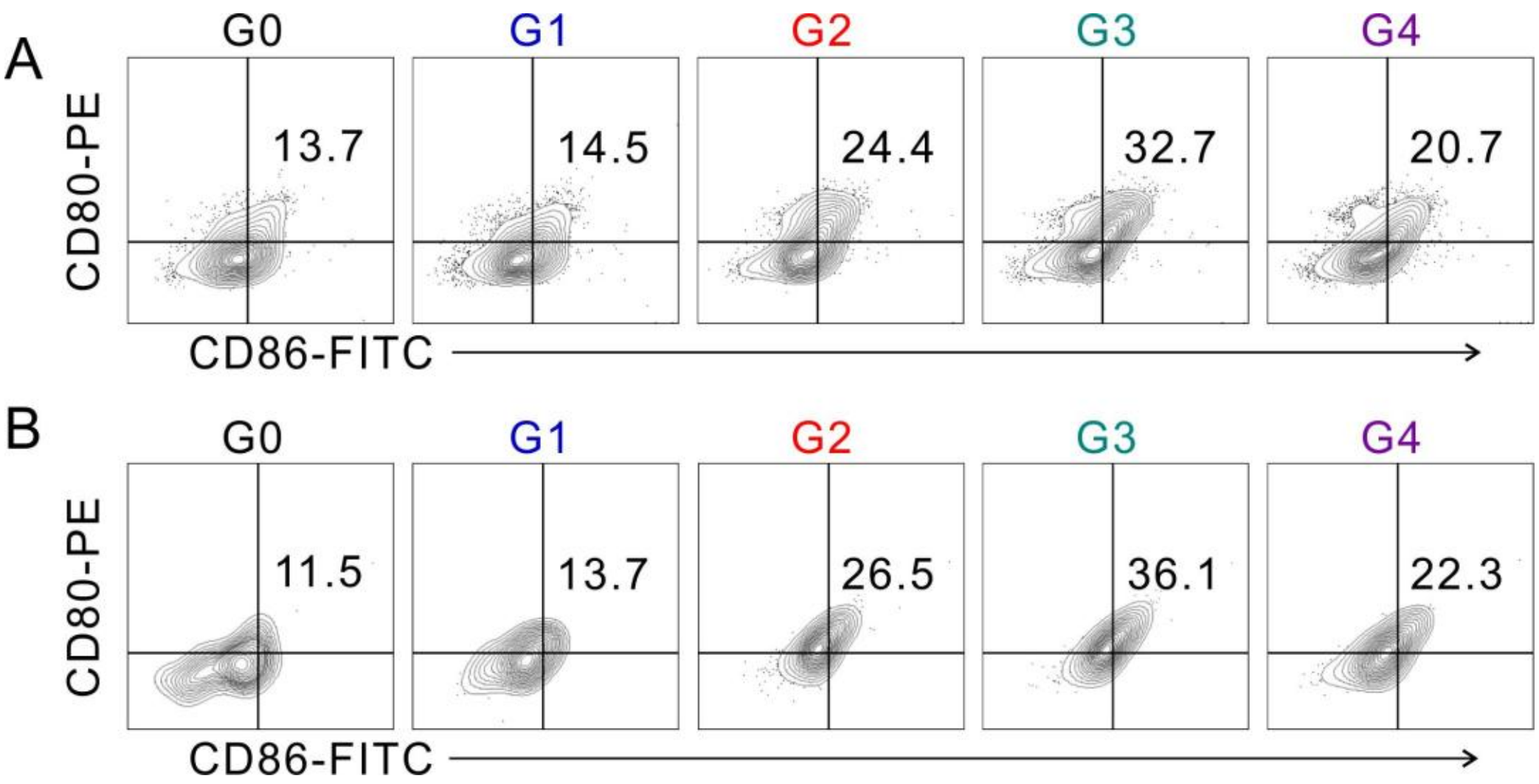

Supplementary Fig. 37 Flow cytometry analysis of DCs infiltration in tumor tissues after different treatments on (**A**) day 14 and (**B**) day 17. G0: Blank; G1: GB1275; G2: Lip@V; G3: GB1275 + Lip@V; G4: GB1275 + Lip.

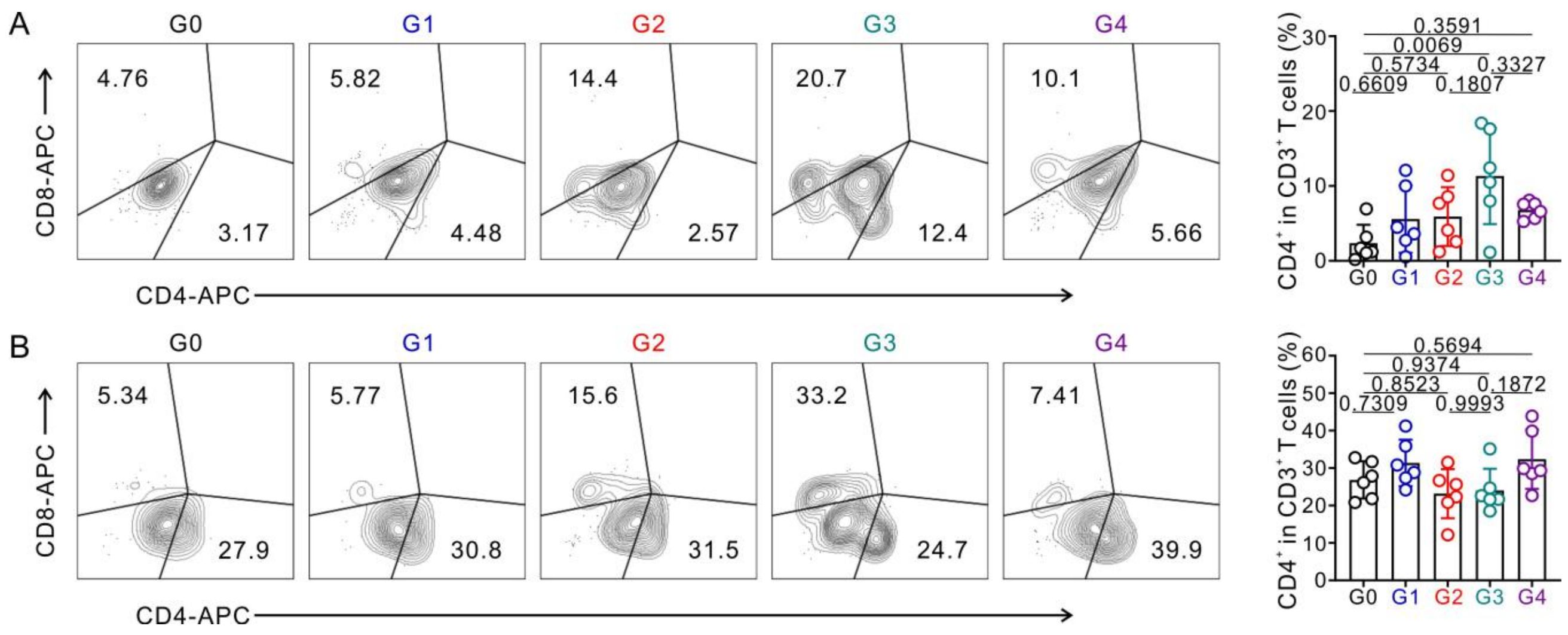

Supplementary Fig. 38 Flow cytometry analysis and corresponding statistics of CD4⁺ T cells infiltration in tumor tissues after different treatments on (**A**) day 14 and (**B**) day 17, $n = 6$ independent samples. G0: Blank; G1: GB1275; G2: Lip@V; G3: GB1275 + Lip@V; G4: GB1275 + Lip. The data are expressed as mean ± s.d. All replicates are biological, and the P values were determined using one-way ANOVA followed by Tukey's multiple comparisons. Source data are provided as a Source Data file.

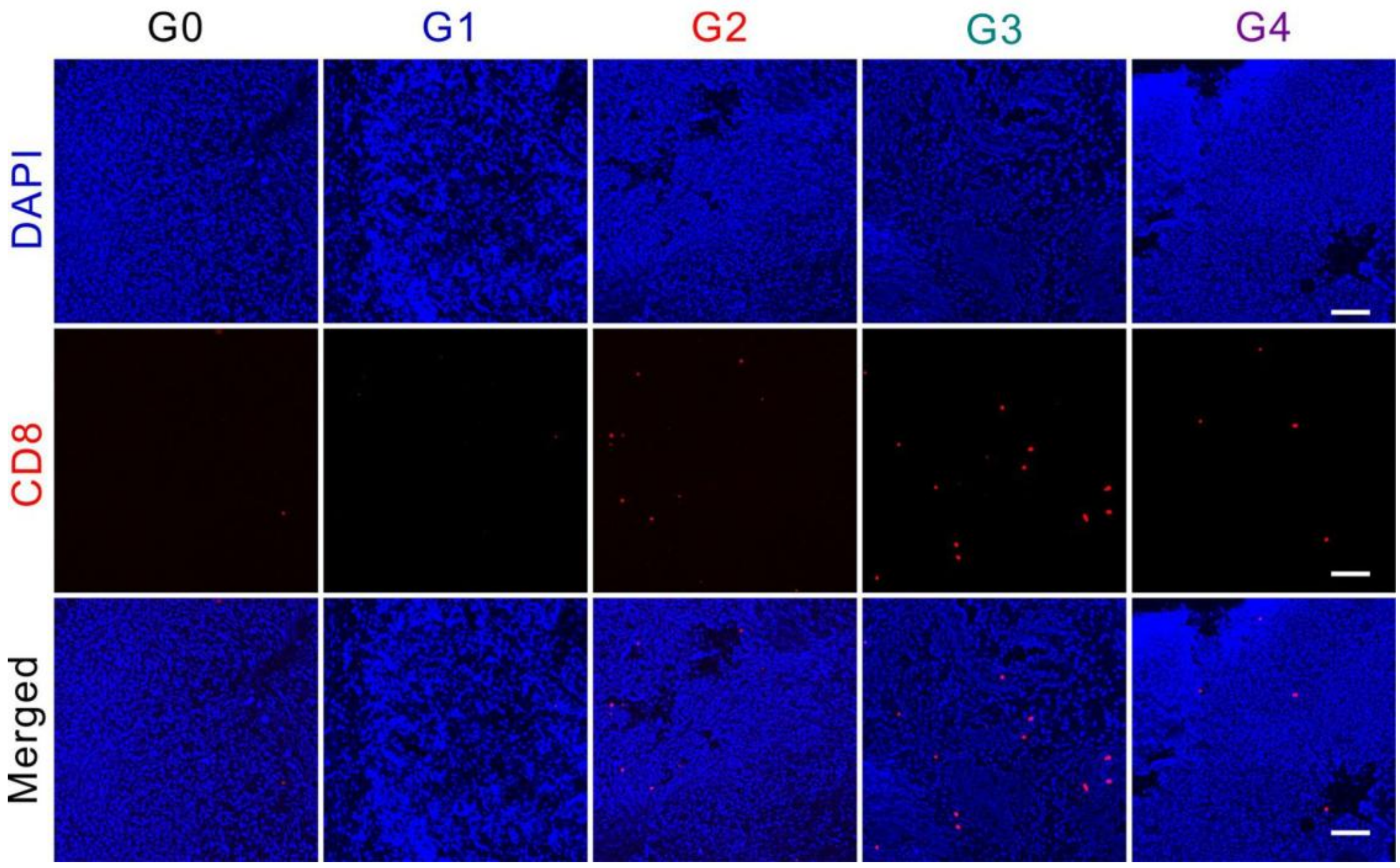

Supplementary Fig. 39 Representative immunofluorescence images of $CD8^+$ T cell staining in tumor tissues on day 14 after different treatments. G0: Blank; G1: GB1275; G2: Lip@V; G3: GB1275 + Lip@V; G4: GB1275 + Lip. Scale bar: 200 μm.

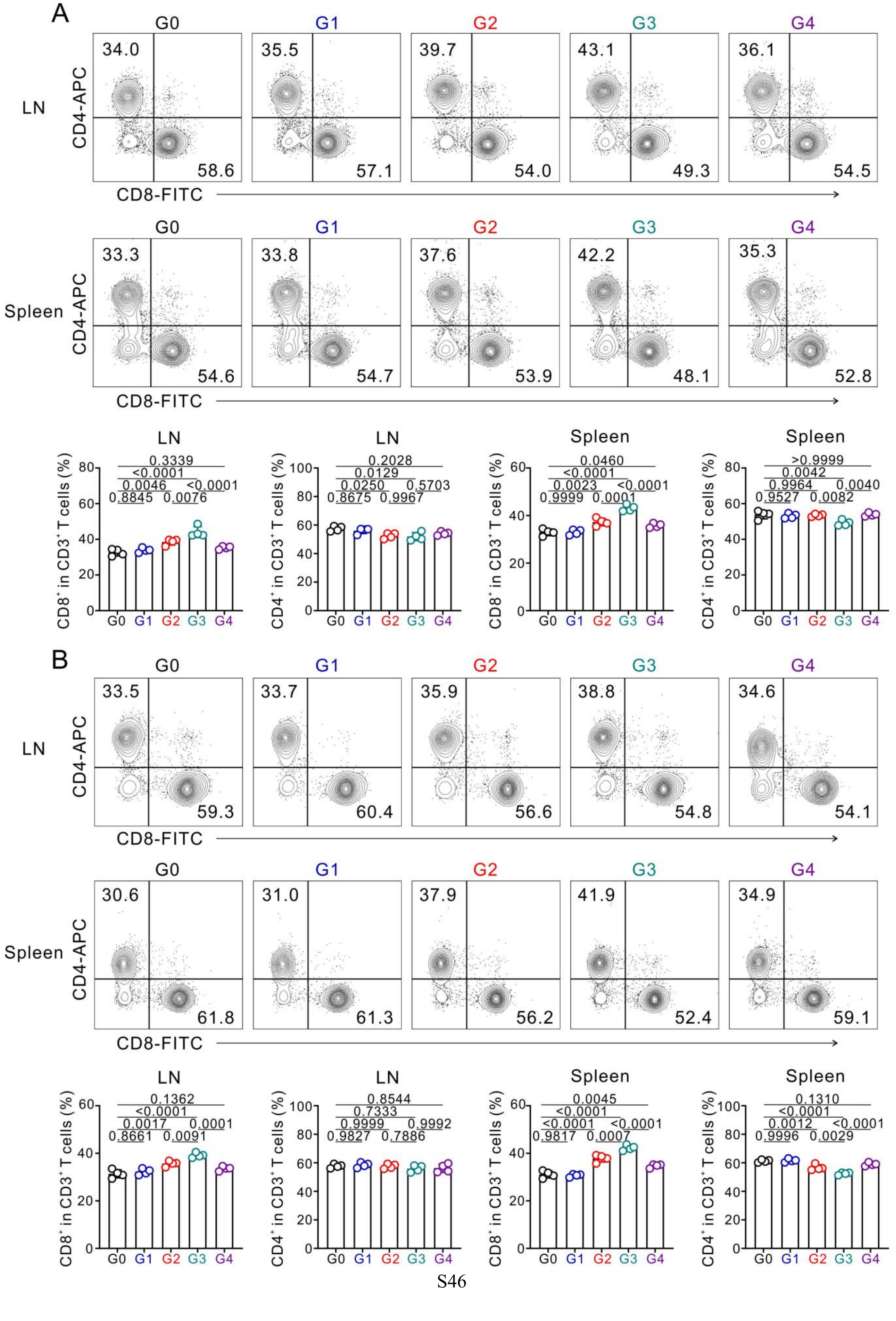

A
B
G0
G1
G2
G3
G4
LN
Spleen
CD4-APC
CD8-FITC
CD8⁺ in CD3⁺ T cells (%)
CD4⁺ in CD3⁺ T cells (%)

Supplementary Fig. 40 Flow cytometry analysis and corresponding statistics of $CD4^+$ T and $CD8^+$ T cells infiltration in LN and spleen after different treatments on **(A)** day 14 and **(B)** day 17, $n = 4$ independent samples. LN represents lymph nodes. G0: Blank; G1: GB1275; G2: Lip@V; G3: GB1275 + Lip@V; G4: GB1275 + Lip. The data are expressed as mean ± s.d. All replicates are biological, and the P values were determined using one-way ANOVA followed by Tukey's multiple comparisons. Source data are provided as a Source Data file.

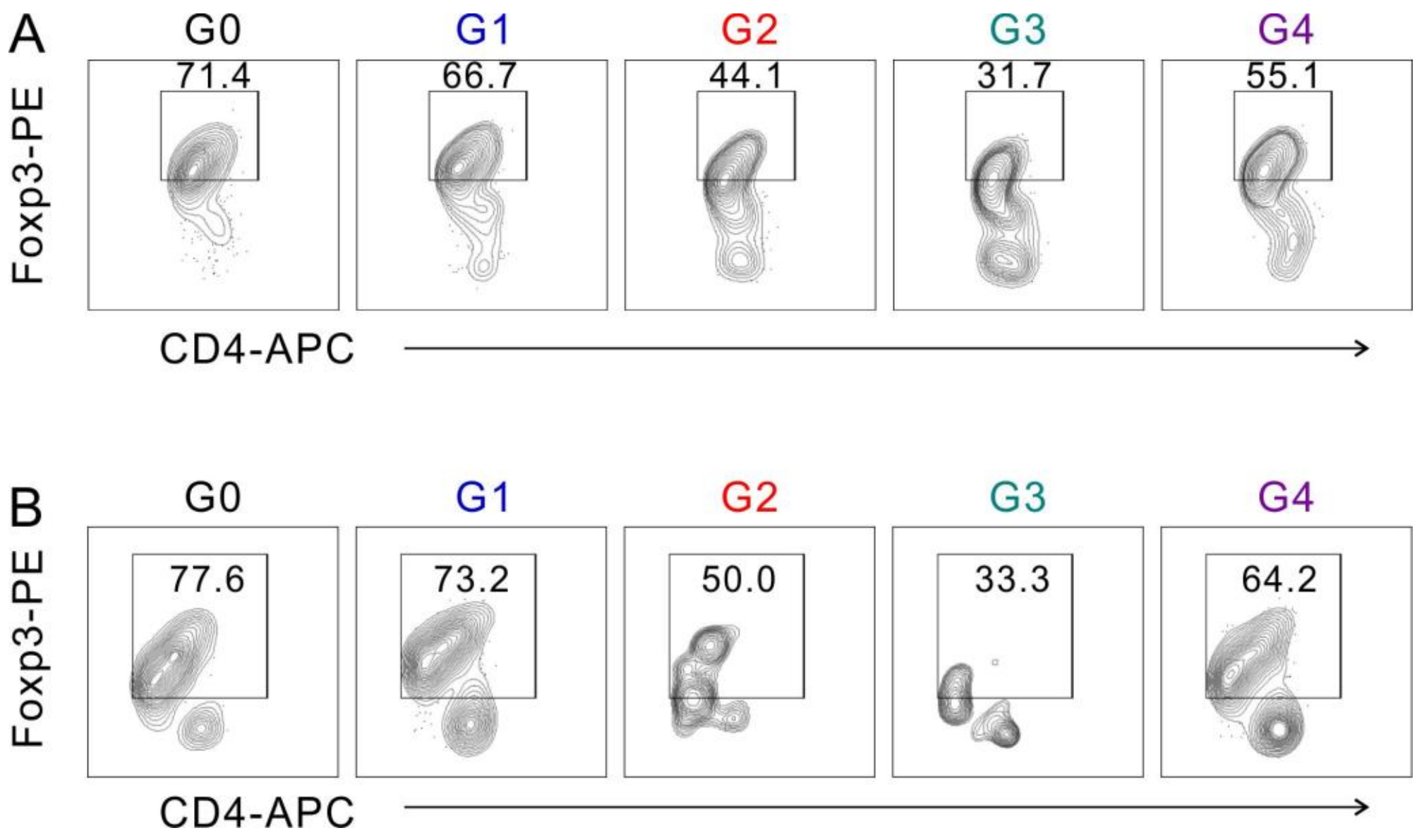

Supplementary Fig. 41 Flow cytometry analysis Tregs infiltration in tumor tissues after different treatments on **(A)** day14 and **(B)** day 17. G0: Blank; G1: GB1275; G2: Lip@V; G3: GB1275 + Lip@V; G4: GB1275 + Lip.

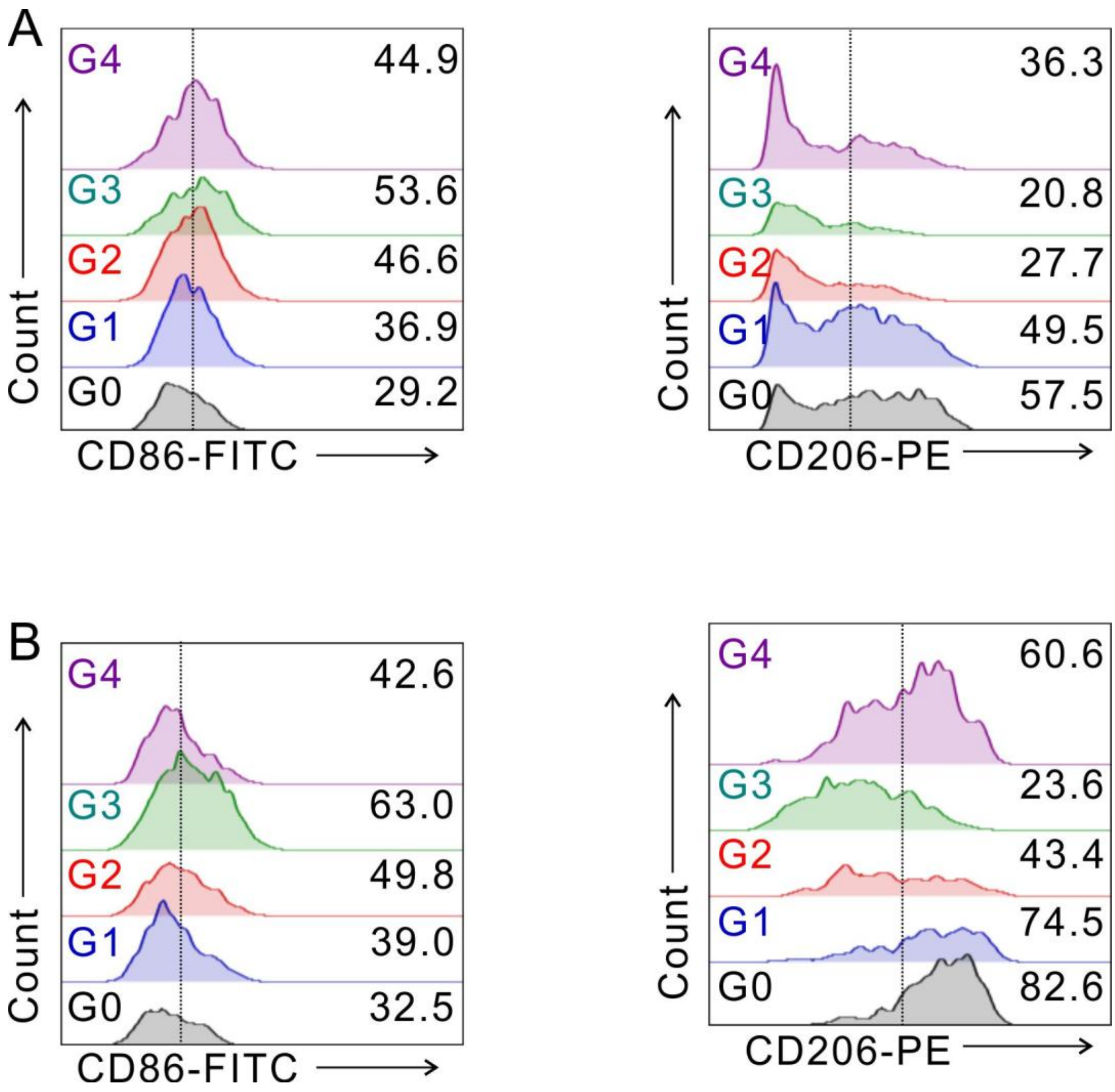

Supplementary Fig. 42 Flow cytometry analysis of the proportion of M1 and M2 cells in tumor tissues after different treatments on **(A)** day14 and **(B)** day 17. G0: Blank; G1: GB1275; G2: Lip@V; G3: GB1275 + Lip@V; G4: GB1275 + Lip.

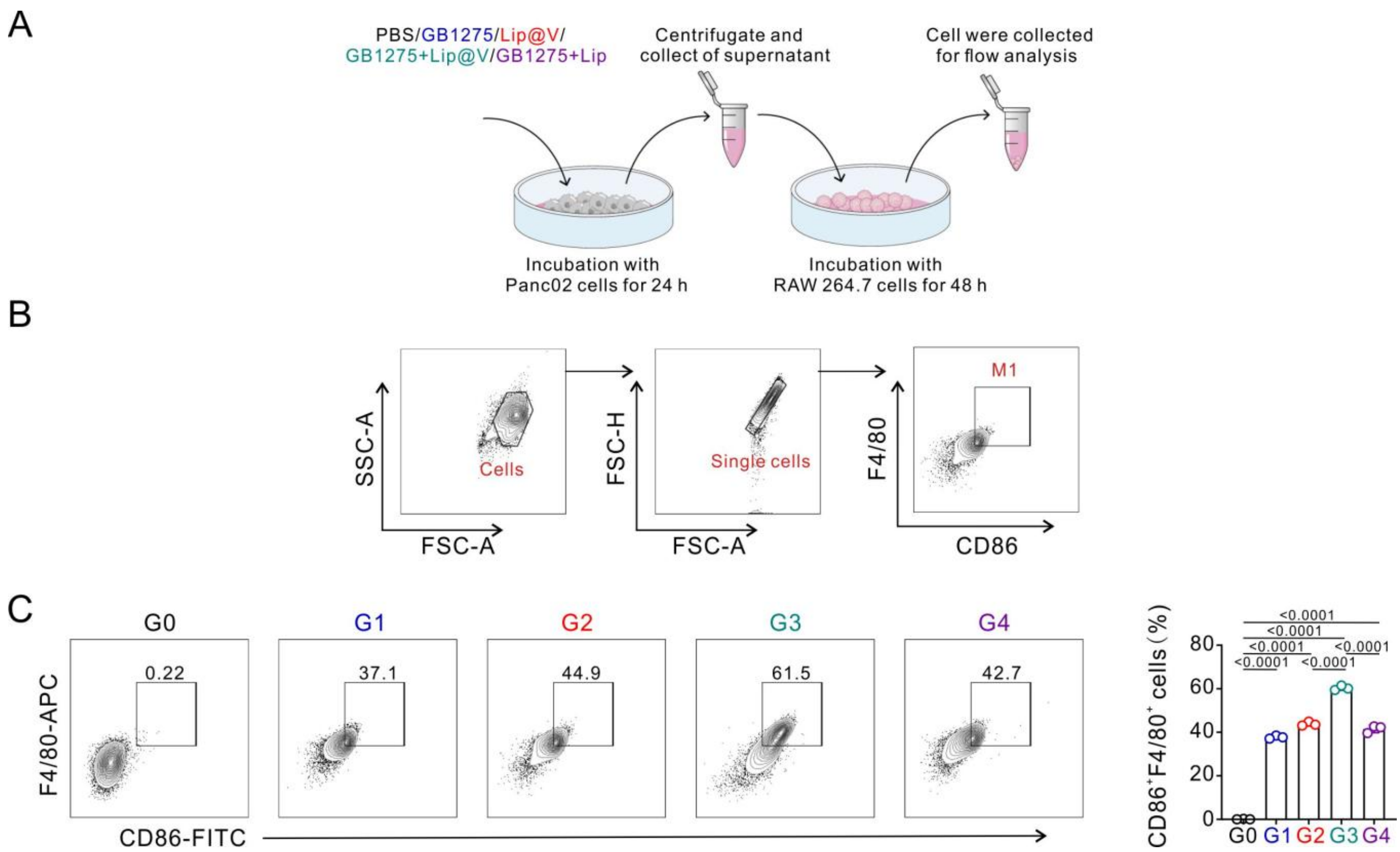

Supplementary Fig. 43 **A** Schematic illustration of the experimental protocols for macrophage polarization in vitro. **B** Gating strategy for flow cytometry analysis of $CD86^+F4/80^+$ cells. **C** Flow cytometry analysis and corresponding statistics of M1 macrophage after different treatments, *n* = 3 independent samples. G0: Blank; G1: GB1275; G2: Lip@V; G3: GB1275 + Lip@V; G4: GB1275 + Lip. The data are expressed as mean ± s.d. All replicates are biological, and the *P* values were determined using one-way ANOVA followed by Tukey's multiple comparisons. Source data are provided as a Source Data file.

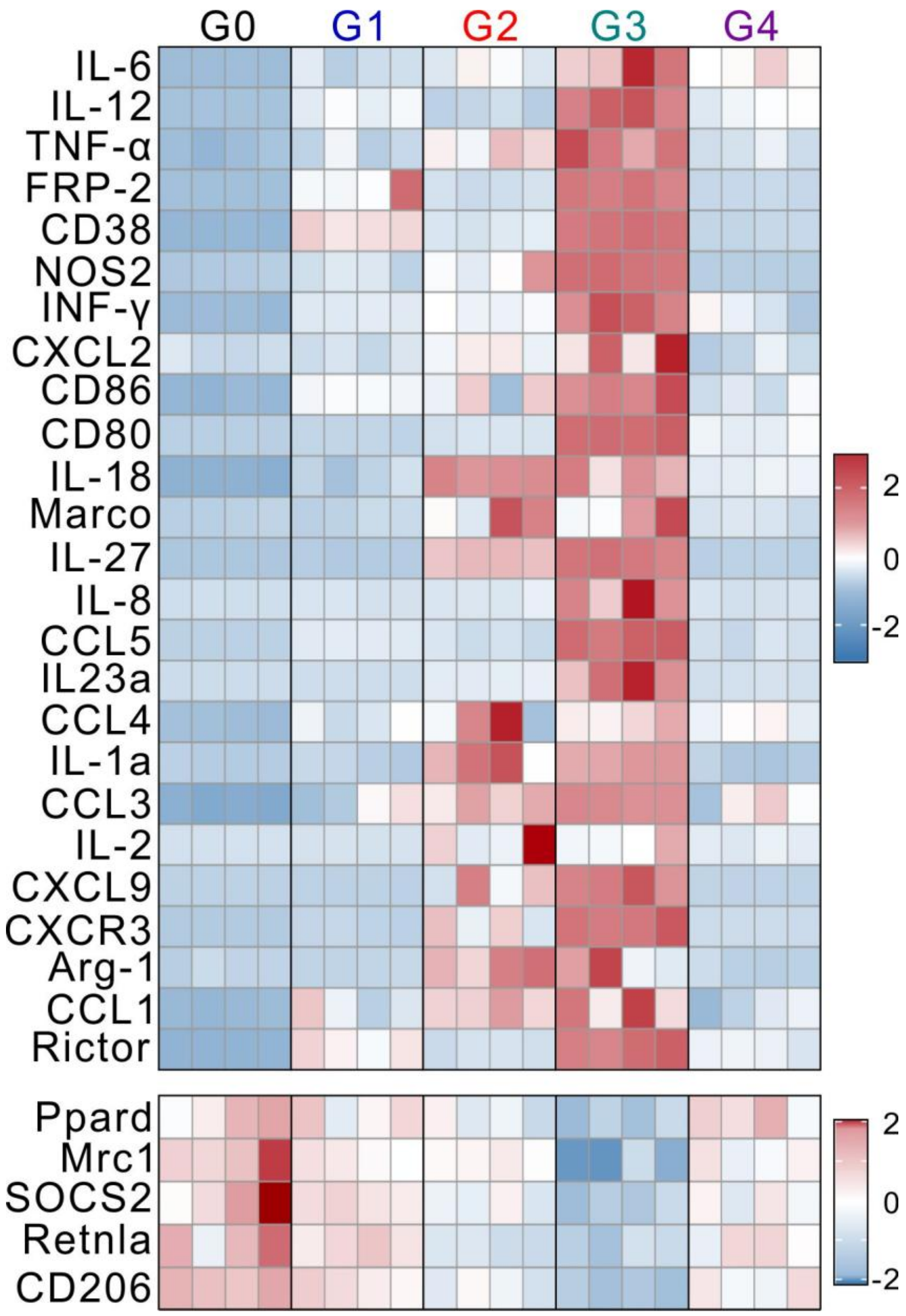

Supplementary Fig. 44 Heatmap of differential gene cluster analysis of M1-and M2-related phenotypes in tumor tissue after different treatments on day 14. G0: Blank; G1: GB1275; G2: Lip@V; G3: GB1275 + Lip@V; G4: GB1275 + Lip. Source data are provided as a Source Data file.

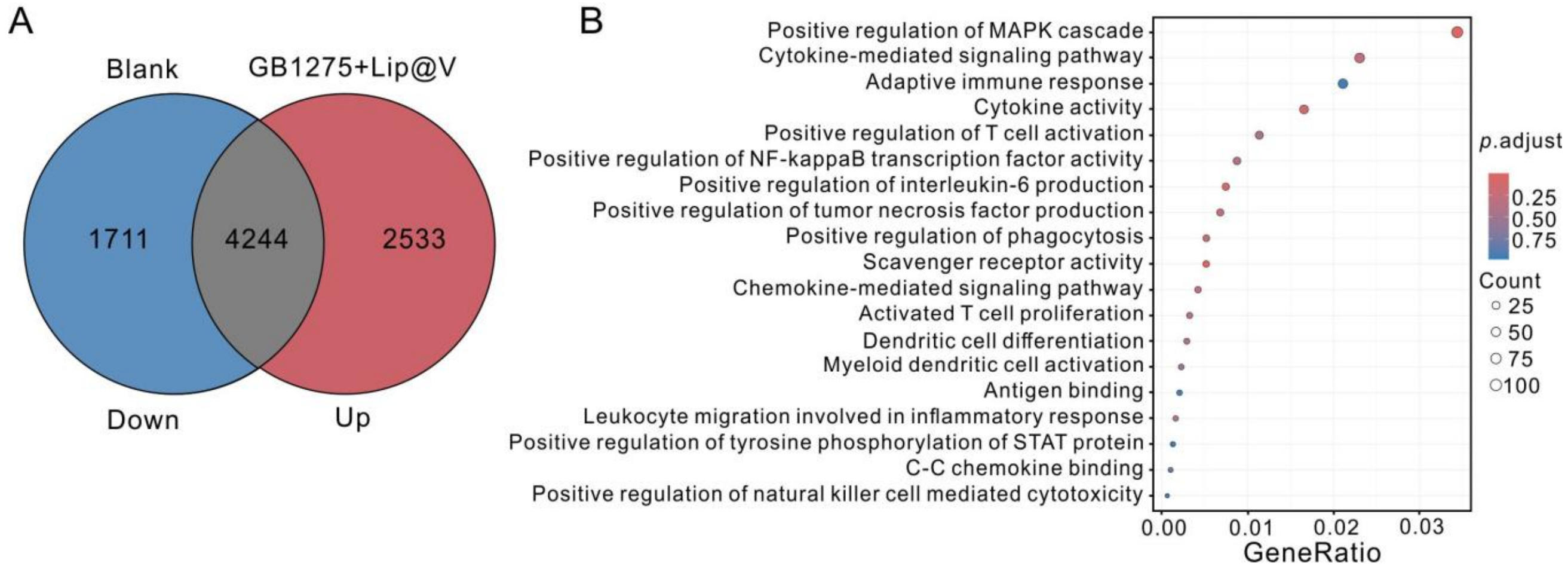

Supplementary Fig. 45 **A** Venn diagram of primary transcripts of Panc02 tumors in the control group and GB1275 + Lip@V treatment group. **B** GO enrichment analysis of major types of immune/inflammation-related biological processes after GB1275 + Lip@V treatment. In the GO enrichment analysis, gene ratio is the ratio of the number of differential genes to the total number of genes in the pathway.

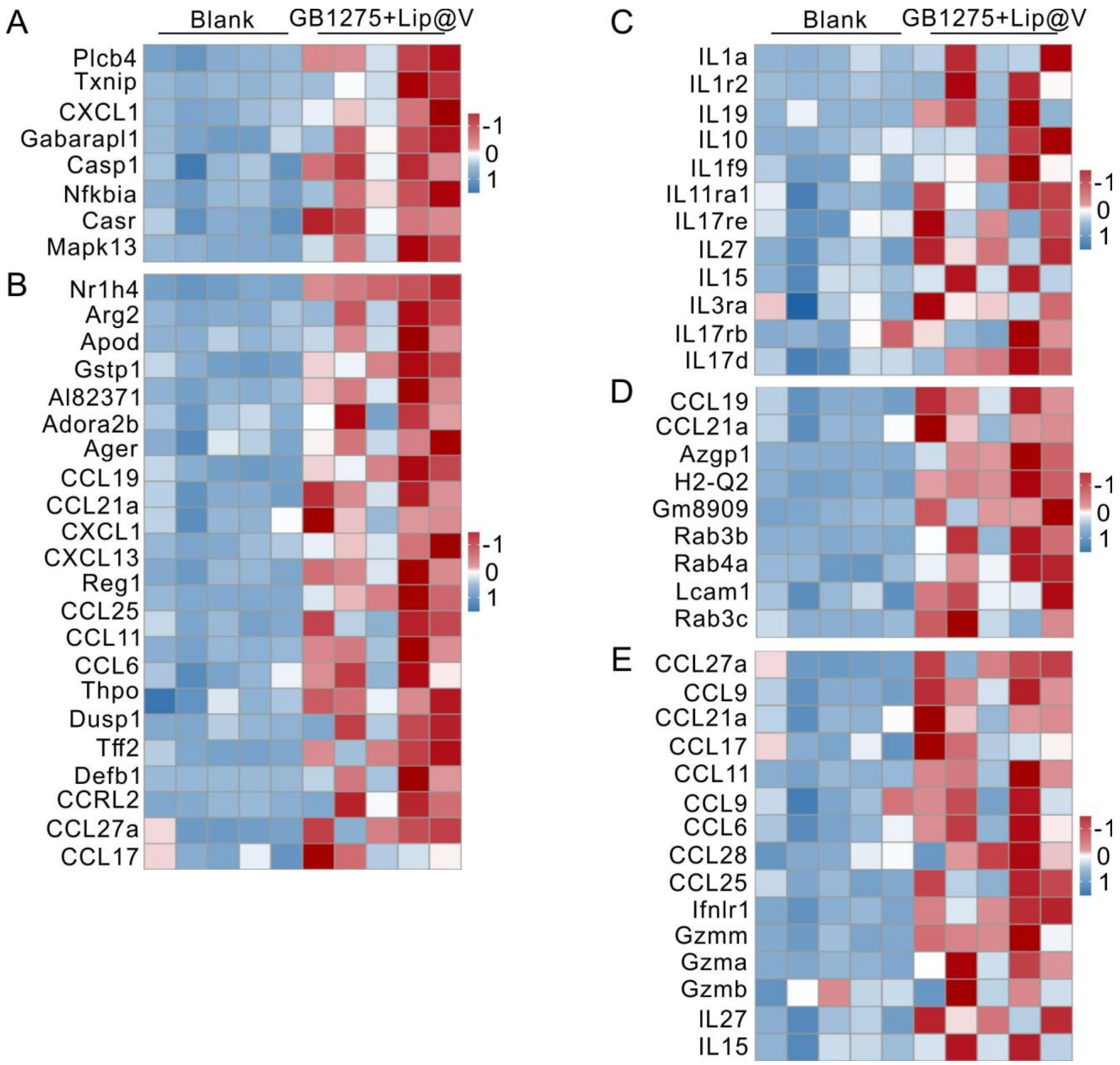

Supplementary Fig. 46 Supplementary Fig. 46 Heatmaps of *z*-scores for upregulated genes in (**A**) NOD-like receptor signaling pathway-related, (**B**) cytokine-related, (**C**) IL-related, (**D**) antigen-processing-related, and (**E**) T-cell-related between the control group and the GB1275 + Lip@V treatment group.

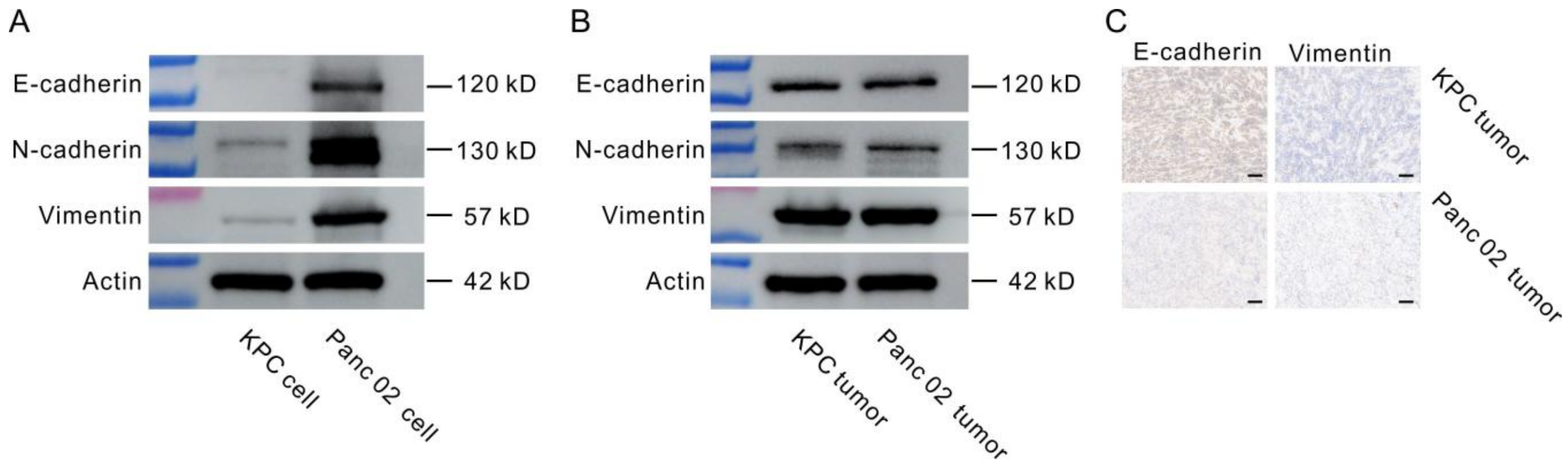

Supplementary Fig. 47 Representative immunoblot of E-cadherin, N-cadherin, Vimentin and actin (loading control) in (**A**) KPC/Panc02 cells as well as (**B**) subcutaneous tumor tissues derived from the two cell lines. **C** Representative IHC staining of E-cadherin and Vimentin in KPC and Panc02 tumor tissues. Scale bar: 100 μm.

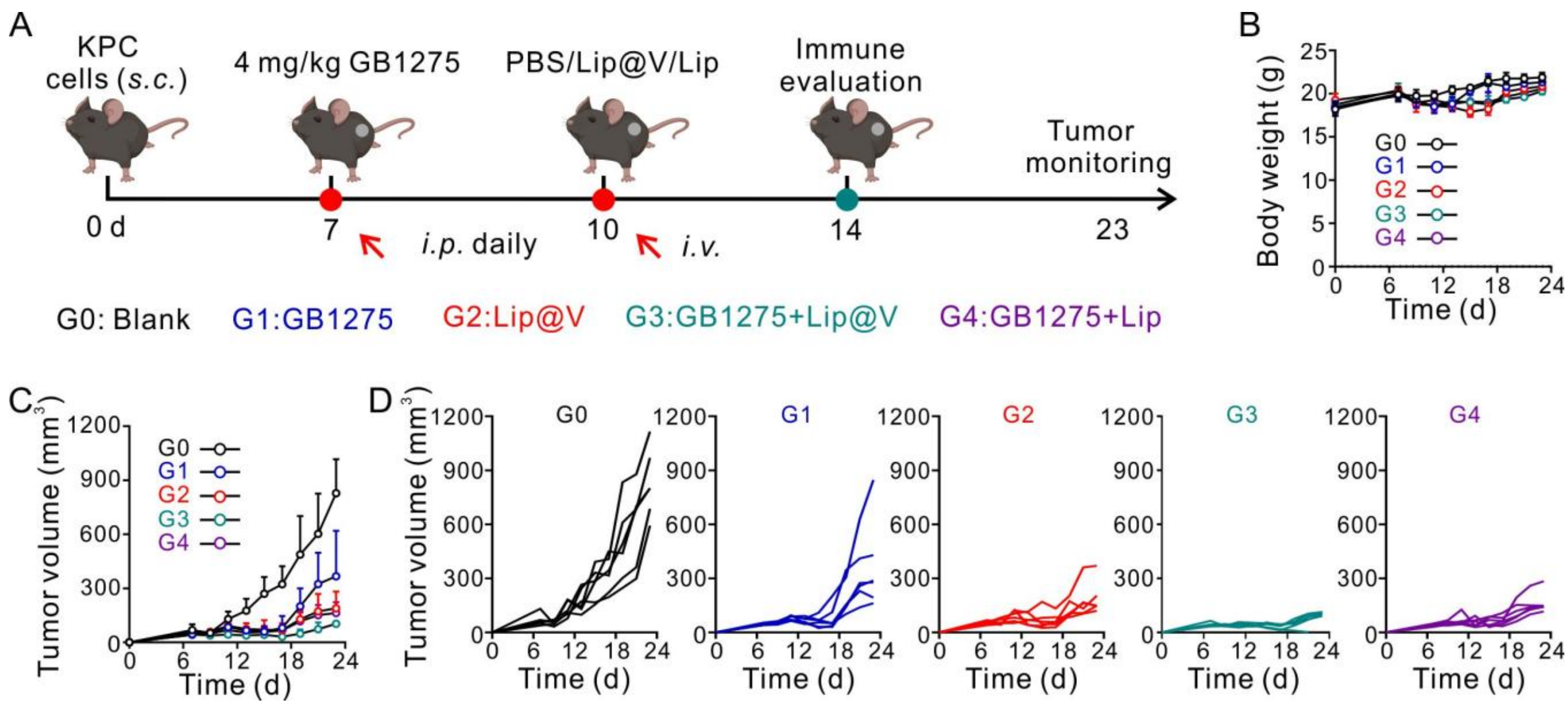

Supplementary Fig. 48 **A** Schematic illustration of the experimental protocols for the combination of GB1275 with drug-loaded bacteria for anti-KPC tumor therapy. *s.c.*, subcutaneous injection. *i.p.* daily, daily intraperitoneal injection. *i.v.*, intravenous injection. **B** Changes in mouse body weights of mice after different treatments, $n = 6$ independent samples. **C, D** Tumor growth curves and individual tumor growth curves after different treatments. $n = 6$ independent samples in Supplementary Fig. 49C. The data are expressed as mean ± s.d. All replicates are biological, and the P values in C were determined using two-way ANOVA followed by Tukey's multiple comparisons tests. Source data are provided as a Source Data file. Figure A was created in BioRender. Zhang, B. (2026) https://BioRender.com/79b3hb8.

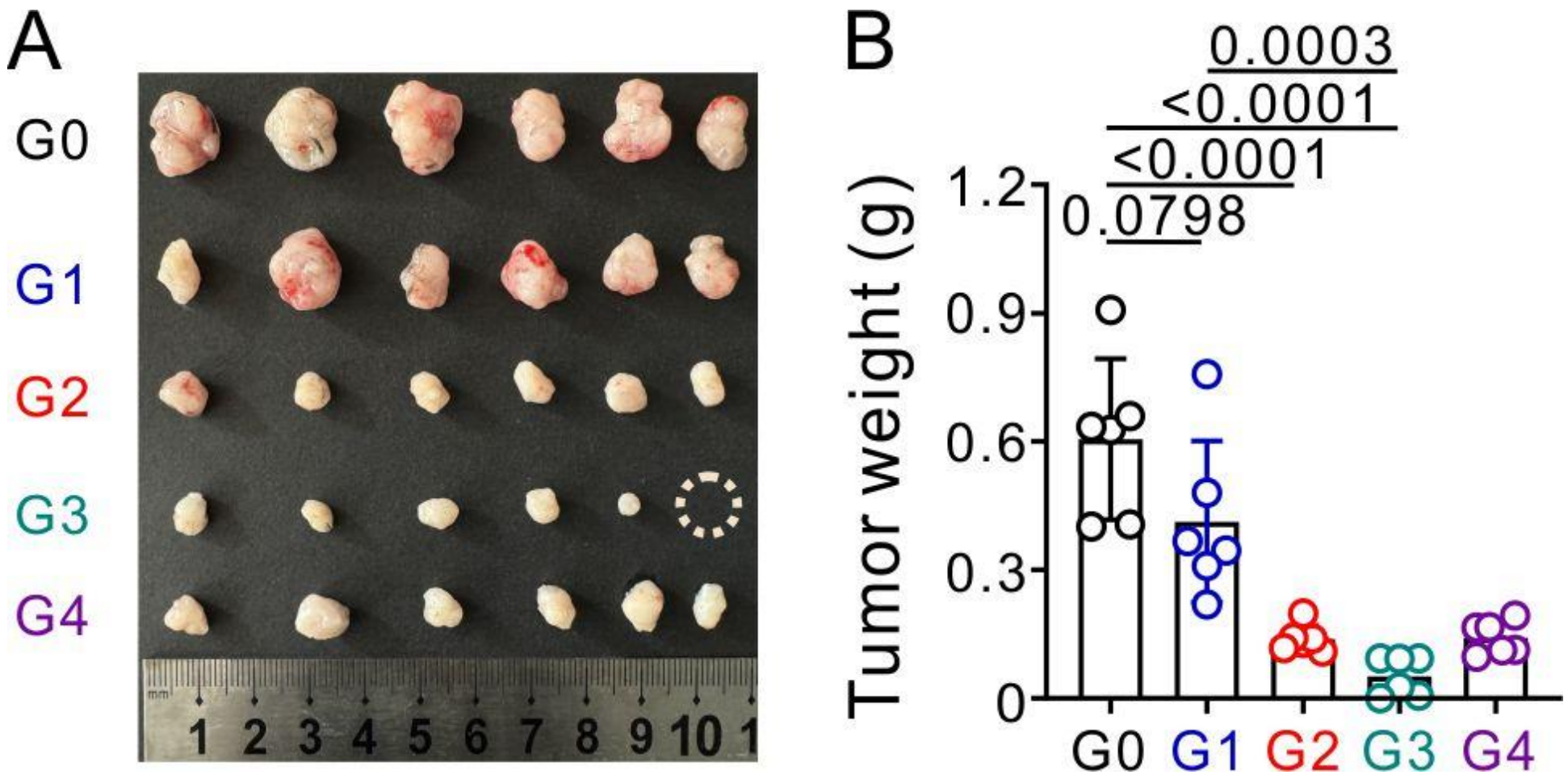

Supplementary Fig. 49 **A, B** Photographs and tumor weight analysis of the harvested tumors from different treatment groups, *n* = 6 independent samples. G0: Blank; G1: GB1275; G2: Lip@V; G3: GB1275 + Lip@V; G4: GB1275 + Lip. The data are expressed as mean ± s.d. All replicates are biological, and the *P* values were determined using one-way ANOVA followed by Tukey's multiple comparisons tests. Source data are provided as a Source Data file.

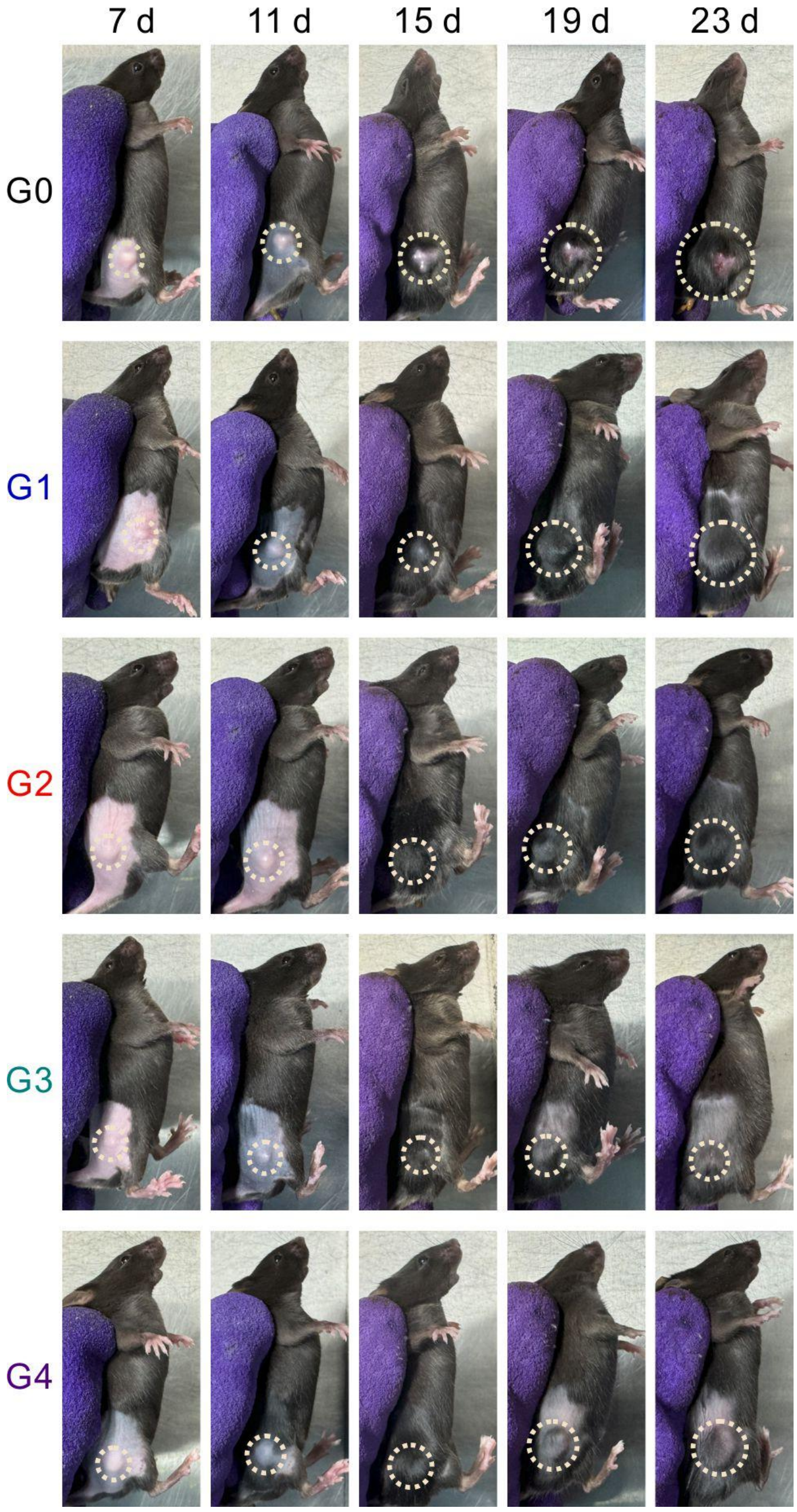

Supplementary Fig. 50 **G** Representative photographs of mice at different time points after different treatments. The dotted circle represents the tumor site. G0: Blank; G1: GB1275; G2: Lip@V; G3: GB1275 + Lip@V; G4: GB1275 + Lip.

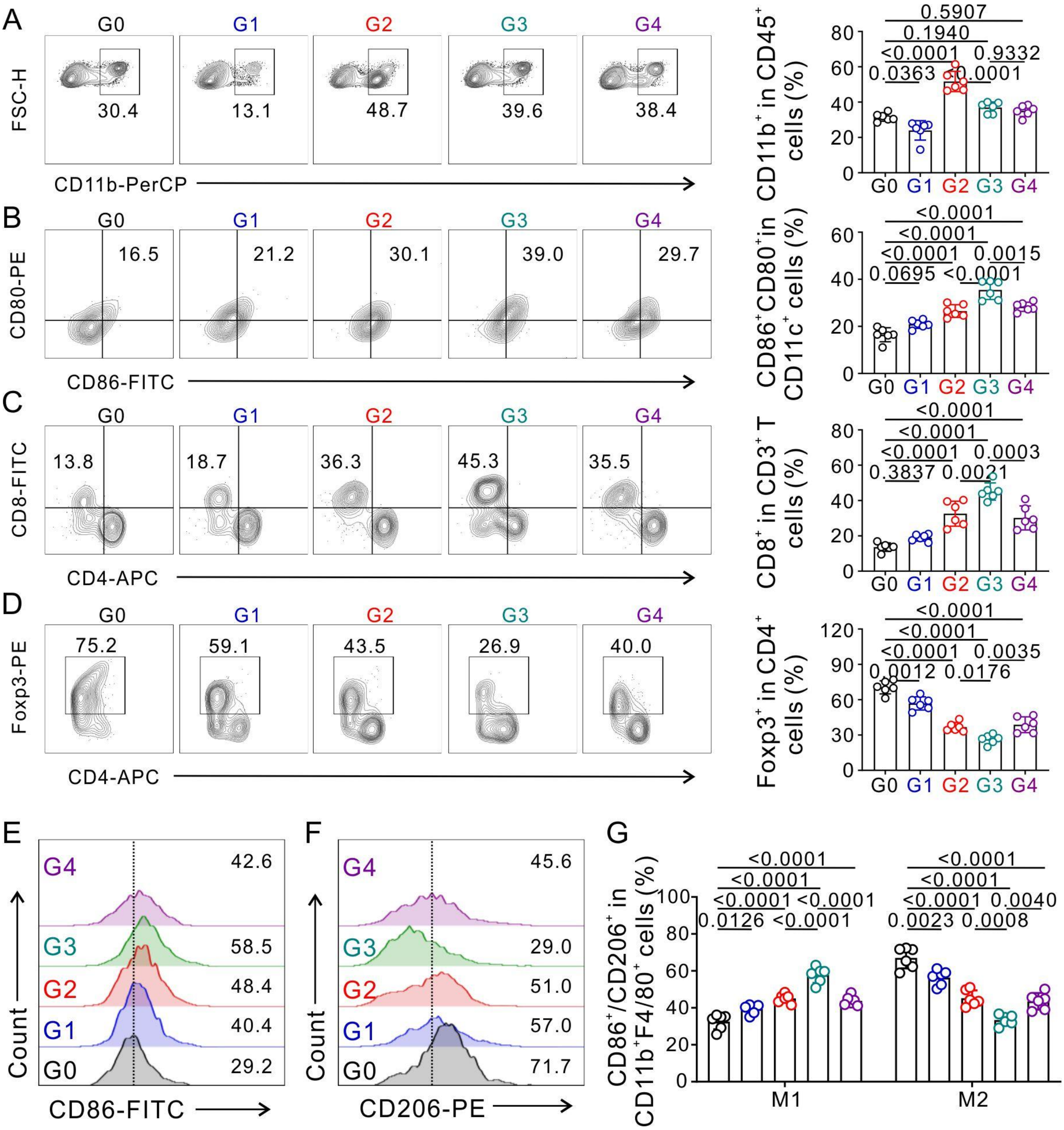

Supplementary Fig. 51 **A-D** Flow cytometry analysis and the corresponding statistics of CD11b^{+} cells, DCs, CD8^{+} T cells and Tregs infiltration in KPC tumor tissues after different treatments on day 14， *n* = 6 independent samples. **E-G** Flow cytometry analysis and the corresponding statistics of the proportion of M1 and M2 cells in KPC tumor tissues after different treatments on day 14, *n* = 6 independent samples. G0: Blank; G1: GB1275; G2: Lip@V; G3: GB1275 + Lip@V; G4: GB1275 +

Lip. The data are expressed as mean ± s.d. All replicates are biological, and the P values were determined using one-way ANOVA followed by Tukey's multiple comparisons tests. Source data are provided as a Source Data file.

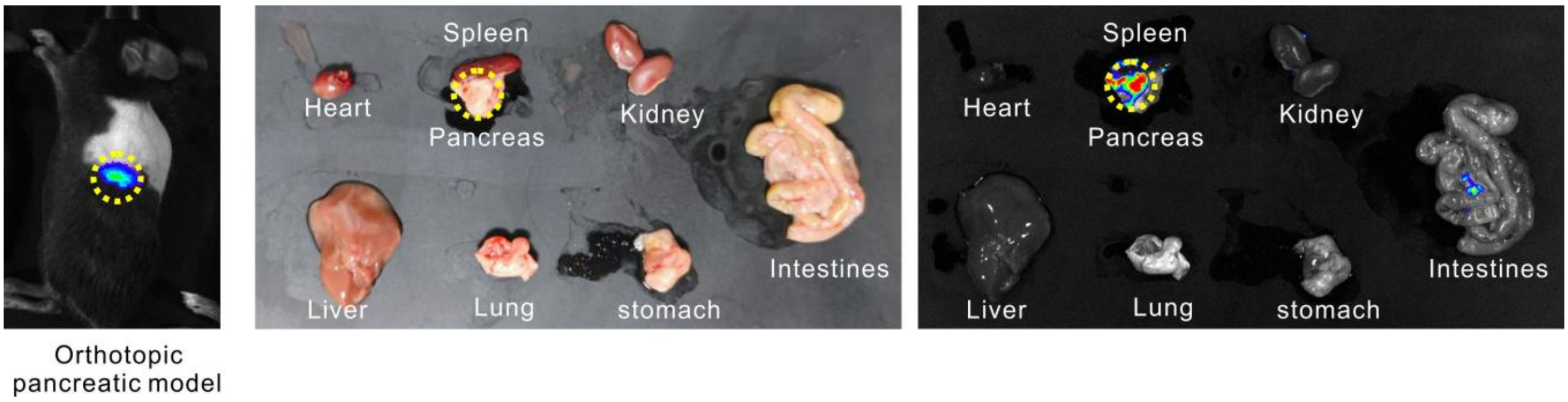

Supplementary Fig. 52 Bioluminescence imaging of in orthotopic pancreatic cancer models in C57BL/6 mice established with Panc02-luc cells, including whole-body bioluminescence imaging and detailed bioluminescent visualization of major organs and the tumor.

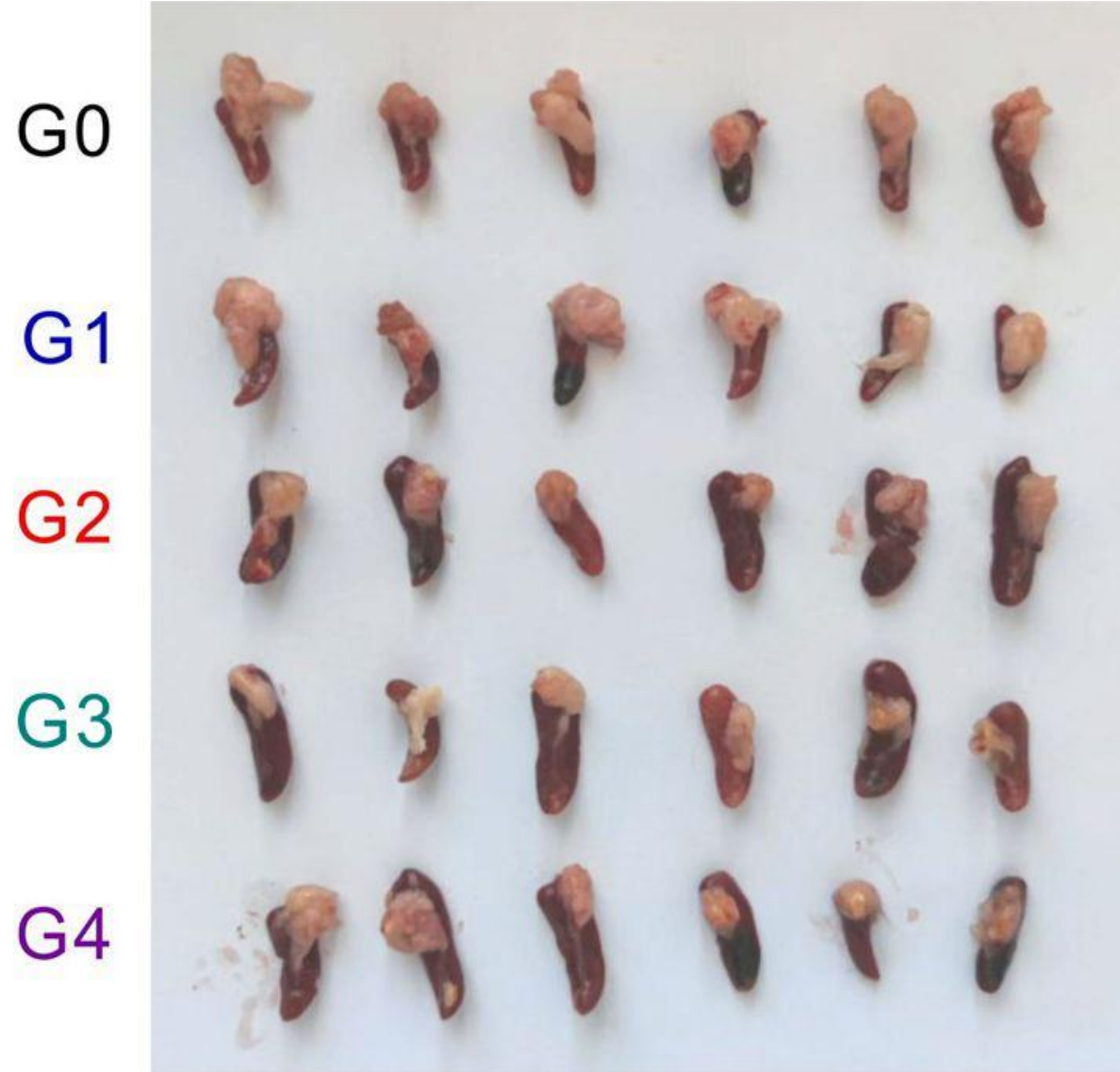

Supplementary Fig. 53 Bioluminescence imaging of spleens and orthotopic pancreatic tumors after different treatments. G0: Blank; G1: GB1275; G2: Lip@V; G3: GB1275 + Lip@V; G4: GB1275 + Lip.

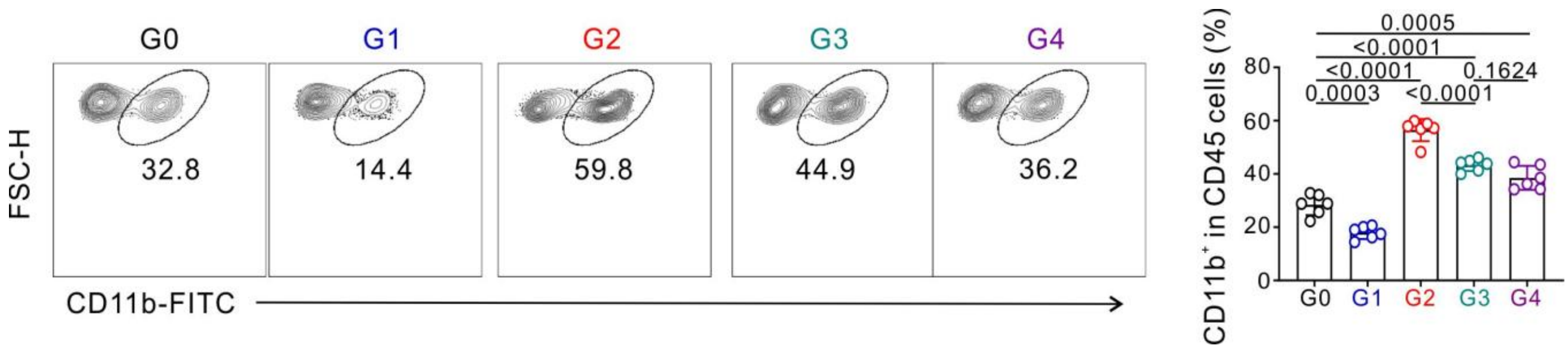

Supplementary Fig. 54 Flow cytometry analysis and corresponding statistics of CD11b$^+$ cells infiltration in tumor tissues after different treatments on day 14, $n = 6$ independent samples. G0: Blank; G1: GB1275; G2: Lip@V; G3: GB1275 + Lip@V; G4: GB1275 + Lip. The data are expressed as mean ± s.d. All replicates are biological, and the *P* values were determined using one-way ANOVA followed by Tukey's multiple comparisons. Source data are provided as a Source Data file.

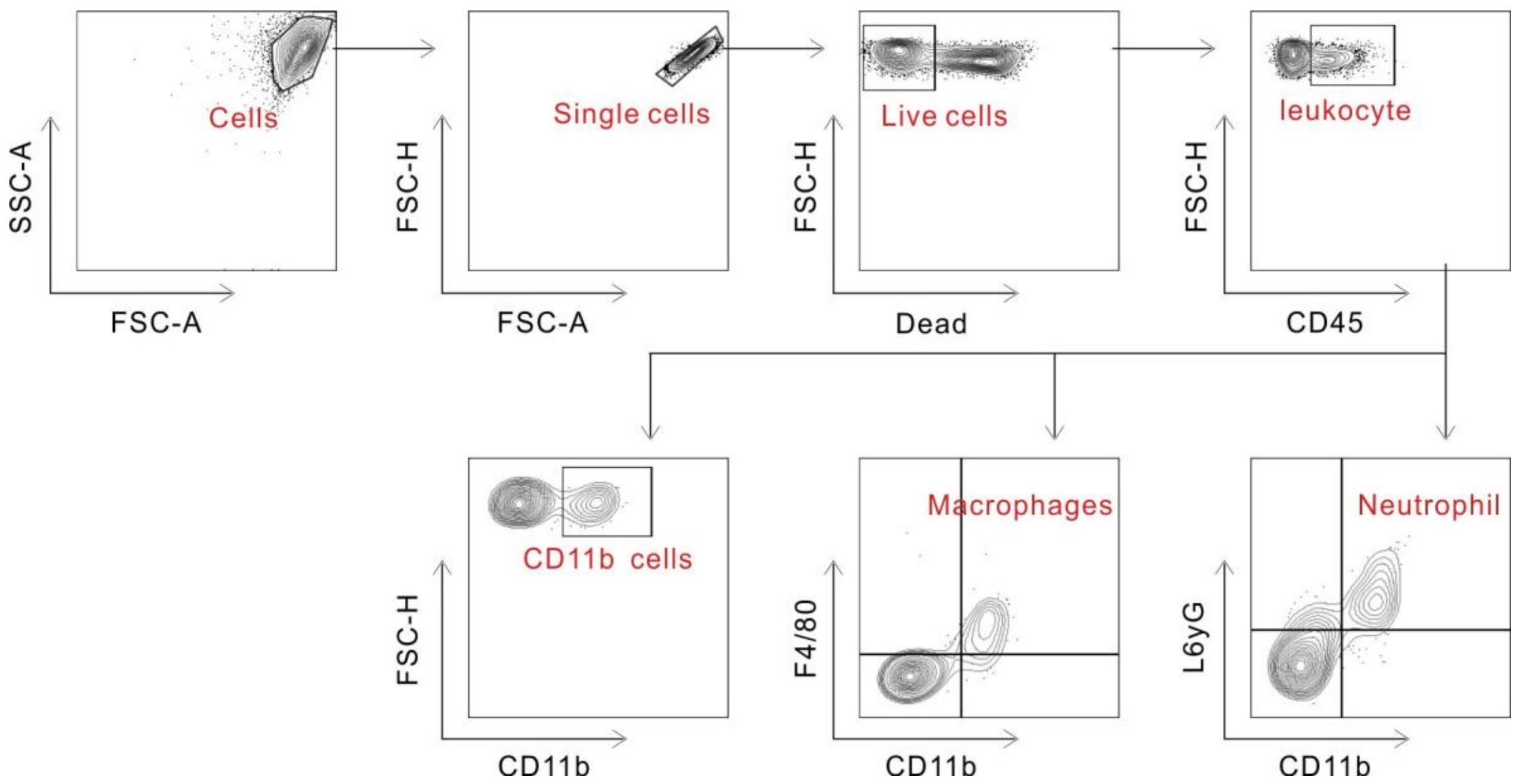

Supplementary Fig. 55 The gating process of flow cytometry analysis for CD11b$^+$ cells and their subsets (macrophages and neutrophils) in Fig. 2 and Fig. 3.

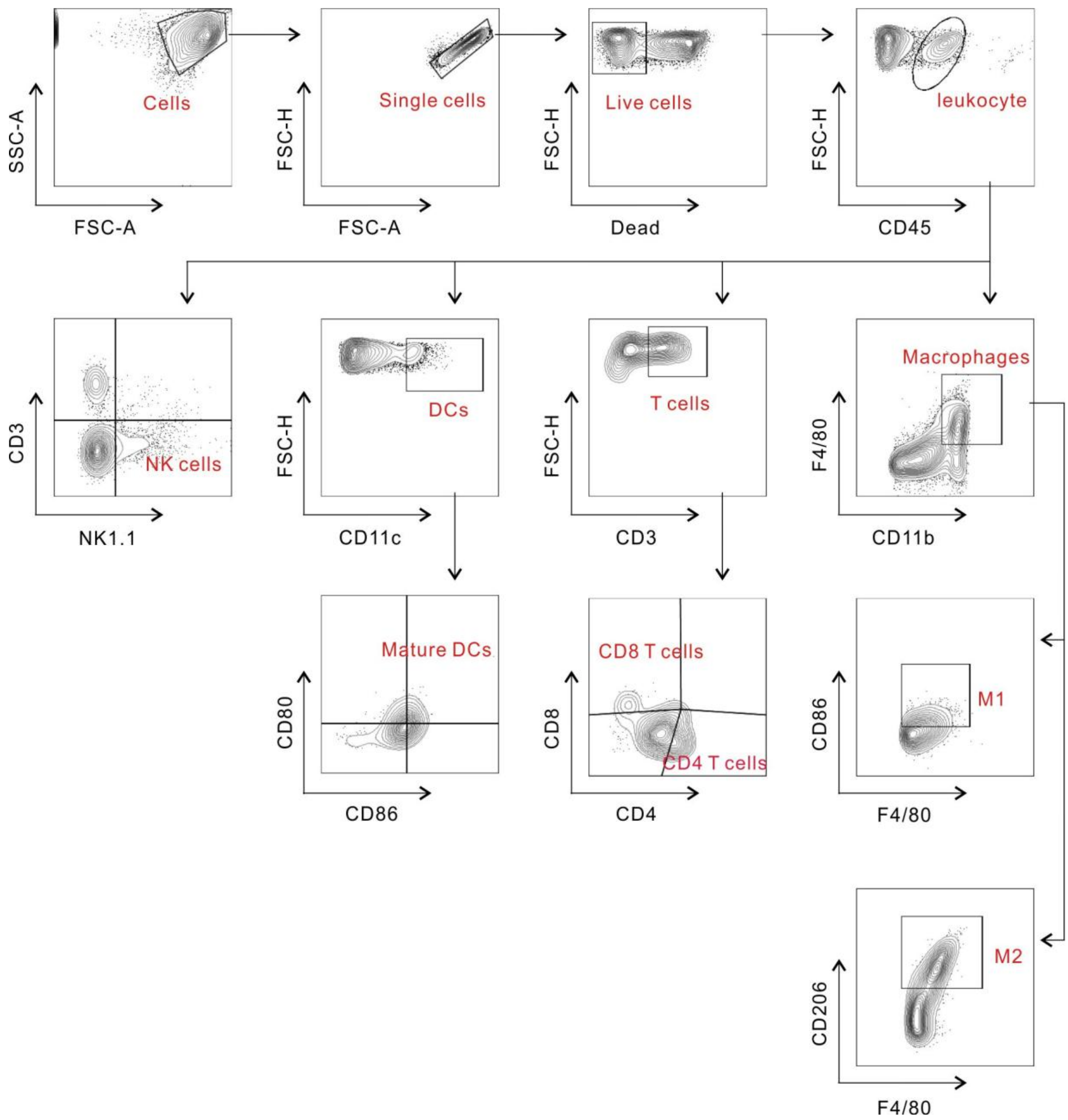

Supplementary Fig. 56 The gating process of flow cytometry analysis for $CD45^+$ immune cells and their subsets in Fig. 4, including $CD45^+$ cells, NK cells, mature DCs, $CD8^+$ T cells, $CD4^+$ T cells, as well as macrophage subsets (M1-type macrophages and M2-type macrophages).

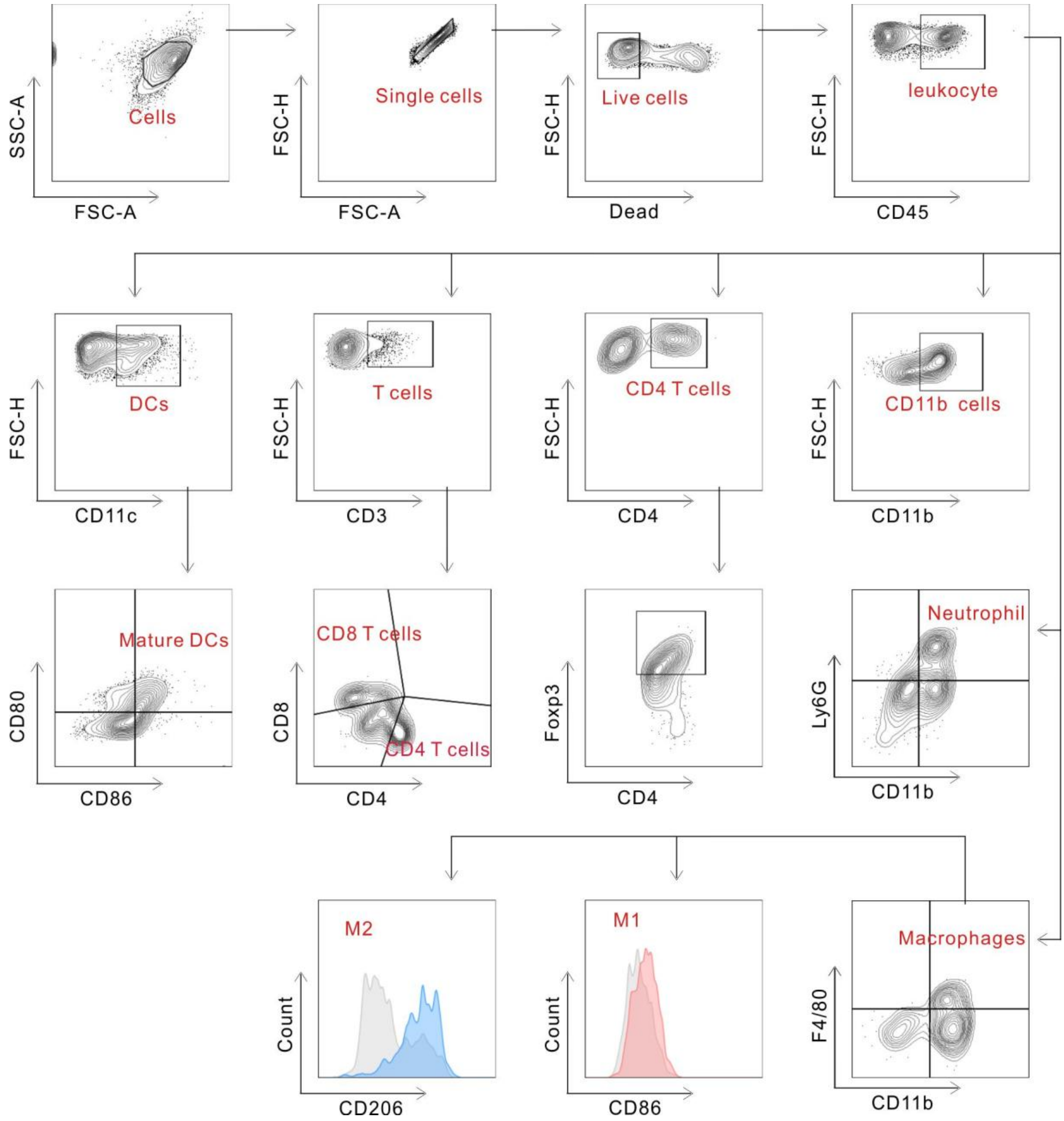

Supplementary Fig. 57 The gating process of flow cytometry analysis for $CD11b^{+}$ cells and their subsets (macrophages and neutrophils), DCs, $CD8^{+}$ T cells, $CD4^{+}$ T cells, Tregs, as well as macrophage subsets (M1-type macrophages and M2-type macrophages) in Fig. 6.

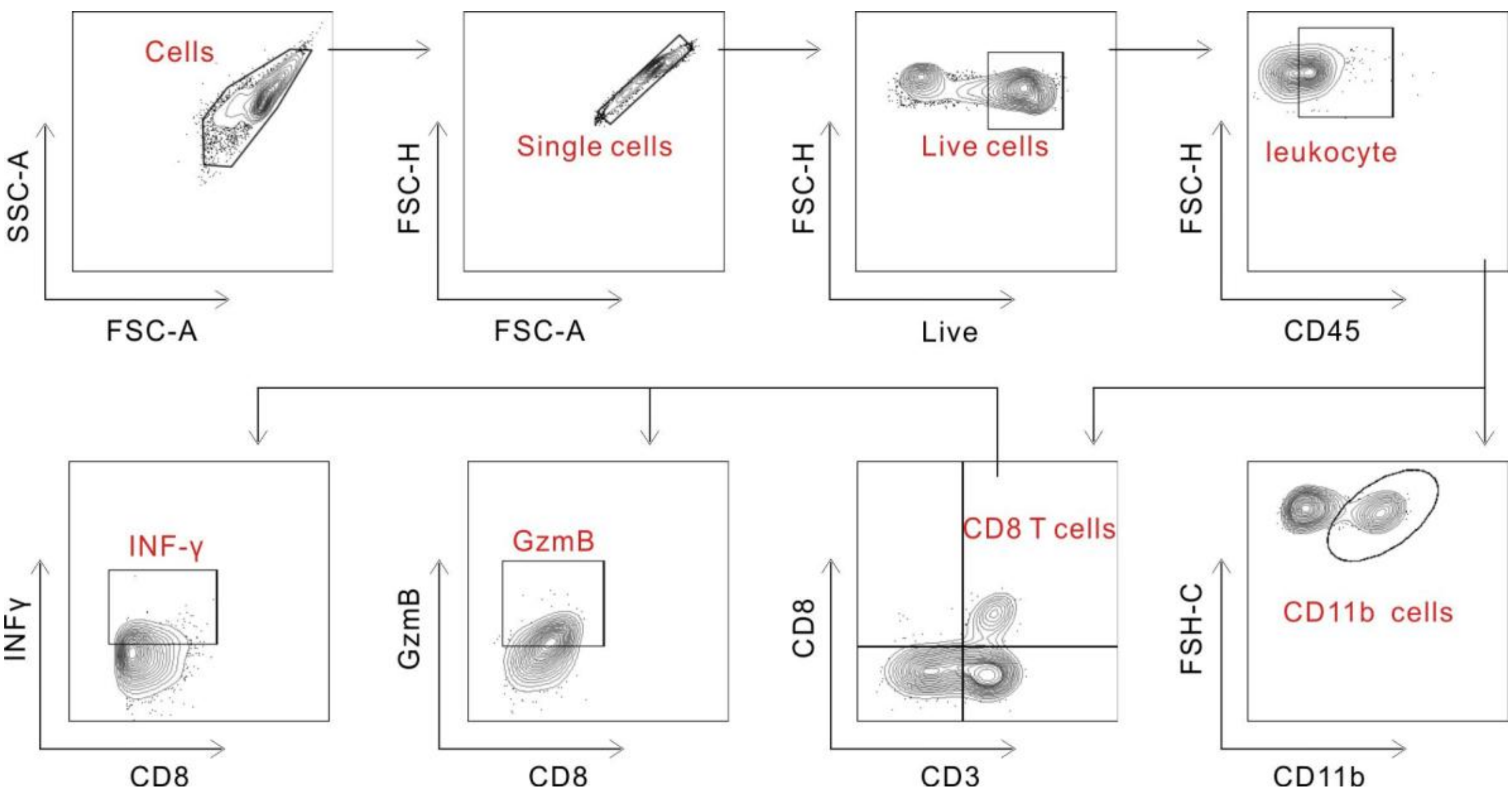

Supplementary Fig. 58 The gating process of flow cytometry analysis for CD11b⁺ cells, CD8⁺ T cells and their functional subsets (IFN-γ⁺CD8⁺T cells, GzmB⁺CD8⁺T cells) in Fig. 7.
