# Supplementary material for: CD11b agonist GB1275 potentiates oncolytic bacterial therapy against pancreatic cancer in mouse tumor models
Source: Nat Commun. 2026 Aug 21;17:10739. doi: 10.1038/s41467-026-76823-7 (PMC13661619; doi:10.1038/s41467-026-76823-7)
Supplement: Supplementary file 2 [file 41467_2026_76823_MOESM2_ESM.pdf]

nature portfolio

Corresponding author(s): Yuan-Di Zhao

Last updated by author(s): Jul 27, 2026

Reporting Summary

Nature Portfolio wishes to improve the reproducibility of the work that we publish. This form provides structure for consistency and transparency in reporting. For further information on Nature Portfolio policies, see our Editorial Policies and the Editorial Policy Checklist.

Statistics

For all statistical analyses, confirm that the following items are present in the figure legend, table legend, main text, or Methods section.

n/a	Confirmed	
☐	☒	The exact sample size (*n*) for each experimental group/condition, given as a discrete number and unit of measurement
☐	☒	A statement on whether measurements were taken from distinct samples or whether the same sample was measured repeatedly
☐	☒	The statistical test(s) used AND whether they are one- or two-sided<br>*Only common tests should be described solely by name; describe more complex techniques in the Methods section.*
☒	☐	A description of all covariates tested
☐	☒	A description of any assumptions or corrections, such as tests of normality and adjustment for multiple comparisons
☐	☒	A full description of the statistical parameters including central tendency (e.g. means) or other basic estimates (e.g. regression coefficient) AND variation (e.g. standard deviation) or associated estimates of uncertainty (e.g. confidence intervals)
☐	☒	For null hypothesis testing, the test statistic (e.g. *F*, *t*, *r*) with confidence intervals, effect sizes, degrees of freedom and *P* value noted<br>*Give P values as exact values whenever suitable.*
☒	☐	For Bayesian analysis, information on the choice of priors and Markov chain Monte Carlo settings
☒	☐	For hierarchical and complex designs, identification of the appropriate level for tests and full reporting of outcomes
☒	☐	Estimates of effect sizes (e.g. Cohen's *d*, Pearson's *r*), indicating how they were calculated

Our web collection on statistics for biologists contains articles on many of the points above.

Software and code

Policy information about availability of computer code

Data collection	Flow-cytomery data were collected with CytExpert (ver. 2.3.0.84). Confocal images were collected with FluoView31S (ver.2.3.1.163).
Data analysis	Flow-cytomery data were analyzed with FlowJo (ver. 10.8.1). Statistical analysis was performed with GraphPad Prism (ver. 8.0); ImageJ (ver. 1.4.3.67) were used to analyze the 3D reconstruction of confocal fluorescence images. Confocal images were analyzed with FluoView31S (ver.2.3.1.163). The plots were assembled with Coreldraw x8 (ver. 18.0.0.450)

For manuscripts utilizing custom algorithms or software that are central to the research but not yet described in published literature, software must be made available to editors and reviewers. We strongly encourage code deposition in a community repository (e.g. GitHub). See the Nature Portfolio guidelines for submitting code & software for further information.

Data

Policy information about availability of data

All manuscripts must include a data availability statement. This statement should provide the following information, where applicable:

- Accession codes, unique identifiers, or web links for publicly available datasets
- A description of any restrictions on data availability
- For clinical datasets or third party data, please ensure that the statement adheres to our policy

All data generated or analyzed during this study are included in this published article and its Supplementary Information file. The raw RNA-seq sequencing data

generated in this study have been deposited in the NCBI Sequence Read Archive (SRA) under BioProject accession PRJNA1501942. Processed gene expression matrices are available on Figshare (DOI: 10.6084/m9.figshare.32943350). Source data are provided with this paper.

Research involving human participants, their data, or biological material

Policy information about studies with human participants or human data. See also policy information about sex, gender (identity/presentation), and sexual orientation and race, ethnicity and racism.

Reporting on sex and gender	The research results do not involve Sex or gender.
Reporting on race, ethnicity, or other socially relevant groupings	This study does not involve reporting on race, ethnicity, or other socially relevant groupings.
Population characteristics	This study does not involve population characteristics.
Recruitment	This study does not involve recruitment.
Ethics oversight	This study does not involve ethics oversight.

Note that full information on the approval of the study protocol must also be provided in the manuscript.

Field-specific reporting

Please select the one below that is the best fit for your research. If you are not sure, read the appropriate sections before making your selection.

☒ Life sciences ☐ Behavioural & social sciences ☐ Ecological, evolutionary & environmental sciences

For a reference copy of the document with all sections, see nature.com/documents/nr-reporting-summary-flat.pdf

Life sciences study design

All studies must disclose on these points even when the disclosure is negative.

Sample size	To ensure the accuracy of the experiments, at least three replicates were performed. In the cell experiments, 3-6 samples were used to analyze toxicity, and therapeutic effect of probes. In antitumor experiments, 5-6 mice each group were used to analyze tumor volume, body weight in mice. For other experiments, the sample size for each group was 3-12.
Data exclusions	No data were excluded from the analyses.
Replication	The experiments were reproduced successfully,the number of experiments was listed in corresponding figure legends.
Randomization	The samples were allocated into experimental groups randomly.
Blinding	The investigators were blinded to group allocation during data collection and analysis.

Reporting for specific materials, systems and methods

We require information from authors about some types of materials, experimental systems and methods used in many studies. Here, indicate whether each material, system or method listed is relevant to your study. If you are not sure if a list item applies to your research, read the appropriate section before selecting a response.

Materials & experimental systems

n/a	Involved in the study	
☐	☒	Antibodies
☐	☒	Eukaryotic cell lines
☒	☐	Palaeontology and archaeology
☐	☒	Animals and other organisms
☒	☐	Clinical data
☒	☐	Dual use research of concern
☒	☐	Plants

Methods

n/a	Involved in the study	
☒	☐	ChIP-seq
☐	☒	Flow cytometry
☒	☐	MRI-based neuroimaging

Antibodies

Antibodies used	Anti-Ly-6G was purchased from Merck Millipore (Darmstadt, Germany). Clodronate Liposomes was obtained purchased from Solarbio (Beijing, China). RS 504393 was purchased from Shanghai Yuanye Biotechnology Co., Ltd (Shanghai, China). Fluorophore-labeled antibodies including FITC anti-mouse CD45 (Clone S18009F, Cat# 103106, 1:200), FITC anti-mouse CD11b (Clone M1/70, Cat# 1012347, 1:200), PerCP anti-mouse CD11b (Clone M1/70, Cat# 10124315, 1:200), APC anti-mouse F4/80 (Clone BM8, Cat# 12314071, 1:200), PE anti-mouse Ly-6G (Clone 1A8, Cat# 12764777, 1:200), FITC anti-mouse CD86 (Clone GL-1, Cat# 1050254, 1:200), PE anti-mouse CD206 (Clone C068C2, Cat# 14177424, 1:200), PE anti-mouse CD3 (Clone 17A2, Cat# 100247, 1:200), APC anti-mouse CD4 (Clone RM4-5, Cat# 10059224, 1:200), PE anti-mouse FOXP3 (Clone MF-14, Cat# 12644660, 1:100), FITC anti-mouse CD8a (Clone 53-6.7, Cat# 10070153, 1:200), APC anti-mouse CD11c (Clone N418, Cat# 11731813, 1:200), PE anti-mouse CD80 (Clone 16-10A1, Cat# 10470043, 1:200), APC anti-mouse IFN-γ (Clone XMG1.2, Cat# 50580993, 1:100) and APC anti-mouse Granzyme B (Clone QA16A02, Cat# 372214429, 1:100) were purchased from BioLegend (California, USA). APC-Cy7 anti-human CD45 antibody (Clone 2D1, Cat# 557833, 1:200) was obtained from BD Biosciences (California, USA). Cy5 goat anti rabbit IgG (Polyclonal, Cat# GB27303, 1: 500) and FITC goat anti rabbit IgG (Polyclonal, Cat# GB22303, 1: 200) was obtained from Servicebio (Wuhan, China).
Validation	All information about antibodies can be found on the respective antibody website:<br>FITC anti-mouse CD11b Antibody, https://www.biolegend.com/en-gb/products/fitc-anti-mouse-human-cd11b-antibody-347<br>PE anti-mouse Ly6G Antibody, https://www.biolegend.com/en-gb/products/pe-anti-mouse-ly-6g-antibody-4777?<br>APC anti-mouse F4/80 Antibody, https://www.biolegend.com/en-us/products/apc-anti-mouse-f4-80-antibody-4071<br>APC anti-mouse CD4 Antibody, https://www.biolegend.com/en-us/products/apc-anti-mouse-cd4-antibody-9224<br>FITC anti-mouse CD8 Antibody, https://www.biolegend.com/en-us/products/fitc-anti-mouse-cd8a-antibo]]dy-153<br>PE anti-mouse CD3 Antibody, https://www.biolegend.com/en-us/products/pe-anti-mouse-cd3-antibody-47<br>FITC anti-mouse CD45 Antibody, https://www.biolegend.com/en-us/products/fitc-anti-mouse-cd45-antibody-21256<br>APC/CY7anti-mouse CD45 Antibody,https://www.bdbiosciences.com/en-eu/products/reagents/flow-cytometry-reagents/research-reagents/single-color-antibodies-ruo/apc-cy-7-mouse-anti-human-cd45.557833?tab=product_details<br>APC anti-mouse NK-1.1 Antibody, https://www.biolegend.com/en-us/products/apc-anti-mouse-nk-11-antibody-19843<br>FITC anti-mouse CD86 Antibody, https://www.biolegend.com/en-us/products/fitc-anti-mouse-cd86-antibody-254<br>PE anti-mouse CD80 Antibody, https://www.biolegend.com/en-us/products/pe-anti-mouse-cd80-antibody-43<br>PE anti-mouse CD206 Antibody, https://www.biolegend.com/en-us/products/pe-anti-mouse-cd206-mmr-antibody-7424<br>PE anti-mouse FOXP3 Antibody, https://www.biolegend.com/en-gb/products/pe-anti-mouse-foxp3-antibody-4660<br>APC anti-mouse IFN-γ Antibody, https://www.biolegend.com/en-gb/products/apc-anti-mouse-ifn-gamma-antibody-993<br>APC anti-mouse GzmB Antibody, https://www.biolegend.com/en-gb/products/apc-anti-human-mouse-granzyme-b-recombinant-antibody-14429<br>APC anti-mouse CD11c Antibody,https://www.biolegend.com/ja-jp/products/apc-anti-mouse-cd11c-antibody-1813?GroupID=BLG11937<br>PerCP anti-mouse CD11b Antibody, https://www.biolegend.com/en-gb/products/percp-anti-mouse-human-cd11b-antibody-4315?<br>Cy5 goat anti rabbit IgG; https://www.service-bio.com/goodsdetail?id=4191<br>FITC goat anti rabbit IgG; https://www.service-bio.com/goodsdetail?id=259

Eukaryotic cell lines

Policy information about cell lines and Sex and Gender in Research

Cell line source(s)	NIH 3T3 cells (Cat# CRL-1658) were provided by Wuhan Service Biotechnology Co., Ltd., while murine colon cancer cells (CT26, Cat# CRL-2638), pancreatic cancer cells (Panc02, Cat# CRL-2553), luciferase-labeled mouse pancreatic cancer cells (Panc02-LUC, derived from Pan02, Cat# CRL-2553), and RAW 264.7 macrophages ((ATCC, Cat# TIB-71)were obtained from the American Type Culture Collection (ATCC). Special thanks are due to Professor Gan Lu from the College of Life Sciences and Technology, Huazhong University of Science and Technology (HUST) for the kind donation of the KPC cell line for this study.
Authentication	Each cell line was morphologically confirmed according to the information provided by the cell-source center, and the main Panc02, 4T1, CT26 , Panc02-luc, KPC, 3T3 and RAW cell line were authenticated with short tandem repeat (STR) analysis.
Mycoplasma contamination	All cell lines were confirmed to be mycoplasma-negative by DAPI staining.
Commonly misidentified lines<br>(See ICLAC register)	Panc02, 4T1, CT26 , Panc02-luc, 3T3 ,KPC, and RAW cell line were of stable origin and there was little possibility of misidentification.

Animals and other research organisms

Policy information about studies involving animals; ARRIVE guidelines recommended for reporting animal research, and Sex and Gender in Research

Laboratory animals	BALB/c female mice (BALB/cAnNCrl, 8-week-old weighing 18-20 g) and C57BL/6 female mice (C57BL/6NCrl, 8-week-old weighing 18-20 g) were purchased from Charles River Laboratory Animal Technology Co., Ltd. All mice were housed in an animal facility under constant environmental conditions (room temperature, 20-22°C, relative humidity, 30-70 %). All mice had access to food and water ad libitum.
Wild animals	The study did not involve wild animals.
Reporting on sex	The research results do not involve gender

Field-collected samples	The study did not involve samples collected from the field.
Ethics oversight	All animal experiments were approved by the animal experiment ethics committee of Huazhong University of Science and Technology (IACUC Number: S904).

Note that full information on the approval of the study protocol must also be provided in the manuscript.

Plants

Seed stocks	This study does not involve
Novel plant genotypes	This study does not involve
Authentication	This study does not involve

Flow Cytometry

Plots

Confirm that:

☒ The axis labels state the marker and fluorochrome used (e.g. CD4-FITC).

☒ The axis scales are clearly visible. Include numbers along axes only for bottom left plot of group (a 'group' is an analysis of identical markers).

☒ All plots are contour plots with outliers or pseudocolor plots.

☒ A numerical value for number of cells or percentage (with statistics) is provided.

Methodology

Sample preparation	Following tumor harvest, single-cell suspensions were prepared, washed with PBS, treated with erythrocyte lysis buffer to remove red blood cells, and resuspended in PBS for subsequent experimental use.
Instrument	CytoFLEX flow cytometer
Software	CytExpert (ver. 2.3.0.84) was used to collect and analyze the flow cytometry data.
Cell population abundance	After the cells were centrifuged, the pelleted cells were taken for flow cytometric analysis. With adjusting the gain and compensation, 10,000-60000 cells were taken from each group for analysis.
Gating strategy	The untreated cell group was taken, and the voltage was adjusted to make the intensity of FTIC and PI channels around 1000; the single-stained cell group was taken for compensation adjustment and deducting the mutual influence, finally experimental groups were carry out. If the intensity value was greater than 1000, positive signal was considered to exist.

☒ Tick this box to confirm that a figure exemplifying the gating strategy is provided in the Supplementary Information.
