# Supplementary material for: CD11b agonist GB1275 potentiates oncolytic bacterial therapy against pancreatic cancer in mouse tumor models
Source: Nat Commun. 2026 Aug 21;17:10739. doi: 10.1038/s41467-026-76823-7 (PMC13661619; doi:10.1038/s41467-026-76823-7)
Supplement: Supplementary file 3 [file 41467_2026_76823_MOESM3_ESM.pdf]

nature portfolio

Peer Review File

CD11b agonist GB1275 potentiates oncolytic bacterial therapy against pancreatic cancer in mouse tumor models

Corresponding Author: Professor Yuan-Di Zhao

This file contains all reviewer reports in order by version, followed by all author rebuttals in order by version.

Version 0:

Reviewer comments:

Reviewer #1

(Remarks to the Author)
This is an interesting body of work evaluating the potential use of bacteria in anti-tumor strategies. The biological data and approaches are comprehensive and well controlled. These studies generally demonstrate that a CD11b agonist, which has been shown to block inflammatory myeloid cell infiltration into tissues, can improve the efficacy of anti-tumor responses in mouse models of implanted pancreatic ductal adenocarcinoma.

In most studies, the biological effects observed are clear. Bacteria have a negative effect on tumor growth that is partially neutralized by invading myeloid cells. Administration of the CD11b agonist clearly improved the biological efficacy of the agonist.

However, the immune cell analysis in this work is inadequate. The authors should show their overall immune cell Facs gating scheme in the supplement. The Facs plots for tumor analysis throughout the manuscript are unusual and do not appear to have been properly gated on live cells and CD45+ cells; these plots do not show clearly discriminated populations and the percent of myeloid cells in PDAC tumors are uncharacteristically low for PDAC tumors. It does not appear that Facs analysis was performed by defining cells, single cells, live cells, CD45+ cells and then, then CD45+, then CD11b+, followed by gates for subpopulations Ly6C+, Ly6G+, F4/80. Also did the authors use FMOs (fluorescence minus one and single stains to set the gates?

The graphs of the percent positive cells in each analysis do not define what the percentage is based upon (live cells, CD45%,the current gate?)> Proper analysis would identify the percent positive cells as percent of live or percent of CD45+, etc. The percent of myeloid cells in PDAC tumors is quite low; these are tumors with significant macrophage infiltrate (up to 30% or more of the tumor).
The inadequate Facs analysis appears to underestimate of the number of myeloid cells in PDAC tumors and diminishes confidence in the accuracy of conclusions regarding immune responses in these studies.

In Figures 3E, F, G- the error bars of the graphs are quite large and there does not appear to be a significant difference in upon administration of GB1275. The sample size for this tissue assay is small (n=3) and variance is high for liver. This data should be improved.
In Figure 3H: The groups are mis-labeled as G0-G3 instead of G1-G4. The apparent decrease in CD11b+ subpopulations would be more credible if better Facs plots were shown. As shown, each population is a mere shoulder of the entire tumor populations with no granularity expected in a study about myeloid cell roles in immune therapy.

In Figure 7, there are nearly no T cells in the tumors and the data regarding increased T cell infiltration do not appear significant.

Ideally, the authors should use additional approaches to block myeloid cell entry into tumors to prevent oncolytic bacteria inactivation. Inhibitors of integrin a4b1, Pi3Kgamma, CCR2 are clear examples based on the myeloid cell literature.

Discussion regarding potential deleterious effects of bacteria as drugs should be included in the summary of this manuscript.

Reviewer #2

(Remarks to the Author)
The presented data support the hypothesis that targeting $CD11b^+$ innate immune cells with the agonist GB1275 can enhance oncolytic bacterial therapy. The data provide a strong foundation. However, the following comments are suggested to ensure clarity of interpretation:

•The manuscript states that GB1275 inhibits the infiltration of $CD11b^+$ cells. Please reference or briefly discuss all prior preclinical and clinical studies with GB1275, including details from any ongoing or completed clinical trials. For example, Panni et al., in Science Translational Medicine, demonstrated that a CD11b agonist (leukadherin-1, a precursor to GB1275) reprogrammed the immune microenvironment in pancreatic cancer models. Activating CD11b on myeloid cells led to their retention within the stroma, effectively reducing their infiltration into the tumor core. Another relevant study is a 2021 review in the Journal for ImmunoTherapy of Cancer (also by Panni et al.), which summarizes the preclinical rationale for GB1275 and explicitly states that it reduces macrophage and neutrophil migration. GB1275 has also been evaluated in clinical trials, notably the KEYNOTE-A36 trial (NCT04060342). This Phase 1/2 study combined GB1275 with pembrolizumab (a PD-1 inhibitor) in patients with advanced solid tumors. The rationale for this trial was based on preclinical evidence that GB1275 can modulate the immunosuppressive TME, specifically by reducing the presence of myeloid-derived suppressor cells (MDSCs) and tumor-associated macrophages (TAMs).
•Please confirm whether references 36–37 directly relate to the antitumor effects of bacteria, as implied in the current text.
•Given that the central hypothesis is based on intratumoral bacteria, it would be more convincing to directly show how GB1275 treatment leads to higher and more sustained bacterial presence over time compared to controls, using imaging or molecular methods (e.g., qPCR, 16S rRNA sequencing).
•Comparative analysis of bacterial clearance rates in bacteria-only control mice versus GB1275 combination groups should be presented.
•To substantiate the role of myeloid cells in bacterial clearance within tumors, additional functional experiments are recommended: a combinations of neutrophil and macrophage depletions (separately and combined). For Neutrophil depletion you can use an anti-Ly6G antibody (clone 1A8). For Macrophage depletion you can use either CSF1R inhibitor (e.g., PLX3397 or similar) or Clodronate liposomes. The readouts should include tumor CFU kinetics (e.g., at 12, 24, 48 h), and survival/Kaplan–Meier curves.
•While tumor volume data are presented, survival analysis for the combination therapy group would provide a more clinically relevant assessment of treatment efficacy.
•The introduction of liposomes and gemcitabine onto bacterial surfaces could potentially alter immune recognition. Although the study indicates that GB1275 and Lip@V are safe, further evaluation is warranted to determine whether liposome–bacteria conjugation triggers a different immune response compared to unmodified bacteria.
•The study suggests that increased bacterial load drives M1 macrophage polarization. It would be valuable to demonstrate that bacterial-derived pathogen-associated molecular patterns (PAMPs) are the primary mediators of this polarization in the presence of GB1275.
•A direct comparison with a control group exposed to an M1-polarizing agent (e.g., LPS + IFN-γ) but without bacteria could help isolate the contribution of bacterial PAMPs versus other immune-activating factors.

Reviewer #3

(Remarks to the Author)
I co-reviewed this manuscript with one of the reviewers who provided the listed reports. This is part of the Nature Communications initiative to facilitate training in peer review and to provide appropriate recognition for Early Career Researchers who co-review manuscripts.

Reviewer #4

(Remarks to the Author)
The manuscript presents an interesting and potentially impactful study exploring the use of oncolytic bacteria in combination with the CD11b-targeted agonist GB1275 to enhance anti-tumor immunity in PDAC. The topic is timely, and the study is generally well-conceived. However, several aspects of the experimental design, data presentation, and interpretation raise concerns about whether the data sufficiently support the authors' conclusions. Clarification and additional experimental evidence would substantially strengthen the manuscript.
Major Comments:
1. Only one commercially available murine PDAC cell line (Panc01) was used in this study. Although the authors included cell lines from other cancer types to show the generalizability of the GB1275-bacteria combination, this approach does not adequately address the heterogeneity within PDAC itself. Given that PDAC comprises distinct molecular and phenotypic subtypes (e.g., epithelial vs. mesenchymal), it would be more informative to include additional PDAC cell lines representing these subtypes. Since Panc01 is mesenchymal, including epithelial-subtype PDAC models would provide valuable insights into how tumor subtype–specific TMEs influence treatment efficacy.
2. The treatment regimen begins when tumors reach approximately 70 mm^3, which represents a very early stage of tumor development. In clinical settings, PDAC is typically treated at a more advanced stage. Since the TME and stromal composition differ markedly between early and late-stage tumors, the therapeutic response may vary significantly. The authors should include data from experiments initiated at later tumor stages to better simulate clinical conditions.
3. The authors claim that GB1275 synergizes with Lip@V-induced DNA damage to enhance IFNγ production and activate the cGAS–STING pathway. However, direct evidence demonstrating DNA damage upon Lip@V infection is missing.

Measuring DAMP levels would substantiate this claim. Furthermore, the Western blot images are of poor quality and make it difficult to assess whether a synergistic effect truly exists between Lip@V alone and the combination treatment. Improved image quality and clearer quantification are necessary.
4. GB1275 is described as an inhibitor of $CD11b^+$ cell infiltration; however, it also appears to promote M1 macrophage polarization (Figures 4N–O, S23). The underlying mechanism of this dual effect is not clearly explained. Additional mechanistic discussion or supporting data would help reconcile this observation.
5. The resolution of Figure 7H is too low to discern $CD8^+$ signals, making it difficult to validate the immunofluorescence findings and correlate them with the flow cytometry results shown in Figures 7I–K. Furthermore, the reported percentages of $IFN\gamma^+$ and $GZMB^+$ $CD8^+$ cells appear inconsistent with the total $CD3^+CD8^+$ population. The authors should clarify whether the percentages in Figures 7J–K represent proportions within the $CD3^+CD8^+$ subset or the total tumor cell population. Additionally, in the same figure, Ki67 staining does not appear entirely nuclear. Higher magnification images should be provided to confirm staining specificity and localization.
6. Some in vitro experiments, though technically sound, contribute limited mechanistic insight. For instance, using spheroid models to demonstrate the inhibitory effect of GB1275 on $CD11b^+$ cell infiltration may not accurately reflect in vivo conditions, as spheroids lack key components of the tumor microenvironment such as extracellular matrix architecture and vasculature. This section appears of limited value and could be condensed or omitted.

Minor Comments

The Results section contains extensive background information and data interpretation, which makes it difficult to follow the core findings. The authors should streamline this section to focus more on experimental observations.

Numerous grammatical and typographical errors are present throughout the manuscript. Careful proofreading is required to improve readability and professionalism.

Version 1:

Reviewer comments:

Reviewer #1

(Remarks to the Author)
The authors have satisfied my previous concerns in admirable fashion with one exception.

The authors stated they used inhibitors of CCR2 and PI3Kgamma as alternative methods of suppressing CD11b+ cell adhesion. However, their stated PI3Kγ inhibitor 3-Methyladenine is not a PI3Kγ inhibitor but rather a PI3kinase class III (Vps34) inhibitor, which inhibits autophagy. PI3K gamma is class I PI3K, which regulates adhesion, chemotaxis and transcription, while PI3kinase class III (Vps34) regulates sequestration of cytoplasmic material in lysosomes during autophagy.

The authors should correct the description of these data or eliminate the data altogether.

Reviewer #4

(Remarks to the Author)
The authors have revised the manuscript and addressed most of the concerns. However, one point remains: the response adds a KPC-derived PDAC model but does not fully address the concern regarding subtype heterogeneity. While Panc02 is assumed to be mesenchymal-like, no data are provided, especially for the KPC-derived model, to confirm their molecular or phenotypic subtypes. Without such characterization and without inclusion of epithelial-subtype PDAC models, it remains unclear whether the GB1275+Lip@V combination therapy is effective across the spectrum of PDAC heterogeneity. I recommend that the authors provide subtype characterization for their models and/or include epithelial-subtype PDAC models to strengthen the conclusions.

Reviewer #5

(Remarks to the Author)
The authors have carefully addressed the questions raised by Reviewer #2.

I only have two minor suggestions for clarification:

Please add the methods of drug delivery to the schematic in Supplementary Figure 13.
Please specify the type of cells analyzed in Supplementary Figure 16.

Version 2:

Reviewer comments:

Reviewer #1

(Remarks to the Author)
The authors have satisfied my previous concerns.

Reviewer #4

(Remarks to the Author)
The authors have revised the manuscript and addressed my concerns, I'm satisfied with this version.

Reviewer #5

(Remarks to the Author)
The authors have addressed all my comments and concerns.

Open Access This Peer Review File is licensed under a Creative Commons Attribution 4.0 International License, which permits use, sharing, adaptation, distribution and reproduction in any medium or format, as long as you give appropriate credit to the original author(s) and the source, provide a link to the Creative Commons license, and indicate if changes were made.
In cases where reviewers are anonymous, credit should be given to 'Anonymous Referee' and the source.
The images or other third party material in this Peer Review File are included in the article's Creative Commons license, unless indicated otherwise in a credit line to the material. If material is not included in the article's Creative Commons license and your intended use is not permitted by statutory regulation or exceeds the permitted use, you will need to obtain permission directly from the copyright holder.
To view a copy of this license, visit https://creativecommons.org/licenses/by/4.0/

Response to referees' comments

Reviewer #1:

This is an interesting body of work evaluating the potential use of bacteria in anti-tumor strategies. The biological data and approaches are comprehensive and well controlled. These studies generally demonstrate that a CD11b agonist, which has been shown to block inflammatory myeloid cell infiltration into tissues, can improve the efficacy of anti-tumor responses in mouse models of implanted pancreatic ductal adenocarcinoma. In most studies, the biological effects observed are clear. Bacteria have a negative effect on tumor growth that is partially neutralized by invading myeloid cells. Administration of the CD11b agonist clearly improved the biological efficacy of the agonist.

Response: We deeply appreciate your constructive comments on our experimental work. Your insights have immensely enriched the content of this article and expanded our intellectual horizons. In responses, uniformly use black font to mark the suggestions you provide, blue font to mark the reply, and red font to mark revisions made specifically to address the questions you raised.

1，However, the immune cell analysis in this work is inadequate. The authors should show their overall immune cell Facs gating scheme in the supplement. The Facs plots for tumor analysis throughout the manuscript are unusual and do not appear to have been properly gated on live cells and CD45+ cells; these plots do not show clearly discriminated populations and the percent of myeloid cells in PDAC tumors are uncharacteristically low for PDAC tumors. It does not appear that Facs analysis was performed by defining cells, single cells, live cells, CD45+ cells and then, then CD45+, then CD11b+, followed by gates for subpopulations Ly6C+, Ly6G+, F4/80. Also did the authors use FMOs (fluorescence minus one and single stains to set the gates?

Response: We sincerely appreciate the insightful inputs you have provided. For the flow cytometry analyses in the study that called for revision, we have strictly followed a standardized gating protocol: first, we sequentially defined the populations of total cells,

single cells, viable cells, and $CD45^{+}$ immune cells; subsequently, we further gated the $CD45^{+}$ cell population to identify $CD11b^{+}$ cells, and then subdivided the $CD11b^{+}$ cell subset based on surface markers including $Ly6G^{+}$ and $F4/80^{+}$. Both FMO controls and single-stain controls were implemented in all assays to guarantee the precision of gating demarcations. Corresponding gating strategies has been supplemented for each relevant main text figure and shown in **Supplementary Figures S38-S41**. Following standard gating, the proportion of myeloid cells in pancreatic cancer was significantly elevated. All revisions have been incorporated into the manuscript for your ease of review. Thank you again for your valuable suggestions !

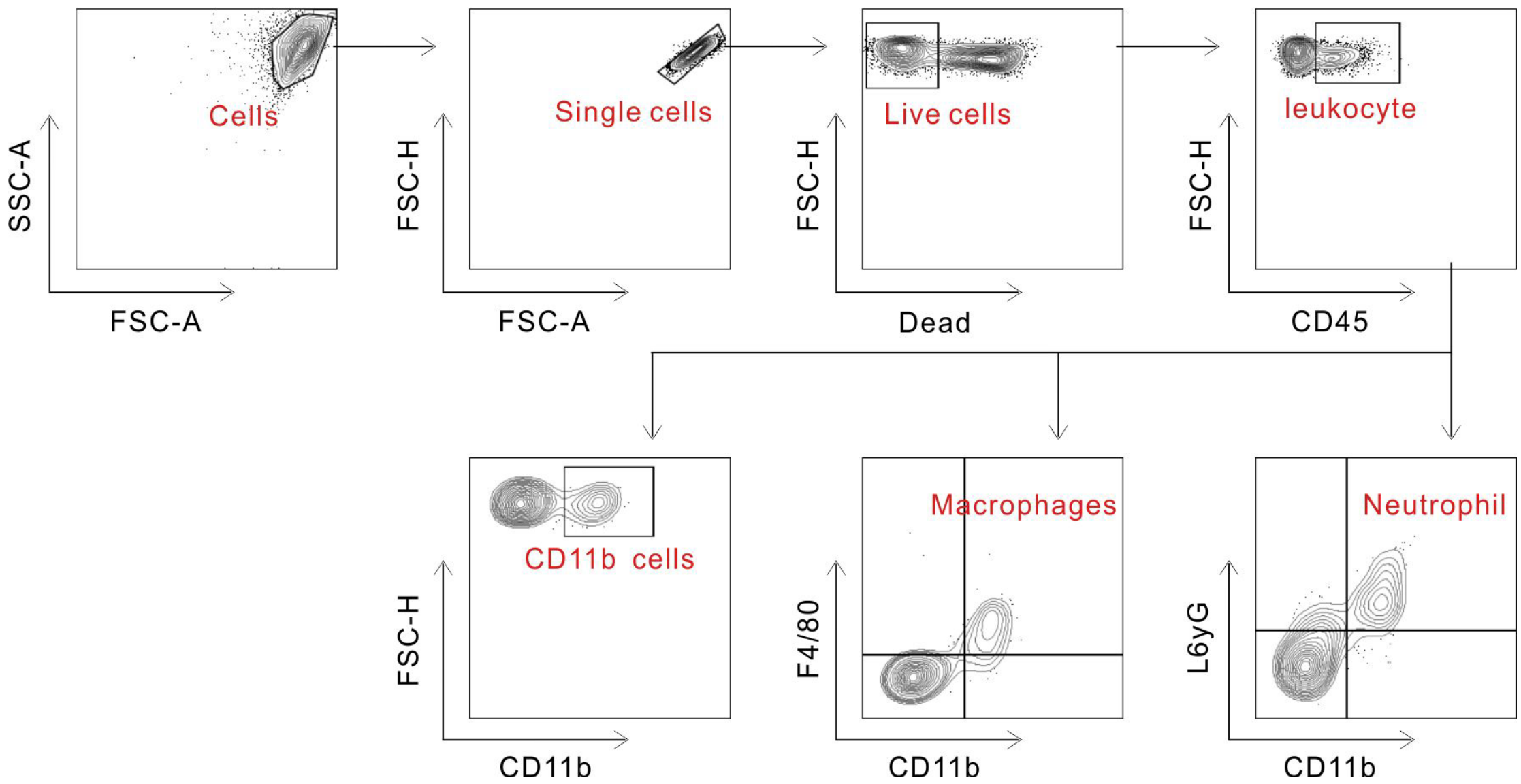

Supplementary Fig. 38 The gating process of flow cytometry analysis for $CD11b^{+}$ cells and their subsets (macrophages and neutrophils) in Fig 2 and Fig 3.

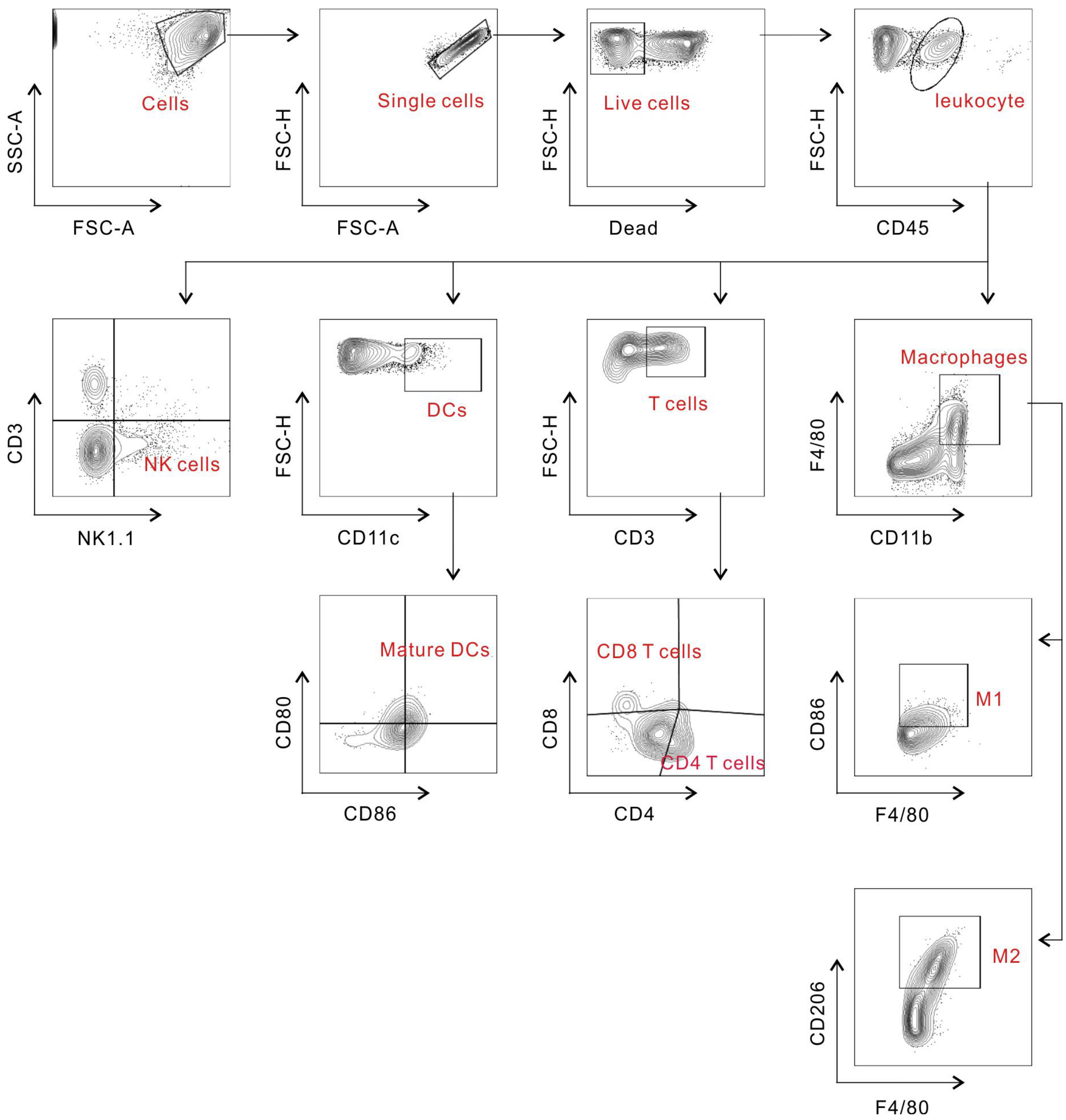

Supplementary Fig. 39 The gating process of flow cytometry analysis for $CD45^{+}$ immune cells and their subsets in Fig 4, including natural killer (NK) cells, mature dendritic cells (DCs), $CD8^{+}$ T cells, $CD4^{+}$ T cells, as well as macrophage subsets (M1-type macrophages and M2-type macrophages).

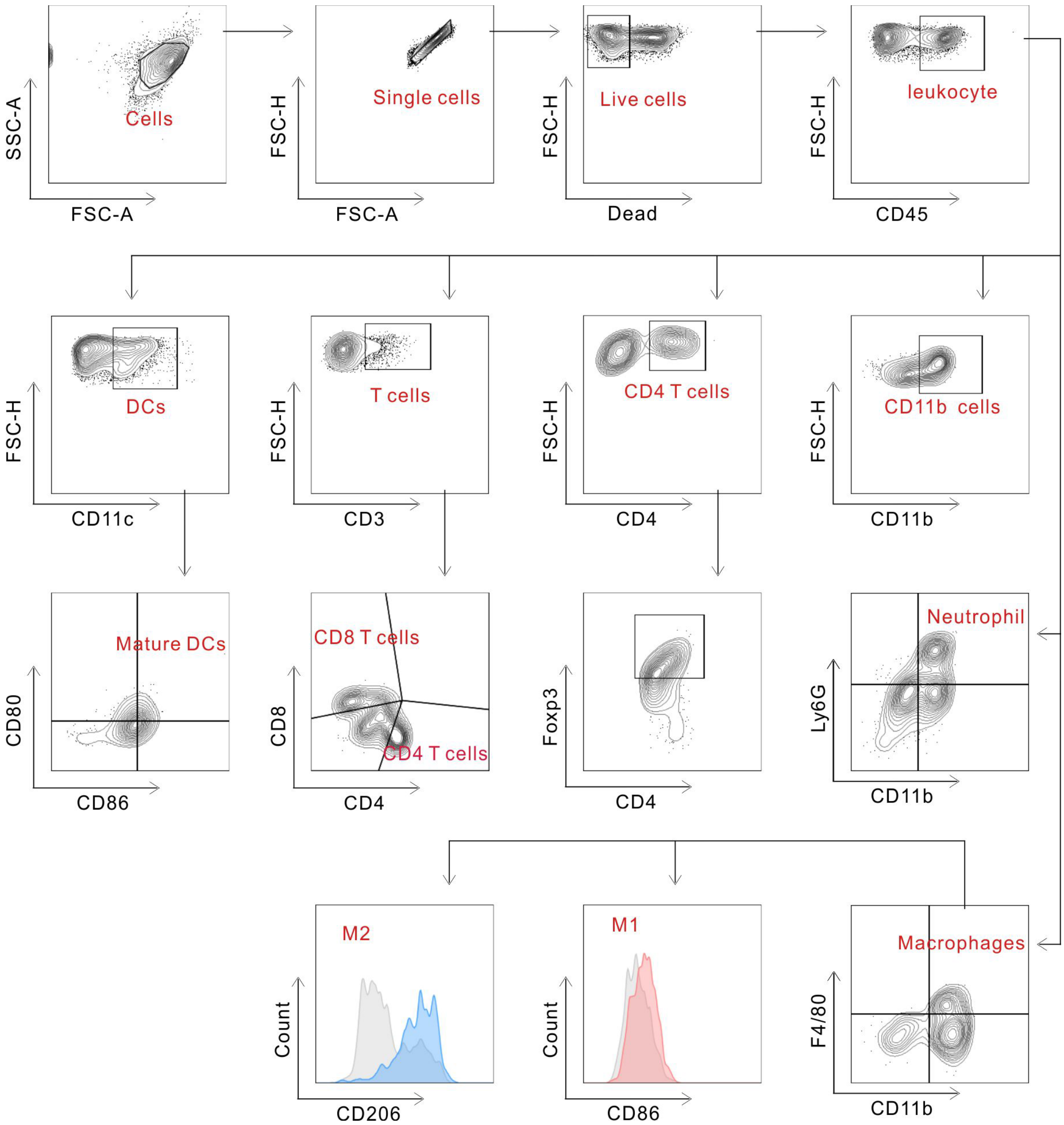

Supplementary Fig. 40 The gating process of flow cytometry analysis for $CD11b^+$ cells and their subsets (macrophages and neutrophils), DCs, $CD8^+$ T cells, $CD4^+$ T cells, regulatory T cells (Treg cells), as well as macrophage subsets (M1-type macrophages and M2-type macrophages) in Fig 6.

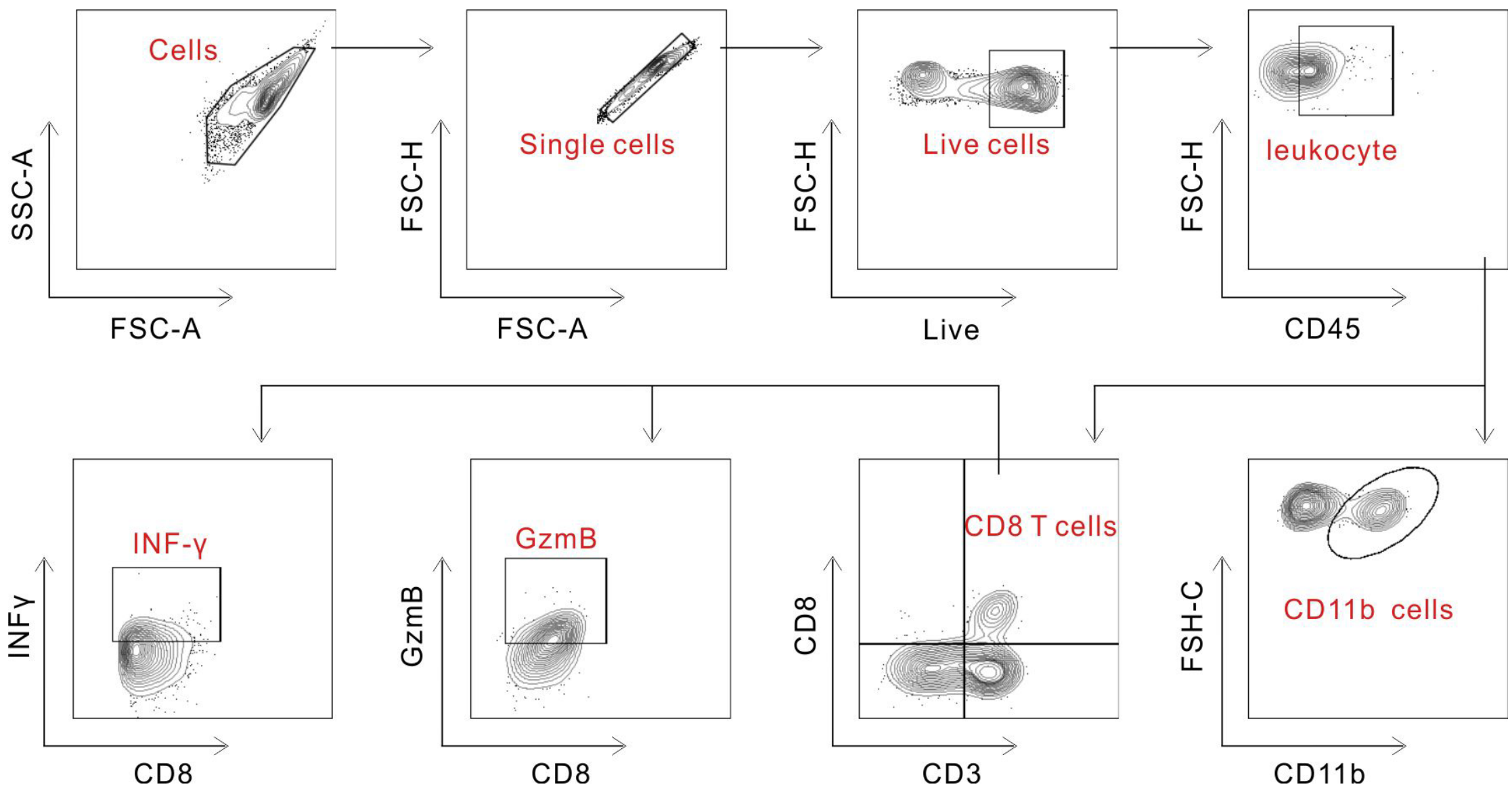

Supplementary Fig. 41 The gating process of flow cytometry analysis for CD11b+ cells, CD8+ T cells and their functional subsets (IFN-γ+CD8+ T cells, GzmB+CD8+ T cells) in Fig 7.

2，The graphs of the percent positive cells in each analysis do not define what the percentage is based upon (live cells, CD45%, the current gate?)> Proper analysis would identify the percent positive cells as percent of live or percent of CD45+, etc. The percent of myeloid cells in PDAC tumors is quite low; these are tumors with significant macrophage infiltrate (up to 30% or more of the tumor). The inadequate Facs analysis appears to underestimate of the number of myeloid cells in PDAC tumors and diminishes confidence in the accuracy of conclusions regarding immune responses in these studies.

Response: Thank you very much for your valuable suggestions! We have strictly followed the standardized gating protocol for flow cytometry and implemented both FMO controls and single-stain controls in all assays. While fully ensuring the accuracy of gating demarcations, we have further standardized the data analysis workflow and clarified the calculation basis and reference criteria for the percentage of positive cells. We have specified the nomenclature of each immune cell type at its first occurrence in the manuscript, and you can readily identify these contents in the manuscript. We

hereby provide the flow cytometry plots of various immune cell subsets and their corresponding quantitative analysis graphs in **Fig. 4** are provided for your review.

We fully concur with your observation that pancreatic ductal adenocarcinoma (PDAC) tumor tissues are typically characterized by extensive macrophage infiltration, which constitutes a core component of the immunosuppressive tumor microenvironment in PDAC. We sincerely thank you for providing the standardized flow cytometry gating protocol. We have re-performed the experimental analyses in strict accordance with this protocol, and our results demonstrated that following the revision of the gating strategy, the proportions of CD11b^{+} cells, macrophages, and neutrophils in Panc02 tumor tissues were significantly increased-thereby, ensuring the authenticity and reliability of the relevant experimental data in this study. The revisions have been incorporated into the manuscript.

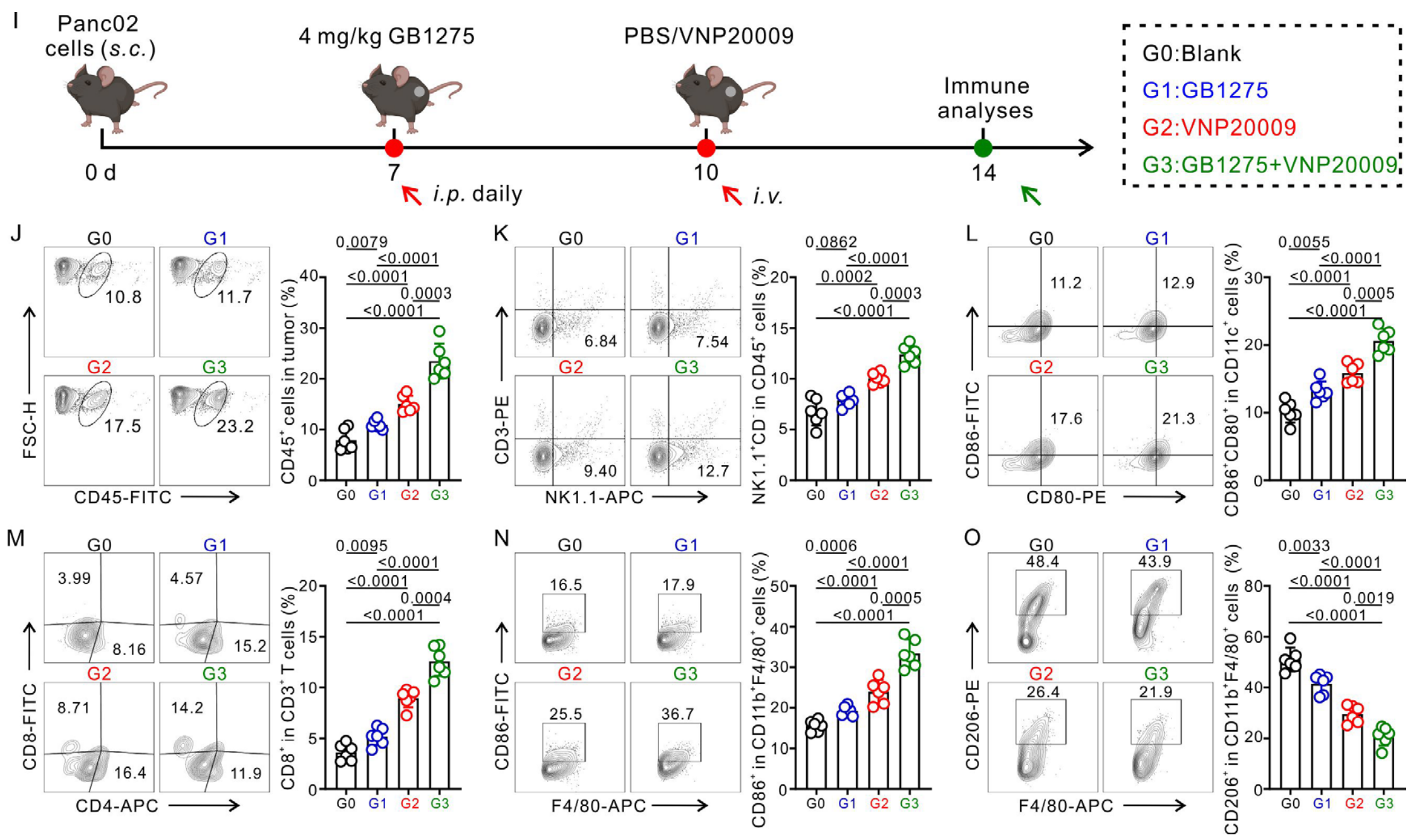

Fig. 4 **I** Schematic illustration of the experimental protocols for immune cell infiltration in tumor tissues after different treatments. *s.c.*, subcutaneous injection. *i.p.* daily, daily intraperitoneal injection. *i.v.,* intravenous injection. This figure was created with BioRender.com. **J-M** Flow cytometry analysis and the corresponding statistics of CD45^{+} cells, NK cells, DCs, and T cells infiltration in Panc02 tumor tissues after different treatments, n = 6 independent samples. Flow cytometry analysis and the corresponding statistics of the proportion of M1 and M2 cells in tumor tissue after

different treatments, *n* = 6 independent samples. The data are expressed as mean ± s.d. All replicates are biological, and statistical differences were calculated using two-tailed Student's t test.

3，In Figures 3E, F, G- the error bars of the graphs are quite large and there does not appear to be a significant difference in upon administration of GB1275. The sample size for this tissue assay is small (*n* = 3) and variance is high for liver. This data should be improved.

Response: Thank you very much for your valuable suggestions! We undertook re-verification and supplementary assays for the experimental data corresponding to **Fig. 3D and 3E**, with the sample size increased to *n* = 5. Following optimized data processing, the error bars in the corresponding plots were markedly reduced. We performed statistical significance analysis on the experimental data of Groups G2 and G3 at each time point, and directly labeled the statistical significance markers at the corresponding time points in the plots. The results demonstrated that all intergroup differences achieved statistical significance, and the variation trend of the experimental data was highly consistent with theoretical expectations. In addition, as shown in **Fig. 3F and 3G,** no significant differences were observed in the major organs (heart, liver, spleen, lung, and kidney) among all groups, consistent with our expectations. We further performed quantitative statistical analysis on the fluorescent signals at the tumor sites and labeled the analysis results at the corresponding positions in the relevant plots; these quantitative results were also consistent with our expectations.

These revisions have been incorporated into the manuscript and highlighted in red on **Page 11**, with the corresponding **Fig. 3** presented on **Page 52** and Supplementary **Fig. 13** provided in the Supporting Information.

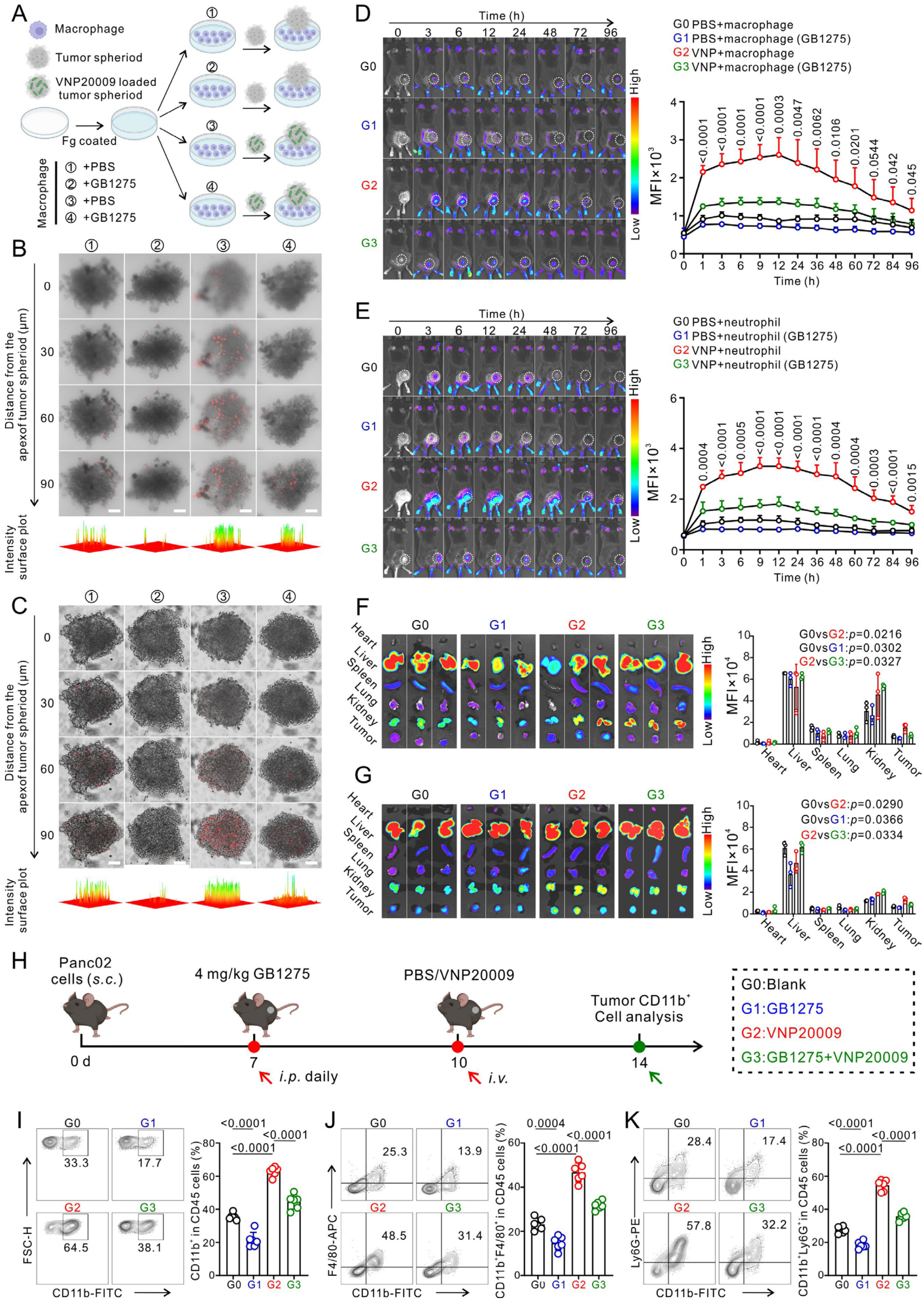

Fig. 3 GB1275 effectively inhibits the abnormal infiltration of CD11b⁺ innate immune cells induced by bacterial intratumoral colonization. A Schematic illustration of co-culture model establishing macrophage-tumor spheroids/bacterial-infiltrated tumor spheroids. Fg (fibrinogen) was used to enhance macrophage adhesion.

VNP2009-loaded tumor spheroids were constructed by incubating VNP20009 with Panc02 tumor spheroids for 3 h. This figure was created with BioRender.com. Confocal fluorescence imaging of tumor spheroids/bacterial-infiltrated tumor spheroids infiltration by **(B)** macrophages and **(C)** neutrophils after different treatments. The red fluorescence represents Cy5.5-labeled macrophages/Cy5.5-labeled neutrophils. The bottom panel is a 3D surface plot of Cy5.5-labeled macrophages/Cy5.5-labeled neutrophils. Scale bar: 20 μm. Representative whole-body fluorescence imaging dynamically monitored the tumor infiltration of $CD11b^+$ innate immune cells (including **(D)** macrophages and **(E)** neutrophils) subjected to different experimental treatments and labeled with Cy5.5 in Panc02 subcutaneous tumor models and bacterial-colonized tumor models. The yellow dotted circle represents the tumor sites. For the bacterial-colonized group, VNP20009 was administered via the tail vein 12 h prior to injection of fluorescently labeled cells. The line graph on the right shows the fluorescence intensity over time curve at the tumor sites of mice in each group from the corresponding whole-body fluorescence imaging, and the p values obtained in the chart represent the fluorescence statistical analysis of the tumor between G2 and G3, $n = 5$ independent samples. Fluorescence imaging of major organs and corresponding quantitative fluorescence statistics were performed at 48 h after in vivo injection of Cy5.5-labeled **(F)** macrophages and **(G)** neutrophils. The specific experimental treatments are consistent with the whole-body fluorescence imaging. The bar chart shows the fluorescence statistics of the corresponding organs, and the p values obtained in the chart represent the fluorescence statistical analysis of the tumor, $n = 3$ independent samples. **H** Schematic illustration of the experimental protocols for GB1275 to inhibit the abnormal infiltration of autologous $CD11b^+$ innate immune cells induced by bacterial intratumoral colonization. *s.c.*, subcutaneous injection. *i.p.* daily, daily intraperitoneal injection. *i.v.,* intravenous injection. This figure was created with BioRender.com. **I-K** Flow cytometry and corresponding statistics of the total $CD11b^+$ cells, macrophages and neutrophils in the tumor tissue after different treatments, $n = 6$ independent samples. The data are expressed as mean ± s.d. All replicates are biological, and statistical differences were calculated using two-tailed Student's t test.

4，In Figure 3H: The groups are mis-labeled as G0-G3 instead of G1-G4. The apparent decrease in CD11b$^+$ subpopulations would be more credible if better Facs plots were shown. As shown, each population is a mere shoulder of the entire tumor populations with no granularity expected in a study about myeloid cell roles in immune therapy.

Response: Thank you very much for your valuable suggestions! The designation of the Blank group has been uniformly standardized as Group G0 throughout the manuscript to address the prior naming inconsistency. We have provided higher-resolution versions of the original flow cytometry data plots, in which the expression trends of CD11b$^+$ cells and their subpopulations are more intuitive and readily observable. Furthermore, after strictly adhering to the flow cytometry gating criteria and standard protocols you proposed, the discrimination of each cell population in the plots has been markedly enhanced, yielding clearer, more intuitive results that are fully consistent with our experimental expectations.

These revisions have been incorporated into the manuscript and highlighted in red on **Page 11**, with the corresponding **Fig. 3** presented on **Page 52**.

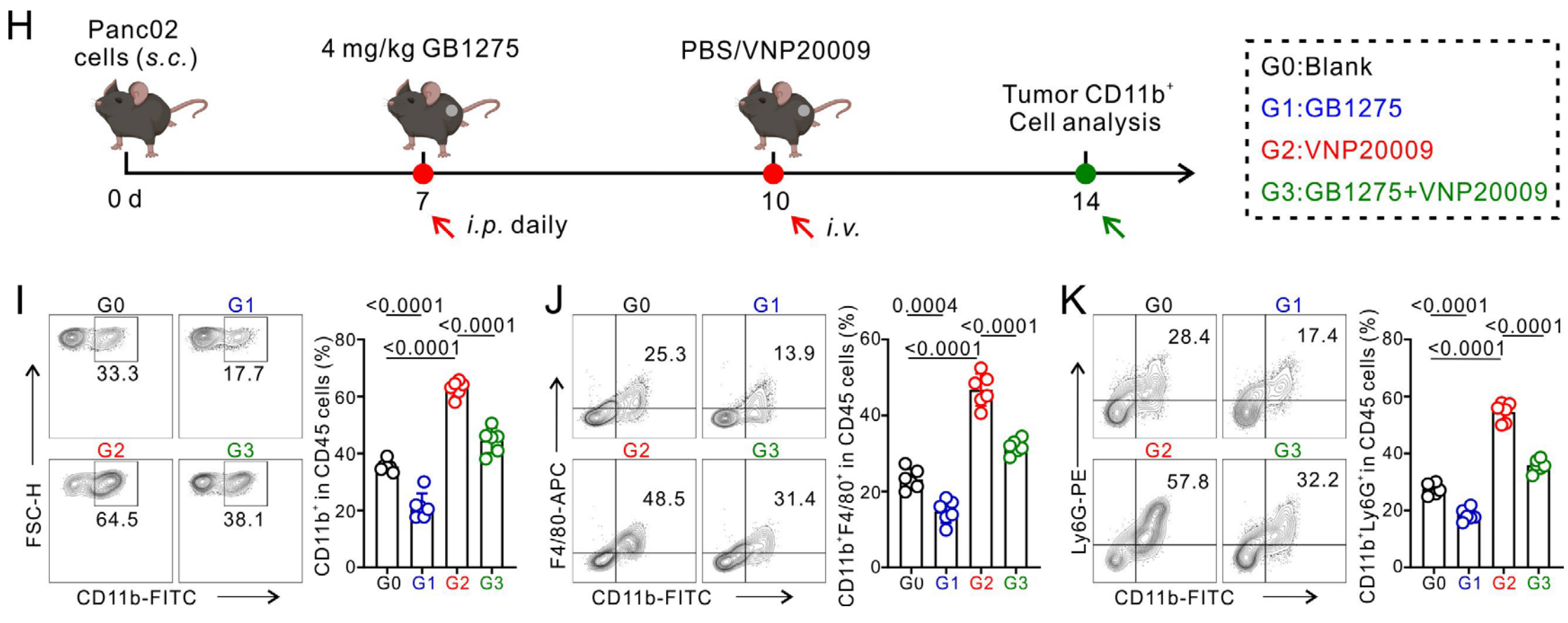

Fig. 3 **H** Schematic illustration of the experimental protocols for GB1275 inhibiting the abnormal infiltration of autologous CD11b$^+$ innate immune cells induced by bacterial intratumoral colonization. *s.c.*, subcutaneous injection. *i.p.* daily, daily intraperitoneal injection. *i.v.,* intravenous injection. This figure was created with BioRender.com. **I-K** Flow cytometry and corresponding statistics of the total CD11b$^+$ cells, macrophages, and neutrophils in the tumor tissue after different treatments, *n* = 6 independent samples.

The data are expressed as mean ± s.d. All replicates are biological, and statistical differences were calculated using two-tailed Student's t test.

5，In Figure 7, there are nearly no T cells in the tumors and the data regarding increased T cell infiltration do not appear significant.

Response: Thank you very much for your valuable suggestions! By strictly following the standardized flow cytometry gating protocol you proposed, we have markedly improved the discrimination of T cell subsets in the plots. The tumor infiltration rate of $CD8^+$ T cells has increased markedly from the original single-digit to double-digit percentages, demonstrating a significant rise in their tumor infiltration levels.

These revisions have been incorporated into the manuscript and highlighted in red on **Page 11**, with the corresponding **Fig. 7** presented on **Page** 62.

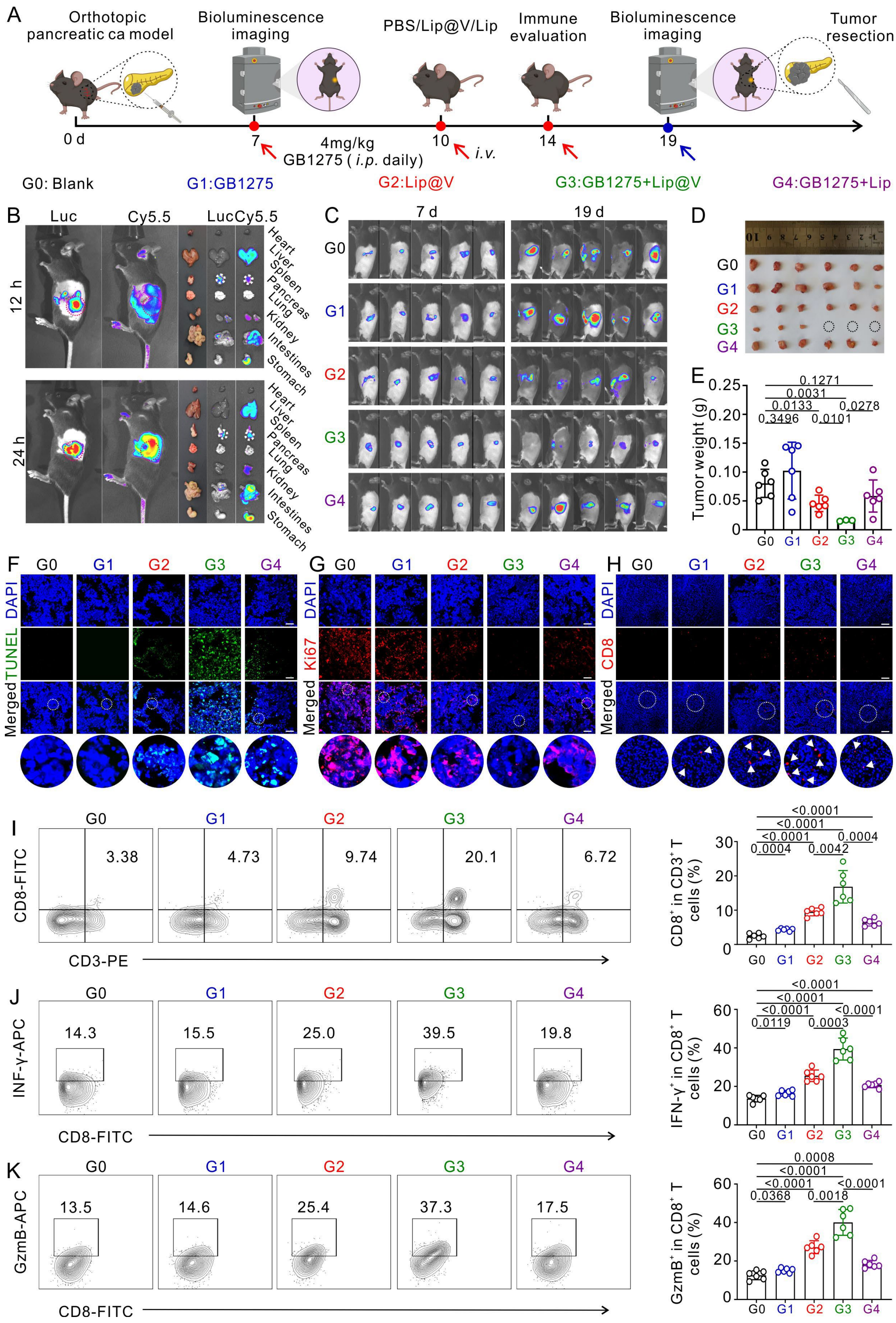

Fig.7 The combination of GB1275 with drug-loaded bacteria significantly inhibits the progression of orthotopic pancreatic cancer. **A** Schematic illustration of the experimental protocols for the combination therapy of GB1275 and drug-loaded

bacteria in the treatment of in situ pancreatic cancer. *s.c.*, subcutaneous injection. *i.p.* daily, daily intraperitoneal injection. *i.v.*, intravenous injection. This figure was reated with BioRender.com. **B** Representative fluorescence imaging combined with bioluminescence imaging co-localizes the enrichment of Lip@V in orthotopic pancreatic cancer at different time points post-administration. The figure includes whole-body fluorescence imaging and whole-body bioluminescence imaging of the in situ pancreatic cancer model, white light images of major organs and tissues, as well as fluorescence imaging and bioluminescence imaging. The fluorescent signals originate from Cy5.5-labeled Lip@V, while the bioluminescence imaging results from light emission produced by Panc02-luc cells catalyzing the substrate. **C** In vivo bioluminescence imaging of in situ pancreatic cancer after different treatments, $n = 5$ independent samples. **D, E** Photographs and tumor weight analysis of the harvested tumors from different treatment groups, $n = 6$ independent samples. **F, G** Representative images of TUNEL and Ki67 immunofluorescence staining of tumor tissues after different treatments. Scale bar: 200 μm. **H** Representative images of $CD8^+$ T cells immunofluorescence staining of tumor tissue after different treatments. To clearly localize the target cells, white triangular arrows were used in the figure to more explicitly highlight the T cells. Scale bar: 200 μm. **I-K** Flow cytometry analysis and the corresponding statistics of $CD8^+$ T cells, IFN-γ-secreting $CD8^+$ T cells, and GzmB-secreting $CD8^+$ T cells infiltration in tumor tissues after different treatments for 14 days, $n = 6$ independent samples. The data are expressed as mean ± s.d. All replicates are biological, and statistical differences were calculated using two-tailed Student's t test.

6，Ideally, the authors should use additional approaches to block myeloid cell entry into tumors to prevent oncolytic bacteria inactivation. Inhibitors of integrin a4β1, Pi3Kgamma, CCR2 are clear examples based on the myeloid cell literature.

Response: The integrin α4β1 inhibitor, phosphatidylinositol 3-kinase gamma (PI3Kγ) inhibitor, and C-C chemokine receptor type 2 (CCR2) inhibitor can all inhibit myeloid cell migration through their distinct mechanisms, and exhibit remarkable potential in

tumor growth inhibition when combined with oncolytic bacteria. This viewpoint is highly consistent with our research rationale. For this reason, we introduced the PI3Kγ inhibitor-3-Methyladenine and the CCR2 inhibitor-RS 504393, and additionally included clodronate liposomes (a macrophage depletor) and anti-Ly6G antibody (a neutrophil depletor) as experimental controls. By blocking myeloid cell infiltration into TME through different approaches, this study aimed to comparatively explore the regulatory effects of various reagents on the abundance and activity of intratumoral oncolytic bacteria (**Supplementary Fig. 13A**). We employed the LB plate counting method to quantitatively analyze intratumoral bacterial counts of each group, and calculated the relative survival rate of bacteria in each group with the PBS-treated group as the reference (**Supplementary Fig. 13B-F**). Results showed that, except RS 504393, 3-Methyladenine, clodronate liposomes, and anti-Ly6G antibody all increased VNP20009 counts in solid tumors to varying degrees. This indicates that intratumoral colonization of VNP20009 does not induce CCR2-dependent myeloid cell infiltration into tumors. Furthermore, we employed mCherry-fluorescently labeled VNP20009 for fluorescence imaging of tumor tissue sections, thereby further verifying directly and intuitively the enhancing effect of 3-Methyladenine, clodronate liposomes, and the anti-Ly6G antibody on intratumoral bacterial abundance and activity (**Supplementary Fig. 13 G, H**). These results confirm that 3-methyladenine, clodronate liposomes, and anti-Ly6G antibody can modulate abnormal myeloid cell infiltration and function via distinct mechanisms, and all exert potential tumor growth inhibition when combined with the oncolytic bacterium VNP20009. Notably, it is critical to clarify that GB1275 and 3-Methyladenine act as a myeloid cell migration inhibitor, whereas clodronate liposomes and the anti-Ly6G antibody are specific myeloid cell depletors-with fundamentally distinct modes of action.

These revisions have been incorporated into the manuscript and highlighted in red on **Page 14**, with the corresponding **Supplementary Fig. 13** provided in the Supporting Information.

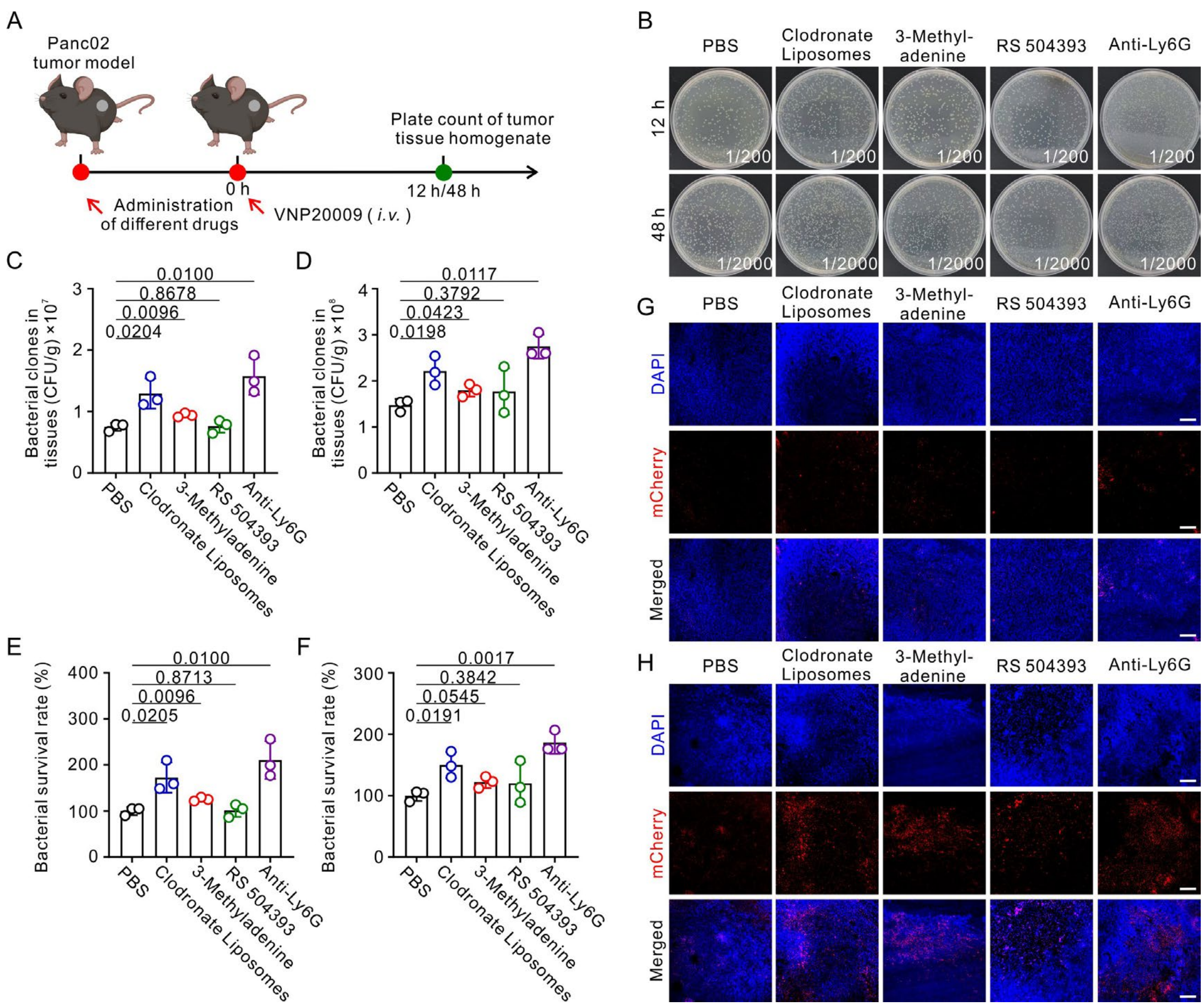

Supplementary Fig. 13 **A** Schematic illustration of the in vivo administration regimen of different drugs and VNP20009 in Panc02 subcutaneous tumor model. *i.p.* daily, daily intraperitoneal injection. *i.v.*, intravenous injection. This figure was created with BioRender.com. (**B-D**) Representative photographs and corresponding quantification of bacterial colonization in tumors obtained from Panc02 tumor-bearing mice at different time points after different treatments, n = 3 independent samples. Bacterial survival rates in tumor tissues of different groups at (**E**) 12 h and (**F**) 48 h, n = 3 independent samples. Representative images of mCherry-VNP20009 immunofluorescence staining of tumor tissues after different treatments at (**G**) 12 h and (**H**) 48 h. Scale bar: 200 μm. The data are expressed as mean ± s.d. All replicates are biological, and statistical differences were calculated using two-tailed Student's t test.

GB1275 was selected in this study primarily because it can enhance the anti-tumor immunotherapeutic efficacy of oncolytic bacteria by inhibiting myeloid cell migration, and additionally, key considerations were given to its targeted specificity for myeloid

cells, low toxicity, safety of in vivo administration, and synergistic effect with oncolytic bacteria in anti-tumor immunotherapy. Experiments confirmed that GB1275 exerts a targeted specific effect on CD11b-highly expressing cells, with no significant effect on CD11b-low or CD11b-negative cells. This high degree of cellular selectivity ensures that it does not interfere with the subsequent adaptive immune response. Furthermore, even at a high concentration of 50 μM, GB1275 exhibited negligible toxicity to normal cells, macrophages and neutrophils, which verified its excellent safety for in vivo application. This characteristic maintains the integrity of the host's antibacterial immune function and lays a critical foundation for the rapid recovery of the innate immune response after the end of treatment. In addition, previous studies have demonstrated that GB1275 can regulate the phenotypic polarization of tumor-associated macrophages (TAMs) by activating the expression of interferon genes, while oncolytic bacteria can also activate the STING signaling pathway via the release of cyclic dinucleotides (CDNs). We hypothesized that the two agents exert a synergistic effect through this mechanism, and this hypothesis has been experimentally verified in the present study. Collectively, compared with other myeloid cell migration inhibitors or depletors, GB1275 combines high safety with functional synergy, making it an optimal candidate reagent for improving the anti-tumor immunotherapeutic efficacy of drug-loaded bacteria.

7，Discussion regarding potential deleterious effects of bacteria as drugs should be included in the summary of this manuscript.

Response: The discussion on the "potential deleterious effects of bacteria when used as drugs" has been added in the summary. In addition, we have streamlined, optimized and refined this section, which has been incorporated into the conclusion section of the manuscript and highlighted in red on **Page 32.**

Nevertheless, bacteria-mediated anti-tumor immunotherapy requires a careful balance between efficacy and risks. As live biotherapeutics, bacteria carry the risks of systemic disseminated infection, inflammatory imbalance, virulence reversion

mutation of attenuated strains, and infection in immunocompromised patients. Subsequent research should focus on screening low-virulence and genetically stable bacterial strains, optimizing administration regimens, formulating personalized treatment plans and enhancing real-time toxicity monitoring throughout the treatment process, so as to maximize the protection of patient safety while exerting the anti-tumor effects of bacteria.

Reviewer #2:

The presented data support the hypothesis that targeting $CD11b^+$ innate immune cells with the agonist GB1275 can enhance oncolytic bacterial therapy. The data provide a strong foundation. However, the following comments are suggested to ensure clarity of interpretation:

Response: We deeply appreciate your constructive comments on our experimental work. Your insights have immensely enriched the content of this article and expanded our intellectual horizons. In responses, uniformly use black font to mark the suggestions you provide, blue font to mark the reply, and red font to mark revisions made specifically to address the questions you raised.

1, The manuscript states that GB1275 inhibits the infiltration of $CD11b^+$ cells. Please reference or briefly discuss all prior preclinical and clinical studies with GB1275, including details from any ongoing or completed clinical trials. For example, Panni et al., in Science Translational Medicine, demonstrated that a CD11b agonist (leukadherin-1, a precursor to GB1275) reprogrammed the immune microenvironment in pancreatic cancer models. Activating CD11b on myeloid cells led to their retention within the stroma, effectively reducing their infiltration into the tumor core. Another relevant study is a 2021 review in the Journal for ImmunoTherapy of Cancer (also by Panni et al.), which summarizes the preclinical rationale for GB1275 and explicitly states that it reduces macrophage and neutrophil migration. GB1275 has also been evaluated in clinical trials, notably the KEYNOTE-A36 trial (NCT04060342). This Phase1/2study combined GB1275 with pembrolizumab (a PD-1 inhibitor) in patients with advanced solid tumors. The rationale for this trial was based on preclinical evidence that GB1275 can modulate the immunosuppressive TME, specifically by reducing the presence of myeloid-derived suppressor cells (MDSCs) and tumor-associated macrophages (TAMs).

Response: Thank you very much for your valuable suggestions! The detailed discussion regarding the preclinical and clinical studies of GB1275 has added in the introduction

section of the manuscript and highlighted in red on **Page 5.**

Early CD11b-targeting strategies have primarily focused on inhibiting its function, yet they often face severe dose challenges. In contrast, GB1275, a novel oral small-molecule allosteric agonist of CD11b, exerts its therapeutic effects by stabilizing the active state of CD11b, and this activation process is independent of integrin saturation. This unique agonistic mechanism enables GB1275 to potentially circumvent the limitations of early strategies and provides a new direction for mitigating dose-limiting toxicity. In a study published in *Science Signaling*, Miguel et al. first reported a strategy of inhibiting leukocyte recruitment by activating CD11b/CD18 with a small-molecule agonist. This agonist was named leukadherin-1, the lead compound subsequently developed into GB1275. These compounds increase the level of CD11b/CD18-dependent cell adhesion in transfected cells and primary human and mouse neutrophils, thereby reducing chemotaxis and transendothelial migration. Studies in a mouse model of experimental nephritis have demonstrated that GB1275 exerts a marked protective effect on renal function. In a study published in *Nature Communications*, Schmid et al. repurposed GB1275 for tumor therapy by activating CD11b to drive the polarization of proinflammatory macrophages and inhibit tumor growth in mouse models bearing murine and human cancers. Later, a study by Panni et al. published in *Science Translational Medicine* showed that GB1275 can reprogram the immune microenvironment in pancreatic cancer models; activation of CD11b on the surface of myeloid cells sequesters these cells within the stromal compartment, thereby effectively abrogating their infiltration into the tumor core. In addition, Panni et al. also published a relevant review in the *Journal for Immunotherapy of Cancer* in 2021, which synthesized the preclinical evidence supporting GB1275 and clearly established its ability to inhibit the migration of macrophages and neutrophils. Recently, DeNardo et al. elucidated the precise mechanistic basis for the anti-pancreatic cancer effect of GB1275: they confirmed that GB1275 can reduce the infiltration of myeloid cells into tumor sites, and further found that GB1275 reshapes the phenotype of tumor-associated macrophages (TAMs) via concurrent inhibition of NF-κB signaling and activation of interferon gene expression. They also established GB1275's potential for combinatorial

therapy with different treatment modalities, further broadening its therapeutic applications in tumor therapy. To date, GB1275 has advanced to clinical trials (Clinical Trial ID: NCT04060342), and the trial is designed on the basis of preclinical evidence demonstrating that GB1275 can reprogram the immunosuppressive tumor microenvironment (TME) by depleting myeloid-derived suppressor cells (MDSCs) and TAMs. This first-in-human clinical trial of GB1275 features dose escalation for three regimens: GB1275 monotherapy (Regimen A), GB1275 plus pembrolizumab (Regimen B), and GB1275 plus nab-paclitaxel and gemcitabine (Regimen C), followed by a Phase 2 expansion cohort enrolling patients with newly diagnosed metastatic pancreatic adenocarcinoma, microsatellite-stable (MSS) colorectal cancer, and PD-L1-positive gastric/gastroesophageal junction (GEJ) cancer. Preliminary clinical data have demonstrated minimal to mild treatment-related toxicities across the investigated treatment regimens, and pharmacokinetic (PK) profiles support twice-daily (BID) administration, with dose escalation currently ongoing. Collectively, these preclinical and clinical findings indicate that GB1275 is a highly promising therapeutic candidate for combinatorial use with oncolytic bacteria. On this basis, the present study investigated the combination of GB1275 with the oncolytic bacterium VNP20009 to explore their synergistic anti-tumor efficacy in pancreatic cancer therapy.

2, Please confirm whether references 36-37 directly relate to the anti-tumor effects of bacteria, as implied in the current text.

Response: Thank you very much for your valuable suggestions! The original References 36-37 were primarily used to elucidate the critical roles of neutrophils and macrophages in antibacterial defense within the innate immune system, thus laying a theoretical foundation for defining their anti-infective immune functions. After colonizing tumors, oncolytic bacteria potently elicit the host innate immune response, which in turn recruit neutrophils and macrophages to the tumor microenvironment. Given the central roles of these two cell types in orchestrating antibacterial defense in vivo, we postulated that they may play pivotal regulatory roles in bacteria-mediated

anti-tumor immunotherapy. To further strengthen the theoretical underpinnings for this hypothesis and to better align with the core focus of the present study, we have reorganized the order of the references and updated the original References 36-37 to new References 37-38.

These revisions have been incorporated into the manuscript and highlighted in red **on Page 47.**

37. Volker B. et al. Neutrophil Extracellular Traps Kill Bacteria. *Science* 303, 1532-1535 (2004).

This study demonstrated that neutrophils possess not only the well-characterized antibacterial mechanism of phagocytosing and eradicating bacteria through the fusion of antimicrobial granules with phagosomes, but also release granule proteins and chromatin following activation; these components collectively form extracellular fibers that can sequester both Gram-positive and Gram-negative bacteria, termed neutrophil extracellular traps (NETs). NETs, in addition to detoxifying bacterial virulence factors and directly eradicating bacteria, are also abundantly present in two models of acute inflammation: experimental shigellosis and spontaneous acute appendicitis in humans. They constitute a form of innate immune response that sequesters microorganisms to prevent their systemic dissemination and mediates detoxification and bacterial clearance by maintaining a high local concentration of antimicrobial effectors.

38. An H. R. et al. Splenic red pulp macrophages eliminate the liver-resistant Streptococcus pneumoniae from the blood circulation of mice. *Sci. Adv.* 11, eadq6399 (2025).

This study demonstrated that splenic red pulp macrophages are the primary cells responsible for clearing liver-resistant *Streptococcus pneumoniae* from the murine bloodstream: they precisely recognize and sequester these bacteria through specific surface receptors, then mediate highly efficient bacterial eradication via phagolysosome fusion, which releases antimicrobial molecules and induces an oxidative burst, a function that is irreplaceable by other immune cell populations. The study also

definitively identifies the spleen as a hepatic "backup immune organ" that slows blood flow through the specialized vascular architecture of the red pulp, thereby creating a specialized microenvironment for macrophage effector function; the spleen and liver therefore form a dual defensive barrier against bacterial pathogens in the systemic circulation. Furthermore, this finding provides a mechanistic explanation for the heightened susceptibility of asplenic patients to *Streptococcus pneumoniae* infection, thus laying a theoretical foundation for infection prophylaxis in this patient cohort (e.g., the implementation of optimized vaccination regimens).

3, Given that the central hypothesis is based on intratumoral bacteria, it would be more convincing to directly show how GB1275 treatment leads to higher and more sustained bacterial presence over time compared to controls, using imaging or molecular methods (e.g., qPCR, 16S rRNA sequencing).

Response: Thank you very much for your valuable suggestions! In this study, we initially only quantified the intratumoral bacterial load via the LB agar plate colony-forming unit (CFU) counting assay. To further strengthen the robustness and persuasiveness of our study’s conclusions, we have further supplemented an additional fluorescent imaging assay to cross-validate the relevant experimental results. In this assay, mCherry-fluorescently labeled VNP20009 was used instead of wild-type VNP20009 in the assay. Coupled with immunofluorescence staining of tumor tissue sections, we further confirmed directly and intuitively that GB1275 treatment significantly increased bacterial colonization abundance in tumor tissues.

These revisions have been incorporated into the manuscript and highlighted in red on **Page 13**, with the corresponding **Supplementary Fig. 12** provided in the Supporting Information.

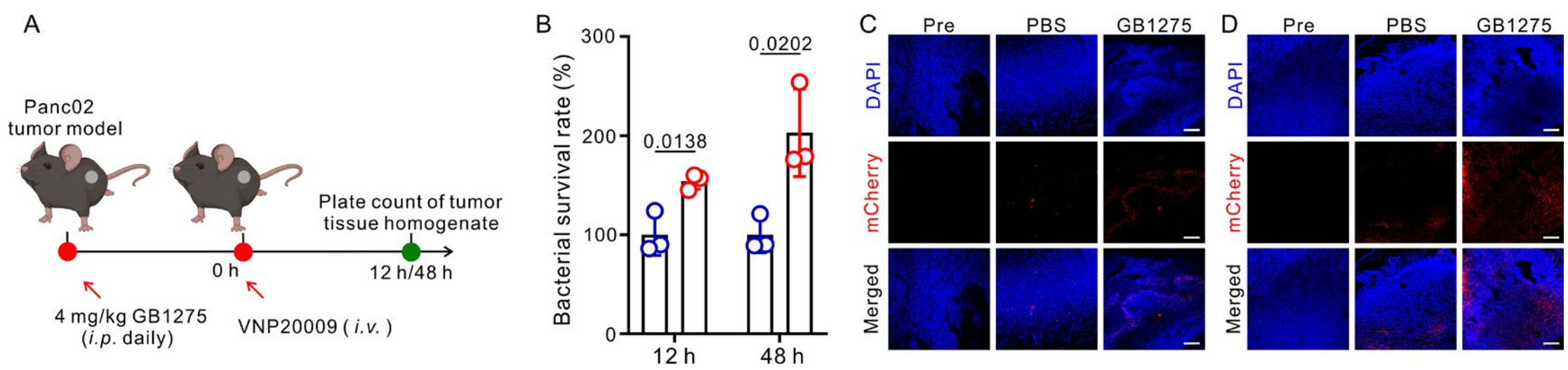

Supplementary Fig. 12 **A** Schematic illustration of the in vivo administration regimen of GB1275 and VNP20009 in Panc02 subcutaneous tumor model (Fig. 4E), with an equal volume of PBS as the control. *i.p.* daily, daily intraperitoneal injection. *i.v.*, intravenous injection. This figure was created with BioRender.com. **B** Bacterial survival rates in tumor tissues of different groups at 12 h and 48 h, n = 3 independent samples. Representative images of mCherry-VNP20009 immunofluorescence staining of tumor tissue after different treatments at (**C**) 12 h and (**D**) 48 h. Scale bar: 200 μm. The data are expressed as mean ± s.d. All replicates are biological, and statistical differences were calculated using two-tailed Student's t test.

4, Comparative analysis of bacterial clearance rates in bacteria-only control mice versus GB1275 combination groups should be presented.

Response: Thank you very much for your valuable suggestions! Original **Figure 4** only presented the bacterial load in tumor tissues of mice from the bacterial monotherapy group and the GB1275 combination treatment group, and did not further calculate the bacterial clearance rate. Upon careful evaluation, we consider that the bacterial survival rate, as an indicator, can more directly and intuitively reflect the effect of GB1275 in promoting intratumoral bacterial colonization and survival in comparison to the clearance rate. Therefore, we have calculated and supplemented the bacterial survival rate data at the 12 h and 48 h time points based on this rationale.

These revisions have been incorporated into the manuscript and highlighted in red on **Page 13**, with the corresponding **Supplementary Fig. 12** provided in the Supporting Information.

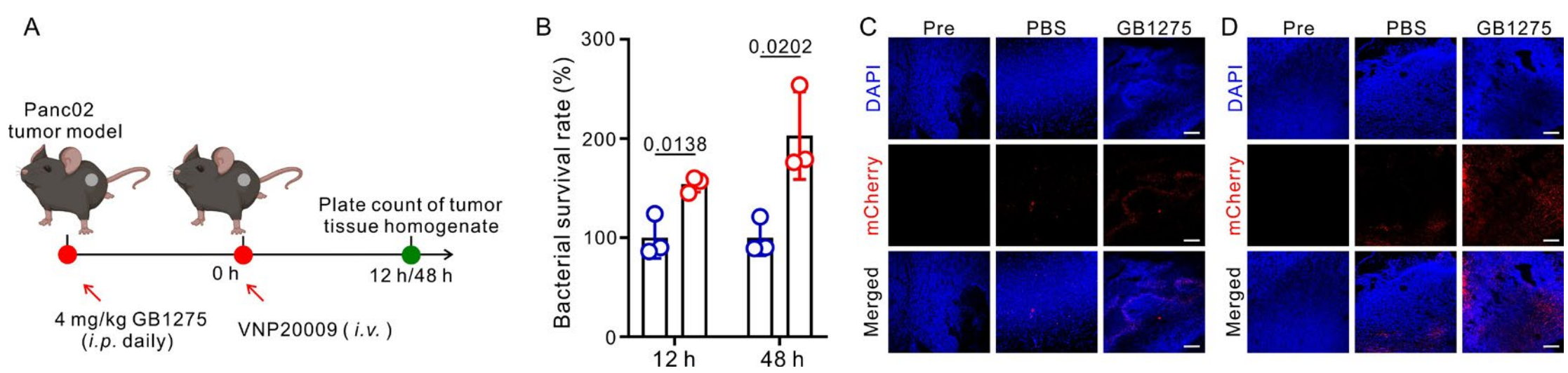

Supplementary Fig. 12 **A** Schematic illustration of the in vivo administration regimen of GB1275 and VNP20009 in Panc02 subcutaneous tumor model (Fig. 4E), with an

equal volume of PBS as the control. *i.p.* daily, daily intraperitoneal injection. *i.v.*, intravenous injection. This figure was created with BioRender.com. **B** Bacterial survival rates in tumor tissues of different groups at 12 h and 48 h, $n = 3$ independent samples. Representative images of mCherry-VNP20009 immunofluorescence staining of tumor tissue after different treatments at (**C**) 12 h and (**D**) 48 h. Scale bar: 200 μm. The data are expressed as mean ± s.d. All replicates are biological, and statistical differences were calculated using two-tailed Student's t test.

5, To substantiate the role of myeloid cells in bacterial clearance within tumors, additional functional experiments are recommended: Combinations of neutrophil and macrophage depletions (separately and combined). For Neutrophil depletion you can use an anti-Ly6G antibody (clone 1A8). For Macrophage depletion you can use either CSF1R inhibitor (e.g., PLX3397 or similar) or Clodronate liposomes. The readouts should include tumor CFU kinetics (e.g., at 12, 24, 48 h), and survival/Kaplan-Meier curves.

Response: Thank you very much for your valuable suggestions! The integrin-targeted macrophage depleter and neutrophil depleter you mentioned can deplete and inhibit innate immune cells through their respective unique mechanisms, and they exhibit significant potential in inhibiting tumor growth when combined with oncolytic bacteria-a viewpoint that is highly consistent with the research rationale of our study. To this end, we additionally introduced clodronate liposomes (a macrophage depleter) and anti-Ly6G antibody (a neutrophil depleter); we also included the PI3Kγ inhibitor 3-Methyladenine and the CCR2 inhibitor RS 504393 (both myeloid cell migration inhibitors) as controls. By blocking myeloid cell infiltration into TME through different approaches, this study aimed to comparatively explore the regulatory effects of various reagents on the abundance and activity of intratumoral oncolytic bacteria (**Supplementary Fig. 13A**). We employed the LB plate counting method to quantitatively analyze intratumoral bacterial counts of each group, and calculated the relative survival rate of bacteria in each group with the PBS-treated group as the reference **(Supplementary Fig. 13B-F)**. Results showed that, except RS 504393, 3-

Methyladenine, clodronate liposomes, and anti-Ly6G antibody all increased VNP20009 counts in solid tumors to varying degrees. This indicates that intratumoral colonization of VNP20009 does not induce CCR2-dependent myeloid cell infiltration into tumors. Furthermore, we employed mCherry-fluorescently labeled VNP20009 for fluorescence imaging of tumor tissue sections, thereby further verifying directly and intuitively the enhancing effect of 3-Methyladenine, clodronate liposomes, and the anti-Ly6G antibody on intratumoral bacterial abundance and activity (**Supplementary Fig. 13 G, H**). These results confirm that 3-methyladenine, clodronate liposomes, and anti-Ly6G antibody can modulate abnormal myeloid cell infiltration and function via distinct mechanisms, and all exert potential tumor growth inhibition when combined with the oncolytic bacterium VNP20009. Notably, it is critical to clarify that GB1275 and 3-Methyladenine act as a myeloid cell migration inhibitor, whereas clodronate liposomes and the anti-Ly6G antibody are specific myeloid cell depletors-with fundamentally distinct modes of action.

These revisions have been incorporated into the manuscript and highlighted in red on **Page 14**, with the corresponding **Supplementary Fig. 13** provided in the Supporting Information.

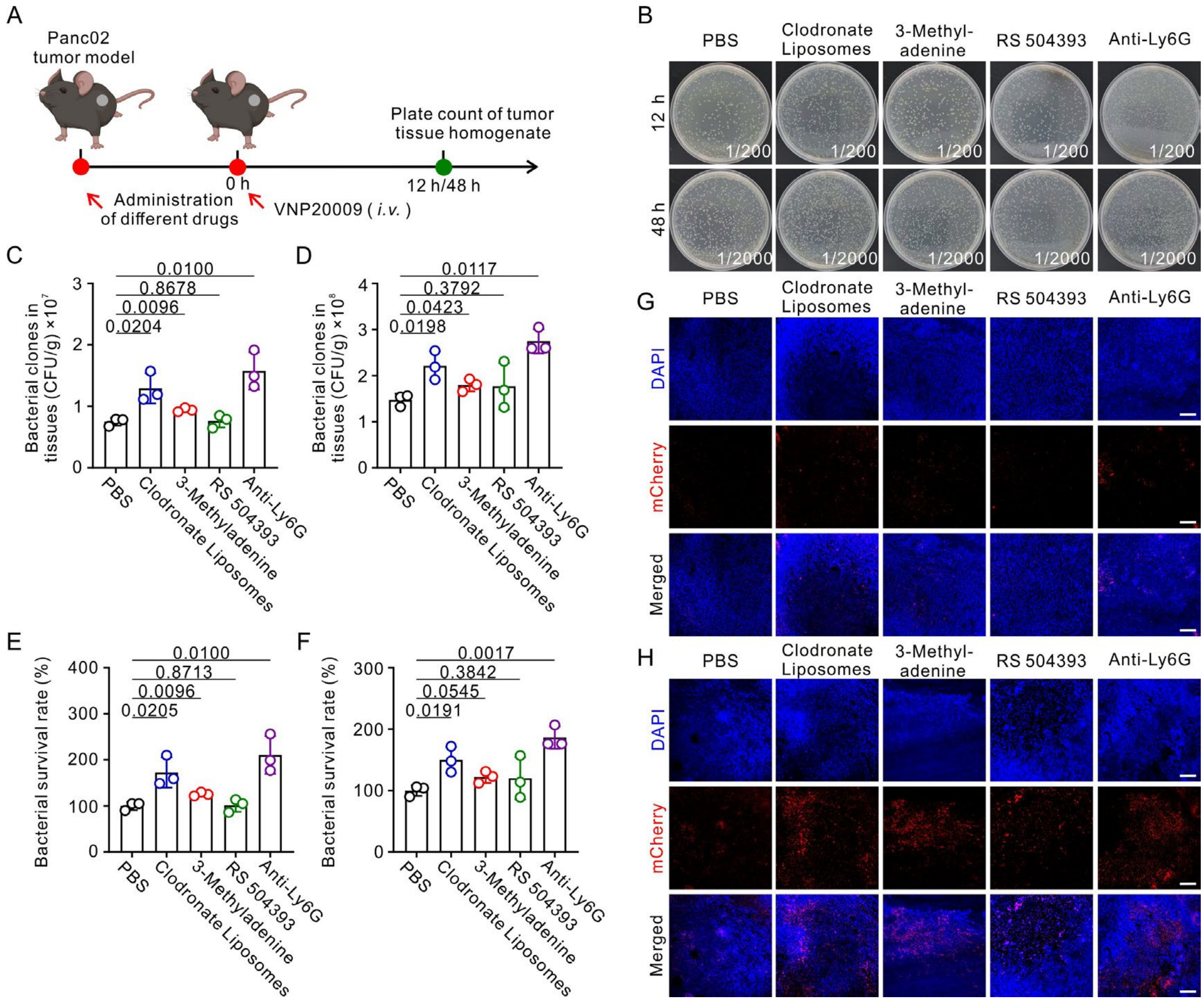

Supplementary Fig. 13 A Schematic illustration of the in vivo administration regimen of different drugs and VNP20009 in Panc02 subcutaneous tumor model. i.p. daily, daily intraperitoneal injection. i.v., intravenous injection. This figure was created with BioRender.com. (B-D) Representative photographs and corresponding quantification of bacterial colonization in tumors obtained from Panc02 tumor-bearing mice at different time points after different treatments, *n* = 3 independent samples. Bacterial survival rates in tumor tissues of different groups at (E) 12 h and (F) 48 h, *n* = 3 independent samples. Representative images of mCherry-VNP20009 immunofluorescence staining of tumor tissues after different treatments at (G) 12 h and (H) 48 h. Scale bar: 200 μm. The data are expressed as mean ± s.d. All replicates are biological, and statistical differences were calculated using two-tailed Student's t test.

On this basis, we further investigated the effects of combining GB1275, 3-Methyladenine, clodronate liposomes or anti-Ly6G antibody with VNP20009 on the survival time of tumor-bearing mice (**Supplementary Fig. 14**). Results showed that

although clodronate liposomes and anti-Ly6G antibody effectively increased intratumoral bacterial colonization, their systemic and prolonged depletion of neutrophils and macrophages during the treatment and detection periods resulted in the death of individual mice when the tumor volume was still below 1500 mm^3. Based on the experimental context, these deaths were most likely attributed to bacterial infection. In contrast, the combination regimens of 3-Methyladenine and GB1275-both belonging to myeloid cell migration inhibitors-exhibited superior safety. Meanwhile, survival data revealed that the GB1275 combination group achieved significantly better therapeutic efficacy than the 3-Methyladenine combination group in terms of survival outcomes. This efficacy difference may be attributed to GB1275's broad-spectrum targeted inhibition of abnormal myeloid cell migration, which enables a more comprehensive regulatory range. Additionally, GB1275 itself can exert anti-tumor effects by polarizing TAMs toward a pro-inflammatory phenotype, thereby exerting a more thorough effect in protecting intratumoral bacteria and ameliorating the tumor immune microenvironment.

These revisions have been incorporated into the manuscript and highlighted in red on **Page 15**, with the corresponding **Supplementary Fig. 14** provided in the Supporting Information.

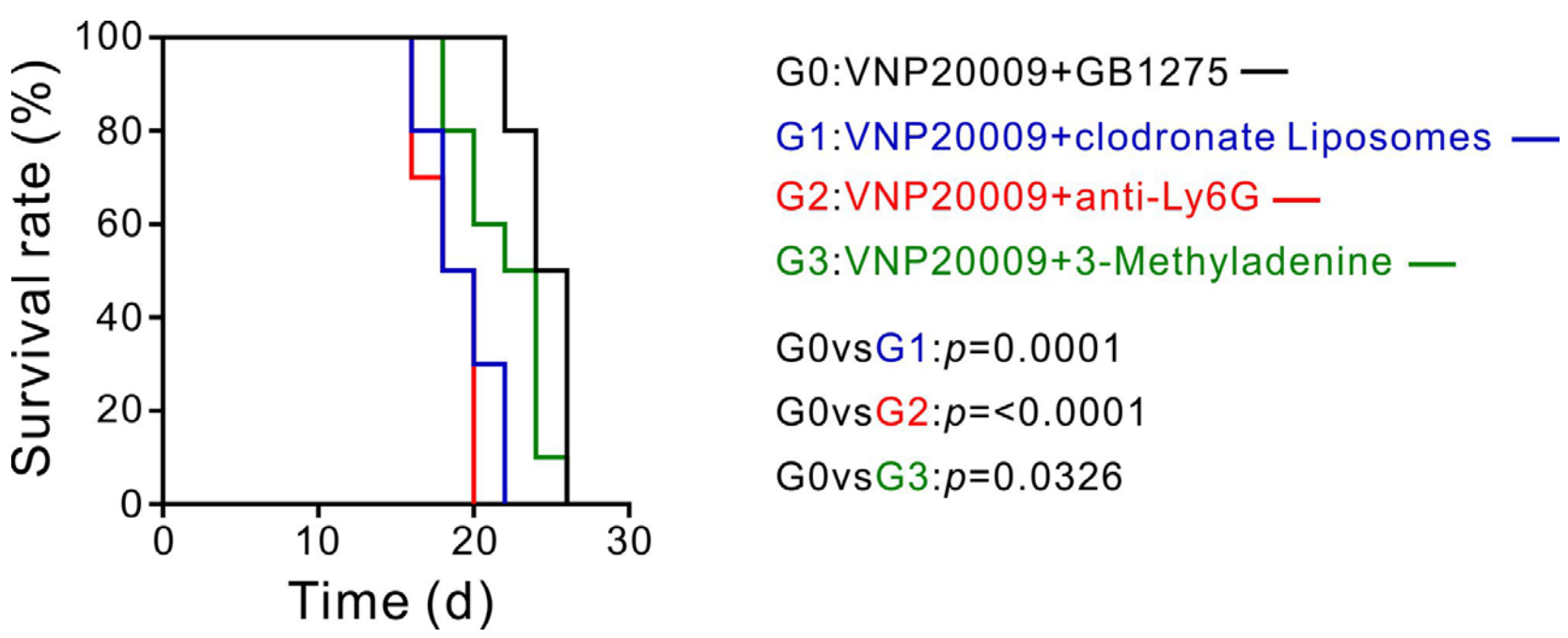

Supplementary Fig. 14 Monitoring of survival time of mice after different treatments.

6, While tumor volume data are presented, survival analysis for the combination therapy group would provide a more clinically relevant assessment of treatment efficacy.

Response: Thank you very much for your valuable suggestions! We fully agree that survival analysis plays a crucial role in enhancing the clinical relevance of research. Tumor volume only reflects the short-term tumor suppressive effect, while survival data better demonstrate the improvement value of treatment on the long-term prognosis of tumor-bearing models. Pancreatic cancer is characterized by a short survival time, and immunotherapy provides a new direction for prolonging the survival of patients. To this end, we investigated the effects of different treatment combinations on the survival of tumor-bearing mice and completed the statistical analysis. The experimental results showed that the combination therapy group significantly prolonged the survival time of mice compared with other control groups.

These revisions have been incorporated into the manuscript and highlighted in red on **Page 23**, with the corresponding **Supplementary Fig. 22** provided in the Supporting Information.

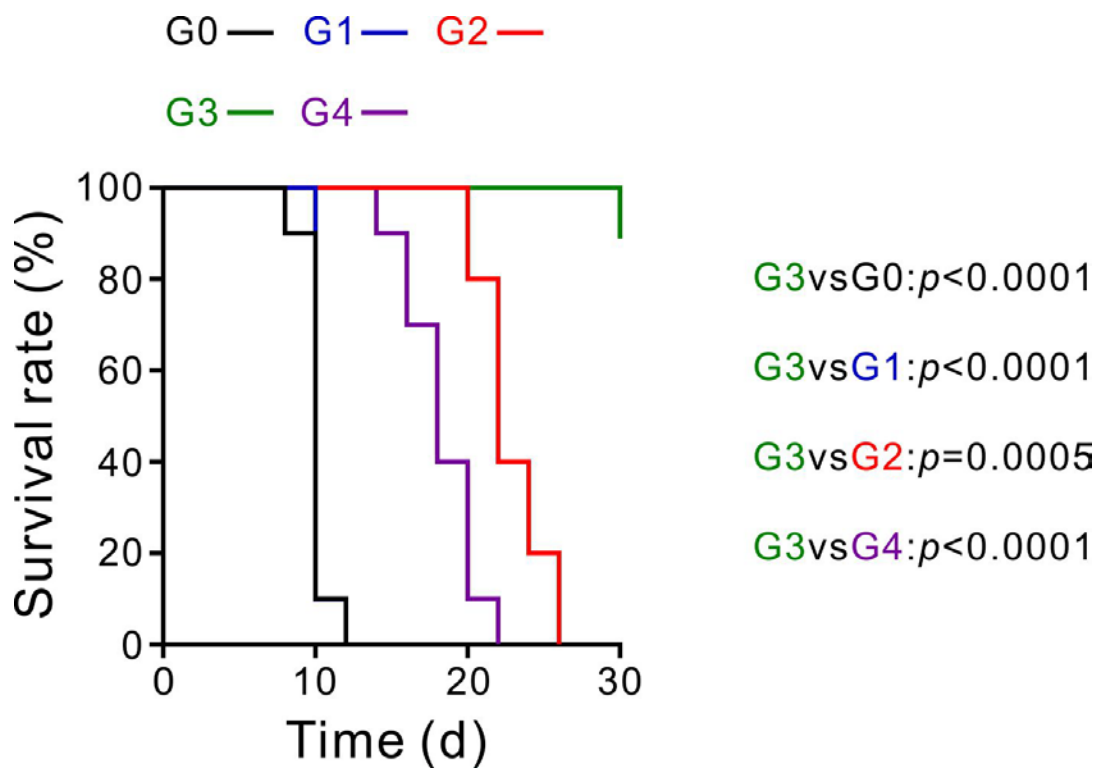

Supplementary Fig. 22 Survival curves of mice after different treatments.

7, The introduction of liposomes and gemcitabine onto bacterial surfaces could potentially alter immune recognition. Although the study indicates that GB1275 and Lip@V are safe, further evaluation is warranted to determine whether liposome–bacteria conjugation triggers a different immune response compared to unmodified bacteria.

Response: Thank you very much for your valuable suggestions! Although liposomal

modification had no adverse effect on the viability or tumor-targeting capacity of VNP20009, the anti-tumor efficacy of such bacteria is highly dependent on their ability to activate the anti-tumor immune system. Thus, compared with unmodified parental VNP20009, liposome-bacterium conjugates are likely to elicit distinct anti-tumor immune responses. In this study, using PBS as the negative control, we focused on the innate immune system-characterized by rapid and robust responses to investigate whether modification with empty liposomes would affect the intratumoral immune activation ability of bacteria (eLip@V). Experimental results showed that liposome modification exerted no significant effect on the intratumoral innate immune responses induced by bacteria (**Supplementary Fig. S19**). This is possible that the GSH-responsive liposomes used in our study can undergo cleavage to release gemcitabine in response to high levels of GSH overexpressed in TME. These liposomes undergo site-specific lysis at the tumor sites without affecting bacterial activity or interfering with the physiological functions and immune effects. These results lay a solid experimental foundation for verifying the feasibility of in vivo anti-tumor immunotherapy based on the combination of GB1275+Lip@V.

These revisions have been incorporated into the manuscript and highlighted in red **on Page 21**, with the corresponding **Supplementary Fig. 19** provided in the Supporting Information.

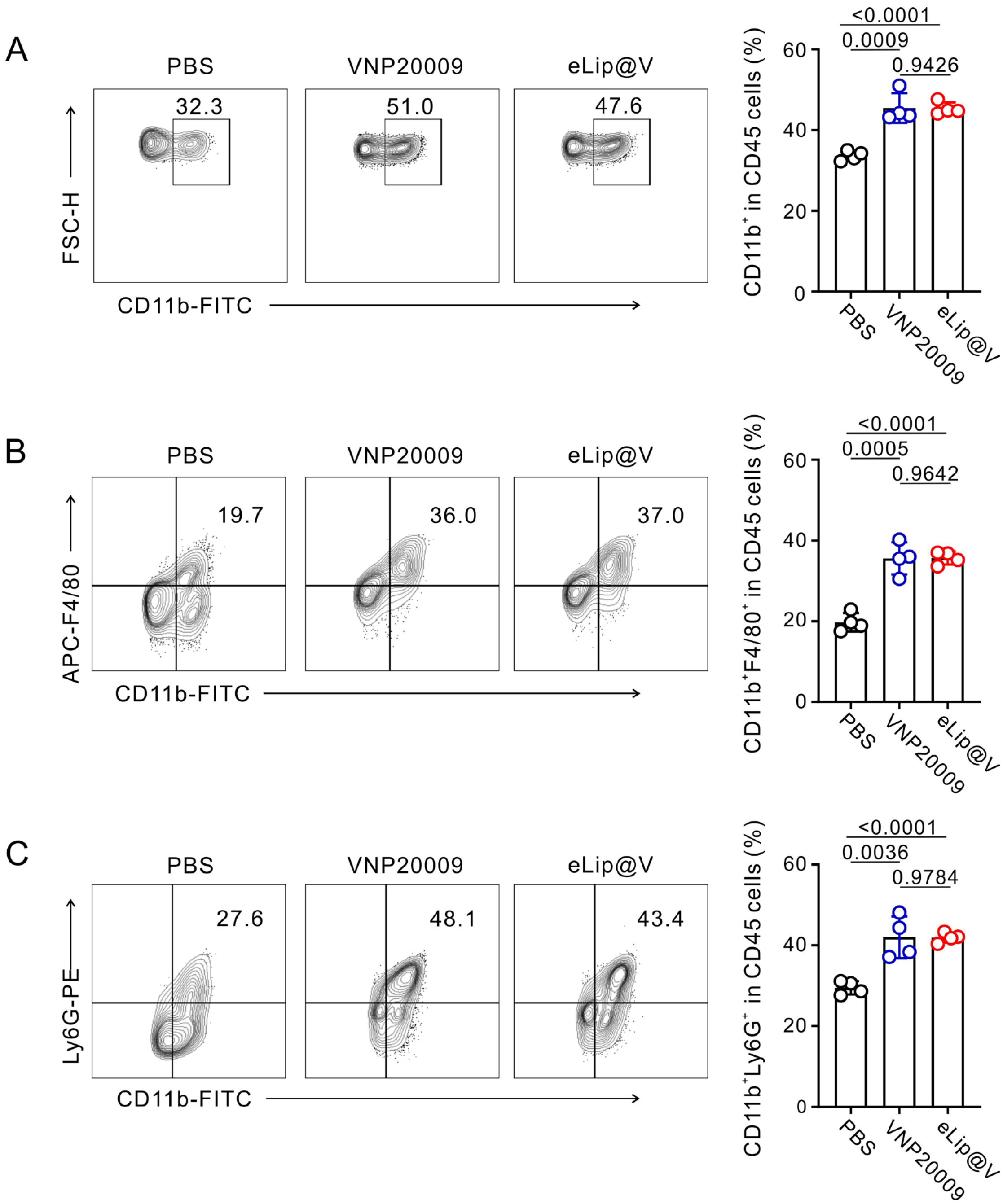

Supplementary Fig. 19 Flow cytometry analysis and corresponding statistics of (A) CD11b+ cells, (B) macrophages and (C) neutrophils infiltration in tumor tissues after different treatments, $n = 4$ independent samples. The data are expressed as mean ± s.d. All replicates are biological, and statistical differences were calculated using two-tailed Student's t test.

8, The study suggests that increased bacterial load drives M1 macrophage polarization.

It would be valuable to demonstrate that bacterial-derived pathogen-associated molecular patterns (PAMPs) are the primary mediators of this polarization in the presence of GB1275.

Response: We greatly appreciate your valuable suggestion. It is crucial to identify the primary mediators during the process of M2 macrophage polarization toward the M1 phenotype mediated by GB1275 in combination with bacteria. While conducting the verification experiment for this suggestion, we have simultaneously performed the relevant experiments corresponding to your next proposal. We compared the macrophage polarization potential of GB1275, LPS, VNP20009, and their different combinations in vitro, with LPS-a classical inducer of macrophage polarization-as the positive control. The results showed that LPS and VNP20009 exhibited significantly stronger macrophage polarization potential than GB1275 (**Supplementary Fig. 16**). Although LPS and VNP20009 showed no significant difference in their ability to induce M1 macrophage polarization, this observation confirms that LPS and bacterial pathogen-associated molecular patterns (PAMPs) exert comparable effects on M1 macrophage polarization. Taken together, these findings further validate the remarkable synergistic efficacy of the GB1275+VNP20009 combination in reversing the immunosuppressive TME, by modulating macrophage polarization and enhancing anti-tumor immune responses.

These revisions have been incorporated into the manuscript and highlighted in red on **Page 21**, with the corresponding **Supplementary Fig. 16** provided in the Supporting Information.

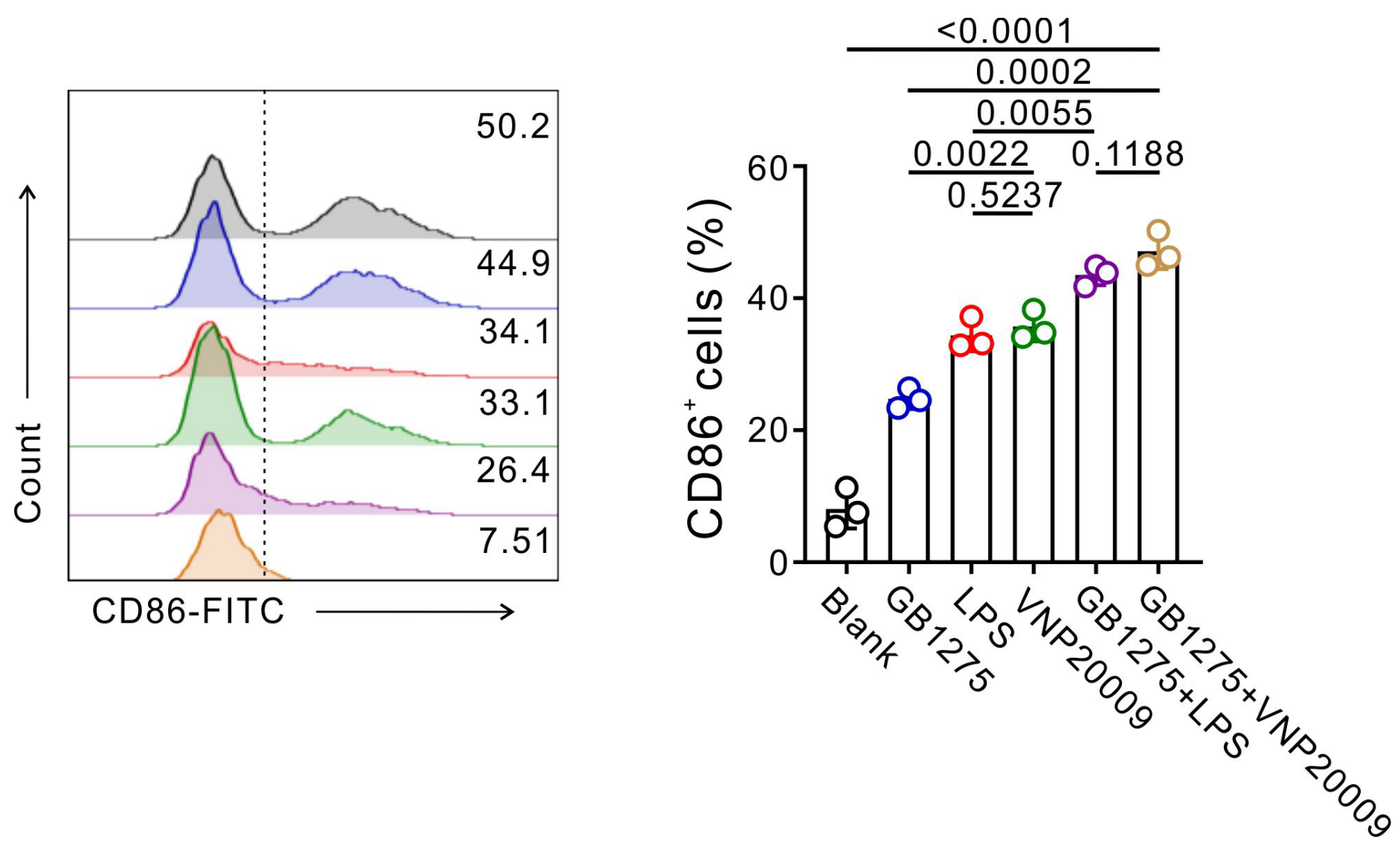

Supplementary Fig. 16 Flow cytometry analysis and corresponding statistics of $CD86^+$ expression levels after different treatments, n = 3 independent samples. The data are expressed as mean ± s.d. All replicates are biological, and statistical differences were calculated using two-tailed Student's t test.

9, A direct comparison with a control group exposed to an M1-polarizing agent (e.g., LPS+IFN-γ) but without bacteria could help isolate the contribution of bacterial PAMPs versus other immune-activating factors.

Response: Thank you very much for your valuable suggestions! It is crucial to establish a control group treated with M1 polarization reagent to evaluate the ability of VNP20009 to induce the polarization of M2 macrophages. We compared the macrophage polarization potential of GB1275, LPS, VNP20009, and their different combinations in vitro, with LPS-a classical inducer of macrophage polarization-as the positive control. The results showed that LPS and VNP20009 exhibited significantly stronger macrophage polarization potential than GB1275 (**Supplementary Fig. 16**). Although LPS and VNP20009 showed no significant difference in their ability to induce M1 macrophage polarization, this observation confirms that LPS and bacterial pathogen-associated molecular patterns (PAMPs) exert comparable effects on M1 macrophage polarization. Taken together, these findings further validate the remarkable synergistic efficacy of the GB1275+VNP20009 combination in reversing the immunosuppressive TME, by modulating macrophage polarization and enhancing anti-

tumor immune responses.

These revisions have been incorporated into the manuscript on **Page 21**, with the corresponding **Supplementary Fig. 16** provided in the Supporting Information for your review.

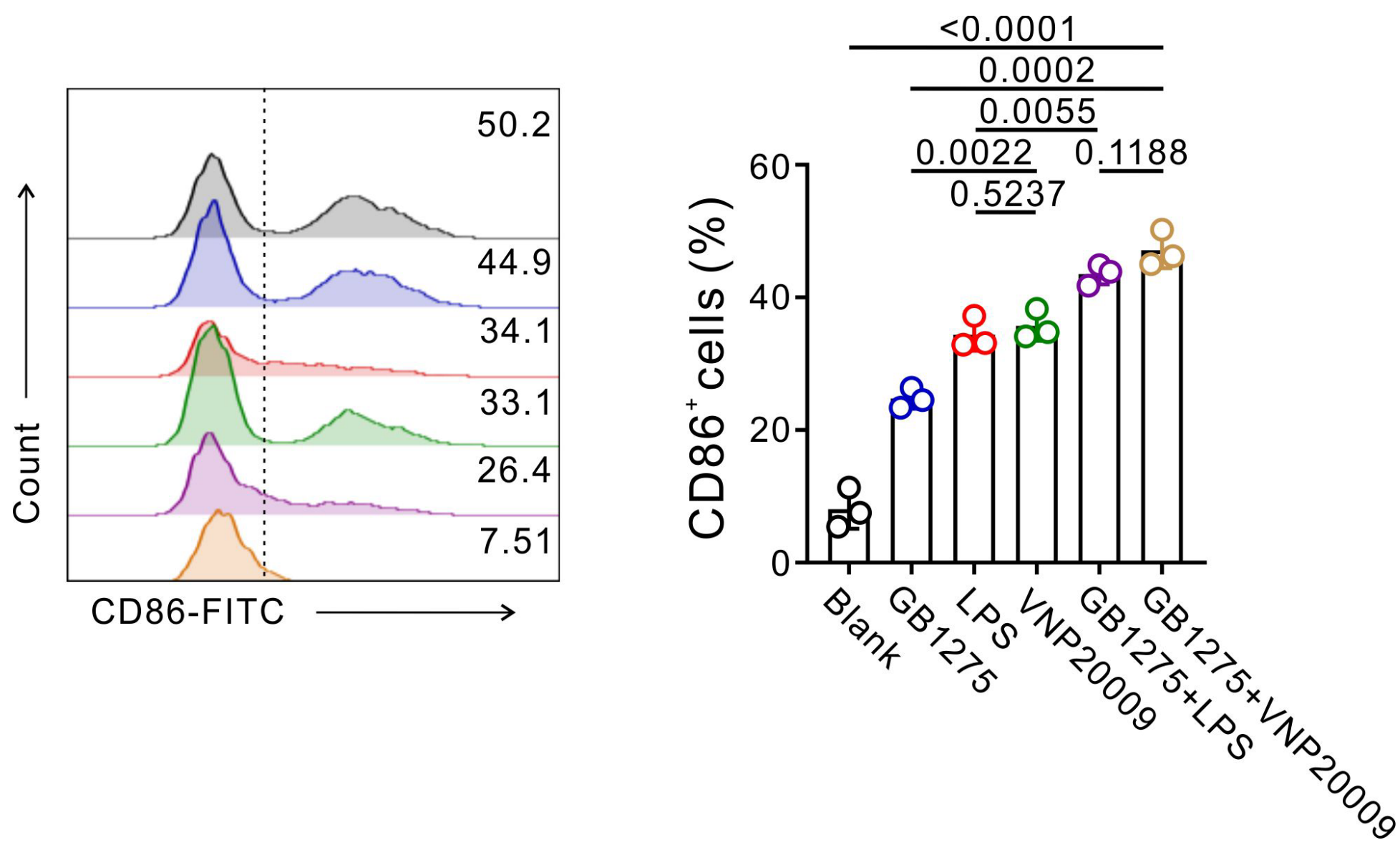

Supplementary Fig. 16 Flow cytometry analysis and corresponding statistics of $CD86^+$ expression levels after different treatments, n = 3 independent samples. The data are expressed as mean ± s.d. All replicates are biological, and statistical differences were calculated using two-tailed Student's t test.

Reviewer #3:

I co-reviewed this manuscript with one of the reviewers who provided the listed reports. This is part of the Nature Communications initiative to facilitate training in peer review and to provide appropriate recognition for Early Career Researchers who co-review manuscripts.

Response: We deeply appreciate your constructive comments on our experimental work. Your insights have immensely enriched the content of this article and expanded our intellectual horizons. In responses, uniformly use black font to mark the suggestions you provide, blue font to mark the reply, and red font to mark revisions made specifically to address the questions you raised.

Reviewer #4:

The manuscript presents an interesting and potentially impactful study exploring the use of oncolytic bacteria in combination with the CD11b-targeted agonist GB1275 to enhance anti-tumor immunity in PDAC. The topic is timely, and the study is generally well-conceived. However, several aspects of the experimental design, data presentation, and interpretation raise concerns about whether the data sufficiently support the authors' conclusions. Clarification and additional experimental evidence would substantially strengthen the manuscript.

Response: We deeply appreciate your constructive comments on our experimental work. Your insights have immensely enriched the content of this article and expanded our intellectual horizons. In responses, uniformly use black font to mark the suggestions you provide, blue font to mark the reply, and red font to mark revisions made specifically to address the questions you raised.

Major Comments:

1, Only one commercially available murine PDAC cell line (Panc01) was used in this study. Although the authors included cell lines from other cancer types to show the generalizability of the GB1275-bacteria combination, this approach does not adequately address the heterogeneity within PDAC itself. Given that PDAC comprises distinct molecular and phenotypic subtypes (e.g., epithelial vs. mesenchymal), it would be more informative to include additional PDAC cell lines representing these subtypes. Since Panc01 is mesenchymal, including epithelial-subtype PDAC models would provide valuable insights into how tumor subtype–specific TMEs influence treatment efficacy.

Response: Thank you very much for your valuable suggestions! When constructing the pancreatic cancer model using the Panc02 cell line in this study, we failed to fully consider the inherent heterogeneity of pancreatic ductal adenocarcinoma (PDAC). To address this issue, we further selected another murine pancreatic cancer model derived from the KPC cell line for subsequent studies, aiming to investigate whether the

inherent heterogeneity of pancreatic cancer impairs the therapeutic efficacy of this combination therapy. We systematically evaluated the tumor inhibitory effects of different treatment combinations on KPC tumors and conducted long-term dynamic follow-up (**Supplementary Fig. 34A**). During the treatment period, no significant fluctuations were detected in the body weight of the mice, indicating that this therapeutic regimen has favorable safety in the KPC tumor model (**Supplementary Fig. 34B**). The results demonstrated that the GB1275+Lip@V combination therapy still exerted robust tumor inhibitory activity in this model, with complete tumor regression observed in one tumor-bearing mouse (**Supplementary Fig. 34 C-G**). Furthermore, analyses of intratumoral immune responses showed that the intratumoral infiltration patterns of $CD11b^+$ cells in the KPC model were consistent with those in the Panc02 model (**Supplementary Fig. 34H**). Additionally, the GB1275+Lip@V group significantly increased the proportions of mature DCs (**Supplementary Fig. 34I**), $CD8^+$ T cells (**Supplementary Fig. 34J**), and M1-type macrophages (**Supplementary Fig. 34L, N**) in KPC tumor tissues, while markedly reducing the proportions of regulatory T cells (Tregs) (**Supplementary Fig. 34K**) and M2-type macrophages (**Supplementary Fig. 34L, N**). This suggests that the combination therapy can effectively reverse the immunosuppressive tumor microenvironment of KPC tumors and exert potent anti-tumor immune activation activity. Collectively, these results confirm that the GB1275+Lip@V combination therapy regimen holds great potential for application in a variety of pancreatic cancer models, and its therapeutic efficacy is not easily interfered with by the inherent heterogeneity of pancreatic cancer itself.

These revisions have been incorporated into the manuscript and highlighted in red on **Page 30**, with the corresponding **Supplementary Fig. 34** provided in the Supporting Information

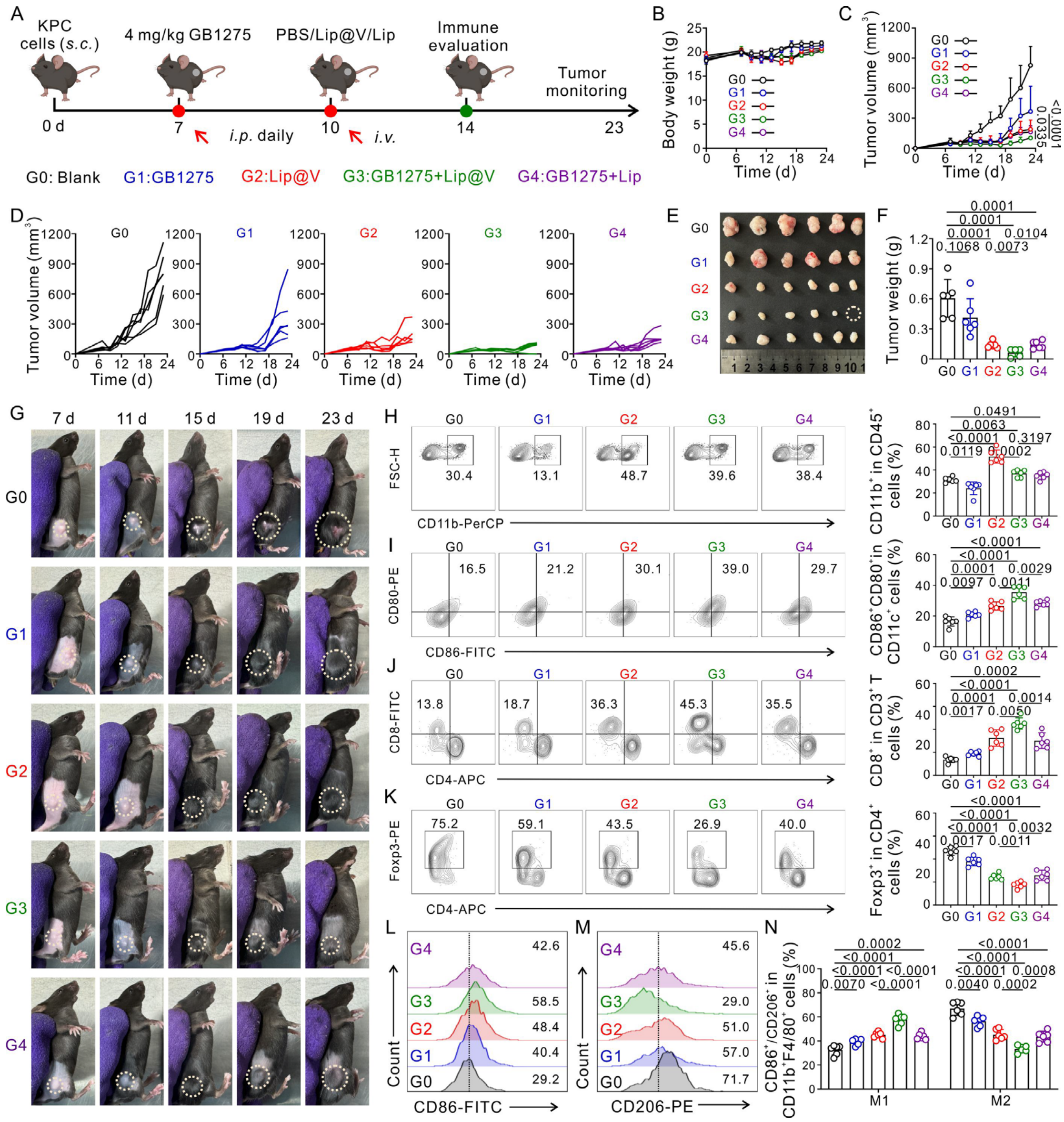

Supplementary Fig. 34 **A** Schematic illustration of the experimental protocols for the combination of GB1275 with drug-loaded bacteria for anti-KPC tumor therapy. *s.c.*, subcutaneous injection. *i.p.* daily, daily intraperitoneal injection. *i.v.*, intravenous injection. This figure was created with BioRender.com. **B** Changes in mouse body weights of mice after different treatments, n = 6 independent samples. **C, D** Tumor growth curves and individual tumor growth curves after different treatments. **E, F** Photographs and tumor weight analysis of the harvested tumors from different treatment groups, n = 6 independent samples. **G** Representative photographs of mice at different time points after different treatments. The dotted circle represents the tumor site. **H-K** Flow cytometry analysis and the corresponding statistics of $CD11b^+$ cells,

DCs, $CD8^+$ T cells and Treg cells infiltration in KPC tumor tissues after different treatments for 14 days， *n* = 6 independent samples. **L-N** Flow cytometry analysis and the corresponding statistics of the proportion of M1 and M2 cells in KPC tumor tissues after different treatments for 14 days, *n* = 6 independent samples. The data are expressed as mean ± s.d. All replicates are biological, and statistical differences were calculated using two-tailed Student's t test.

2. The treatment regimen begins when tumors reach approximately 70 mm^3, which represents a very early stage of tumor development. In clinical settings, PDAC is typically treated at a more advanced stage. Since the TME and stromal composition differ markedly between early and late-stage tumors, the therapeutic response may vary significantly. The authors should include data from experiments initiated at later tumor stages to better simulate clinical conditions.

Response: Thank you very much for your valuable suggestions! The initial therapeutic regimen in this study was initiated when the tumor volume reached approximately 70 mm^3, which differs substantially from the clinical diagnosis and treatment scenario of advanced pancreatic cancer and thus has limited simulation validity. Given the high malignancy and rapid progression of Panc02 tumor, this study adopted a tumor volume of approximately 150 mm^3 as the definition criterion for the advanced model, and initiated the predetermined treatment regimen at this time point. Specifically, the predetermined therapeutic regimen was initiated when the tumors reached this volume (**Supplementary Fig. 23**). Notably, experimental results demonstrated that, compared with other control and monotherapy groups (PBS, GB1275 monotherapy, Lip@V monotherapy, and GB1275+Lip group), the GB1275+Lip@V combination therapy exhibited significantly superior tumor growth inhibitory efficacy against advanced Panc02 pancreatic cancer. This finding suggests that the combination therapy regimen also possesses promising application potential and clinical translational value in the treatment of advanced pancreatic cancer.

These revisions have been incorporated into the manuscript and highlighted in red on

Page 23, with the corresponding **Supplementary Fig. 23** provided in the Supporting Information.

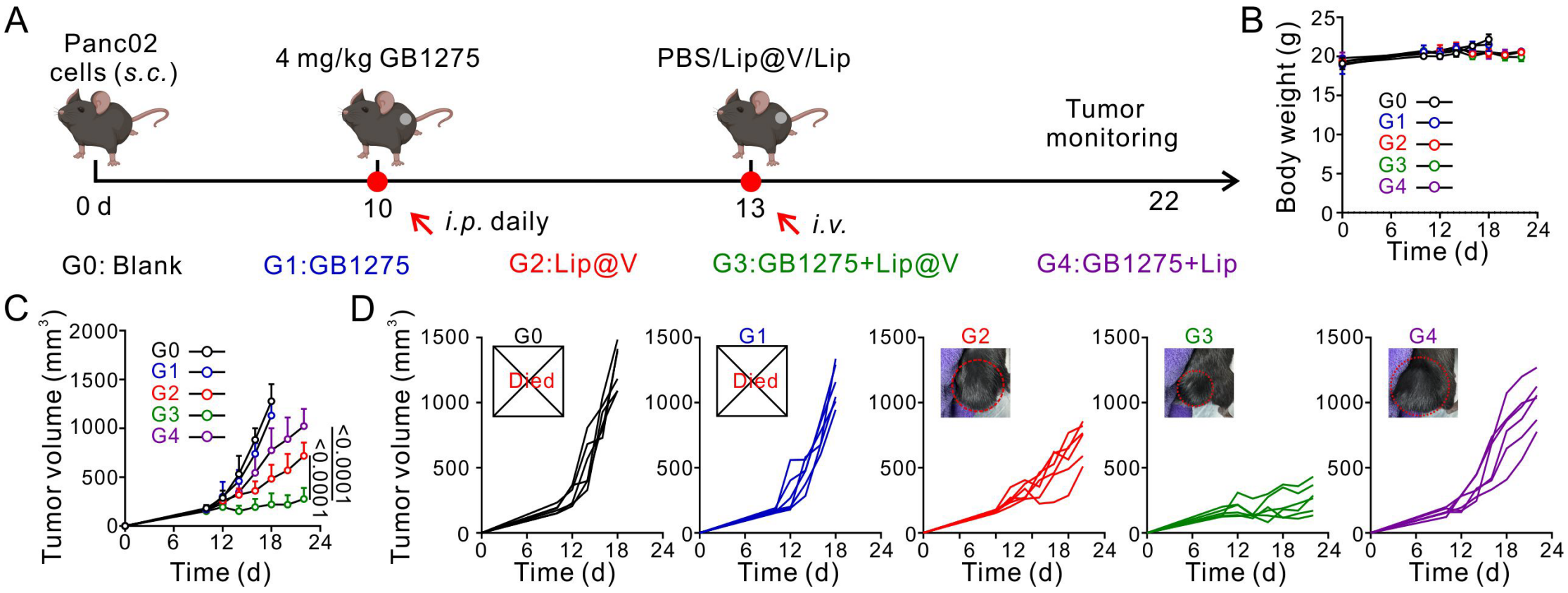

Supplementary Fig. 23 **A** Schematic illustration of the experimental protocols for GB1275-enhanced bacterial anti-tumor therapy for advanced tumors. *s.c.*, subcutaneous injection. *i.p.* daily, daily intraperitoneal injection. *i.v.,* intravenous injection. This figure was created with BioRender.com. **B** Changes in mouse body weights of mice after different treatments. **C, D** Tumor growth curves and individual tumor growth curves after different treatments. In Fig. D, the tumor images in the bar chart are representative images of each group of mice after the end of treatment. The term "Died" indicates that mice were humanely euthanized and recorded as deceased when tumor volume exceeded 1500 mm^3, in accordance with animal research ethical guidelines requiring the establishment of humane endpoints, n = 6 independent samples.

3. The authors claim that GB1275 synergizes with Lip@V-induced DNA damage to enhance IFNγ production and activate the cGAS-STING pathway. However, direct evidence demonstrating DNA damage upon Lip@V infection is missing. Measuring DAMP levels would substantiate this claim. Furthermore, the Western blot images are of poor quality and make it difficult to assess whether a synergistic effect truly exists between Lip@V alone and the combination treatment. Improved image quality and clearer quantification are necessary.

Response: We apologize sincerely for the misunderstanding caused by our

inappropriate expression earlier. We have revised the relevant content as follows and these revisions have been incorporated into the manuscript and highlighted in red on **Page 27**. Accordingly, the pertinent references have been included and highlighted in red in the reference list as entry 42.

Actually, in this study, GB1275 and oncolytic bacteria activate the STING signaling pathway through distinct mechanisms, rather than by inducing DNA damage. Early studies by Maiguel et al. have reported that GB1275 can inhibit leukocyte recruitment by activating the CD11b/CD18 pathway, a strategy initially focused on the treatment of mice with nephritis. Subsequently, Panni et al. extended the research on GB1275 to the field of tumor immunology, confirming that it can simultaneously inhibit NF-κB signaling and activate interferon gene expression, thereby regulating the phenotype of tumor-associated macrophages (TAMs). This finding reveals the potential value of GB1275 as a STING agonist and provides a theoretical basis for its combined application with various therapeutic modalities. (*Cancer Cell* 41, 1073-1090 (2023))

In contrast, the mechanism by which oncolytic bacteria activate STING differs: as exogenous microorganisms, oncolytic bacteria continuously secrete extracellular DNA (eDNA) during proliferation. This eDNA, serving as a pathogen-associated molecular pattern (PAMP), can be specifically recognized by STING receptors in host cells, ultimately inducing the expression of type I interferons. This strategy has been applied in tumor therapy research; for example, Yang et al. enhanced the sensitivity of cGAS in dendritic cells to bacterially secreted eDNA via Mn^{2+}, achieving effective inhibition of tumor growth. (*Adv. Mater*. 36, 2310189 (2024))

Based on the above research background, this study further explores the potential ability of GB1275 and eDNA secreted by Lip@V to synergistically activate the STING pathway in tumor tissues.

We have reperformed the Western blot experiment. It can be clearly determined that there is a synergistic effect between the Lip@V monotherapy group and the combination treatment group.

These revisions have been incorporated into the manuscript and highlighted in red on **Page 28**, with the corresponding **Fig. 6** presented on **Page 59**.

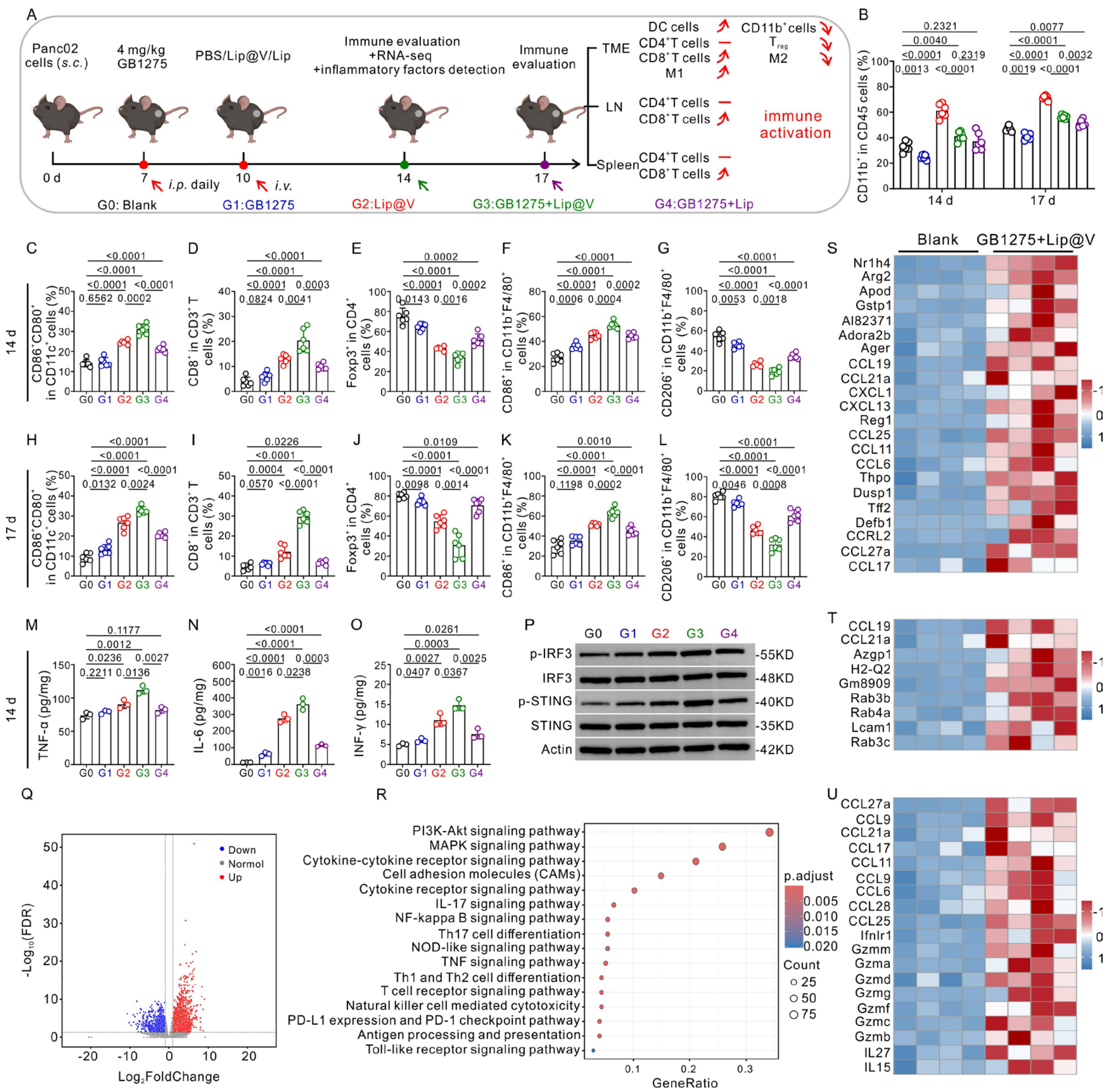

Fig. 6 The combination of GB1275 with drug-loaded bacteria profoundly reshapes the pancreatic cancer immunosuppressive microenvironment and activates adaptive immunity. A Schematic illustration of the experimental protocols for in vivo immune responses triggered by GB1275+Lip@V. *s.c.*, subcutaneous injection. *i.p.* daily, daily intraperitoneal injection. *i.v.*, intravenous injection. TME represents tumor microenvironment; LN represents lymph nodes. The red arrows indicate an increase or decrease in the proportion of cells. The red horizontal line indicates no change in the proportion of cells. This figure was created with BioRender.com. **B** $CD11b^+$ cell infiltration in tumor tissues after different treatments. Corresponding flow cytometry

analysis statistics of DC cells, CD8⁺ T cells, Treg cells infiltration in tumor tissues after different treatments on **(C-E)** day 14 and **(H-J)** day 17, *n* = 6 independent samples. Corresponding flow analysis statistics of the proportion of M1 and M2 cells in tumor tissue after different treatments on **(F, G)** day 14 and **(K, L)** day 17, *n* = 6 independent samples. **M-O** The secretion levels of TNF-α, IL-6, and IFN-γ in tumor tissues after different treatments were detected by ELISA, *n* = 3 independent samples. **P** Representative immunoblot for p-STING, STING, p-IRF3, IRF3, and Actin (loading control) in tumor tissues after different treatments for 14 days. On day 14 after treatment, untreated (blank) and GB1275+Lip@V-treated tumor blocks were taken for transcriptome sequencing, *n* = 5 independent samples. **Q** Volcano plot for identifying differentially expressed genes before and after GB1275+Lip@V treatment. The overall distribution of regulated genes is represented by a volcano plot, in which the abscissa and ordinate represent the fold change (FC) of gene expression in different samples and statistical significance (*p* value) of the difference in gene expression, respectively. The red, blue, and grey dots represent the upregulated, downregulated, and no significant changes of genes, respectively. **R** The enrichment scatter plot to show KEGG enrichment analysis of a selection of immune-related KEGG terms. Gene ratio is the ratio of the number of differential genes to the total number of genes in the pathway. **S** Heat map showing *z*-scores of upregulated individual genes in Cytokine-related gene expression KEGG pathway in Q and R. **T** Heatmap showing *z*-scores of upregulated individual genes expression in antigen processing and presentation-related gene expression KEGG pathway in Q and R. **U** Heatmap of genes involved in T cell recruitment, proliferation, activation, and cytotoxic activity after GB1275+Lip@V treatment. Heat map data are shown as the mean $\log_2$ fold change z-scores. The data are expressed as mean ± s.d. All replicates are biological, and statistical differences were calculated using two-tailed Student's t test.

4. GB1275 is described as an inhibitor of CD11b⁺ cell infiltration; however, it also appears to promote M1 macrophage polarization (Figures 4N-O, S23). The underlying

mechanism of this dual effect is not clearly explained. Additional mechanistic discussion or supporting data would help reconcile this observation.

Response: Thank you very much for your valuable suggestions! In this study, GB1275 regulates macrophage function through two targeted mechanisms, ultimately synergizing with oncolytic bacteria to enhance anti-tumor efficacy. The details are as follows:

1. Inhibiting the migration of macrophages recruited by oncolytic bacteria to preserve bacterial activity.

GB1275 ensures the anti-tumor efficacy of oncolytic bacteria by suppressing macrophage migration. Although oncolytic bacteria can recruit macrophages after colonizing tumor sites, the inherent phagocytic function of macrophages directly kills oncolytic bacteria, thereby impairing their therapeutic potential. Therefore, we used GB1275 to block the massive infiltration of macrophages into tumors induced by oncolytic bacteria, indirectly enhancing the intratumoral survival and activity of oncolytic bacteria.

2. Reprogramming pancreatic cancer-resident TAMs into anti-tumor phenotypes to initiate innate immunity.

In the immunosuppressive microenvironment of pancreatic cancer, which is rich in tumor-associated macrophages (TAMs), macrophages are ideal targets for initiating local innate immune responses. Studies have shown that GB1275 can activate the STING pathway via FAK-mediated mitochondrial dysfunction, inducing the expression of type I interferon-related genes; whereas oncolytic bacteria can also release cyclic dinucleotides to directly activate STING signaling in macrophages. Thus, their combination can reprogram the originally immunosuppressive TAMs in pancreatic cancer tissues into macrophages with anti-tumor functions, thereby effectively triggering the intratumoral innate immune response.

5. The resolution of Figure 7H is too low to discern $CD8^+$ signals, making it difficult to

validate the immunofluorescence findings and correlate them with the flow cytometry results shown in Figures 7I-K. Furthermore, the reported percentages of IFNγ^{+} and GZMB^{+} CD8^{+} cells appear inconsistent with the total CD3^{+}CD8^{+} population. The authors should clarify whether the percentages in Figures 7J-K represent proportions within the CD3^{+}CD8^{+} subset or the total tumor cell population.

Response: Thank you very much for your valuable suggestions! We have re-performed the experiments in this section and strictly followed the standardized gating strategy: first, we sequentially defined the cell populations of total cells, single cells, viable cells, and CD45^{+} immune cells, followed by further gating and sorting for the CD45^{+} cell population. Meanwhile, fluorescence minus one (FMO) controls and single-stain controls were employed in all experiments to ensure the accuracy of gating boundaries. Furthermore, we have clearly specified the calculation reference for the positive cell percentages in each flow cytometry analysis plot in the manuscript. After strictly adhering to the flow cytometry gating criteria and standard protocols, the discrimination of each cell population in the plots has been markedly enhanced, yielding clearer, more intuitive results that are fully consistent with our experimental expectations.

These revisions have been incorporated into the manuscript and highlighted in red **on Page 32**, with the corresponding **Fig. 7** presented on **Page 63**.

For the TUNEL, Ki67, and CD8^{+} T cell immunofluorescence staining included in **Figure 7**, we have magnified the key regions to present the staining details more clearly, and all the corresponding magnified images are incorporated into the original figure. As observed in the magnified images of Panels F and G, the TUNEL and Ki67 staining signals were highly colocalized with DAPI (a nuclear marker), indicating that these signals are primarily localized in the cell nucleus. The fluorescent signals of CD8^{+} T cells can be clearly visualized in the magnified image of Panel H. In addition, to precisely localize CD8^{+} T cells, white triangular arrows have been added to the magnified images, which more distinctly highlight the positions of CD8^{+} T cells. These revisions have been incorporated into the manuscript and highlighted in red on **Page 32**, with the corresponding **Fig. 7** presented on **Page 63**.

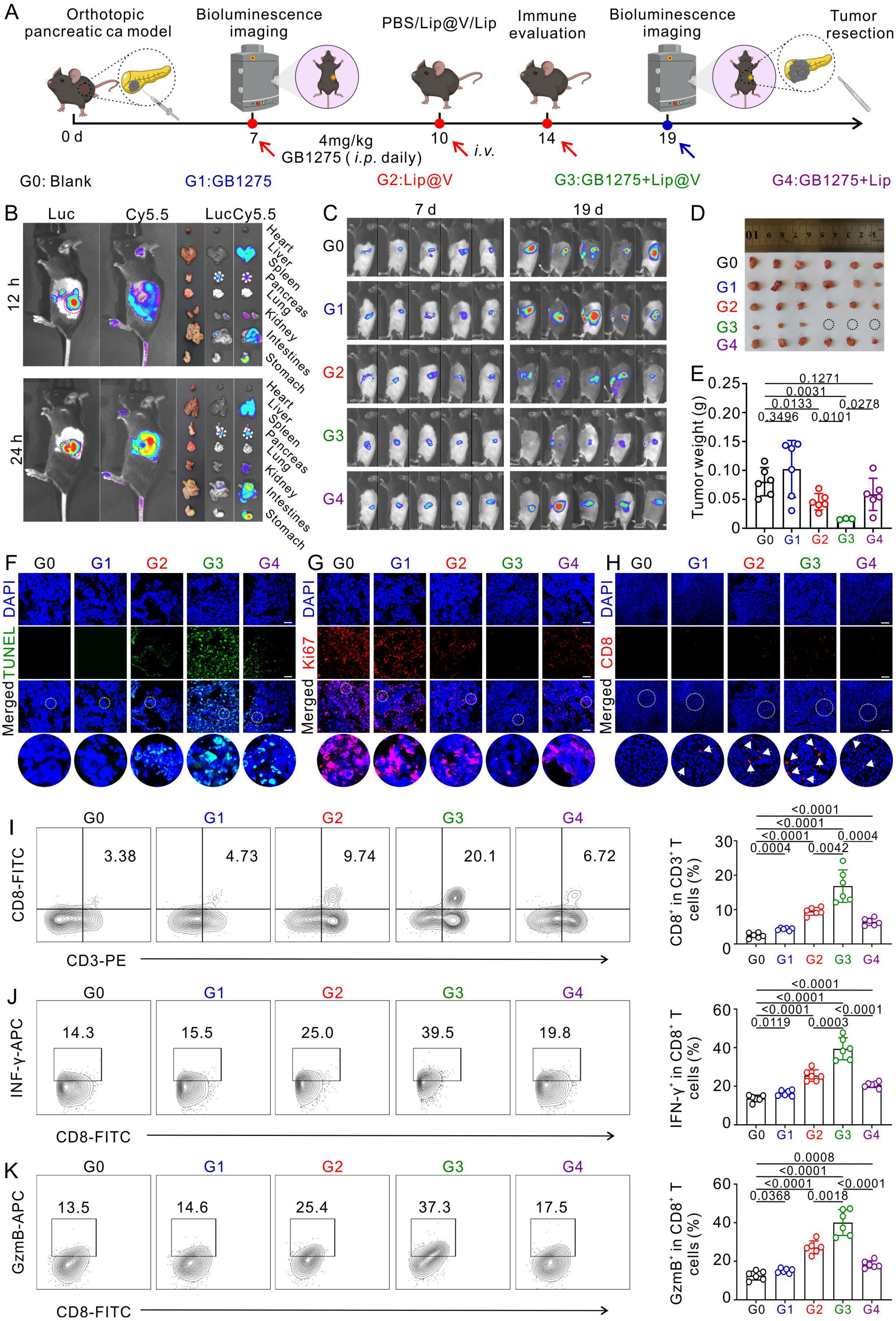

Fig.7 The combination of GB1275 with drug-loaded bacteria significantly inhibits

the progression of orthotopic pancreatic cancer. A Schematic illustration of the experimental protocols for the combination therapy of GB1275 and drug-loaded bacteria in the treatment of in situ pancreatic cancer. *s.c.*, subcutaneous injection. *i.p.* daily, daily intraperitoneal injection. *i.v.*, intravenous injection. This figure was created with BioRender.com. **B** Representative fluorescence imaging combined with bioluminescence imaging co-localizes the enrichment of Lip@V in orthotopic pancreatic cancer at different time points post-administration. The figure includes whole-body fluorescence imaging and whole-body bioluminescence imaging of the in situ pancreatic cancer model, white light images of major organs and tissues, as well as fluorescence imaging and bioluminescence imaging. The fluorescent signals originate from Cy5.5-labeled Lip@V, while the bioluminescence imaging results from light emission produced by Panc02-luc cells catalyzing the substrate. **C** In vivo bioluminescence imaging of in situ pancreatic cancer after different treatments, $n = 5$ independent samples. **D, E** Photographs and tumor weight analysis of the harvested tumors from different treatment groups, $n = 6$ independent samples. **F, G** Representative images of TUNEL and Ki67 immunofluorescence staining of tumor tissues after different treatments. Scale bar: 200 μm. H Representative images of $CD8^+$ T cells immunofluorescence staining of tumor tissue after different treatments. To clearly localize the target cells, white triangular arrows were used in the figure to more explicitly highlight the T cells. Scale bar: 200 μm. **I-K** Flow cytometry analysis and the corresponding statistics of $CD8^+$ T cells, IFN-γ-secreting $CD8^+$ T cells, and GzmB-secreting $CD8^+$ T cells infiltration in tumor tissues after different treatments for 14 days, $n = 6$ independent samples. The data are expressed as mean ± s.d. All replicates are biological, and statistical differences were calculated using two-tailed Student's t test.

6. Some in vitro experiments, though technically sound, contribute limited mechanistic insight. For instance, using spheroid models to demonstrate the inhibitory effect of GB1275 on $CD11b^+$ cell infiltration may not accurately reflect in vivo conditions, as spheroids lack key components of the tumor microenvironment such as extracellular

matrix architecture and vasculature. This section appears of limited value and could be condensed or omitted.

Response: We sincerely appreciate your valuable suggestions and fully agree with your viewpoint! Indeed, in vitro tumor spheroid organoids lack the extracellular matrix architecture and vascular system present in the tumor microenvironment, and both components play a crucial role in the mediation of $CD11b^+$ cell adhesive function by GB1275.

The core original intention of designing this tumor spheroid organoid experiment was to verify the inhibitory effect of GB1275-enhanced adhesiveness on the migration of $CD11b^+$ cells in vitro, thereby presenting the migration process of $CD11b^+$ cells toward tumor spheroids in a more intuitive manner. To this end, we made targeted designs for the reagents and procedures of the in vitro organoid experiment. To mimic the key role of extracellular matrix-mediated adhesion, the bottoms of 96-well plates were pre-modified with fibrinogen prior to the recruitment assay, so as to simulate the adhesive microenvironment of $CD11b^+$ cells provided by the extracellular matrix and vascular system. (*J. Biomol. Screen.* 12, 406-417 (2007).) The detailed experimental procedure is as follows: First, $CD11b^+$ cells (including RAW264.7 macrophages and neutrophils isolated from mouse peripheral blood) were seeded into the 96-well plates modified with fibrinogen. Subsequently, 2 μM GB1275 was added to the experimental groups requiring GB1275 treatment, and the mixture was incubated statically for 30 min after mixing with $CD11b^+$ cells. Gravitational force was utilized to promote sufficient contact between $CD11b^+$ cells and the plate bottoms for enhanced adhesion; finally, tumor spheroids and bacterium-colonized tumor spheroids were added to initiate the experiment. Therefore, the core objective of this experiment was to mimic the extracellular matrix and vascular system with bovine fibrinogen in vitro, so as to clarify the mediating effect of GB1275 on the adhesive function of $CD11b^+$ cells, and this experiment holds certain scientific significance.

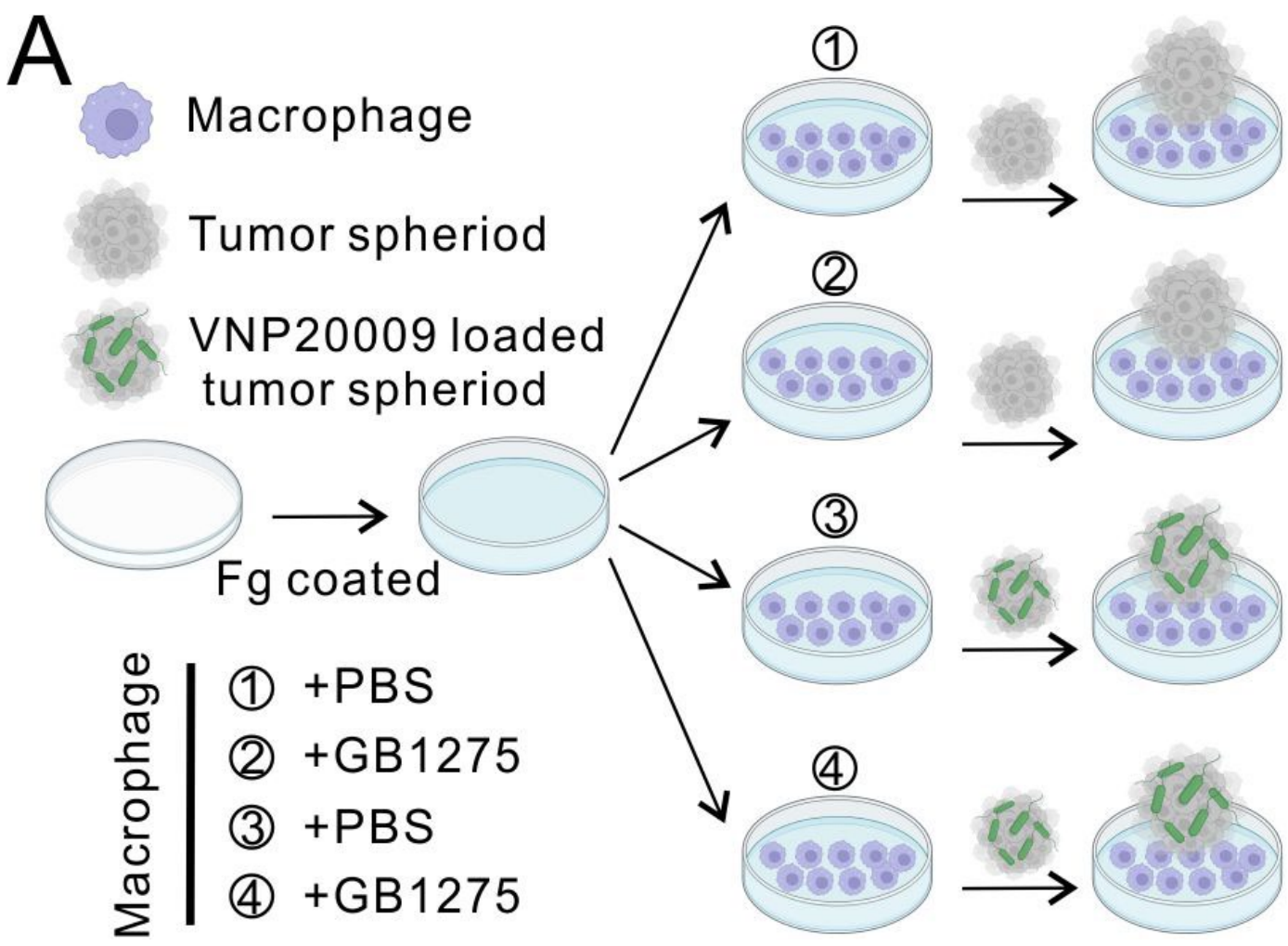

This figure is a schematic diagram of **Figure 3A**, where Fg is the abbreviation for fibrinogen. It is used to mimic the extracellular matrix and vascular system, thereby facilitating GB1275 to promote the adhesion of $CD11b^+$ cells.

Minor Comments

7, The Results section contains extensive background information and data interpretation, which makes it difficult to follow the core findings. The authors should streamline this section to focus more on experimental observations.

Response: Thank you very much for your valuable suggestions! We have optimized the Results section of the paper, simplified the redundant background information therein and deleted the content with low relevance to the core conclusions of this study. Meanwhile, we have further strengthened the in-depth analysis and focused discussion of the experimental results of this study, shifting the primary focus more onto the discussion of the experimental results. These revisions have been incorporated into the Discussion of manuscript and highlighted in red on **Page 34**.

8, Numerous grammatical and typographical errors are present throughout the manuscript. Careful proofreading is required to improve readability and professionalism.

Response: Thank you very much for your valuable suggestions! We have conducted a

comprehensive check of the entire manuscript, revised all grammatical and formatting errors one by one, and effectively improved the readability and professionalism of the manuscript. These revisions have been incorporated into the manuscript and highlighted in red.

Response to referees' comments

Reviewer #1:

The authors have satisfied my previous concerns in admirable fashion with one exception. The authors stated they used inhibitors of CCR2 and PI3Kgamma as alternative methods of suppressing CD11b+ cell adhesion. However, their stated PI3Kγ inhibitor 3-Methyladenine is not a PI3Kγ inhibitor but rather a PI3kinase class III (Vps34) inhibitor, which inhibits autophagy. PI3K gamma is class I PI3K, which regulates adhesion, chemotaxis and transcription, while PI3kinase class III (Vps34) regulates sequestration of cytoplasmic material in lysosomes during autophagy. The authors should correct the description of these data or eliminate the data altogether.

Response: We deeply appreciate your constructive comments on our experimental work! We fully agree with your precise and rigorous comment, and we apologize for the errors present in our original manuscript. We mistakenly categorized 3-methyladenine (3-MA) as a PI3Kγ inhibitor and falsely interpreted its function as suppressing $CD11b^+$ cell adhesion. Consistent with your clarification, 3-MA acts as a class III PI3K (Vps34) inhibitor to block autophagy, whereas PI3Kγ belongs to class I PI3K and mediates cell adhesion, chemotaxis and transcription. Considering the mismatch between the reagent and our initial experimental objective, we have entirely deleted all 3-MA-related content from the revised manuscript, including corresponding figures, quantitative data and relevant discussion paragraphs, to eliminate misleading information for readers. All results and discussion regarding the CCR2 inhibitor are fully retained and refined to independently support our conclusion about $CD11b^+$ myeloid cell recruitment. We have thoroughly checked the entire manuscript to guarantee no leftover inaccurate descriptions of 3-MA exist.

These revisions have been incorporated into the manuscript and highlighted in red on **Page 14**, with the corresponding **Supplementary Fig. 13** provided in the Supporting Information.

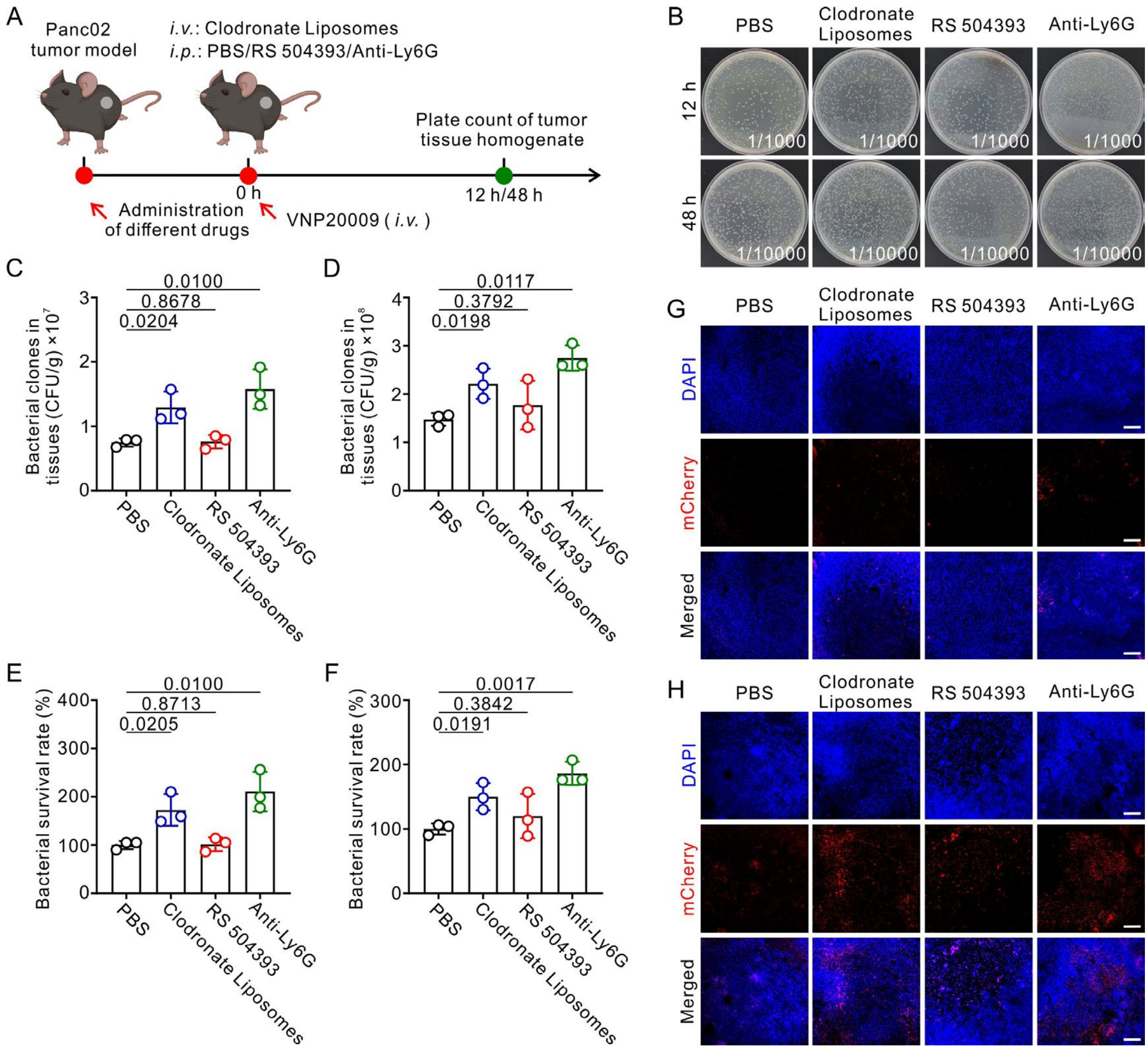

Supplementary Fig. 13 A Schematic illustration of the in vivo administration regimen of different drugs and VNP20009 in Panc02 subcutaneous tumor model. *i.p.*, intraperitoneal injection. *i.v.*, intravenous injection. This figure was created with BioRender.com. (B-D) Representative photographs and corresponding quantification of bacterial colonization in tumors obtained from Panc02 tumor-bearing mice at different time points (C: 12 h and D: 48 h) after different treatments, $n = 3$ independent samples. Bacterial survival rates in tumor tissues of different groups at (E) 12 h and (F) 48 h, $n = 3$ independent samples. Representative images of mCherry-VNP20009 immunofluorescence staining of tumor tissues after different treatments at (G) 12 h and (H) 48 h. Scale bar: 200 μm. The data are expressed as mean ± s.d. All replicates are biological, and statistical differences were calculated using two-tailed Student's t test.

Reviewer #4:

The authors have revised the manuscript and addressed most of the concerns. However, one point remains: the response adds a KPC-derived PDAC model but does not fully address the concern regarding subtype heterogeneity. While Panc02 is assumed to be mesenchymal-like, no data are provided, especially for the KPC-derived model, to confirm their molecular or phenotypic subtypes. Without such characterization and without inclusion of epithelial-subtype PDAC models, it remains unclear whether the GB1275+Lip@V combination therapy is effective across the spectrum of PDAC heterogeneity. I recommend that the authors provide subtype characterization for their models and/or include epithelial-subtype PDAC models to strengthen the conclusions.

Response: We deeply appreciate your constructive comments on our experimental work! we sincerely appreciate your valuable comments that our study lacked molecular subtype verification for the Panc02 and KPC pancreatic cancer models, and the absence of an independent epithelial-subtype model made it difficult to verify whether the GB1275+Lip@V combination therapy works against heterogeneous pancreatic ductal adenocarcinoma. Based on this concern, we performed Western blot detection of EMT markers on KPC and Panc02 cell lines as well as their derived subcutaneous tumor tissues, and further supplemented immunohistochemical staining of E-cadherin and Vimentin on tumor specimens. We combined three layers of evidence including purified cultured tumor cells, in vivo tumor lysate Western blot and in situ tissue IHC staining to mutually validate the two classic epithelial and mesenchymal PDAC subtypes, fully addressing the concerns regarding subtype heterogeneity.

We simultaneously detected the protein levels of epithelial marker E-cadherin and mesenchymal markers N-cadherin and Vimentin. Without stromal cell interference, distinct differences in molecular profiles were observed at the cellular level: Panc02 cells showed low E-cadherin expression and high levels of N-cadherin and Vimentin, exhibiting a molecular profile leaning more toward a mesenchymal-like phenotype; KPC cells exhibited robust E-cadherin expression with barely detectable mesenchymal markers, presenting a profile more inclined to a typical epithelial-like phenotype.

Abundant vimentin-positive cancer-associated fibroblasts within solid tumors dilute specific signals from malignant cells, leading to milder marker differences between two tumor groups compared with cellular samples, yet the expression trends of E-cadherin and N-cadherin in tumor lysates were consistent with cellular results. We further conducted IHC staining for E-cadherin and Vimentin on tumor tissues, and the in situ staining results matched the WB data perfectly: KPC tumor tissues displayed markedly stronger E-cadherin positive staining than Panc02 tumors, while comparable Vimentin signals were observed in both groups. This phenomenon arises from ubiquitous vimentin expression in stromal fibroblasts, which masks intrinsic vimentin differences in tumor cells and does not interfere with the judgment of subtype tendency.

The results from cellular Western blot, tumor tissue Western blot and tumor immunohistochemistry were highly consistent across all three detection systems, confirming that the molecular profile of the KPC model leans more toward epithelial-subtype PDAC, while the Panc02 model leans more toward mesenchymal-subtype PDAC. The GB1275+Lip@V combination regimen exerted prominent anti-tumor effects in both PDAC models with distinct EMT tendencies, covering the major heterogeneous subtypes driven by epithelial-mesenchymal transition. These data collectively demonstrate the broad therapeutic potential of our combinatorial treatment. We have supplemented all supporting subtype characterization data including cellular WB, tumor tissue WB and IHC images together with detailed analysis in the revised manuscript.

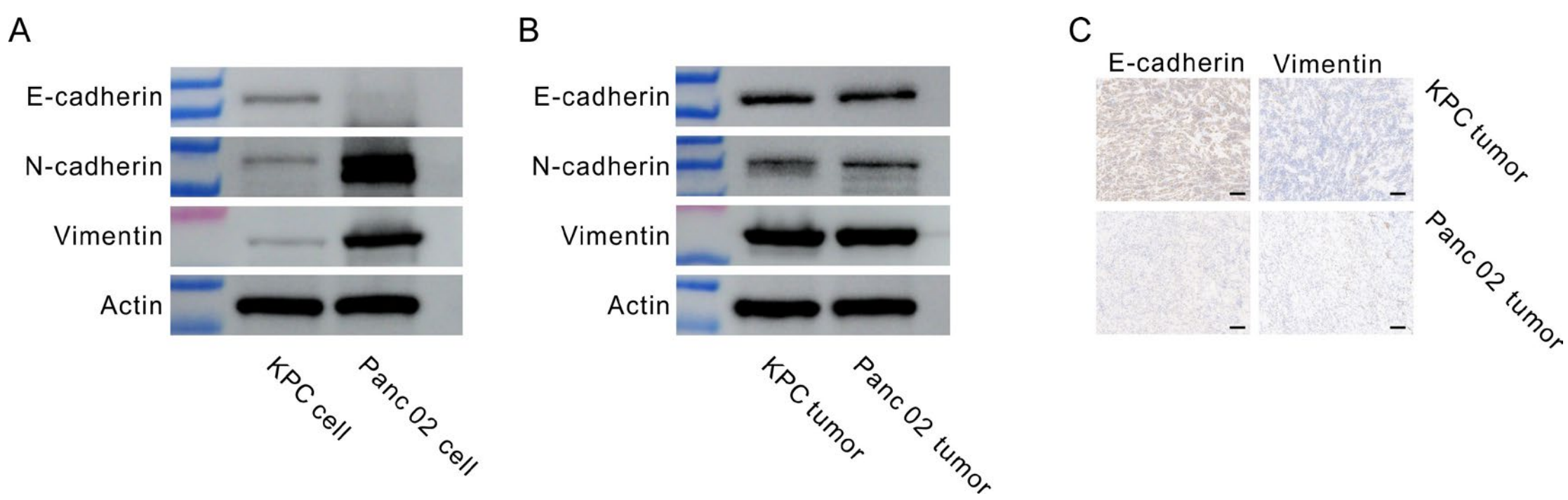

Supplementary Fig. 34 Representative immunoblot of E-cadherin, N-cadherin,

Vimentin and actin (loading control) in **(A)** KPC/Panc02 cells as well as **(B)** subcutaneous tumor tissues derived from the two cell lines. Representative IHC staining of E-cadherin and Vimentin in KPC and Panc02 tumor tissues. Scale bar: 100 μm.

The above results confirmed that the GB1275 + Lip@V combination therapy regimen exerts remarkable anti-tumor immunotherapeutic efficacy in the Panc02 pancreatic cancer model. However, it should be noted that different pancreatic ductal adenocarcinomas exhibit inherent heterogeneity, a characteristic that may compromise the universality of the therapeutic regimen. Pancreatic ductal adenocarcinoma is frequently accompanied by epithelial-mesenchymal transition (EMT). Tumors of distinct EMT subtypes possess markedly divergent invasive and metastatic potential, which may greatly compromise the broad-spectrum anti-tumor efficacy of therapeutic regimens. To investigate the impact of tumor heterogeneity on the therapeutic efficacy of the GB1275 + Lip@V combination therapy, we further introduced the KPC-derived mouse PDAC model for parallel validation. We first utilized the epithelial marker E-cadherin and mesenchymal markers N-cadherin and Vimentin to characterize the EMT subtypes of the two murine pancreatic cancer models via Western blot analysis of cell lines and tumor tissues, as well as immunohistochemical staining of tumor specimens. Experimental results revealed obvious differences in marker expression between the two cell lines at the cellular level **(Supplementary Fig. 34A, B)**. Panc02 cells exhibited low E-cadherin expression along with high levels of N-cadherin and Vimentin, displaying a molecular profile leaning toward a mesenchymal-like phenotype. In

contrast, KPC cells showed abundant E-cadherin expression and weak signals of mesenchymal markers, consistent with an epithelial-like phenotype. Western blot results of tumor tissues demonstrated that the expression trends of E-cadherin and N-cadherin were fully consistent with those observed in cellular experiments. However, no significant intergroup difference in Vimentin expression was detected in tumor tissues. This is mainly attributed to the widespread expression of Vimentin by abundant cancer-associated fibroblasts within the tumor microenvironment, which masks the intrinsic differences in Vimentin expression in tumor cells between the two models. We further performed immunohistochemical staining on tumor tissues, and the staining results were consistent with the tumor tissue Western blot data **(Supplementary Fig. 35C)**. E-cadherin expression was remarkably higher in KPC tumor tissues than in Panc02 tumor tissues, while comparable Vimentin levels were observed in both groups. Collectively, results from multiple layers of experiments confirmed that the KPC model leans more toward the epithelial-like pancreatic cancer subtype, whereas the Panc02 model leans more toward the mesenchymal-like pancreatic cancer subtype.

These revisions have been incorporated into the manuscript and highlighted in red on **Page 30**, with the corresponding **Supplementary Fig. 34** provided in the Supporting Information.

Reviewer #5:

The authors have carefully addressed the questions raised by Reviewer #2. I only have two minor suggestions for clarification:

Response: We deeply appreciate your constructive comments on our experimental work. Your insights have immensely enriched the content of this article and expanded our intellectual horizons. In responses, uniformly use black font to mark the suggestions you provide, blue font to mark the reply, and red font to mark revisions made specifically to address the questions you raised.

Please add the methods of drug delivery to the schematic in Supplementary Figure 13.

Response: Thank you very much for your valuable suggestions! We have supplemented detailed administration information of each drug into the schematic diagram. Corresponding schematic diagram has been supplemented and shown in **Supplementary Figures S13**.

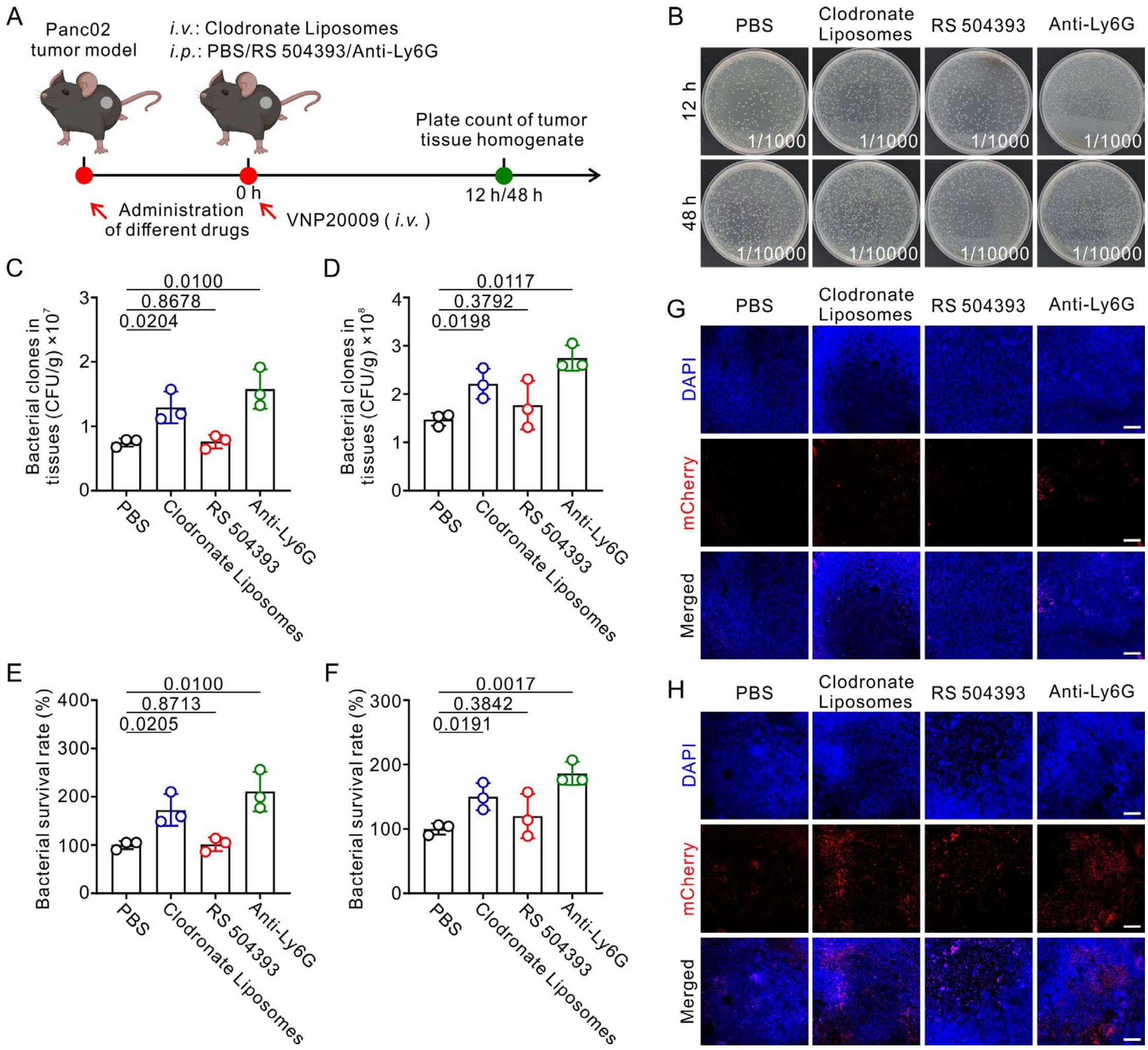

Supplementary Fig. 13 A Schematic illustration of the in vivo administration regimen of different drugs and VNP20009 in Panc02 subcutaneous tumor model. *i.p.*, intraperitoneal injection. *i.v.*, intravenous injection. This figure was created with BioRender.com. (B-D) Representative photographs and corresponding quantification of bacterial colonization in tumors obtained from Panc02 tumor-bearing mice at different time points (C: 12 h and D: 48 h) after different treatments, n = 3 independent samples. Bacterial survival rates in tumor tissues of different groups at (E) 12 h and (F) 48 h, n = 3 independent samples. Representative images of mCherry-VNP20009 immunofluorescence staining of tumor tissues after different treatments at (G) 12 h and (H) 48 h. Scale bar: 200 μm. The data are expressed as mean ± s.d. All replicates are biological, and statistical differences were calculated using two-tailed Student's t test.

Please specify the type of cells analyzed in Supplementary Figure 16.

Response: Thank you very much for your valuable suggestions! We have revised the ambiguous descriptions in the manuscript and supplemented a schematic diagram of the experimental procedure to better illustrate our experimental design. Corresponding schematic diagram has been supplemented and shown in **Supplementary Figures S16**.

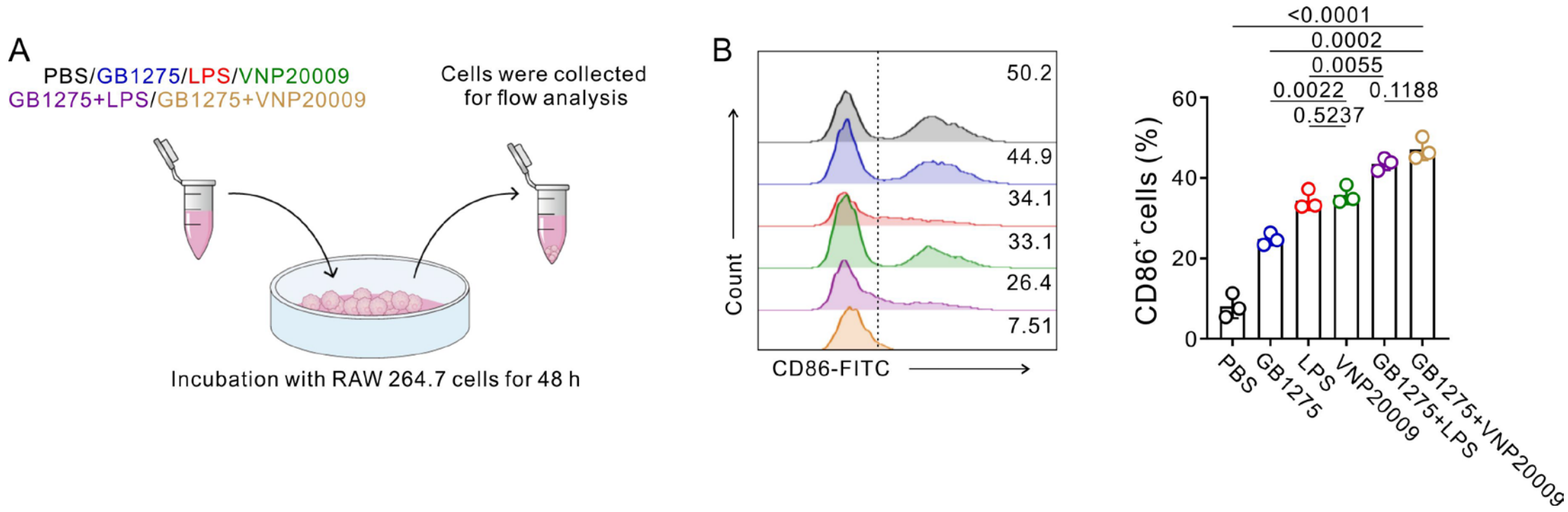

Supplementary Fig. 16 A Schematic illustration of the experimental protocols for macrophage polarization in vitro. B Flow cytometry analysis and corresponding statistics of $CD86^+$ expression levels after different treatments, n = 3 independent samples. The data are expressed as mean ± s.d. All replicates are biological, and statistical differences were calculated using two-tailed Student's t test.

Response to referees' comments

Reviewer #1:

The authors have satisfied my previous concerns.

Response: We sincerely appreciate your careful reading of our manuscript and all the precious comments you offered.

Reviewer #4:

The authors have revised the manuscript and addressed my concerns, I'm satisfied with this version.

Response: We are truly grateful for your detailed evaluation and thoughtful suggestions on this work.

Reviewer #5:

The authors have addressed all my comments and concerns.

Response: We would like to express our sincere gratitude for your professional review and constructive feedback.
